# Comparative Genomics of the Waterfowl Innate Immune System

**DOI:** 10.1093/molbev/msac160

**Published:** 2022-07-26

**Authors:** Elinor Jax, Paolo Franchini, Vaishnovi Sekar, Jente Ottenburghs, Daniel Monné Parera, Roman T Kellenberger, Katharine E Magor, Inge Müller, Martin Wikelski, Robert H S Kraus

**Affiliations:** Department of Migration, Max Planck Institute of Animal Behavior, Radolfzell, Germany; Department of Biology, University of Konstanz, Konstanz, Germany; Department of Veterinary Medicine, University of Cambridge, Cambridge, United Kingdom; Department of Biology, University of Konstanz, Konstanz, Germany; Department of Biology and Biotechnologies “Charles Darwin”, Sapienza University, Rome, Italy; Department of Biology, Lund University, Lund, Sweden; Department of Molecular Biosciences, The Wenner-Gren Institute, Stockholm University, Sweden; Wildlife Ecology and Conservation Group, Wageningen University, Wageningen, The Netherlands; Forest Ecology and Forest Management Group, Wageningen University, Wageningen, The Netherlands; Department of Biology, University of Konstanz, Konstanz, Germany; Department of Plant Sciences, University of Cambridge, Cambridge, United Kingdom; Department of Biological Sciences and Li Ka Shing Institute of Virology, University of Alberta, Edmonton, Canada; Department of Migration, Max Planck Institute of Animal Behavior, Radolfzell, Germany; Department of Biology, University of Konstanz, Konstanz, Germany; Department of Migration, Max Planck Institute of Animal Behavior, Radolfzell, Germany; Centre for the Advanced Study of Collective Behaviour, University of Konstanz, Konstanz, Germany; Department of Migration, Max Planck Institute of Animal Behavior, Radolfzell, Germany; Department of Biology, University of Konstanz, Konstanz, Germany

**Keywords:** DNA polymorphism, genetic divergence, natural selection, mallard, Anatidae

## Abstract

Animal species differ considerably in their ability to fight off infections. Finding the genetic basis of these differences is not easy, as the immune response is comprised of a complex network of proteins that interact with one another to defend the body against infection. Here, we used population- and comparative genomics to study the evolutionary forces acting on the innate immune system in natural hosts of the avian influenza virus (AIV). For this purpose, we used a combination of hybrid capture, next- generation sequencing and published genomes to examine genetic diversity, divergence, and signatures of selection in 127 innate immune genes at a micro- and macroevolutionary time scale in 26 species of waterfowl. We show across multiple immune pathways (AIV-, toll-like-, and RIG-I -like receptors signalling pathways) that genes involved genes in pathogen detection (i.e., toll-like receptors) and direct pathogen inhibition (i.e., antimicrobial peptides and interferon-stimulated genes), as well as host proteins targeted by viral antagonist proteins (i.e., mitochondrial antiviral-signaling protein, [MAVS]) are more likely to be polymorphic, genetically divergent, and under positive selection than other innate immune genes. Our results demonstrate that selective forces vary across innate immune signaling signalling pathways in waterfowl, and we present candidate genes that may contribute to differences in susceptibility and resistance to infectious diseases in wild birds, and that may be manipulated by viruses. Our findings improve our understanding of the interplay between host genetics and pathogens, and offer the opportunity for new insights into pathogenesis and potential drug targets.

## Introduction

Animals share their environment with a wide array of pathogens, and their ability to fight infections is crucial for survival. Interestingly, even closely related species can differ in their susceptibility to particular infectious diseases. Finding the molecular basis of these differences is not an easy task, as a successful immune response requires coordination of many individual components of the complex immune system. Comparative immunogenetics and population genetics have played a pivotal role in investigating whether putative “susceptibility genes” are under selection in natural populations (Barreiro and Quintana-Murci 2010). However, such investigations are usually limited to a small number of immune genes or gene families, and encompassing studies looking at whole pathways within the immune system are few (but see Han et al. 2013; Darfour-Oduro et al. 2016; Tian et al. 2019). As a result, our knowledge of the selective processes across complex immune pathways is limited, and signatures of important host–pathogen interactions up- or downstream of the well-studied genes might have been missed.

The innate immune system is the first line of defense upon infection, and nonspecific in the sense that it rapidly recognizes general patterns of a wide range of pathogens (Akira et al. 2006). In innate immunity signaling pathways, cellular pattern recognition receptors (PRRs) such as toll-like receptors (TLRs) and RIG-I-like receptors (RLRs) detect conserved molecules on microbes (Murphy and Weaver 2016). Many PRRs activate downstream signaling pathways that culminate in the activation of transcription factors and the production of interferons (IFNs) (Bowie and Unterholzner 2008). The IFNs then initiate immune responses in infected and neighboring cells, which involves the expression of numerous IFN-stimulated genes (ISGs). Some of these ISGs (such as RIG-I) amplify and regulate the IFN response, whereas other ISGs (such as myxovirus resistance gene [Mx]) directly inhibit the life cycle of pathogens (Bowie and Unterholzner 2008).

Opposing views exist on the mode of evolution on the ancient innate immune system (Mukherjee et al. 2014): 1) new mutations are rapidly lost as natural selection has already optimized these genes, 2) coevolution with rapidly evolving pathogens creates and retains high genetic variation in them (Parham 2003). In reality, the evolutionary history of innate immune genes is likely to vary as their functions differ widely. And indeed, some studies have found that innate immune genes may experience different selection pressures based on their position in gene networks (Han et al. 2013; Darfour-Oduro et al. 2016) or even on different domains within the same gene, for example, the extracellular and intracellular domains in the TLRs (Alcaide and Edwards 2011). To add to the complexity, viruses can manipulate critical steps in innate immune signaling pathways via protein–protein interaction (reviewed in Bowie and Unterholzner 2008; Unterholzner and Almine 2019), which may alter the selection pressure on the targeted host proteins. Since most studies assess only a small number of genes from particular innate immune signaling pathways, we do not know much about the evolutionary history of the majority of the genes in innate signaling pathways. Studying genetic diversity and evolution of a wide range of innate immune genes simultaneously thus provides an opportunity to learn more about the interplay between pathogens and host immunity.

Waterfowl (family Anatidae; including ducks, geese, and swans) are a taxon of high interest for evolutionary genetics and comparative immunology. All waterfowl species live in aquatic habitats, which are ideal ecosystems for diverse pathogens, and allow for prolonged survival of viruses in particular (Hinshaw et al. 1979; Brown et al. 2007). Waterfowl commonly aggregate in high numbers with closely related species, which facilitates cross-species transmission of infectious diseases. Last but not least, waterfowl are one of the primary reservoirs of the avian influenza virus (AIV) (Stallknecht and Shane 1988; Webster et al. 1992), a zoonotic disease with a high impact on human health (WHO 2022). Field observations revealed that the occurrence of the AIV differs among waterfowl species (Olsen et al. 2006; Stallknecht and Shane 1988) and experimental studies showed that waterfowl differ in their susceptibility to AIV (Perkins and Swayne 2002; Brown et al. 2006). While ducks, and in particular the mallard *Anas platyrhynchos*, show little signs of infection by the AIV (Perkins and Swayne 2002; Brown et al. 2006), geese and swans seem to be more susceptible to highly pathogenic AIV (HPAIV) (Ellis et al. 2004; Brown et al. 2008). Waterfowl are thus an ideal system to study evolutionary patterns in the innate immune system.

In waterfowl, genetic diversity and selection of the innate immune system has mainly been characterized in avian β-defensin genes, which code for antimicrobial peptides that interfere with microbial membranes (Ganz 2003). While most β-defensins are primarily under purifying selection in waterfowl, evidence for balancing selection was found on a recently duplicated β-defensin gene in mallards (Chapman et al. 2016). In birds, evolutionary patterns have also been characterized in the TLR family. Similar to TLRs in mammals, avian TLRs are generally under purifying selection with low to moderate nucleotide diversity, but show signatures of positive directional selection in the extracellular leucine-rich repeat (LRR) domain involved in pathogen detection (Alcaide and Edwards 2011; Grueber et al. 2014; Velová et al. 2018; Yang et al. 2021). However, signatures of selection on other components of the avian innate immune system have been less well characterized.

In this study, we assessed genetic variation and divergence of the innate immune system in waterfowl, and conducted a comprehensive comparison of evolutionary patterns in innate immune genes across entire gene networks. Modern high throughput DNA technologies allowed us widen the focus beyond specific genes to consider many of the known components of the PRR signaling pathways. Using a hybrid capture approach, we sequenced innate immune genes in four populations of wild mallards from around the world as well as from farm mallards and Pekin ducks. This enabled us to study the genetic diversity and population genetics of a wide range of immune genes in the main host of AIV at a microevolutionary timescale. We hypothesized that genes involved in detection of pathogens may be more divergent than other immune genes, as they are likely coevolving with distinct pathogen communities at different locations. By sequencing the same genes in five further duck species, and including published genomic data from 20 goose species (Ottenburghs et al. 2016), we further assessed the forces of natural selection acting on the target genes at a macroevolutionary time scale in 26 species of waterfowl. We hypothesized that innate immune genes may show different evolutionary patterns depending on their function and pathway position. We provide the first comprehensive analysis of the population genetics and evolution of innate immunity signaling pathways in waterfowl.

## Results and Discussion

### Reference-Based Assembly and Retrieval of Immune Genes

To assess genetic variation and evolutionary patterns in the innate immune system of waterfowl, we first used customized molecular baits and hybrid capture DNA sequencing to genotype 127 innate immune genes in wild mallards (*A. platyrhynchos*) from four different populations. To investigate whether there may be an impact of domestication on the immune system in mallards, we further genotyped the same immune genes in mallards reared to be released into the wild to increase the size of hunted populations (hereafter called farm mallards) and Pekin ducks (*A. platyrhynchos domesticus*). We also genotyped a sample of individuals from five further duck species (*Anas crecca, Anas penelope, Anas americana, Aythya ferina, Aythya fuligula*). As waterfowl are important hosts of the AIV, which can be detected by different classes of PRRs, including TLRs and RLRs (Evseev and Magor 2019; Magor 2022), we included genes across the TLR, RIG-I, and Influenza A signaling pathways and additional genes (interferon-induced transmembrane protein 3 [IFITM3] and β-defensins) that have been studied in mallards previously (supplementary table S1, Supplementary Material online). The sequenced regions added up to approximately 1.77 Mbp, with individual genes ranging from 90 bp to 100 kbp in size including both introns and exons. The number of sequencing reads per individual ranged from 0.36 to 4.89 million for the wild and domesticated mallards, and from 0.96 to 1.84 million for the other duck species. On average, 95.33% and 91.27% of the sequencing reads from wild and domesticated mallards and from the other duck species successfully mapped to the mallard reference genome, respectively (supplementary table S2, Supplementary Material online). The average sequencing depth for the protein-coding sequence for each gene ranged from 2.24× to 265.90× in the wild and domesticated mallards and from 1.53× to 158.29× for the other duck species (supplementary table S3, Supplementary Material online). A total of 119 genes (four of which with two isoforms) were included in the intraspecies analyses after excluding genes based on a number of exclusion criteria (see Reference-Based Assembly and Retrieval of Immune Genes).

To recover immune genes from related duck species, we used the same set of baits as for the mallards. Even though the baits were designed using the mallard genome, the majority of the immune genes were successfully captured and sequenced in the other duck species as well (supplementary table S3, Supplementary Material online). We thereby provide a resource for comparative immunology for >100 innate immune genes from five duck species of which several lack a reference genome. Our hybrid capture approach opens up avenues for future comparative studies in closely related species for which the genomes are not yet available (cf. Förster et al. 2018). We expect that the capture process works sufficiently well for analyses as presented here in at least all species of the tribe Anatini and Aythyini based on our results from *Anas* spp. and *Aythya* spp. (Del Hoyo et al. 2014).

To examine evolutionary patterns in a wider range of waterfowl species, we further mined immune genes from published genomic data of 20 goose species (supplementary table S4, Supplementary Material online). We found 103 of the mallard immune genes (of which one has two isoforms) in the goose genomes, after excluding genes with premature stop codons in one or several goose species (supplementary table S5, Supplementary Material online). Considering that species of ducks and geese differ in their susceptibility to infectious diseases such as AIV (Capua and Mutinelli 2001; Perkins and Swayne 2002; Brown et al. 2008; Phuong et al. 2011), future studies of the excluded genes and their differences would be of great value.

### Genetic Variation, Population Divergence and Evidence of Natural Selection in Waterfowl

#### The Pekin Duck Flock Had Lower Median Nucleotide Diversity Than Wild and Farm Mallards

To measure the degree of polymorphism for the coding sequence of each gene in the mallards, we used nucleotide and amino acid diversity (Nei 1987). The average nucleotide diversity per site (*π*) across all genes was 0.005 ± 0.005 (mean ± SD) (supplementary table S6, Supplementary Material online) in wild mallards (*n* = 64), 0.004 ± 0.004 in farm mallards (*n* = 16, supplementary table S7, Supplementary Material online), and 0.003 ± 0.003 in Pekin ducks (*n* = 16, supplementary table S8, Supplementary Material online). The average amino acid diversity per site across all genes was 0.005 ± 0.008 (supplementary table S9, Supplementary Material online) in wild mallards (*n* = 64), 0.005 ± 0.008 in farm mallards (*n* = 16, supplementary table S10, Supplementary Material online), and 0.003 ± 0.006 in Pekin ducks (*n* = 16, supplementary table S10, Supplementary Material online). Some of the genes with the highest nucleotide and amino acid diversity in wild mallards were *chemokine (C–C motif) ligand 5* (*CCL5*), *lipopolysaccharide binding protein* (*LBP*), *mitochondrial antiviral-*signaling (*MAVS*) *protein,* and *avian* β*-defensin 8*, *9*, *10, 11, and 12* (*AvBD8, AvBD9, AvBD10, AvBD11,* and *AvBD12*) (supplementary table S9, Supplementary Material online). Our results thus confirm that several β-defensin genes display high polymorphism in waterfowl, as has been shown previously (Chapman et al. 2016).

We calculated and compared nucleotide and amino acid diversity across all immune genes (*n* = 123) and between geographically distinct populations of wild and domestic mallard populations (supplementary tables S10 and S11, Supplementary Material online). While no difference was detected between any of the wild populations or the farm mallards, the Pekin duck population had significantly lower median nucleotide diversity than all other populations (Kruskal–Wallis *χ*^2^ = 34.547, *P* = 1.852e−06, Pairwise Wilcoxon rank sum test, adjusted *P-*values <0.05, supplementary fig. S1*A*and note S1, Supplementary Material online). Similarly, the Pekin duck population had significantly lower median amino acid diversity than all other populations except the Greenland population (Kruskal–Wallis *χ*^2^ = 19.923, *P* = 0.001, Pairwise Wilcoxon rank sum test, adjusted *P-*values <0.05, supplementary fig. S1*B*and note S1, Supplementary Material online). The lower nucleotide and amino acid diversity in the domesticated ducks suggests that at least this Pekin duck population has lost some genetic diversity in the innate immune system during domestication. Future studies including more domesticated populations are needed to show whether the patterns detected in our study apply to domesticated ducks in general.

#### Genetic Divergence of Immune Genes in Mallards

To determine the degree of adaptive divergence between immune genes within a species (here in mallards), we estimated genetic distances between the mallard populations. The pairwise genetic distances (*F*_ST_, Hudson et al. 1992) between the wild mallard populations were all low, with a slightly higher *F*_ST_ value when comparing the mallards from the Greenland population with the other populations (supplementary table S12, Supplementary Material online). Similar patterns were found in previous studies carried out with mitochondrial DNA and single-nucleotide polymorphism (SNP) markers (Kulikova et al. 2005; Kraus et al. 2011a; Kraus et al. 2011b; Kraus et al. 2013; Kraus et al. 2016).

**Fig. 1.**
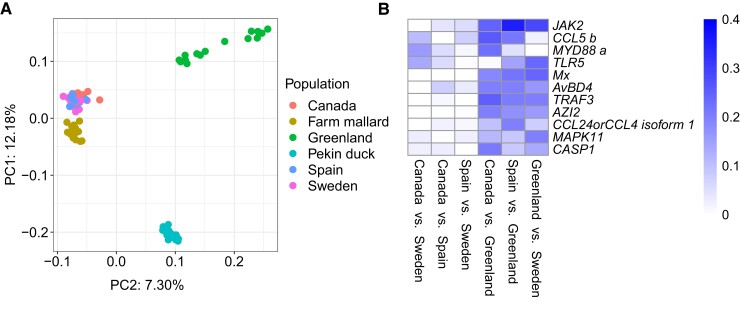
Genetic differentiation of immune genes between mallard populations. (*A*) Sample clustering using variance components estimated by the PCA based on SNPs from all wild and domesticated mallards included in this study. The PCA shows tight clustering of the Canadian, Spanish, and Swedish mallards, separating the Greenlandic mallards and Pekin duck flock from the other populations. Farm mallards cluster more closely with wild mallards than Pekin ducks. (*B*) Pairwise distance per gene (*F*_ST_) as estimated in DNASP. Here, all genes where *F*_ST_ > 0.20 in at least one pairwise comparison between the wild mallard populations are visualized (for an extended heatmap see supplementary fig. S3, Supplementary Material online). The heatmap is clustered for rows and columns.

We further evaluated the genetic distance of each wild mallard population to the farm mallards and the Pekin ducks (supplementary table S12, Supplementary Material online). The farm mallards had the lowest divergence to the Swedish, Spanish and Canadian mallard population and higher divergence to the Greenland population and the Pekin ducks; likely because they were raised in Sweden and may have ancestry in the Swedish wild mallard population. As expected, the Pekin ducks were most genetically differentiated from the wild mallards, and also showed a higher genetic distance to the Greenland population than to the remaining mallard populations. The genetic distances between populations are visualized using a principal component analysis (PCA) conducted on SNPs from the immune genes (fig. 1*A*).

To determine the genetic differentiation (*F*_ST_) per gene, we calculated and plotted the *F*_ST_ for each gene among all wild populations (supplementary table S9, Supplementary Material online), between wild mallards and farm mallards, and between wild mallards and Pekin ducks (supplementary fig. S2, Supplementary Material online).

In wild mallards, *Janus kinase 2* (*JAK2*) and *TNF receptor-associated factor 3* (*TRAF3*) had the highest *F_ST_* values. *JAK2* is downstream of IFN receptors, and *TRAF3* is recruited to *MAVS* signaling and other pathways leading to *NF-ĸB*, likely targets for pathogen subversion. The ISGs *Mx* and *IFITM3* were further among the genes with the highest *F_ST_* values. Duck *IFITM3* has antiviral activity against avian influenza, and low sequence conservation with chicken *IFITM3*, suggesting that it is also a common target for subversion (Blyth et al. 2016). The *F_ST_* values for several PRRs (*TLR5*, *TLR15*, *TLR2a*, *DDX58*/*RIG-I*, *IFIH1*/*MDA5*, *TLR2*, *TLR4*) were further above the average *F_ST_* value for all genes (supplementary table S9, Supplementary Material online). These results suggest that host proteins that detect—or interact with—pathogens are more likely to be divergent than other immune genes, which could be caused by adaptation to local pathogen communities.

When comparing wild and farm mallards, a large proportion of the genes with the highest *F*_ST_ values were β-defensins (supplementary fig. S2*A*, Supplementary Material online). *AvBD1* stood out in particular, having the highest *F*_ST_ value between wild and farm mallards, while having a low *F*_ST_ value in wild mallards as well as between wild mallards and Pekin ducks. β-Defensins show direct antimicrobial action against microorganisms, and variation in antimicrobial activity has been observed in different alleles of some mallard β-defensin genes (Helin et al. 2020). Further studies are required to investigate if the observed genetic divergence for some immune genes between farmed and wild mallards may be a result of selection from different pathogen communities in their environment and whether they have an impact on the survival of farmed mallards in the wild.

When looking at the *F*_ST_ values between wild mallards and Pekin ducks, some genes with low *F*_ST_ value in wild mallards had a relatively high *F*_ST_ value when comparing wild mallards and Pekin ducks (e.g., *PIK3R3*, *AKT1*, *CTSK*, *PML*, supplementary fig. S2*B*, Supplementary Material online). As domesticated ducks have been under artificial selection for traits affecting body weight and egg production for a long time (Cheng et al. 2003; Gu et al. 2020), it is difficult to know whether the high genetic divergence observed between wild mallards and Pekin ducks for particular immune genes is a result of differences in pathogen pressure in their environment or rather due to breeding for other traits and genetic linkage. As Pekin ducks are often used as a model species in studies of AIV, characterizing the immunological differences between wild mallards and Pekin ducks is of high importance. Future studies including a wider range of Peking duck flocks would be highly beneficial.

The ISG *Mx*, was among the genes with highest *F*_ST_ value in all population comparisons (supplementary table S9 and fig. S2, Supplementary Material online). Associations between *Mx* haplotype and influenza infection status have been found in some duck species (Dillon and Runstadler 2010). Interestingly, there is also evidence that at least some duck *Mx* alleles are unable to inhibit the multiplication of AIV in avian and murine cells (Bazzigher et al. 1993). Apart from *Mx*, little is otherwise known about associations between innate immune gene haplotype and infection status in mallards.

We also estimated the pairwise genetic distance between each mallard population for each gene (supplementary fig. S3, Supplementary Material online). In general, the pairwise *F*_ST_ value between the wild populations from Canada, Spain, and Sweden was low, while the pairwise *F*_ST_ values between the Greenland population and the other wild population was more pronounced. For 11 genes, the *F*_ST_ was >0.2, as visualized in fig. 1*B*. *JAK2*, *TRAF3*, and *Mx* were among the genes with the highest pairwise *F*_ST_ between the Greenland and the remaining wild mallard populations. These genes would be excellent targets for future association studies.

Finally, we estimated *F*_ST_ including only nonsynonymous SNPs (*F*_ST__NON-SYN_ hereafter) to see whether variation at the protein level contributes to genetic differentiation between populations. In general, the average *F*_ST__NON-SYN_ between population was slightly lower than the average *F*_ST_, with relative genetic distances between populations remaining unchanged (supplementary table S13, Supplementary Material online). While most genes with a high *F*_ST_ in wild mallards also had a high *F*_ST__NON-SYN_ (e.g., *TRAF3*), the gene with the highest *F*_ST_ in wild mallards (*JAK2*) had a relatively low *F*_ST__NON-SYN_, suggesting that *JAK2* is under purifying selection (supplementary table S9 and fig. S4, Supplementary Material online). Interestingly, several genes with low *F*_ST_ among wild mallards but high *F*_ST_ between wild mallards and Pekin ducks had low *F*_ST__NON-SYN_ between wild mallards and Pekin ducks (e.g., *AKT1*, *CTSK*, *PML*, supplementary figs. S5 and S6, Supplementary Material online). The high differentiation at the nucleotide level but not the protein level suggest that these genes are under purifying selection as well. The gene *RIG-I* (*DDX58*) further showed a similar pattern with high *F*_ST_ but low *F*_ST__NON-SYN_ between wild mallards and Pekin ducks. The genes with the highest *F*_ST_ and *F*_ST__NON-SYN_ between wild and farm mallards corresponded well (supplementary fig. S7, Supplementary Material online).

#### Evidence of Natural Selection in Mallards and Waterfowl

We looked for evidence of natural selection in the immune genes at a micro- and macroevolutionary time scale in mallards and 25 additional waterfowl species of which four were dabbling duck species, two diving duck species and 20 geese species. Note that some taxa (i.e., swans) within the waterfowl order are under-represented or missing in the dataset. To detect genes under natural selection, we used Tajima’s *D* statistics (Tajima 1989), the McDonald–Kreitman (MK) test (McDonald and Kreitman 1991), and estimated the ratio of nonsynonymous and synonymous changes (d*N*/d*S*). While scanning for signals of natural selection across whole genes will allow for detection of genes that are under strong selection, weak signatures of selection can be masked by different selection patterns in specific codons. This can be particularly true for immune genes such as the TLRs, that have an extracellular domain involved in recognition of pathogens and an intracellular domain involved in signaling (Werling et al. 2009). To identify codons that might be affected by host–pathogen coevolution, we therefore also estimated the strength of selection on individual codons using models implemented in BayeScan (Foll and Gaggiotti 2008), Datamonkey (Pond and Frost 2005), and PAML (Yang 1997). Finally, we determined whether episodic diversifying selection has occurred in genes on certain branches in the species tree for the 26 waterfowl species using branch-site models (Smith et al. 2015) implemented in Datamonkey. The results from the different site models in PAML (M1a/M2a and M7/M8) were similar (supplementary table S14, Supplementary Material online), and as such the results from the more conservative M1a/M2a comparison (supplementary table S15, Supplementary Material online) was used for further comparisons with the data from the HYPHY analyses. The set of sites identified with PAML (M1a/M2a) and HYPHY were also similar, with 116 out of the 140 and 136 sites identified by PAML (supplementary table S15, Supplementary Material online) or at least two of the models in HYPHY (supplementary table S16, Supplementary Material online), respectively, overlapping (supplementary table S17, Supplementary Material online).

##### Pathway Position Has an Influence on Natural Selection of Genes in Waterfowl

**Fig. 2.**
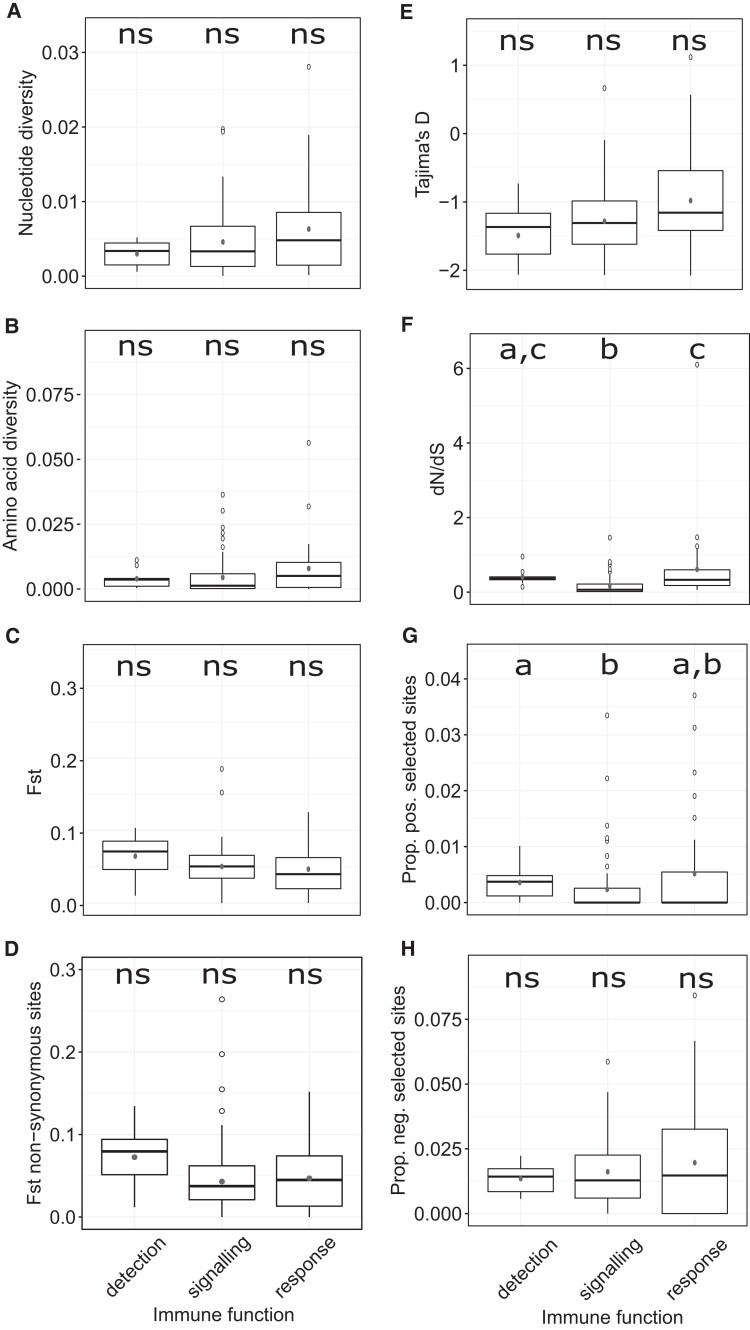
Boxplots showing (*A*) nucleotide diversity, (*B*) amino acid diversity, (*C*) average population divergence (*F_ST_*), (*D*) average population divergence of nonsynonymous sites only (*F_ST_*_NON-SYN_), (*E*) Tajima’s *D*, (*F*) d*N*/d*S*, (*G*) proportion of positively selected sites, and (*H*) proportion of negatively selected sites per gene for the functional groups. Significant differences were detected between groups when comparing d*N*/d*S* and the proportion of positively selected sites. The nucleotide diversity, amino acid diversity, *F*_ST_, and Tajima’s *D* were estimated using wild mallards only. d*N*/d*S* and the proportion of selected sites was estimated from a total of 26 species of waterfowl. d*N*/d*S* was estimated using PAML and the proportion of selected sites from HyPhy. The box shows the median and the 25% and 75% quantile. The lower whisker shows the smallest observation greater than or equal to lower hinge - 1.5×IQR, while the upper whisker shows the largest observation less than or equal to upper hinge + 1.5×IQR. The filled dots show the mean, and the open circles mark outliers. Medians with different letters were significantly different (P < 0.05, Kruskal–Wallis nonparametric ANOVA, Wilcoxon rank sum test, with FDR correction, Note S2). ns, nonsignificant, prop, proportion, pos., positive, neg., negative.

To investigate if innate immune genes show different evolutionary patterns depending on their pathway position, we estimated and compared the level of DNA polymorphism (*π*), amino acid diversity, DNA divergence (*F_ST_*), DNA divergence when including nonsynonymous changes only (*F_ST_*_NON-SYN_), and the type of selection pattern (Tajima’s *D*, d*N*/d*S*, proportion of selected sites) in genes belonging to three different functional groups; detection, signaling and response (fig. 2). No significant difference was detected in the median for all genes between the groups for nucleotide or amino acid diversity in wild mallards (fig. 2*A*and*B*), FST (fig. 2*C*), and FST NON-SYN (fig. 2*D*) among all wild mallard populations, Tajima’s *D* (fig. 2*E*), and the proportion of negatively selected sites (fig. 2*H*) (Kruskal–Wallis, *P* > 0.05, supplementary note S2, Supplementary Material online). In contrast, the d*N*/d*S* ratio was higher in genes with a function in detection and response than in genes with a function in signaling (fig. 2*F*, Kruskal–Wallis *χ*^2^ = 32.084, *P* = 1.079e−07, Wilcoxon rank sum test, adjusted *P*-values <0.05, supplementary note S2, Supplementary Material online). Similarly, the proportion of positively selected sites was higher in genes involved in detection than in genes involved in signaling (fig. 2*G*, Kruskal–Wallis *χ*^2^ = 8.079, *P* = 0.01761, Wilcoxon rank sum test, adjusted *P* = 0.0094).

**Fig. 3.**
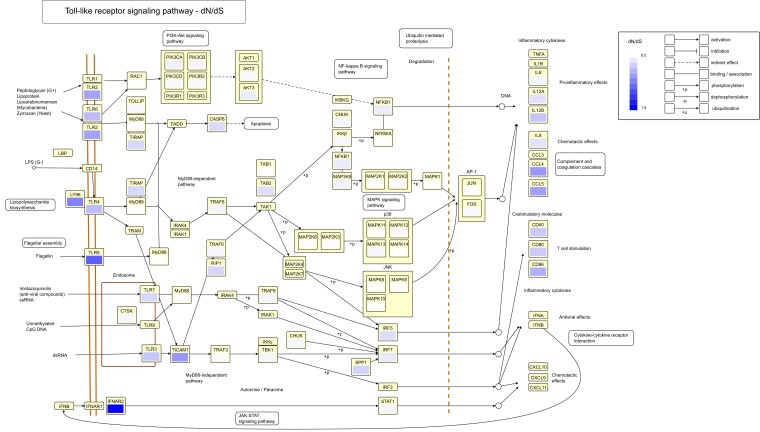
Ratio of nonsynonymous to synonymous changes (d*N*/d*S*) mapped on the Toll-like receptor signaling pathway from the KEGG database (Kanehisa and Goto 2000; Kanehisa 2019; Kanehisa et al. 2021). Each box represents one gene in the pathway and the color within the box shows d*N*/d*S* for that particular gene. d*N*/d*S* was estimated from a total of 26 species of waterfowl using PAML. Small boxes without a color indication were not included in the hybrid capture, usually because they were not annotated in the mallard genome at the start of the study.

To visualize the influence of pathway position on d*N*/d*S* in waterfowl, we mapped the d*N*/d*S* values on the TLR signaling pathway from the KEGG database (fig. 3). Our results are consistent with previous studies showing that nonsynonymous substitution levels differ along the TLR pathway. However, in contrast to our findings, earlier studies concluded that downstream genes had lower nonsynonymous substitution rates than upstream genes (Song et al. 2012; Han et al. 2013; Darfour-Oduro et al. 2016). This discrepancy could be due to different gene sets being included in the analysis. For example, we included some β-defensins and ISGs in our study, which were some of the genes with highest d*N*/d*S* in waterfowl (supplementary table S9, Supplementary Material online). Still, several inflammatory cytokines and co-stimulatory proteins in the TLR signaling pathway had higher d*N*/d*S* values than most signaling molecules in waterfowl (fig. 3).

The fact that nonsynonymous changes and the proportion of positively selected sites were higher in detector molecules than in signaling molecules in waterfowl is likely a result of positive selection in regions that recognize pathogens, as has been shown in avian TLRs previously (Downing et al. 2010; Grueber et al. 2014; Khan et al. 2019). In line with this hypothesis, many TLRs (*TLR1A*, *2*, *2a*, *4*, *5*, *7*, *21*, and *15*) had a high number of positively selected sites in waterfowl when compared with all other tested genes (supplementary tables S15 and S16, Supplementary Material online).

##### Signatures of Selection Were Detected on Host Proteins Known To Be Targeted By Viral Antagonist Proteins

Pathogens have developed strategies to evade and subvert the immune response. Many viruses, for instance, encode antagonist proteins that inhibit critical steps in innate immune signaling pathways via protein–protein interaction (reviewed in Bowie and Unterholzner 2008; Unterholzner and Almine 2019). Interestingly, several of the genes with high nucleotide diversity, amino acid diversity, high *F*_ST_ values and high proportion of positively selected sites in waterfowl are known to be targeted by viral antagonist proteins.

**Fig. 4.**
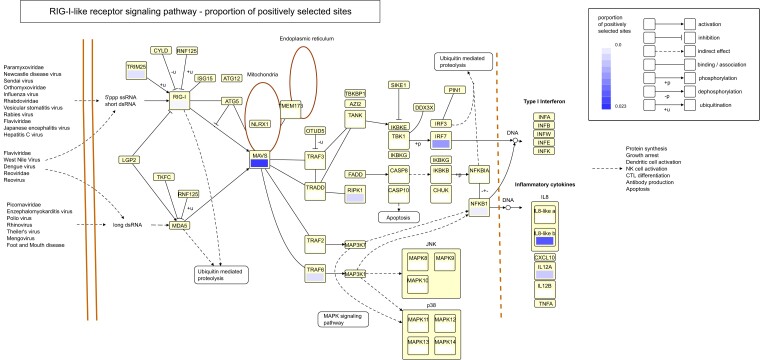
Proportion of positively selected sites mapped on the RIG-I-like receptor signaling pathway from the KEGG database (Kanehisa and Goto 2000; Kanehisa 2019; Kanehisa et al. 2021). Each box represents one gene in the pathway and the color within the box shows the proportion of positively selected sites among all sites within the CDS for that particular gene. White indicates a value of 0, and darker shades of blue denote higher proportions. The proportion of selected sites was estimated from a total of 26 species of waterfowl using HyPhy (Pond and Muse 2005). Small boxes without a color indication were not included in the hybrid capture, usually because they were not yet annotated in the mallard genome at the beginning of this study.

To visualize the selection on different components of the pathway, we mapped the proportion of positively selected sites on genes from the RIG-I like receptor signaling pathway (fig. 4). Again, we observe that the majority of the genes with positively selected sites (e.g., *MAVS*, *IL8, IRF7, TRAF6, TRIM25, RIG-I*) are those that are targeted by viral antagonist proteins (reviewed in Bowie and Unterholzner 2008), and some of these specifically by AIV nonstructural proteins. For example, the AIV nonstructural protein 1 (NS1) can block TRIM25-mediated RIG-I CARD ubiquitination (Gack et al. 2009; Koliopoulos et al. 2018) as well as type I IFN signaling downstream of RIG-I by inhibiting the activation of transcription factors such as *IRF3* (Mibayashi et al. 2007; Opitz et al. 2007). Furthermore, the AIV nonstructural protein PB1-F2 inhibits IFN production in human and avian cells by interacting with the MAVS protein (Varga and Palese 2011; Xiao et al. 2020). Global approaches like ours may thus be suitable for detecting host proteins targeted by pathogens to evade the host immune response.

##### Signatures of Positive Selection on Branches Provide Candidate Genes for Understanding Species-Specific Differences in Susceptibility To Infectious Diseases

As codon-based site models usually only detect positive selection when sites are under selection in numerous lineages, we further determined if episodic diversifying selection has occurred among genes of certain lineages in the species tree for the 26 waterfowl species (four dabbling and two diving duck species and 20 geese species). Briefly, we tested for each branch in the phylogeny whether a proportion of sites in each gene have evolved under positive selection, using the adaptive branch-site random effects likelihood (aBSREL) algorithm (Smith et al. 2015) implemented in Datamonkey.

**Fig. 5.**
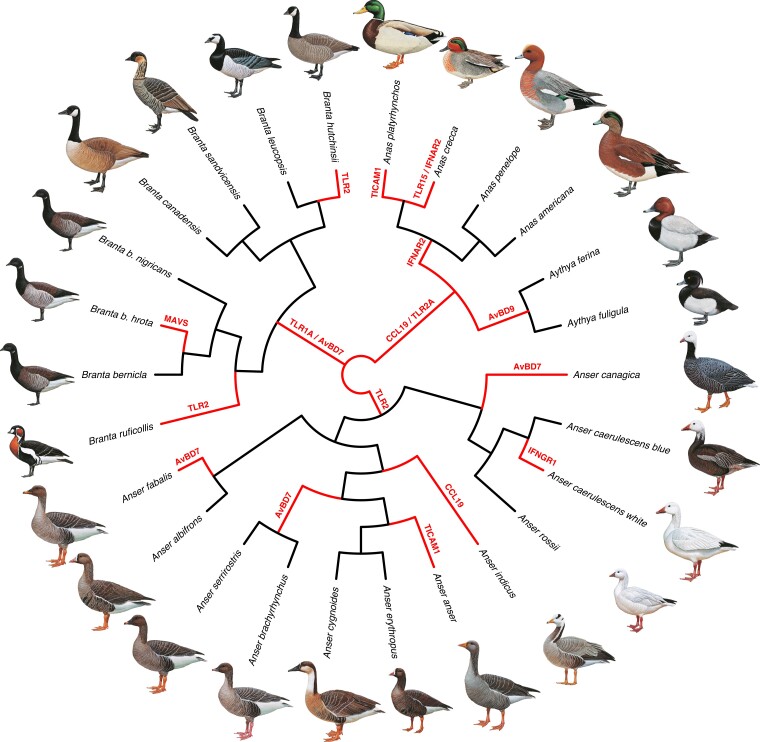
Evidence of positive selection in one or several immune genes across the waterfowl phylogeny. Evidence of positive selection was estimated using aBSREL models (Smith et al. 2015) using HyPhy (Pond and Muse 2005). Branches with adjusted P-values <0.05 for any of the tested immune genes are shown in red with the gene(s) under selection indicated on the branch. The displayed phylogenetic tree is the summary of 10,000 trees downloaded from http://birdtree.org (Jetz et al. 2012). Drawings used with permission of the Handbook of Birds of the World (Del Hoyo et al. 2006).

Signs of positive selection were detected in one or several branches for 11 genes (*AvBD7*, *AvBD9*, *CCL19*, *IFNAR2*, *IFNGR1*, *MAVS*, *TICAM1*, *TLR1A*, *TLR2*, *TLR2A*, *TLR15*) out of the 105 tested immune genes, as visualized in fig. 5. The gene *AvBD7* is under positive selection in all *Branta spp*. and several *Anser spp. TLR2* and *TLR2a* are further under selection in all *Anser* spp. as well as some *Branta* spp. and in all ducks respectively. *AvBD7* is one of the avian β-defensins that have duplicated and/or lost their function through pseudogenization in some avian lineages, and was the β-defensin with the highest number of branches subject to episodic diversifying selection in a study of the evolution of antimicrobial peptides in 53 avian species from different orders (Cheng et al. 2015). Likewise, two of the *TLRs* that showed signs of episodic diversifying selection on several branches in our study (*TLR1* and *TLR2*) have gone through a duplication event in the avian lineage (Cormican et al. 2009; Alcaide and Edwards 2011). When compared with other avian *TLRs*, *TLR2A* had a higher degree of positive selection on terminal branches than internal branches (including in the *Anatidae* lineage), which (Grueber et al. 2014) suggested might indicate that *TLR2A* has a higher degree of species-specific selection than other avian *TLRs*. Our result further supports previous research showing that *TICAM1*, also known as *TRIF*, is under strong species-specific selection in avian lineages (Shultz and Sackton 2019). TICAM1 is the adaptor protein through which the viral sensing TLR3 initiates downstream signaling in birds (Santhakumar et al. 2017). Interestingly, several of the detected genes are involved in IFN response. Mallards (and potentially other waterfowl) limit AIV spread and viremia early through a rapid RIG-I receptor-mediated type I IFN signal at the site(s) of infection (Evseev and Magor 2019). The large variation of different influenza strains circulating in mallard populations (Latorre-Margalef et al. 2014) may thus exert strong positive selection on genes of the RIG-I gene cascade. The genes detected by the branch-site model are good candidates for future studies assessing species-specific differences in susceptibility to infectious diseases.

##### The ISG Mx and Avian-Specific TLR15 Are Under Positive Selection in Mallards

The genes *TLR15* and *Mx*, deviated from neutrality in several of our selection analyses. *TLR15* is an avian and reptilian specific TLR with no apparent ortholog in mammals (Alcaide and Edwards 2011; Brownlie and Allan 2011; Voogdt et al. 2018), and is upregulated during bacterial, viral and yeast infections (Higgs et al. 2006; Boyd et al. 2012; Jie et al. 2013). *TLR15* was one of three genes under adaptive evolution in wild mallards according to the MK test (supplementary tables S18 and S19, Supplementary Material online). The majority of the positions with fixed differences between the mallard and the tufted duck were located in the LRR domain (supplementary table S20, Supplementary Material online). *TLR15* was also the only gene with a SNP under diversifying selection leading to a nonsynonymous change on the protein level in wild mallards according to the BayeScan analysis (supplementary table S21, Supplementary Material online). Again, the SNP under selection was located in the LRR ectodomain (supplementary table S21, Supplementary Material online) in TLR15 (see predicted 3D protein structure in supplementary fig. S8, Supplementary Material online). Despite being located in the most variable LRRs of TLR15 (LRR6), it has so far not been found to be under natural selection in birds (Alcaide and Edwards 2011; Grueber et al. 2014; Wang et al. 2016; Velová et al. 2018). At this position all mallards from Greenland had a thymine (G**T**C = valine), whereas the mallards from Sweden, Spain, and Canada had a mix of thymines (G**T**C = valine) and cytosines (G**C**C = alanine). In birds, a high number of positively selected sites have previously been found in the LRR domains of *TLR15* (Wang et al. 2016; Khan et al. 2019). However, a study in chicken has shown that activation of TLR15 involves proteolytic cleavage of the LRR ectodomain (de Zoete et al. 2011), suggesting that genetic variation in this domain could be functionally neutral. In addition, *TLR15* has been revealed to cryptically pseudogenize in some birds (Fiddaman et al. 2021) which could partially explain the high sequence variation detected in this gene. We did, however, not detect any signs of pseudogenization of *TLR15* in the mallard genome, and a test for relaxation of selection pressure (implemented in Datamonkey) on *TLR15* in the mallard versus all other investigated taxa was not significant (*K* = 0.62, *P* = 0.67, LR = 0.18).

Mx codes for IFN-induced GTPase proteins that interfere with viral replication (Haller et al. 2007). *Mx* is upregulated in ducks and geese during viral infection (Chen et al. 2017; Helin et al. 2018; Jax et al. 2021). Like *TLR15*, *Mx* was one of three genes under adaptive evolution in wild mallards according to the MK test (supplementary tables S18 and S19, Supplementary Material online). It further contained the only SNP under diversifying selection that led to a nonsynonymous change on the protein level when including both wild and domesticated mallards in the BayeScan analysis (supplementary table S22, Supplementary Material online). This nonsynonymous SNP is located in the dynamin central domain (supplementary table S20, Supplementary Material online) of the *Mx* gene (see predicted 3D protein structure in supplementary fig. S9, Supplementary Material online). In our study, all wild mallards had an adenine (A, **A**TT = isoleucine) at this amino acid position while some farm mallards (*n* = 10) and Pekin ducks (*n* = 2) had a guanine (G, **G**TT = valine). To our knowledge this position has not been reported to be under positive diversifying selection in birds previously (Berlin et al. 2008; Zeng et al. 2016). However, the overall high polymorphism and the evolutionary pattern observed in *Mx* in our study is comparable with the results of previous research in ducks (Chen et al. 2017; Helin et al. 2018). Functional assays on the effect of the genetic variants in ducks and geese would be of high value to understand the role of *Mx* and *TLR15* polymorphisms in susceptibility and resistance to infections.

## Conclusion

To conclude, we show that pathway position has an influence on the evolutionary history of innate immune genes in waterfowl. More specifically, up- and downstream host proteins that detect- or interact with pathogens were more likely to be under selection than other innate immune genes. Interestingly, we also found that several proteins known to be targeted by viral antagonist proteins had high DNA polymorphism, divergence, and signatures of selection in waterfowl. Our results give new insights into the interplay between host genetics and pathogens, and provide candidate genes that may inform new approaches for treating and preventing zoonotic diseases.

## Materials and Methods

### Sampling

We included samples from 64 wild mallards (*A. platyrhynchos*) from four populations (Sweden *n* = 16, Spain *n* = 16, Canada *n* = 16, and Greenland *n* = 16) and from a total of 16 individuals from five species of wild ducks (*A. crecca n* = 4, *A. penelope n* = 3, *A. americana n* = 3, *Ay. ferina n* = 3, *Ay. fuligula n* = 3). Sampling, DNA isolation as well as identification and removal of closely related individuals from the wild ducks have been described previously (Kraus et al. 2012; Kraus et al. 2013). To investigate whether domesticated mallards have a similar genetic diversity in immune genes as wild mallards, we also included samples from 16 farmed mallards from a single farm in Sweden raised to be released into the wild to increase the harvestable population (Söderquist 2015) and 16 Pekin ducks (*A. platyrhynchos domesticus*) from a single agricultural breeding facility. Michele Wille at Uppsala University, Sweden, kindly provided us with red blood cells from farm mallards, and a breeder in Southern Germany provided whole blood from Pekin ducks. We extracted DNA using a DNeasy Blood & Tissue Kit (Qiagen GmbH, Hilden Germany), and further purified and concentrated samples with a concentration of <50 ng/µl with DNA Clean & Concentrator^™^-5 (Zymo Research, Freiburg Germany). To allow for interspecies analyses, we further included genomic data from a study on the phylogeny of all goose species (ENA accession number PRJEB20373; Ottenburghs et al. 2016; Ottenburghs et al. 2017).

### Bait Design

Customized molecular baits to capture targets from a pool of isolated DNA were designed by MYcroarray (ArborBiosciences, MI, USA) for a total of 127 immune genes (supplementary table S1, Supplementary Material online). We chose immune genes from the TLR signaling pathway (apla04620), the Influenza A pathway (apla05164), and the RIG-I-like receptor signaling pathway (apla04622) for mallard in the Kyoto Encyclopedia of Genes and Genomes (KEGG) database (Kanehisa and Goto 2000; Kanehisa et al. 2010). We further included *IFITM3* and all known β-defensins as some of these genes or gene regions have been studied previously in ducks (Blyth et al. 2016; Chapman et al. 2016). We designed the baits for whole genes including 500 bp down- and upstream of the CDS (target sequences downloaded from BioMart, Ensembl release 91, Kinsella et al. 2011). The targeted region added up to approximately 1.77 Mbp, with individual genes ranging from 90 bp to 100 kbp in size including introns and exons.

### Library Preparation and Enrichment

We prepared libraries for all duck samples using a NEBNext Ultra II DNA Library Prep Kit for Illumina and NEBNext Multiplex Oligos for Illumina (Dual Index Primers Set 1, New England Biolabs, Frankfurt am Main, Germany) and Agencourt AMPure XP Beads 60mL (Beckman Coulter, Krefeld, Germany). We produced libraries according to the manufacturer's protocol, and pooled them in groups of five before doing the enrichment step. We enriched each pool using the MYcroarray MYBaits kit version 3 and the set of custom-designed probes targeting 127 immune genes (supplementary table S1, Supplementary Material online) following the manufacturer’s instructions. We ran the hybridization reaction with the NEBNext Ultra II Q5 Master Mix (New England Biolabs, Frankfurt am Main, Germany) for 24 h at 65°C, subsequently bound all pools to Dynabeads MyOne Streptavidin C1 magnetic beads (Invitrogen, Karlsruhe, Germany). We finally washed the bound libraries according to a standard target capture protocol (Blumenstiel et al. 2010). We assessed the concentration and quality of the libraries on a Qubit v.2.0 fluorometer (Life Technologies, Darmstadt, Germany) and a 2100 Bioanalyzer (Agilent Technologies, Waldbronn, Germany), respectively, before and after capture. The mallard samples were sequenced to 2×250 bp paired-end on an Illumina HiSeq2500 platform, and the samples from the other duck species to 2×250 paired-end on an Illumina MiSeq at Tufts University Core Facility (TUCF Genomics, MA, USA).

### Reference-Based Assembly and Retrieval of Immune Genes

We checked the quality of the sequencing reads using FASTQC v0.11.4 (Andrews 2010), and trimmed low-quality bases and removed remaining adapters using Trimmomatic v.0.32 (Bolger et al. 2014) with the following settings; LEADING:10 HEADCROP:5 TRAILING:10 SLIDINGWINDOW:4:20 MINLEN:70. We aligned the filtered reads to the mallard reference genome (BGI_duck_1.0, GenBank assembly accession: GCA_000355885.1; Huang et al. 2013) using Bowtie2 v.2.2.3 (Langmead and Salzberg 2012) for the mallards, and SMALT v.0.7.6 (https://www.sanger.ac.uk/tool/smalt-0/) for the other ducks species. SMALT has been shown to be appropriate for mapping paired-end reads to distantly related reference genomes (Frantz et al. 2013). We used SAMtools v.1.3.1 (Li et al. 2009) with default settings to retrieve alignment statistics, to process the alignment files, and to make a consensus fastq file for each individual. We converted the resulting fastq files to fasta files using a customized python script and made multi-fasta files containing all genes of interests for each individual using BEDtools v.2.26.0 (Quinlan and Hall 2010). BED files with coordinates of the genomic regions (supplementary table S23, Supplementary Material online) were used for calculating coverage, depth and getting the gene-specific multi-fasta files. We estimated the sequencing depth of the protein-coding regions using SAMtools, and excluded genes that had an average sequencing depth of <10× across the protein-coding regions from all analyses (*DHX58, IRF7*, *MAP2K7*, *NLRX1*, *SOCS3*, *TLR21*). For each gene, we aligned the CDS of all samples with ClustalW v.2.0.12 with default setting (Thompson et al. 1994) using sequences downloaded from BioMart, Ensembl release 91 as reference, and manually curated them in MEGA7 (Kumar et al. 2016). For those genes where two isoforms are reported in the genome, we included both isoforms in the analyses. For cases where certain parts of the genes were missing, we replaced the missing nucleotides with Ns. We then excluded individuals with >25% nucleotide sequence missing in the protein-coding region of a gene from the intraspecies analyses for that particular gene (supplementary tables S24–S27, Supplementary Material online). We also excluded genes if they did not have data for 50% of the individuals (*JUN*, *FOS*, *DHX58*). Finally, we excluded *AvBD3-3* and *AvBD3-2* as they were not detected in the majority of the individuals. We reconstructed haplotypes from diploid genotypes to allow for analyses of genetic variation in the mallards using PHASE v.2.1.1 (Stephens et al. 2001) with default settings and the options -d1 -MR. We used the command-line version of Seqphase (Flot 2010) to convert the fasta sequence alignments to PHASE input and from PHASE output formats, and a customized R script (R Core Team 2014) to retain the haplotypes with the highest probabilities for each individual.

To allow for interspecies analyses, we included the diploid gene sequences from one randomly selected individual of each duck species (*A. platyrhynchos*, *A. crecca, A. penelope, A. americana*, *Ay. ferina*, *Ay. fuligula*) from this study. We further included genomic data for all species of geese (*n* = 20, supplementary table S4, Supplementary Material online) from a study by Ottenburghs et al. (2016). We aligned the goose genomic data to the same mallard reference genome using SMALT and extracted genes of interest using the same approach as described above. We excluded 24 genes due to premature stop codons in one or several species (supplementary table S5, Supplementary Material online), as the species with stop codons are unlikely to express the same functional isoforms as the mallard for these particular genes. We prepared multiple CDS alignment files for each gene for all species (*n* = 26) in MEGA7 (Kumar et al. 2016). We then constructed a species tree for all 26 duck and goose species to allow for selection analyses in a phylogenetic framework. We obtained a total of 10,000 phylogenetic trees for the investigated species from http://www.birdtree.org (Jetz et al. 2012) using the (Hackett et al. 2008) full tree backbone. We merged the trees in MEGA7 to obtain one consensus tree for further analyses. Out of the 26 species used in this study, one species (*Anser serrirostris*) and three subspecies (*Branta bernicla bernicla*, *B. bernicla hrota*, *B. bernicla nigricans*) were missing in the birdtree database. We therefore added these species manually according to the most recent phylogenetic tree for geese (Ottenburghs et al. 2016). We unrooted the final tree (supplementary fig. S10, Supplementary Material online) using the Analyses of Phylogenetics and Evolution (APE) v5.4-1 package (Paradis et al. 2004; Popescu et al. 2012) in R. For some of the genes or isoforms, a premature stop codon appeared in one of the exons in several of the bird species. As these species likely do not have the same functional isoform as the mallard for these particular genes, we excluded these genes from the interspecies selection analyses. The entire protein-coding sequence was used for all analyses (including regions that have undergone gene conversion in TLR1 and TLR2 in birds) except when specified.

### Genetic Variation, Population Divergence and Evidence of Natural Selection in Waterfowl

#### Genetic Variation in Mallards

We calculated nucleotide diversity (pi [π], the average number of nucleotide differences per site between two sequences, Nei 1987) in the wild mallards (*n* = 64), farm mallards (*n* = 16), and Pekin ducks (*n* = 16) separately using the phased coding sequence for each gene (*n* = 123) with DnaSP v.6 (Rozas et al. 2017). We further estimated the levels and patterns of nucleotide variation for each population in DnaSP. Using the translated version of the same sequences, we additionally calculated the average number of amino acid differences per site between two sequences in MEGA X (Kumar et al. 2018) for the same groups. We used a Kruskal–Wallis rank sum test (Hollander et al. 2013) and a pairwise Wilcoxon test with false discovery rate (FDR) correction (Benjamini and Hochberg 1995) to test whether the nucleotide and amino acid diversity differed between mallard populations, using the R Stats package v.3.4.2. We used an FDR-adjusted *P*-value <0.05 as the criterion for statistical significance for all comparisons.

#### Genetic Differentiation between Mallard Populations

We estimated the amount of DNA divergence between the populations (*F*_ST_) from the phased coding sequences for all genes (*n* = 123) combined, as well as for each immune gene in DnaSP. We generated a heatmap visualizing genes with pairwise *F*_ST_ values higher than 0.20 between at least two populations using the pheatmap v.1.0.12 package in R (Kolde and Kolde 2015). We also determined the average *F*_ST_ value for all populations for each gene in DnaSP. We visualized the genetic distances between populations using a PCA using the R package SNPRelate (Zheng et al. 2012). To investigate the contribution of the protein level to the genetic differentiation between populations, we also performed the *F*_ST_ analysis including only nucleotide sites that lead to a nonsynonymous change on the protein level. The sequences were generated from the manually curated protein-coding sequences in DnaSP.

#### Evidence of Natural Selection in Mallards and Waterfowl

We used several intra- and interspecies approaches to test for evidence of natural selection for each immune gene in mallards and waterfowl. In the first approach we used Tajima’s *D* statistics, which tests if a DNA sequence has evolved neutrally by comparing the number of segregating sites with the pairwise differences between individuals from one species (Tajima 1989). We calculated Tajima’s *D* values for the phased protein-coding sequences for each gene (*n* = 123) in wild mallards (*n* = 64) using DnaSP v.6.

In the second approach, we used a MK test, which detects genes that deviate from natural selection by comparing the polymorphism in one species with the divergence to another species (McDonald and Kreitman 1991). For this test, we used the phased protein-coding sequence in wild mallards (*n* = 64) and from one of the diving duck species, the tufted duck *Ay. fuligula* (*n* = 3). We chose the tufted duck for this analysis as it is more susceptible to HPAIV than mallards (Keawcharoen et al. 2008) and has been overrepresented among identified positive cases during outbreaks of HPAIV in wild birds (Bragstad et al. 2007). We excluded genes where one or several of the tufted duck individuals had >25% nucleotide sequence missing in the protein-coding region of gene from the analysis. We ran the test for each gene (*n* = 112) using DnaSP. We adjusted the *P*-values for multiple comparisons using the FDR method (Benjamini and Hochberg 1995), using the R Stats package. We predicted domains for the genes that deviated from neutrality using Interpro v69.0 (Finn et al. 2016), to identify the location of the specific differences within the protein-coding region. To investigate whether the selection pattern was similar in each mallard population when compared with the tufted duck, we additionally ran the MK test separately for each mallard population for the genes that were under selection in the whole dataset.

In a third approach, we assessed d*N*/d*S* for 105 genes in ducks and geese using maximum likelihood methods in a phylogenetic framework. We performed the analysis using CODEML in the PAML v.3.14 software package (Yang 2007). We report the estimated d*N*/d*S* (ω) from model 0, which assumes a constant d*N*/d*S* ratio over the whole protein-coding region (Yang 2007), as the d*N*/d*S* for each gene.

To investigate whether certain codons were under natural selection in immune genes in ducks and geese, we also estimated the strength of selection on individual codons.

First, we investigated whether single nucleotides may be under natural selection in mallard populations using the *F*_ST_-outlier approach implemented in BayeScan v2.1 (Foll and Gaggiotti 2008). For this purpose, we called SNPs in all immune genes (*n* = 127 as specified in supplementary table S1, Supplementary Material online, including introns and exons) using the variant detector freebayes v.9.9.2 (Garrison and Marth, unpublished data). We filtered the VCF file using VCFtools v.0.1.13 (Danecek et al. 2011) as specified in the *dDocent_filters* script (http://ddocent.com/filtering/) with some exceptions (supplementary table S28, Supplementary Material online). We generated one VCF file for wild mallards, and a separate VCF file for wild and domesticated mallards combined. We then converted both VCF files to BayeScan format using PGDSpider v.2.1.1.5 (Lischer and Excoffier 2012) and ran them in BayeScan. We considered SNPs with a *q*-value <0.1 significant and report the *F*_ST_ values for these SNPs. For nonsynonymous SNPs under diversifying selection (in *TLR15* and *Mx*), we modeled 3D topologies of proteins containing the corresponding amino acid changes using the I-TASSER server (https://zhanglab.ccmb.med.umich.edu/I-TASSER/; Roy et al. 2010). We visualized protein domains (from Fulton et al. 2014; Wang et al. 2016) and amino acid changes in the corresponding 3D models with the highest confidence score using PyMol Molecular Graphics System v.2.5.0 (Schrödinger LLC 2010).

Second, we performed a series of interspecies selection analyses for each target gene using HYPHY v.2.3.13 software (Pond and Muse 2005) implemented in the Datamonkey webserver (http://www.datamonkey.org/; Pond and Frost 2005). We detected signals for negative selection for each codon using FEL v.2.00 (Fixed Effect Likelihood; Kosakovsky Pond and Frost 2005) and SLAC (Single Likelihood Ancestral Counting; Kosakovsky Pond and Frost 2005). We detected signals for positive selection for each codon using SLAC, FEL, FUBAR v.2.1 (Fast, Unconstrained Bayesian AppRoximation; Murrell et al. 2013), and MEME v.2.0.1 (Mixed Effects Model of Evolution; Murrell et al. 2012). We used default values for each model to set the level of statistical significance (*P* < 0.1 for SLAC, FEL, and MEME, and posterior probability > 0.9 for FUBAR). These significance cut-offs are typically used for these analyses to avoid overestimation of positive selection while having a useful threshold for explorative studies (Cheng et al. 2015; Wang et al. 2016). To avoid reporting false positive results, we only considered codons with significant selection signals from two or more methods to be under selection. For comparison, we also investigated sites under positive selection using random site models in PAML. Briefly, we compared the null model, in which sites are under neutral evolution or purifying selection with alternative models that allow for positive selection. We tested for the presence of positively selected sites (M2/M1 and M8/M7) that were identified with Bayes’ Emperical Bayes. *P*-values were computed using the *χ*^2^ statistics for the ΔLRT (Likelihood Ratio Test) of two models. We applied FDR to adjust for multiple testing, and report sites with a posterior probability higher than 95%. To investigate whether TLR15 is under relaxed evolutionary pressure in waterfowl, we used the RELAX model (Wertheim et al. 2014) in the HYPHY v.2.3.13 software (Pond and Muse 2005).

Third, we used the aBSREL algorithm v2.0 (Smith et al. 2015) using the HYPHY v.2.3.13 software (Pond and Muse 2005) implemented in the Datamonkey webserver (http://www.datamonkey.org/; Pond and Frost 2005) to determine whether episodic diversifying selection has occurred on a proportion of sites in specific lineages in the species tree for the 26 waterfowl species. We corrected the *P*-values at each branch for multiple testing using the Holm–Bonferroni correction, and considered adjusted P-values <0.05 significant.

### Immune Pathway Function

We classified the immune genes into three functional groups (detection, signaling, and response) to allow for comparison of DNA polymorphism and evolutionary patterns in immune genes with different functions (supplementary table S29, Supplementary Material online). We classified genes involved in the detection of pathogens as detector molecules (*n* = 11); these include surface and cytoplasmic PRRs. We considered effector molecules that either directly inhibit the growth and fitness of pathogens, or that contribute to the upregulation of the defenses in nearby cells response molecules (e.g., IFN-induced transmembrane proteins, ISGs, antimicrobial peptides, cytokines, IFNs, *n* = 33). We considered the remaining genes in the pathways to be signaling molecules (*n* = 79).

We used a Kruskal–Wallis tests (Hollander et al. 2013) and pairwise Wilcoxon rank sum tests with Bonferroni–Holm adjustment (Benjamini and Hochberg 1995) to compare the nucleotide diversity, amino acid diversity, average *F*_ST_, Tajima’s D, d*N*/d*S*, and the proportion of positively and negatively selected sites (as identified through the HYPHY analyses) between the functional groups using the R Stats package v.3.4.2 (R Core Team 2014). We used a probability level of FDR <0.05 as the criterion for statistical significance for all comparisons between groups.

## Supplementary Material

msac160_Supplementary_Data

## Data Availability

Raw Illumina sequences are deposited in NCBI’s Sequence Read Archive (SRA) database with accession number PRJNA814885. CDS alignment files of all included genes are available on Figtree under DOI 10.6084/m9.figshare.20161283 and 10.6084/m9.figshare.20161286. Tissue and DNA samples are available upon request.
