# Supplementary material for: Comparative Genomics of the Waterfowl Innate Immune System
Source: Mol Biol Evol. 2022 Jul 26;39(8):msac160. doi: 10.1093/molbev/msac160 (PMC9356732; doi:10.1093/molbev/msac160)
Supplement: msac160_Supplementary_Data [file msac160_supplementary_data.docx]

Research Article

Discoveries

Supplementary files –

Comparative genomics of the waterfowl innate immune system

Elinor Jax^a,b,c^, Paolo Franchini^b,d^, Vaishnovi Sekar^e,f^, Jente Ottenburghs^g,h^, Daniel Monné Parera^b^, Roman T. Kellenberger^i^, Katharine E. Magor^j^, Inge Müller^a,b^, Martin Wikelski^a,k^, Robert H.S. Kraus^a,b^

^a^Department of Migration, Max Planck Institute of Animal Behavior, Radolfzell, Germany

^b^Department of Biology, University of Konstanz, Konstanz, Germany

^c^Department of Veterinary Medicine, University of Cambridge, Cambridge, UK

^d^Department of Biology and Biotechnologies “Charles Darwin”, Sapienza University, Rome, Italy

^e^Department of Biology, Lund University, Lund, Sweden

^f^Department of Molecular Biosciences, The Wenner-Gren Institute, Stockholm University, Sweden

^g^Wildlife Ecology and Conservation Group, Wageningen University, Wageningen, The Netherlands

^h^Forest Ecology and Forest Management Group, Wageningen University, Wageningen, The Netherlands

^i^Department of Plant Sciences, University of Cambridge, Cambridge, United Kingdom

^j^Department of Biological Sciences and Li Ka Shing Institute of Virology, University of Alberta, Edmonton, Canada

^k^Centre for the Advanced Study of Collective Behaviour, University of Konstanz, Konstanz, Germany

Corresponding author: Elinor Jax, ejax@ab.mpg.de

# Supplementary files

## Supplementary figures

**
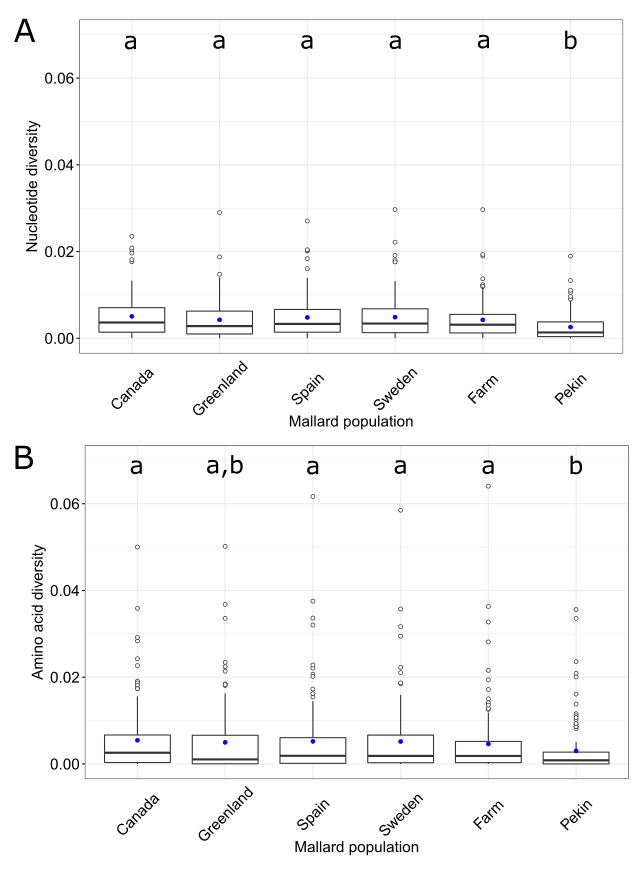
**

#### **Figure S1.** Boxplots showing **A)** nucleotide diversity and **B)** amino acid diversity for each mallard population. The box shows the median and the 25% and 75% quantile. The lower whisker shows the smallest observation greater than or equal to lower hinge - 1.5 * IQR, while the upper whisker shows the largest observation less than or equal to upper hinge + 1.5 * IQR. The blue dots show the mean, and the open circles mark outliers. Medians with different letters were significantly different (p<0.05, Kruskal-Wallis non-parametric ANOVA, Wilcoxon rank sum test, with FDR correction, Note S1).

#### **
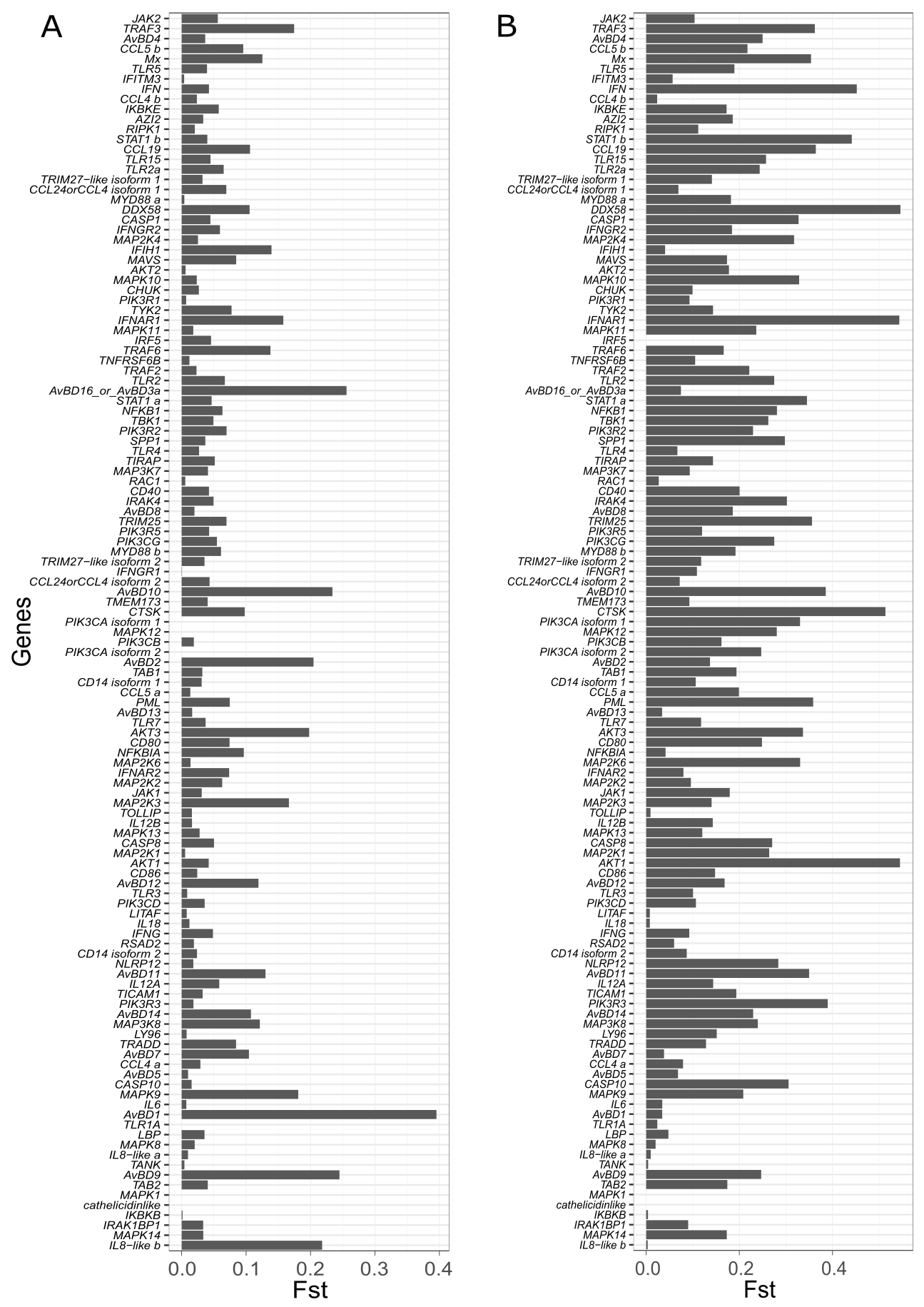
Figure S2.** Average genetic differentiation (F_ST_) per gene between **A)** wild and farm mallards (Canadian, Greenlandic, Spanish and Swedish mallards combined vs. farm mallards, and **B)** wild mallards and Pekin ducks (Canadian, Greenlandic, Spanish and Swedish mallards combined vs. Pekin ducks. Genes are sorted according to the F_ST_ value in wild mallards, which are listed in Table S9.


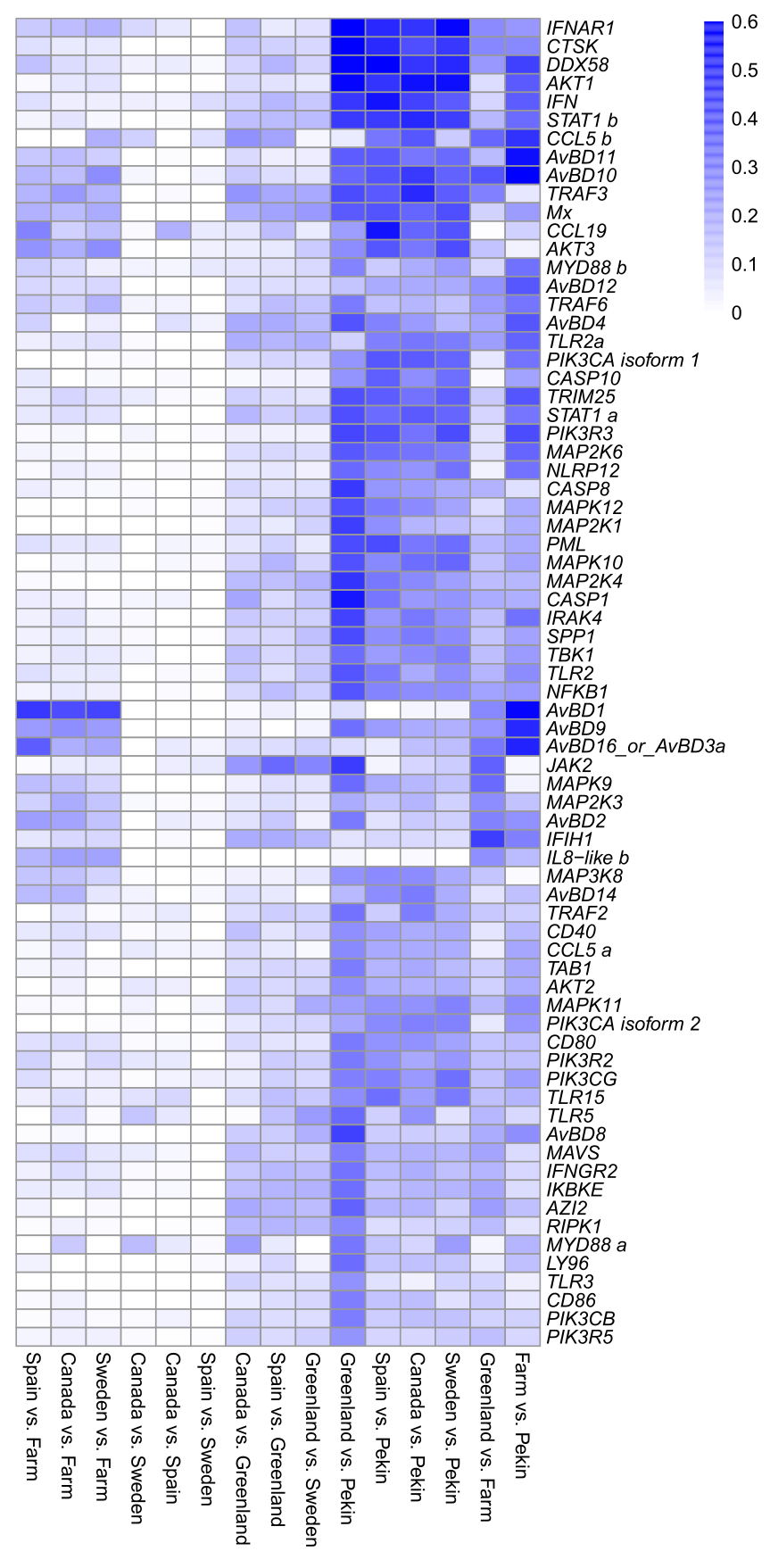


#### **Figure S3.** Pairwise genetic distance per gene, visualising all genes where F_ST_ > 0.25 in at least one pairwise comparison. The heatmap is clustered for rows and columns.


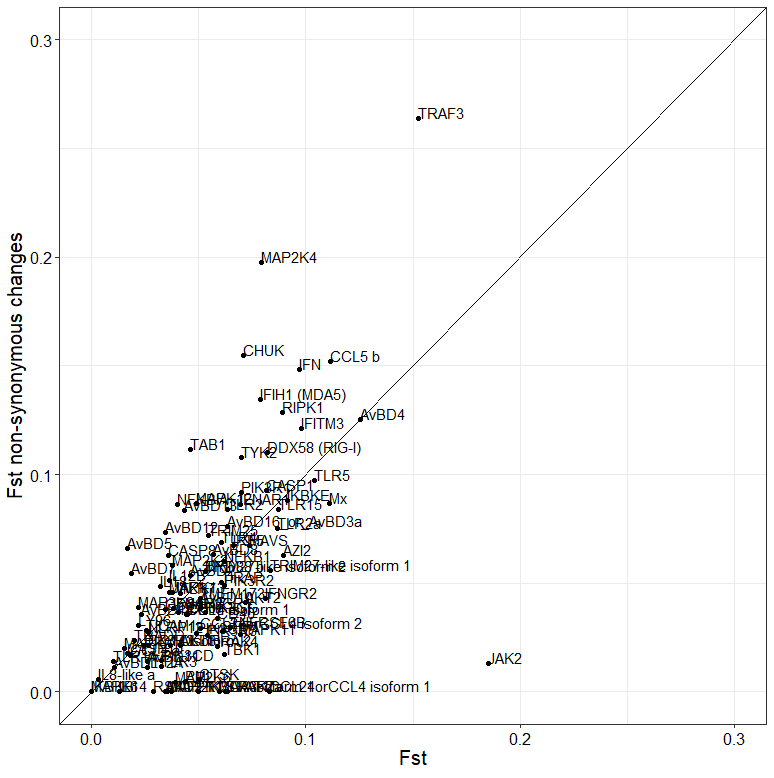


#### **Figure S4**. Genetic differentiation per gene in wild mallards based on all SNP positions (F_ST_) vs. non-synonymous SNPs only (F_ST_ non-synonymous changes). The black line denotes a 45° diagonal where each axis has the same value.


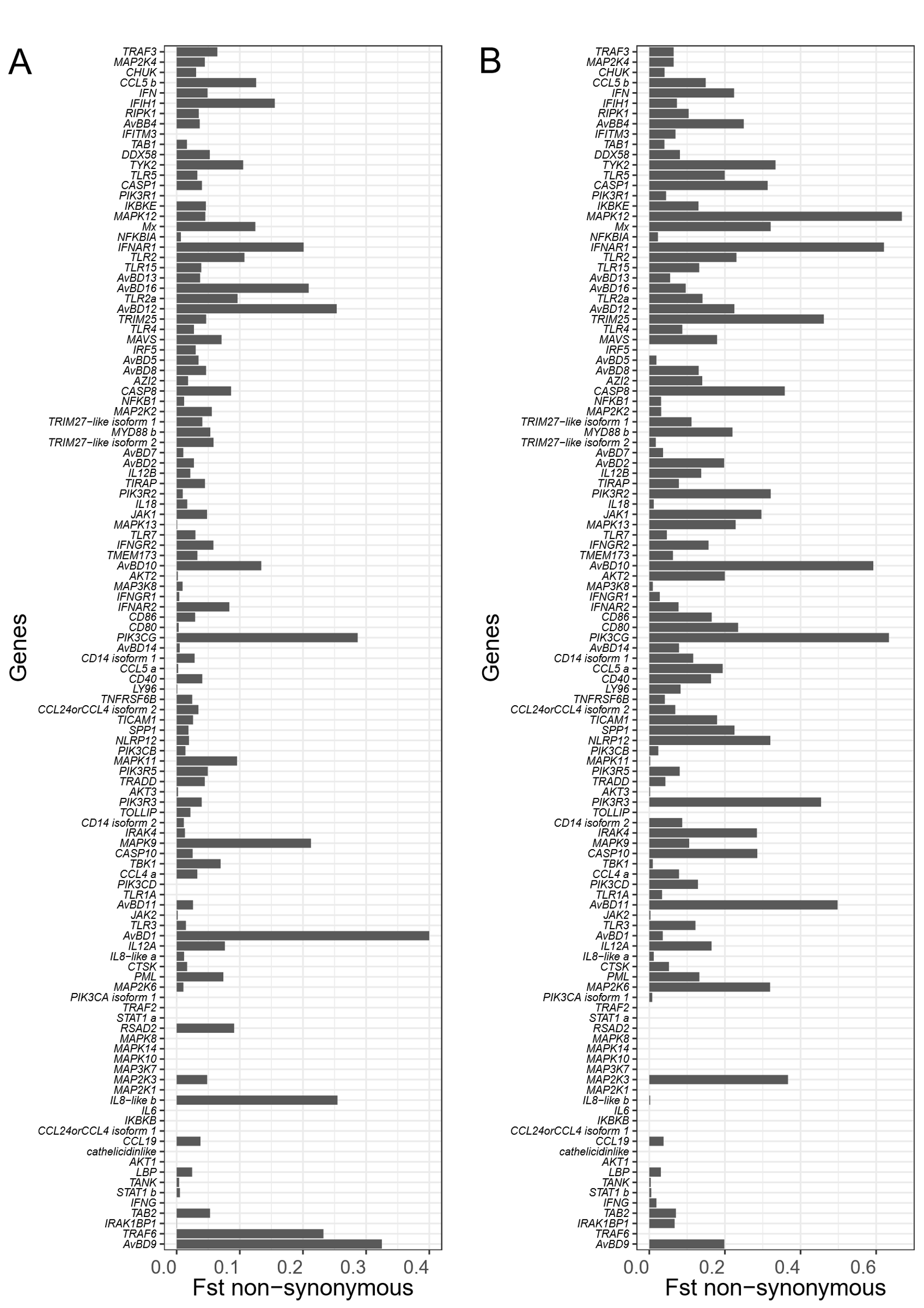


#### **Figure S5.** Average genetic differentiation (F_ST_) per gene between **A)** wild and farm mallards (Canadian, Greenlandic, Spanish and Swedish mallards combined vs. farm mallards, and **B)** wild mallards and Pekin ducks (Canadian, Greenlandic, Spanish and Swedish mallards combined vs. Pekin ducks when including non-synonymous changes only. The genes are sorted according to the F_ST_ value in wild mallards when including non-synonymous changes only, which are listed in Table S9.


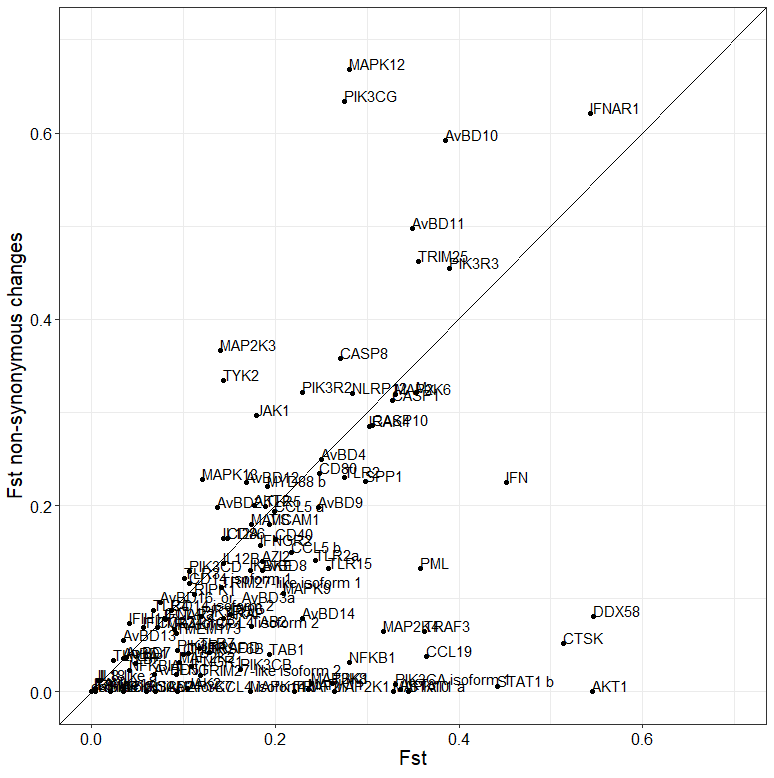


#### **Figure S6**. Genetic differentiation per gene in wild mallards vs. Pekin ducks based on all SNP positions (F_ST_) vs. non-synonymous SNPs only (F_ST_ non-synonymous changes). The black line denotes a 45° diagonal where each axis has the same value.


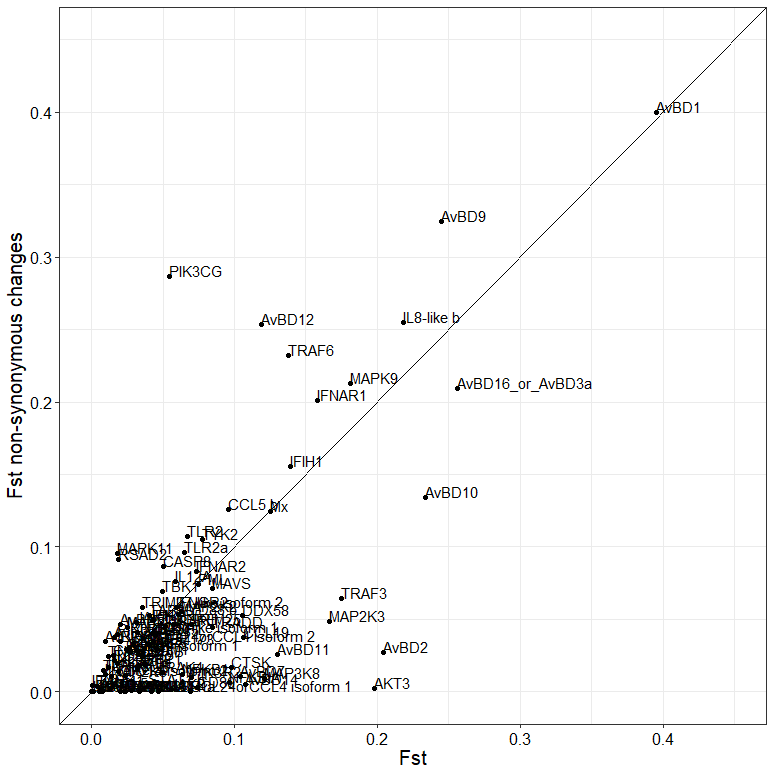


#### **Figure S7.** Genetic differentiation per gene in wild mallards vs. farm mallards based on all SNP positions (F_ST_) vs. non-synonymous SNPs only (F_ST_ non-synonymous changes). The black line denotes a 45° diagonal where each axis has the same value.


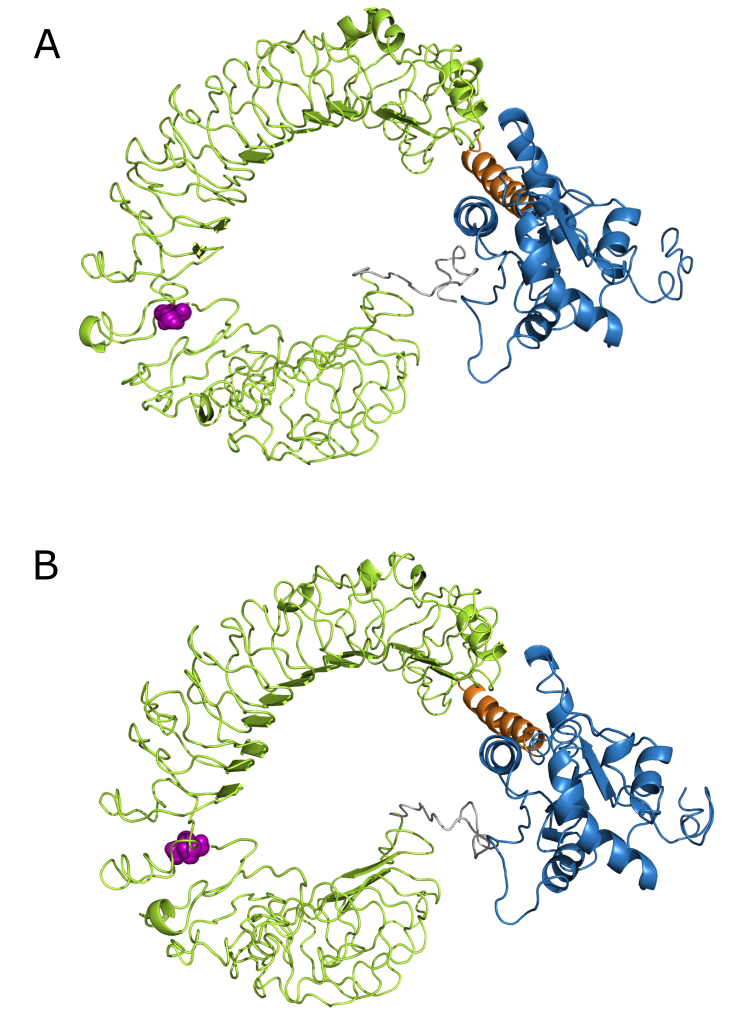


#### **Figure S8**. Predicted 3D structure of the mallard TLR15 protein. Signal peptide in grey, LRR ectodomain in green, transmembrane domain in orange and TIR-domain in blue. The non-synonymous amino acid change determined to be under positive selection in the BayeScan analysis of wild mallards, alanine (model **A**) to valine (model **B**) at position 261 of the mallard TLR15 CDS, is shown in purple. For more details see main text and Table S21.


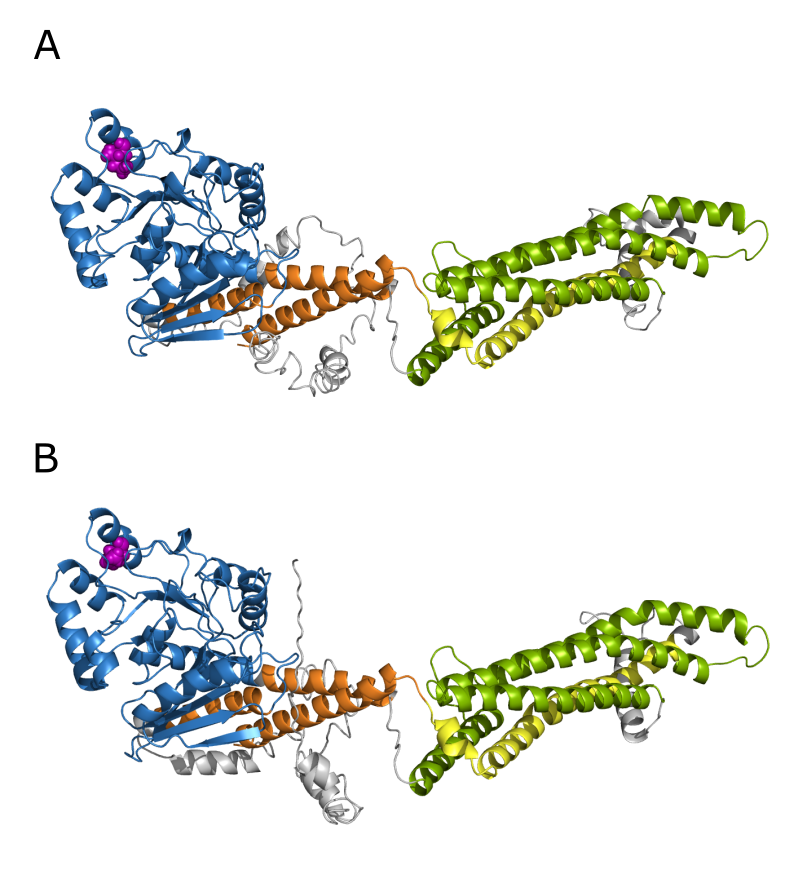


#### **Figure S9.** Predicted 3D structure of the mallard Mx protein. Bundle signalling element (BSE) in orange, G-domain in blue, Middle stalk domain in green, and GTPase effector domain in yellow. The non-synonymous amino acid change determined to be under positive selection in the BayeScan analysis of wild and domesticated mallards, isoleucine (model **A**) to valine (model **B**) at position 322 of the mallard Mx CDS, is shown in purple. For more details see main text and table S22.


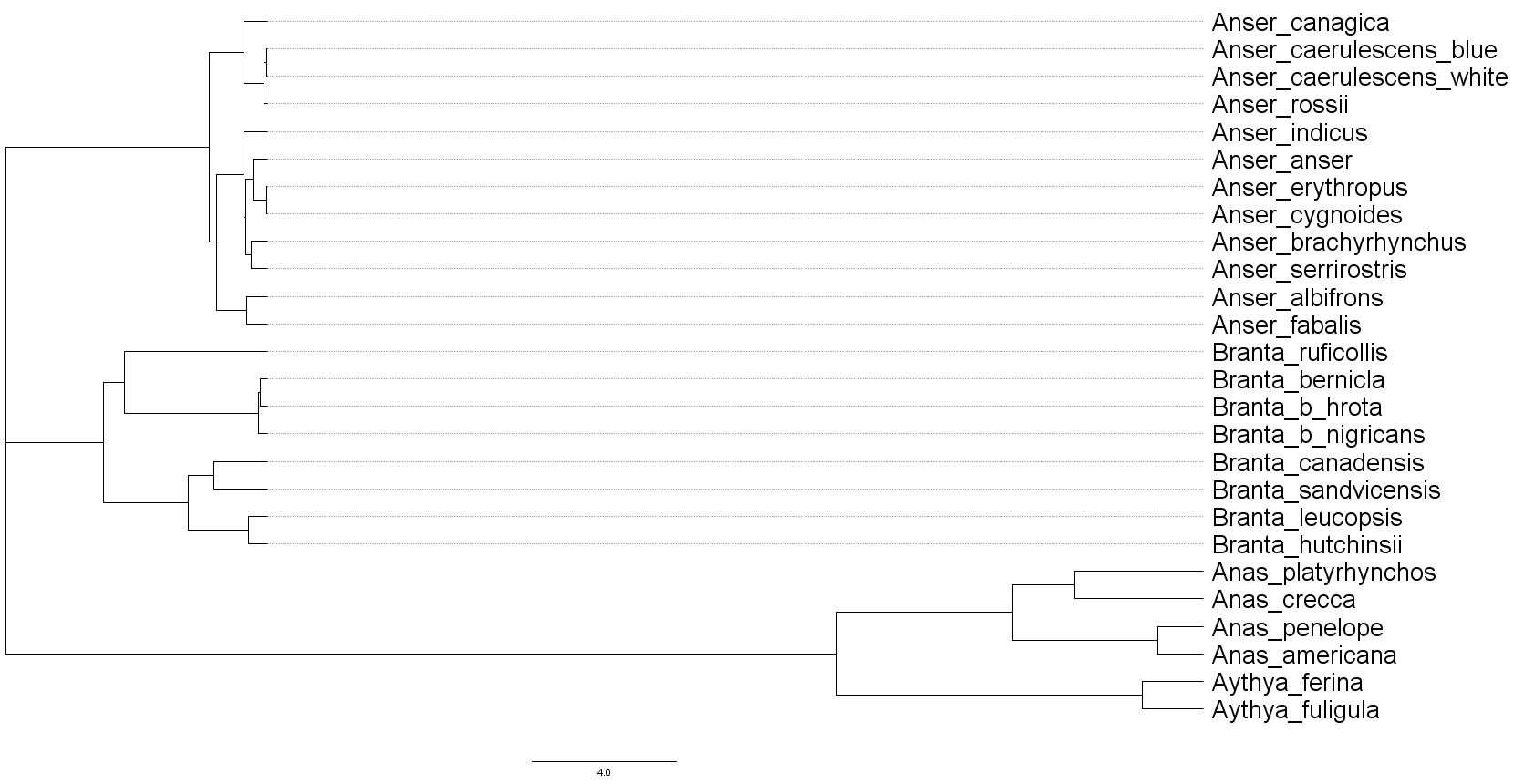


#### **Figure S10**. Phylogenetic tree used for selection analysis in PAML and HyPhy. The phylogenetic tree is the summary of 10000 trees downloaded from <http://birdtree.org> (Jetz et al. 2012)

## Supplementary tables

#### Table S1 List of the immune genes included in the capture

| Ensembl ID | Gene ID | Chromosome/scaffold name | Gene start (bp) | Gene end (bp) | Gene description (Ensembl release 91, Anas platyrhynchos) |
| --- | --- | --- | --- | --- | --- |
| ENSAPLG00000016314 | AKT1 | KB743650.1 | 139360 | 175814 | AKT serine/threonine kinase 1 [Source:HGNC Symbol;Acc:HGNC:391] |
| ENSAPLG00000011477 | AKT3 | KB742963.1 | 423821 | 483138 | AKT serine/threonine kinase 3 [Source:HGNC Symbol;Acc:HGNC:393] |
| ENSAPLG00000012380 | AZI2 | KB742966.1 | 2107270 | 2121829 | 5-azacytidine induced 2 [Source:HGNC Symbol;Acc:HGNC:24002] |
| ENSAPLG00000015244 | CASP10 | KB742719.1 | 78529 | 86721 | caspase 10 [Source:HGNC Symbol;Acc:HGNC:1500] |
| ENSAPLG00000014872 | CASP8 | KB742719.1 | 55431 | 60677 | caspase 8 [Source:HGNC Symbol;Acc:HGNC:1509] |
| ENSAPLG00000013558 | CD40 | KB743810.1 | 291672 | 294699 | CD40 molecule [Source:HGNC Symbol;Acc:HGNC:11919] |
| ENSAPLG00000005913 | CD86 | KB744434.1 | 31920 | 41134 | CD86 molecule [Source:HGNC Symbol;Acc:HGNC:1705] |
| ENSAPLG00000011503 | CHUK | KB742907.1 | 23134 | 54201 | conserved helix-loop-helix ubiquitous kinase [Source:HGNC Symbol;Acc:HGNC:1974] |
| ENSAPLG00000012768 | CTSK | KB744049.1 | 12821 | 15312 | cathepsin K [Source:HGNC Symbol;Acc:HGNC:2536] |
| ENSAPLG00000013187 | DHX58 | KB743181.1 | 355201 | 359586 | DExH-box helicase 58 [Source:HGNC Symbol;Acc:HGNC:29517] |
| ENSAPLG00000011284 | FOS | KB742685.1 | 132403 | 134032 | Fos proto-oncogene, AP-1 transcription factor subunit [Source:HGNC Symbol;Acc:HGNC:3796] |
| ENSAPLG00000010913 | IFIH1 | KB744072.1 | 1085549 | 1111688 | interferon induced with helicase C domain 1 [Source:HGNC Symbol;Acc:HGNC:18873] |
| ENSAPLG00000011322 | IFNAR1 | KB742682.1 | 794345 | 807422 | interferon alpha and beta receptor subunit 1 [Source:HGNC Symbol;Acc:HGNC:5432] |
| ENSAPLG00000012876 | IFNG | KB744042.1 | 347538 | 351885 | interferon gamma [Source:HGNC Symbol;Acc:HGNC:5438] |
| ENSAPLG00000002012 | IFNGR1 | KB742937.1 | 243681 | 253980 | interferon gamma receptor 1 [Source:HGNC Symbol;Acc:HGNC:5439] |
| ENSAPLG00000011237 | IFNGR2 | KB742682.1 | 766665 | 775159 | interferon gamma receptor 2 [Source:HGNC Symbol;Acc:HGNC:5440] |
| ENSAPLG00000006379 | IKBKB | KB744812.1 | 409767 | 420934 | inhibitor of nuclear factor kappa B kinase subunit beta [Source:HGNC Symbol;Acc:HGNC:5960] |
| ENSAPLG00000014351 | IKBKE | KB742563.1 | 635378 | 644771 | inhibitor of nuclear factor kappa B kinase subunit epsilon [Source:HGNC Symbol;Acc:HGNC:14552] |
| ENSAPLG00000006147 | IL12A | KB744208.1 | 566466 | 568812 | interleukin 12A [Source:HGNC Symbol;Acc:HGNC:5969] |
| ENSAPLG00000009092 | IL12B | KB742414.1 | 396397 | 403983 | interleukin 12B [Source:HGNC Symbol;Acc:HGNC:5970] |
| ENSAPLG00000005942 | IL18 | KB746795.1 | 112538 | 114574 | interleukin 18 [Source:HGNC Symbol;Acc:HGNC:5986] |
| ENSAPLG00000011754 | IL6 | KB743177.1 | 1054878 | 1057794 | interleukin 6 [Source:HGNC Symbol;Acc:HGNC:6018] |
| ENSAPLG00000004376 | IRAK1BP1 | KB742953.1 | 321038 | 421223 | interleukin 1 receptor associated kinase 1 binding protein 1 [Source:HGNC Symbol;Acc:HGNC:17368] |
| ENSAPLG00000012255 | IRAK4 | KB742451.1 | 1774293 | 1786587 | interleukin 1 receptor associated kinase 4 [Source:HGNC Symbol;Acc:HGNC:17967] |
| ENSAPLG00000006203 | IRF5 | KB743988.1 | 21711 | 25833 | interferon regulatory factor 5 [Source:HGNC Symbol;Acc:HGNC:6120] |
| ENSAPLG00000012752 | IRF7 | KB742884.1 | 385356 | 387613 | interferon regulatory factor 7 [Source:HGNC Symbol;Acc:HGNC:6122] |
| ENSAPLG00000012047 | JAK1 | KB743412.1 | 1070 | 20199 | Janus kinase 1 [Source:HGNC Symbol;Acc:HGNC:6190] |
| ENSAPLG00000004748 | JAK2 | KB742640.1 | 386552 | 430950 | Janus kinase 2 [Source:HGNC Symbol;Acc:HGNC:6192] |
| ENSAPLG00000005928 | JUN | KB743412.1 | 2014943 | 2015905 | Jun proto-oncogene, AP-1 transcription factor subunit [Source:HGNC Symbol;Acc:HGNC:6204] |
| ENSAPLG00000002299 | LITAF | KB744198.1 | 63012 | 65879 | lipopolysaccharide induced TNF factor [Source:HGNC Symbol;Acc:HGNC:16841] |
| ENSAPLG00000004845 | LY96 | KB743235.1 | 169963 | 175410 | lymphocyte antigen 96 [Source:HGNC Symbol;Acc:HGNC:17156] |
| ENSAPLG00000015480 | MAP2K1 | KB742800.1 | 574303 | 596101 | mitogen-activated protein kinase kinase 1 [Source:HGNC Symbol;Acc:HGNC:6840] |
| ENSAPLG00000007148 | MAP2K3 | KB742987.1 | 145084 | 157802 | mitogen-activated protein kinase kinase 3 [Source:HGNC Symbol;Acc:HGNC:6843] |
| ENSAPLG00000013074 | MAP2K4 | KB742686.1 | 50688 | 114279 | mitogen-activated protein kinase kinase 4 [Source:HGNC Symbol;Acc:HGNC:6844] |
| ENSAPLG00000005826 | MAP2K6 | KB743912.1 | 233901 | 251452 | mitogen-activated protein kinase kinase 6 [Source:HGNC Symbol;Acc:HGNC:6846] |
| ENSAPLG00000002833 | MAP2K7 | KB743363.1 | 327 | 2528 | mitogen-activated protein kinase kinase 7 [Source:HGNC Symbol;Acc:HGNC:6847] |
| ENSAPLG00000002702 | MAP3K7 | KB742594.1 | 899223 | 935792 | mitogen-activated protein kinase kinase kinase 7 [Source:HGNC Symbol;Acc:HGNC:6859] |
| ENSAPLG00000009205 | MAP3K8 | KB742630.1 | 58050 | 72273 | mitogen-activated protein kinase kinase kinase 8 [Source:HGNC Symbol;Acc:HGNC:6860] |
| ENSAPLG00000006116 | MAPK1 | KB743228.1 | 670282 | 684011 | mitogen-activated protein kinase 1 [Source:HGNC Symbol;Acc:HGNC:6871] |
| ENSAPLG00000005856 | MAPK10 | KB742619.1 | 3962587 | 4019261 | mitogen-activated protein kinase 10 [Source:HGNC Symbol;Acc:HGNC:6872] |
| ENSAPLG00000004462 | MAPK11 | KB743204.1 | 2155932 | 2174086 | mitogen-activated protein kinase 11 [Source:HGNC Symbol;Acc:HGNC:6873] |
| ENSAPLG00000004157 | MAPK12 | KB743204.1 | 2114254 | 2146062 | mitogen-activated protein kinase 12 [Source:HGNC Symbol;Acc:HGNC:6874] |
| ENSAPLG00000002168 | MAPK13 | KB743510.1 | 63 | 12637 | mitogen-activated protein kinase 13 [Source:HGNC Symbol;Acc:HGNC:6875] |
| ENSAPLG00000002603 | MAPK14 | KB743510.1 | 17400 | 34232 | mitogen-activated protein kinase 14 [Source:HGNC Symbol;Acc:HGNC:6876] |
| ENSAPLG00000016448 | MAPK8 | KB743335.1 | 859286 | 881206 | mitogen-activated protein kinase 8 [Source:HGNC Symbol;Acc:HGNC:6881] |
| ENSAPLG00000013460 | MAPK9 | KB743857.1 | 225455 | 241776 | mitogen-activated protein kinase 9 [Source:HGNC Symbol;Acc:HGNC:6886] |
| ENSAPLG00000013878 | NFKB1 | KB742932.1 | 441355 | 484880 | nuclear factor kappa B subunit 1 [Source:HGNC Symbol;Acc:HGNC:7794] |
| ENSAPLG00000011028 | NFKBIA | KB742730.1 | 790981 | 794030 | NFKB inhibitor alpha [Source:HGNC Symbol;Acc:HGNC:7797] |
| ENSAPLG00000014072 | NLRX1 | KB744100.1 | 74344 | 78435 | NLR family member X1 [Source:HGNC Symbol;Acc:HGNC:29890] |
| ENSAPLG00000012924 | PIK3CA | KB743644.1 | 1765833 | 1788081 | phosphatidylinositol-4,5-bisphosphate 3-kinase catalytic subunit alpha [Source:HGNC Symbol;Acc:HGNC:8975] |
| ENSAPLG00000003554 | PIK3CB | KB744477.1 | 683783 | 740675 | phosphatidylinositol-4,5-bisphosphate 3-kinase catalytic subunit beta [Source:HGNC Symbol;Acc:HGNC:8976] |
| ENSAPLG00000008183 | PIK3CD | KB742794.1 | 82414 | 103591 | phosphatidylinositol-4,5-bisphosphate 3-kinase catalytic subunit delta [Source:HGNC Symbol;Acc:HGNC:8977] |
| ENSAPLG00000006669 | PIK3CG | KB742471.1 | 563930 | 592062 | phosphatidylinositol-4,5-bisphosphate 3-kinase catalytic subunit gamma [Source:HGNC Symbol;Acc:HGNC:8978] |
| ENSAPLG00000004772 | PIK3R1 | KB742448.1 | 1039592 | 1090140 | phosphoinositide-3-kinase regulatory subunit 1 [Source:HGNC Symbol;Acc:HGNC:8979] |
| ENSAPLG00000016065 | PIK3R5 | KB746093.1 | 418890 | 441854 | phosphoinositide-3-kinase regulatory subunit 5 [Source:HGNC Symbol;Acc:HGNC:30035] |
| ENSAPLG00000003568 | RAC1 | KB743145.1 | 26643 | 33568 | Rac family small GTPase 1 [Source:HGNC Symbol;Acc:HGNC:9801] |
| ENSAPLG00000015420 | RIPK1 | KB743404.1 | 1374524 | 1392704 | receptor interacting serine/threonine kinase 1 [Source:HGNC Symbol;Acc:HGNC:10019] |
| ENSAPLG00000006016 | RSAD2 | KB743007.1 | 206857 | 212148 | radical S-adenosyl methionine domain containing 2 [Source:HGNC Symbol;Acc:HGNC:30908] |
| ENSAPLG00000012088 | SOCS3 | KB742551.1 | 286669 | 287303 | suppressor of cytokine signaling 3 [Source:HGNC Symbol;Acc:HGNC:19391] |
| ENSAPLG00000015131 | TAB1 | KB744410.1 | 60905 | 80777 | TGF-beta activated kinase 1 (MAP3K7) binding protein 1 [Source:HGNC Symbol;Acc:HGNC:18157] |
| ENSAPLG00000008173 | TAB2 | KB742386.1 | 415205 | 432269 | TGF-beta activated kinase 1/MAP3K7 binding protein 2 [Source:HGNC Symbol;Acc:HGNC:17075] |
| ENSAPLG00000007876 | TANK | KB744072.1 | 669954 | 692210 | TRAF family member associated NFKB activator [Source:HGNC Symbol;Acc:HGNC:11562] |
| ENSAPLG00000008445 | TBK1 | KB744799.1 | 49079 | 71477 | TANK binding kinase 1 [Source:HGNC Symbol;Acc:HGNC:11584] |
| ENSAPLG00000014359 | TICAM1 | KB744239.1 | 110073 | 112303 | toll like receptor adaptor molecule 1 [Source:HGNC Symbol;Acc:HGNC:18348] |
| ENSAPLG00000001925 | TIRAP | KB743106.1 | 212159 | 212984 | TIR domain containing adaptor protein [Source:HGNC Symbol;Acc:HGNC:17192] |
| ENSAPLG00000008976 | TLR3 | KB742391.1 | 217675 | 223928 | toll like receptor 3 [Source:HGNC Symbol;Acc:HGNC:11849] |
| ENSAPLG00000012625 | TLR4 | KB743815.1 | 116840 | 122803 | toll like receptor 4 [Source:HGNC Symbol;Acc:HGNC:11850] |
| ENSAPLG00000001279 | TLR5 | KB742799.1 | 310497 | 313067 | toll like receptor 5 [Source:HGNC Symbol;Acc:HGNC:11851] |
| ENSAPLG00000004139 | TLR7 | KB744694.1 | 21806 | 26090 | toll like receptor 7 [Source:HGNC Symbol;Acc:HGNC:15631] |
| ENSAPLG00000014631 | TMEM173 | KB742452.1 | 62286 | 66328 | transmembrane protein 173 [Source:HGNC Symbol;Acc:HGNC:27962] |
| ENSAPLG00000014394 | TNFRSF6B | KB744325.1 | 174535 | 179257 | TNF receptor superfamily member 6b [Source:HGNC Symbol;Acc:HGNC:11921] |
| ENSAPLG00000009156 | TOLLIP | KB743110.1 | 86024 | 103854 | toll interacting protein [Source:HGNC Symbol;Acc:HGNC:16476] |
| ENSAPLG00000005392 | TRADD | KB743829.1 | 1072476 | 1080797 | TNFRSF1A associated via death domain [Source:HGNC Symbol;Acc:HGNC:12030] |
| ENSAPLG00000016230 | TRAF2 | KB742736.1 | 655448 | 676020 | TNF receptor associated factor 2 [Source:HGNC Symbol;Acc:HGNC:12032] |
| ENSAPLG00000011933 | TRAF3 | KB743197.1 | 270352 | 297934 | TNF receptor associated factor 3 [Source:HGNC Symbol;Acc:HGNC:12033] |
| ENSAPLG00000013488 | TRAF6 | KB742537.1 | 865621 | 875533 | TNF receptor associated factor 6 [Source:HGNC Symbol;Acc:HGNC:12036] |
| ENSAPLG00000004947 | TRIM25 | KB742588.1 | 458691 | 466549 | tripartite motif containing 25 [Source:HGNC Symbol;Acc:HGNC:12932] |
| ENSAPLG00000000983 | cathelicidinlike | KB755733.1 | 16 | 177 | Novel gene |
| ENSAPLG00000014377 | CD14 | KB742452.1 | 22265 | 44368 | Novel gene |
| ENSAPLG00000003319 | MAVS | KB742464.1 | 2071992 | 2076188 | Novel gene |
| ENSAPLG00000009793 | CCL5 | KB742489.1 | 1579335 | 1580073 | Novel gene |
| ENSAPLG00000013262 | STAT1 | KB742542.1 | 882907 | 885554 | Novel gene |
| ENSAPLG00000006823 | PIK3R3 | KB742606.1 | 295222 | 346517 | Novel gene |
| ENSAPLG00000003004 | SPP1 | KB742619.1 | 3622446 | 3624837 | Novel gene |
| ENSAPLG00000012692 | IL8-like | KB742619.1 | 2217224 | 2219608 | Novel gene |
| ENSAPLG00000012697 | IL8-like | KB742619.1 | 2233587 | 2236693 | Novel gene |
| ENSAPLG00000003430 | IFN | KB742655.1 | 930474 | 931028 | Novel gene |
| ENSAPLG00000011364 | IFNAR2 | KB742682.1 | 837694 | 848168 | Novel gene |
| ENSAPLG00000009391 | CCL24/CCL4 | KB742811.1 | 451577 | 475770 | Novel gene |
| ENSAPLG00000009838 | CCL5 | KB742811.1 | 483423 | 485503 | Novel gene |
| ENSAPLG00000013556 | CASP1 | KB742811.1 | 820236 | 823436 | Novel gene |
| ENSAPLG00000007417 | CD80 | KB742931.1 | 1563029 | 1582485 | Novel gene |
| ENSAPLG00000002771 | TRIM27L ^a^ | KB742989.1 | 95007 | 108877 | Novel gene |
| ENSAPLG00000011045 | MAP2K2 | KB743016.1 | 255175 | 261771 | Novel gene |
| ENSAPLG00000009929 | TLR-15 | KB743040.1 | 2572220 | 2591289 | Novel gene |
| ENSAPLG00000011397 | TLR2 | KB743211.1 | 424317 | 426689 | Novel gene |
| ENSAPLG00000011399 | TLR2a | KB743211.1 | 432865 | 435216 | Novel gene |
| ENSAPLG00000001534 | PML | KB743248.1 | 82853 | 83458 | Novel gene |
| ENSAPLG00000005400 | CCL19 | KB743432.1 | 425482 | 427431 | Novel gene |
| ENSAPLG00000016235 | AKT2 | KB743761.1 | 140 | 5098 | Novel gene |
| ENSAPLG00000013226 | STAT1 | KB743811.1 | 230744 | 245451 | Novel gene |
| ENSAPLG00000015976 | Mx | KB743889.1 | 32453 | 62323 | Novel gene |
| ENSAPLG00000005139 | MYD88 | KB744154.1 | 102077 | 110088 | Novel gene |
| ENSAPLG00000011675 | PIK3R2 | KB744335.1 | 16861 | 28174 | Novel gene |
| ENSAPLG00000002049 | TLR1-A | KB744909.1 | 5476 | 7938 | Novel gene |
| ENSAPLG00000008655 | LBP | KB745027.1 | 25371 | 31584 | Novel gene |
| ENSAPLG00000010090 | NLRP12 | KB745096.1 | 3855 | 7596 | Novel gene |
| ENSAPLG00000012022 | TYK2 | KB745726.1 | 2981 | 13212 | Novel gene |
| ENSAPLG00000014726 | TLR21 | KB750090.1 | 2813 | 4757 | Novel gene |
| ENSAPLG00000015091 | DDX58 | KB751155.1 | 114375 | 133540 | Novel gene |
| ENSAPLG00000010194 | MYD88 | KB752419.1 | 1322 | 3057 | Novel gene |
| ENSAPLG00000012383 | CCL4 | KB819788.1 | 512 | 1080 | Novel gene |
| ENSAPLG00000012773 | CCL4 | KB820844.1 | 1 | 1135 | Novel gene |
| ENSAPLG00000001297 | AvBD4 | KB742693.1 | 10680 | 10808 | Novel gene |
| ENSAPLG00000012287 | AvBD1 | KB744359.1 | 116019 | 117844 | Novel gene |
| ENSAPLG00000011932 | AvBD10 | KB744359.1 | 86044 | 86503 | Novel gene |
| ENSAPLG00000011923 | AvBD11 | KB744359.1 | 63130 | 65532 | Novel gene |
| ENSAPLG00000011922 | AvBD12 | KB744359.1 | 57518 | 58188 | Novel gene |
| ENSAPLG00000011919 | AvBD13 | KB744359.1 | 50832 | 51892 | Novel gene |
| ENSAPLG00000012580 | AvBD14 | KB742693.1 | 18789 | 19108 | Novel gene |
| ENSAPLG00000012288 | AvBD16 (=AvBD3a) | KB744359.1 | 116388 | 123049 | Novel gene |
| ENSAPLG00000012251 | AvBD2 | KB744359.1 | 110444 | 113568 | Novel gene |
| ENSAPLG00000012465 | AvBD5 | KB742693.1 | 6740 | 7818 | Novel gene |
| ENSAPLG00000012239 | AvBD7 | KB744359.1 | 105819 | 106943 | Novel gene |
| ENSAPLG00000011572 | AvBD8 | KB744359.1 | 100429 | 100518 | Novel gene |
| ENSAPLG00000012056 | AvBD9 | KB744359.1 | 94514 | 96095 | Novel gene |
| ENSAPLG00000014996 | IFITM3 | KB745312.1 | 69809 | 71132 | Novel gene |

^a^ TRIM27L = TRIM27-like

#### Table S2 The number of sequencing reads and genome alignment statistics for each individual duck

| Individual | Population | Total sequencing reads | Mapped reads | Percentage of mapped reads |
| --- | --- | --- | --- | --- |
| CARM001_S1 | Canada | 1421094 | 1341092 | 94.37% |
| CARM002_S2 | Canada | 687342 | 656526 | 95.52% |
| CARM003_S3 | Canada | 2060960 | 1938930 | 94.08% |
| CARM007_S4 | Canada | 3847822 | 3485251 | 90.58% |
| CARM008_S5 | Canada | 2907842 | 2737976 | 94.16% |
| CARM009_S6 | Canada | 1637888 | 1549685 | 94.61% |
| CARM011_S7 | Canada | 2011538 | 1915494 | 95.23% |
| CARM012_S8 | Canada | 1373066 | 1310896 | 95.47% |
| CARM013_S9 | Canada | 1773304 | 1683669 | 94.95% |
| CARM014_S10 | Canada | 1887504 | 1798938 | 95.31% |
| CARM015_S11 | Canada | 1590320 | 1525566 | 95.93% |
| CARM017_S12 | Canada | 1618604 | 1557022 | 96.20% |
| CARM018_S13 | Canada | 2429240 | 2329850 | 95.91% |
| CARM019_S14 | Canada | 968524 | 931181 | 96.14% |
| CARM020_S15 | Canada | 1679414 | 1614383 | 96.13% |
| CASL001_S16 | Canada | 1937874 | 1848288 | 95.38% |
| ESDO006_S17 | Spain | 1817220 | 1746095 | 96.09% |
| ESDO021_S18 | Spain | 2199654 | 2109468 | 95.90% |
| ESDO028_S19 | Spain | 1947916 | 1861455 | 95.56% |
| ESDO038_S20 | Spain | 1538174 | 1457468 | 94.75% |
| ESDO045_S21 | Spain | 2242066 | 2130516 | 95.02% |
| ESDO051_S22 | Spain | 2523006 | 2379415 | 94.31% |
| ESDO053_S23 | Spain | 1493004 | 1376687 | 92.21% |
| ESDO071_S24 | Spain | 2307184 | 2172880 | 94.18% |
| ESDO085_S25 | Spain | 1446640 | 1372298 | 94.86% |
| ESDO092_S26 | Spain | 1027670 | 983029 | 95.66% |
| ESDO095_S27 | Spain | 1615050 | 1541692 | 95.46% |
| ESDO105_S28 | Spain | 3166500 | 3022669 | 95.46% |
| ESDO108_S29 | Spain | 2234062 | 2144112 | 95.97% |
| ESDO110_S30 | Spain | 1824426 | 1754607 | 96.17% |
| ESDO111_S31 | Spain | 1874168 | 1803518 | 96.23% |
| ESDO115_S32 | Spain | 1816600 | 1744099 | 96.01% |
| GLIS001_S33 | Greenland | 1906400 | 1821967 | 95.57% |
| GLIS002_S34 | Greenland | 1538516 | 1480599 | 96.24% |
| GLIS009_S35 | Greenland | 1874142 | 1784344 | 95.21% |
| GLNU009_S42 | Greenland | 2363774 | 2167974 | 91.72% |
| GLNU002_S36 | Greenland | 1798472 | 1725279 | 95.93% |
| GLNU003_S37 | Greenland | 1729828 | 1655561 | 95.71% |
| GLNU004_S38 | Greenland | 957644 | 915193 | 95.57% |
| GLNU005_S39 | Greenland | 1587542 | 1500165 | 94.50% |
| GLNU007_S40 | Greenland | 1373414 | 1307293 | 95.19% |
| GLNU008_S41 | Greenland | 2149846 | 2024539 | 94.17% |
| GLNU011_S43 | Greenland | 2406496 | 2255805 | 93.74% |
| GLNU012_S44 | Greenland | 1555788 | 1468197 | 94.37% |
| GLNU015_S45 | Greenland | 1677534 | 1595667 | 95.12% |
| GLNU019_S46 | Greenland | 1576072 | 1505897 | 95.55% |
| GLNU020_S47 | Greenland | 2036794 | 1928775 | 94.70% |
| GLNU021_S48 | Greenland | 1936520 | 1842754 | 95.16% |
| SEOB003_S49 | Sweden | 892990 | 857791 | 96.06% |
| SEOB005_S50 | Sweden | 917626 | 886144 | 96.57% |
| SEOB006_S51 | Sweden | 1956128 | 1878673 | 96.04% |
| SEOB007_S52 | Sweden | 1348326 | 1293299 | 95.92% |
| SEOB008_S53 | Sweden | 1671716 | 1610575 | 96.34% |
| SEOB009_S54 | Sweden | 1995164 | 1900711 | 95.27% |
| SEOB010_S55 | Sweden | 2305578 | 2214243 | 96.04% |
| SEOB011_S56 | Sweden | 1659506 | 1591715 | 95.91% |
| SEOB012_S57 | Sweden | 1828310 | 1745085 | 95.45% |
| SEOB013_S58 | Sweden | 1824616 | 1728353 | 94.72% |
| SEOB014_S59 | Sweden | 2389202 | 2273635 | 95.16% |
| SEOB016_S60 | Sweden | 2263652 | 2123544 | 93.81% |
| SEOB017_S61 | Sweden | 775164 | 717837 | 92.60% |
| SEOB018_S62 | Sweden | 1382542 | 1302741 | 94.23% |
| SEOB019_S63 | Sweden | 4894552 | 4645764 | 94.92% |
| SEOB020_S64 | Sweden | 2739066 | 2619598 | 95.64% |
| FARM001_S65 | Farm duck | 2798336 | 2686978 | 96.02% |
| FARM003_S66 | Farm duck | 1676222 | 1605141 | 95.76% |
| FARM004_S67 | Farm duck | 2685080 | 2572587 | 95.81% |
| FARM005_S68 | Farm duck | 1646790 | 1586712 | 96.35% |
| FARM011_S69 | Farm duck | 2046076 | 1973902 | 96.47% |
| FARM012_S70 | Farm duck | 1402824 | 1351275 | 96.33% |
| FARM014_S71 | Farm duck | 2080546 | 1995275 | 95.90% |
| FARM016_S72 | Farm duck | 1565166 | 1509248 | 96.43% |
| FARM017_S73 | Farm duck | 776504 | 740967 | 95.42% |
| FARM019_S74 | Farm duck | 560554 | 539688 | 96.28% |
| FARM020_S75 | Farm duck | 1737676 | 1667820 | 95.98% |
| FARM021_S76 | Farm duck | 3051770 | 2922466 | 95.76% |
| FARM023_S77 | Farm duck | 1890510 | 1797914 | 95.10% |
| FARM024_S78 | Farm duck | 2422746 | 2309147 | 95.31% |
| FARM027_S79 | Farm duck | 1867914 | 1765780 | 94.53% |
| FARM029_S80 | Farm duck | 2021054 | 1883713 | 93.20% |
| Pekin002_S81 | Pekin duck | 2080768 | 1969836 | 94.67% |
| Pekin004_S82 | Pekin duck | 1815642 | 1723507 | 94.93% |
| Pekin006_S83 | Pekin duck | 2019218 | 1924369 | 95.30% |
| Pekin009_S84 | Pekin duck | 1697404 | 1628495 | 95.94% |
| Pekin010_S85 | Pekin duck | 359346 | 344087 | 95.75% |
| Pekin012_S86 | Pekin duck | 948308 | 909915 | 95.95% |
| Pekin013_S87 | Pekin duck | 2797850 | 2702553 | 96.59% |
| Pekin014_S88 | Pekin duck | 4417278 | 4257772 | 96.39% |
| Pekin015_S89 | Pekin duck | 1976370 | 1904789 | 96.38% |
| Pekin016_S90 | Pekin duck | 1866496 | 1792275 | 96.02% |
| Pekin017_S91 | Pekin duck | 1785828 | 1722402 | 96.45% |
| Pekin022_S92 | Pekin duck | 1649570 | 1583923 | 96.02% |
| Pekin023_S93 | Pekin duck | 1616382 | 1553036 | 96.08% |
| Pekin024_S94 | Pekin duck | 1942906 | 1869773 | 96.24% |
| Pekin025_S95 | Pekin duck | 1648672 | 1585610 | 96.17% |
| Pekin027_S96 | Pekin duck | 1269086 | 1207717 | 95.16% |
| CSANAM001_S10 | *Anas americana* | 1322470 | 1200525 | 90.78% |
| CSANAM004_S11 | *Anas americana* | 1025900 | 966898 | 94.25% |
| CSANAM007_S12 | *Anas americana* | 1166836 | 1079646 | 92.53% |
| CSANCR001_S1 | *Anas crecca* | 1047536 | 962794 | 91.91% |
| CSANCR002_S2 | *Anas crecca* | 1034808 | 972308 | 93.96% |
| CSANCR003_S3 | *Anas crecca* | 980886 | 906236 | 92.39% |
| CSANCR007_S16 | *Anas crecca* | 1314732 | 1186032 | 90.21% |
| CSANPE001_S4 | *Anas penelope* | 1136556 | 1063224 | 93.55% |
| CSANPE002_S5 | *Anas penelope* | 1061512 | 971800 | 91.55% |
| CSANPE003_S6 | *Anas penelope* | 1324564 | 1218775 | 92.01% |
| CSAYFE003_S13 | *Aythya ferina* | 960114 | 883382 | 92.01% |
| CSAYFE005_S14 | *Aythya ferina* | 1034774 | 949222 | 91.73% |
| CSAYFE006_S15 | *Aythya ferina* | 1380110 | 1202484 | 87.13% |
| CSAYFU001_S7 | *Aythya fuligula* | 1014716 | 927697 | 91.42% |
| CSAYFU002_S8 | *Aythya fuligula* | 1839696 | 1593003 | 86.59% |
| CSAYFU005_S9 | *Aythya fuligula* | 1365346 | 1206537 | 88.37% |

CARM, CASL = wild mallard from Canada

ESDO = wild mallard from Spain

GLIS and GLNU = wild mallard from Greenland

SEOB = wild mallard from Sweden

FARM = Farm mallard

Pekin = Pekin duck

CSANAM = *Anas americana*

CSANCR = *Anas crecca*

CSANPE = *Anas penelope*

CSAYFE = *Aythya ferina*

CSAYFU = *Aythya fuligula*

#### Table S3 Average sequencing depth for the protein coding sequence for each gene for the mallards and the remaining duck species

| Ensembl ID | Gene ID | Scaffold | Average sequencing depth | SD sequencing depth | Average sequencing depth | SD sequencing depth | Average sequencing depth | SD sequencing depth | Average sequencing depth | SD sequencing depth |
| --- | --- | --- | --- | --- | --- | --- | --- | --- | --- | --- |
|  |  |  | wild mallards (n=64) | wild mallards (n=64) | farm mallards (n=16) | farm mallards (n=16) | Pekin ducks (n=16) | Pekin ducks (n=16) | remaining duck species (n=16) | remaining duck species (n=16) |
| ENSAPLG00000000983 | cathelicidinlike | KB755733.1 | 10.55 | 5.25 | 12.58 | 8.38 | 10.52 | 4.31 | 2.85 | 1.42 |
| ENSAPLG00000001279 | TLR5 | KB742799.1 | 244.53 | 80.73 | 259.88 | 97.20 | 265.90 | 135.54 | 155.76 | 17.15 |
| ENSAPLG00000001297 | AvBD4 | KB742693.1 | 41.16 | 14.87 | 41.17 | 18.47 | 31.89 | 15.32 | 26.92 | 7.20 |
| ENSAPLG00000001534 | PML | KB743248.1 | 40.31 | 13.20 | 39.87 | 17.92 | 46.29 | 25.48 | 25.93 | 9.61 |
| ENSAPLG00000001925 | TIRAP | KB743106.1 | 15.37 | 5.92 | 15.13 | 7.45 | 14.84 | 7.03 | 9.24 | 3.38 |
| ENSAPLG00000002012 | IFNGR1 | KB742937.1 | 202.81 | 68.17 | 222.64 | 85.59 | 219.25 | 109.17 | 132.95 | 16.84 |
| ENSAPLG00000002049 | TLR1A | KB744909.1 | 182.19 | 61.23 | 178.99 | 70.63 | 179.54 | 84.68 | 116.49 | 21.33 |
| ENSAPLG00000002168 | MAPK13 | KB743510.1 | 129.18 | 41.72 | 137.96 | 55.30 | 137.47 | 63.32 | 90.78 | 17.80 |
| ENSAPLG00000002299 | LITAF | KB744198.1 | 170.54 | 58.14 | 162.58 | 59.57 | 175.49 | 82.89 | 130.39 | 24.60 |
| ENSAPLG00000002603 | MAPK14 | KB743510.1 | 176.70 | 56.95 | 187.89 | 74.30 | 195.90 | 93.85 | 123.46 | 17.13 |
| ENSAPLG00000002702 | MAP3K7 | KB742594.1 | 221.08 | 73.32 | 239.36 | 93.55 | 240.84 | 121.32 | 130.34 | 12.42 |
| ENSAPLG00000002771 | TRIM27L ^a^ | KB742989.1 | 64.67 | 21.12 | 67.25 | 28.53 | 70.23 | 33.65 | 41.76 | 9.46 |
| ENSAPLG00000002833 | MAP2K7 | KB743363.1 | 4.20 | 1.99 | 3.88 | 2.42 | 5.09 | 3.01 | 1.98 | 1.44 |
| ENSAPLG00000003004 | SPP1 | KB742619.1 | 99.71 | 33.25 | 101.62 | 40.56 | 109.30 | 53.39 | 64.52 | 15.99 |
| ENSAPLG00000003319 | MAVS | KB742464.1 | 22.78 | 7.38 | 24.80 | 10.26 | 26.45 | 15.50 | 11.87 | 5.07 |
| ENSAPLG00000003430 | IFN | KB742655.1 | 135.21 | 68.89 | 187.26 | 75.87 | 129.40 | 98.33 | 92.87 | 30.52 |
| ENSAPLG00000003554 | PIK3CB | KB744477.1 | 223.32 | 75.67 | 220.13 | 79.68 | 240.44 | 117.52 | 143.72 | 14.27 |
| ENSAPLG00000003568 | RAC1 | KB743145.1 | 172.48 | 56.09 | 182.00 | 68.41 | 181.73 | 85.30 | 119.44 | 18.24 |
| ENSAPLG00000004139 | TLR7 | KB744694.1 | 228.94 | 75.10 | 231.05 | 88.39 | 236.91 | 119.22 | 151.03 | 19.68 |
| ENSAPLG00000004157 | MAPK12 | KB743204.1 | 183.89 | 59.69 | 193.01 | 74.64 | 196.51 | 97.39 | 122.24 | 18.04 |
| ENSAPLG00000004376 | IRAK1BP1 | KB742953.1 | 185.38 | 60.63 | 189.26 | 69.02 | 195.26 | 104.10 | 119.46 | 13.72 |
| ENSAPLG00000004462 | MAPK11 | KB743204.1 | 233.08 | 79.00 | 238.45 | 88.00 | 247.54 | 123.22 | 149.28 | 14.78 |
| ENSAPLG00000004748 | JAK2 | KB742640.1 | 162.69 | 85.38 | 243.39 | 97.32 | 160.18 | 124.93 | 108.76 | 35.36 |
| ENSAPLG00000004772 | PIK3R1 | KB742448.1 | 154.97 | 79.24 | 219.55 | 83.90 | 148.07 | 114.92 | 104.08 | 33.06 |
| ENSAPLG00000004845 | LY96 | KB743235.1 | 189.49 | 63.59 | 197.15 | 76.46 | 203.85 | 100.53 | 117.30 | 16.74 |
| ENSAPLG00000004947 | TRIM25 | KB742588.1 | 134.50 | 43.04 | 134.74 | 51.15 | 141.95 | 67.06 | 99.85 | 17.48 |
| ENSAPLG00000005139 | MYD88 b | KB744154.1 | 67.03 | 22.30 | 70.84 | 29.16 | 63.70 | 30.87 | 41.98 | 11.45 |
| ENSAPLG00000005392 | TRADD | KB743829.1 | 74.30 | 24.39 | 74.02 | 30.23 | 78.50 | 34.64 | 51.66 | 13.01 |
| ENSAPLG00000005400 | CCL19 | KB743432.1 | 11.92 | 5.85 | 16.90 | 7.07 | 14.02 | 11.84 | 9.03 | 4.30 |
| ENSAPLG00000005826 | MAP2K6 | KB743912.1 | 103.16 | 33.29 | 96.61 | 36.99 | 107.24 | 51.32 | 72.97 | 12.81 |
| ENSAPLG00000005856 | MAPK10 | KB742619.1 | 171.78 | 55.45 | 171.78 | 68.19 | 189.17 | 93.82 | 116.29 | 15.48 |
| ENSAPLG00000005913 | CD86 | KB744434.1 | 248.55 | 82.85 | 238.59 | 84.69 | 259.72 | 129.78 | 158.29 | 19.37 |
| ENSAPLG00000005928 | JUN | KB743412.1 | 21.87 | 7.08 | 22.78 | 9.59 | 23.34 | 10.84 | 17.54 | 4.20 |
| ENSAPLG00000005942 | IL18 | KB746795.1 | 208.26 | 69.13 | 185.68 | 71.15 | 213.43 | 103.59 | 132.29 | 15.57 |
| ENSAPLG00000006016 | RSAD2 | KB743007.1 | 196.33 | 64.35 | 190.37 | 70.24 | 206.65 | 104.93 | 128.57 | 18.03 |
| ENSAPLG00000006116 | MAPK1 | KB743228.1 | 219.29 | 72.20 | 236.47 | 92.94 | 234.43 | 113.06 | 149.59 | 13.84 |
| ENSAPLG00000006147 | IL12A | KB744208.1 | 125.16 | 41.52 | 124.86 | 47.61 | 133.82 | 57.26 | 79.14 | 17.89 |
| ENSAPLG00000006203 | IRF5 | KB743988.1 | 23.68 | 7.81 | 24.26 | 9.59 | 23.64 | 15.57 | 8.76 | 3.29 |
| ENSAPLG00000006379 | IKBKB | KB744812.1 | 188.69 | 61.92 | 170.09 | 59.53 | 188.74 | 91.95 | 129.37 | 22.72 |
| ENSAPLG00000006669 | PIK3CG | KB742471.1 | 191.05 | 63.58 | 205.48 | 79.15 | 204.51 | 98.24 | 125.74 | 17.07 |
| ENSAPLG00000006823 | PIK3R3 | KB742606.1 | 131.03 | 41.76 | 137.74 | 53.57 | 136.28 | 64.40 | 86.77 | 11.36 |
| ENSAPLG00000007148 | MAP2K3 | KB742987.1 | 36.84 | 12.17 | 38.20 | 15.64 | 37.56 | 18.21 | 24.36 | 6.09 |
| ENSAPLG00000007417 | CD80 | KB742931.1 | 111.98 | 36.45 | 116.16 | 47.55 | 119.72 | 58.64 | 79.64 | 18.29 |
| ENSAPLG00000007876 | TANK | KB744072.1 | 199.71 | 68.23 | 210.28 | 82.20 | 213.91 | 107.23 | 125.98 | 13.90 |
| ENSAPLG00000008173 | TAB2 | KB742386.1 | 199.81 | 65.68 | 221.82 | 85.61 | 213.18 | 105.28 | 139.62 | 22.55 |
| ENSAPLG00000008183 | PIK3CD | KB742794.1 | 145.32 | 47.59 | 145.39 | 58.03 | 153.57 | 68.27 | 101.95 | 19.53 |
| ENSAPLG00000008445 | TBK1 | KB744799.1 | 223.29 | 74.69 | 243.61 | 93.93 | 244.52 | 121.07 | 139.41 | 16.54 |
| ENSAPLG00000008655 | LBP | KB745027.1 | 69.51 | 27.67 | 58.60 | 20.64 | 94.56 | 60.03 | 43.64 | 24.52 |
| ENSAPLG00000008976 | TLR3 | KB742391.1 | 235.86 | 79.26 | 233.39 | 83.69 | 248.13 | 119.39 | 153.57 | 17.75 |
| ENSAPLG00000009092 | IL12B | KB742414.1 | 173.87 | 56.97 | 164.38 | 61.54 | 186.81 | 90.01 | 123.99 | 16.41 |
| ENSAPLG00000009156 | TOLLIP | KB743110.1 | 229.71 | 76.76 | 244.33 | 93.28 | 251.56 | 127.85 | 147.14 | 17.81 |
| ENSAPLG00000009205 | MAP3K8 | KB742630.1 | 166.86 | 54.37 | 172.92 | 64.94 | 177.44 | 80.31 | 120.79 | 15.39 |
| ENSAPLG00000009391 | CCL24^b^ | KB742811.1 | 132.63 | 42.68 | 123.17 | 47.53 | 144.65 | 79.31 | 88.65 | 25.09 |
| ENSAPLG00000009793 | CCL5 a | KB742489.1 | 24.59 | 8.59 | 21.77 | 9.01 | 37.04 | 16.57 | 18.66 | 8.22 |
| ENSAPLG00000009838 | CCL5 b | KB742811.1 | 125.28 | 41.08 | 113.44 | 45.66 | 132.15 | 61.52 | 89.80 | 23.89 |
| ENSAPLG00000009929 | TLR15 | KB743040.1 | 184.89 | 61.78 | 198.44 | 77.83 | 204.01 | 96.78 | 122.87 | 20.22 |
| ENSAPLG00000010090 | NLRP12 | KB745096.1 | 71.12 | 22.74 | 72.18 | 29.38 | 75.21 | 34.93 | 52.48 | 13.16 |
| ENSAPLG00000010194 | MYD88 a | KB752419.1 | 43.44 | 14.91 | 51.54 | 19.89 | 49.23 | 26.45 | 36.30 | 9.13 |
| ENSAPLG00000010913 | IFIH1 | KB744072.1 | 185.71 | 64.12 | 194.44 | 74.55 | 202.43 | 98.35 | 114.62 | 14.84 |
| ENSAPLG00000011028 | NFKBIA | KB742730.1 | 132.44 | 42.49 | 148.12 | 63.20 | 138.23 | 65.25 | 93.89 | 19.33 |
| ENSAPLG00000011045 | MAP2K2 | KB743016.1 | 56.79 | 18.81 | 56.61 | 22.76 | 58.83 | 27.82 | 39.46 | 8.94 |
| ENSAPLG00000011237 | IFNGR2 | KB742682.1 | 166.90 | 54.32 | 187.08 | 73.75 | 174.83 | 81.10 | 112.16 | 21.16 |
| ENSAPLG00000011284 | FOS | KB742685.1 | 12.17 | 3.36 | 13.04 | 4.81 | 12.74 | 5.15 | 9.09 | 2.66 |
| ENSAPLG00000011322 | IFNAR1 | KB742682.1 | 230.43 | 76.59 | 259.98 | 102.61 | 249.24 | 119.01 | 140.72 | 15.75 |
| ENSAPLG00000011364 | IFNAR2 | KB742682.1 | 196.74 | 63.89 | 223.31 | 91.75 | 211.85 | 98.43 | 127.31 | 17.80 |
| ENSAPLG00000011397 | TLR2 | KB743211.1 | 135.06 | 44.97 | 138.96 | 51.58 | 138.96 | 67.53 | 91.35 | 20.31 |
| ENSAPLG00000011399 | TLR2a | KB743211.1 | 122.49 | 40.47 | 119.45 | 46.93 | 129.58 | 62.83 | 79.31 | 10.40 |
| ENSAPLG00000011477 | AKT3 | KB742963.1 | 205.54 | 70.23 | 214.58 | 79.13 | 221.51 | 110.54 | 127.59 | 15.30 |
| ENSAPLG00000011503 | CHUK | KB742907.1 | 206.18 | 83.73 | 208.60 | 91.50 | 216.57 | 104.25 | 127.73 | 22.78 |
| ENSAPLG00000011572 | AvBD8 | KB744359.1 | 126.01 | 45.37 | 130.38 | 49.51 | 136.23 | 64.67 | 76.38 | 18.46 |
| ENSAPLG00000011675 | PIK3R2 | KB744335.1 | 21.55 | 6.91 | 22.58 | 9.52 | 22.08 | 10.54 | 12.62 | 4.82 |
| ENSAPLG00000011754 | IL6 | KB743177.1 | 102.15 | 34.75 | 104.67 | 41.60 | 109.47 | 47.09 | 67.58 | 10.63 |
| ENSAPLG00000011919 | AvBD13 | KB744359.1 | 63.46 | 22.35 | 66.54 | 28.90 | 62.33 | 34.44 | 41.69 | 11.55 |
| ENSAPLG00000011922 | AvBD12 | KB744359.1 | 84.59 | 26.90 | 82.69 | 34.16 | 85.87 | 48.18 | 48.75 | 15.35 |
| ENSAPLG00000011923 | AvBD11 | KB744359.1 | 218.34 | 71.99 | 210.90 | 79.06 | 227.80 | 111.19 | 148.18 | 30.55 |
| ENSAPLG00000011932 | AvBD10 | KB744359.1 | 119.66 | 40.01 | 113.40 | 42.14 | 137.23 | 81.90 | 74.70 | 18.49 |
| ENSAPLG00000011933 | TRAF3 | KB743197.1 | 203.18 | 68.40 | 215.10 | 79.40 | 215.05 | 107.43 | 138.51 | 15.91 |
| ENSAPLG00000012022 | TYK2 | KB745726.1 | 21.02 | 7.00 | 19.33 | 8.16 | 21.53 | 10.66 | 11.20 | 4.45 |
| ENSAPLG00000012047 | JAK1 | KB743412.1 | 182.56 | 61.77 | 191.15 | 73.43 | 198.57 | 93.49 | 115.60 | 14.40 |
| ENSAPLG00000012056 | AvBD9 | KB744359.1 | 177.23 | 57.30 | 180.10 | 67.76 | 177.86 | 81.23 | 119.45 | 24.55 |
| ENSAPLG00000012088 | SOCS3 | KB742551.1 | 2.31 | 0.88 | 2.24 | 0.91 | 2.52 | 1.41 | 1.53 | 0.63 |
| ENSAPLG00000012239 | AvBD7 | KB744359.1 | 64.64 | 21.45 | 62.98 | 23.16 | 68.30 | 29.41 | 43.89 | 11.36 |
| ENSAPLG00000012251 | AvBD2 | KB744359.1 | 209.87 | 81.12 | 191.03 | 65.90 | 221.23 | 114.52 | 128.45 | 23.18 |
| ENSAPLG00000012255 | IRAK4 | KB742451.1 | 187.25 | 62.66 | 199.22 | 78.05 | 197.35 | 94.70 | 120.26 | 17.27 |
| ENSAPLG00000012287 | AvBD1 | KB744359.1 | 26.69 | 12.90 | 22.51 | 12.48 | 40.67 | 19.12 | 16.89 | 9.49 |
| ENSAPLG00000012288 | AvBD16_or_AvBD3a | KB744359.1 | 163.73 | 54.89 | 162.46 | 62.41 | 166.48 | 82.65 | 121.17 | 27.35 |
| ENSAPLG00000012380 | AZI2 | KB742966.1 | 237.42 | 78.98 | 250.72 | 92.68 | 258.26 | 131.97 | 144.41 | 15.82 |
| ENSAPLG00000012383 | CCL4 b | KB819788.1 | 106.74 | 35.00 | 100.08 | 45.78 | 119.42 | 68.53 | 86.72 | 28.13 |
| ENSAPLG00000012465 | AvBD5 | KB742693.1 | 45.79 | 14.97 | 46.62 | 19.26 | 47.25 | 23.64 | 30.57 | 11.14 |
| ENSAPLG00000012580 | AvBD14 | KB742693.1 | 73.19 | 27.14 | 66.80 | 25.78 | 75.26 | 36.34 | 52.36 | 10.50 |
| ENSAPLG00000012625 | TLR4 | KB743815.1 | 169.54 | 54.97 | 153.92 | 56.89 | 174.94 | 82.63 | 114.94 | 20.07 |
| ENSAPLG00000012692 | IL8-like b | KB742619.1 | 175.77 | 57.79 | 175.00 | 65.92 | 182.11 | 89.20 | 112.37 | 17.79 |
| ENSAPLG00000012697 | IL8-like a | KB742619.1 | 155.51 | 52.91 | 153.38 | 56.28 | 161.82 | 86.74 | 93.95 | 12.56 |
| ENSAPLG00000012752 | IRF7 | KB742884.1 | 3.83 | 1.26 | 4.10 | 1.89 | 4.03 | 1.53 | 2.17 | 1.16 |
| ENSAPLG00000012768 | CTSK | KB744049.1 | 32.25 | 10.63 | 28.10 | 12.12 | 31.72 | 15.23 | 23.61 | 7.48 |
| ENSAPLG00000012773 | CCL4 a | KB820844.1 | 128.78 | 41.36 | 120.07 | 47.02 | 141.47 | 66.96 | 94.41 | 29.34 |
| ENSAPLG00000012876 | IFNG | KB744042.1 | 180.10 | 60.01 | 182.70 | 70.78 | 186.52 | 86.38 | 113.97 | 13.40 |
| ENSAPLG00000012924 | PIK3CA | KB743644.1 | 233.25 | 77.75 | 246.68 | 96.52 | 257.80 | 127.01 | 147.09 | 17.60 |
| ENSAPLG00000013074 | MAP2K4 | KB742686.1 | 218.71 | 72.37 | 211.72 | 77.97 | 232.56 | 114.55 | 146.58 | 19.93 |
| ENSAPLG00000013187 | DHX58 | KB743181.1 | 10.05 | 3.04 | 9.59 | 4.41 | 10.85 | 4.70 | 6.68 | 2.08 |
| ENSAPLG00000013226 | STAT1 b | KB743811.1 | 178.75 | 58.42 | 177.04 | 67.37 | 188.94 | 94.09 | 121.84 | 18.02 |
| ENSAPLG00000013262 | STAT1 a | KB742542.1 | 162.49 | 53.58 | 161.31 | 62.81 | 172.77 | 81.52 | 106.95 | 24.10 |
| ENSAPLG00000013460 | MAPK9 | KB743857.1 | 224.37 | 76.27 | 228.37 | 89.49 | 245.07 | 123.90 | 139.78 | 15.60 |
| ENSAPLG00000013488 | TRAF6 | KB742537.1 | 167.44 | 54.63 | 171.26 | 63.52 | 175.34 | 82.21 | 110.53 | 15.56 |
| ENSAPLG00000013556 | CASP1 | KB742811.1 | 39.14 | 13.18 | 38.90 | 17.14 | 45.00 | 20.85 | 26.34 | 8.06 |
| ENSAPLG00000013558 | CD40 | KB743810.1 | 14.42 | 4.83 | 13.54 | 6.21 | 14.62 | 7.20 | 8.20 | 3.43 |
| ENSAPLG00000013878 | NFKB1 | KB742932.1 | 185.67 | 61.41 | 195.15 | 74.74 | 201.57 | 98.68 | 116.54 | 16.58 |
| ENSAPLG00000014072 | NLRX1 | KB744100.1 | 6.40 | 2.25 | 6.31 | 2.53 | 7.62 | 4.66 | 3.33 | 1.38 |
| ENSAPLG00000014351 | IKBKE | KB742563.1 | 25.39 | 8.10 | 23.58 | 8.97 | 26.43 | 11.54 | 15.20 | 4.59 |
| ENSAPLG00000014359 | TICAM1 | KB744239.1 | 74.66 | 23.53 | 75.85 | 30.33 | 80.20 | 40.06 | 50.28 | 15.35 |
| ENSAPLG00000014377 | CD14 | KB742452.1 | 21.77 | 7.11 | 22.39 | 8.90 | 23.62 | 11.02 | 15.02 | 5.14 |
| ENSAPLG00000014394 | TNFRSF6B | KB744325.1 | 16.82 | 5.64 | 18.54 | 7.28 | 16.95 | 6.80 | 10.82 | 3.62 |
| ENSAPLG00000014631 | TMEM173 | KB742452.1 | 18.73 | 6.17 | 18.24 | 7.61 | 19.58 | 8.42 | 10.68 | 3.45 |
| ENSAPLG00000014726 | TLR21 | KB750090.1 | 5.07 | 1.80 | 4.27 | 1.72 | 4.74 | 2.29 | 2.92 | 0.82 |
| ENSAPLG00000014872 | CASP8 | KB742719.1 | 156.40 | 52.57 | 165.79 | 65.57 | 169.66 | 83.44 | 104.77 | 16.97 |
| ENSAPLG00000014996 | IFITM3 | KB745312.1 | 13.27 | 4.58 | 12.64 | 5.32 | 13.22 | 6.31 | 6.07 | 2.19 |
| ENSAPLG00000015091 | DDX58 | KB751155.1 | 151.82 | 77.49 | 201.91 | 78.45 | 147.63 | 114.19 | 109.95 | 35.14 |
| ENSAPLG00000015131 | TAB1 | KB744410.1 | 71.20 | 23.04 | 74.31 | 31.45 | 73.48 | 33.98 | 49.11 | 13.23 |
| ENSAPLG00000015244 | CASP10 | KB742719.1 | 202.83 | 67.62 | 216.11 | 84.40 | 220.60 | 108.14 | 135.96 | 18.28 |
| ENSAPLG00000015420 | RIPK1 | KB743404.1 | 173.72 | 55.91 | 177.78 | 68.49 | 185.59 | 84.25 | 111.30 | 15.13 |
| ENSAPLG00000015480 | MAP2K1 | KB742800.1 | 207.76 | 69.29 | 216.37 | 85.69 | 221.23 | 102.34 | 133.61 | 17.56 |
| ENSAPLG00000015976 | Mx | KB743889.1 | 150.49 | 49.09 | 154.21 | 59.01 | 159.89 | 76.73 | 100.39 | 13.08 |
| ENSAPLG00000016065 | PIK3R5 | KB746093.1 | 50.40 | 15.40 | 46.11 | 17.98 | 52.73 | 23.81 | 33.50 | 8.88 |
| ENSAPLG00000016230 | TRAF2 | KB742736.1 | 147.05 | 47.35 | 151.19 | 61.41 | 156.42 | 77.71 | 102.05 | 17.80 |
| ENSAPLG00000016235 | AKT2 | KB743761.1 | 23.66 | 8.16 | 17.97 | 6.95 | 24.96 | 12.06 | 6.36 | 2.41 |
| ENSAPLG00000016314 | AKT1 | KB743650.1 | 235.69 | 76.01 | 243.70 | 93.62 | 250.60 | 121.87 | 144.34 | 16.86 |
| ENSAPLG00000016448 | MAPK8 | KB743335.1 | 234.29 | 78.96 | 232.09 | 84.93 | 249.39 | 126.44 | 150.14 | 15.54 |

Remaining species = Anas crecca n=4, Anas penelope n=3, Anas americana n=3, Aythya ferina n=3, Aythya fuligula n=3

^a^ TRIM27L = TRIM27-like

^b^ CCL24 = CCL24orCCL4

#### Table S4 Included goose species

| Species | |
| --- | --- |
| Common Name | Scientific Name |
| GENUS ANSER |  |
| Greater White-fronted Goose | *A. albifrons* |
| Lesser White-fronted Goose | *A. erythropus* |
| Greylag Goose | *A. anser* |
| Swan Goose | *A. cygnoides* |
| Pink-footed Goose | *A. brachyrhynchus* |
| Taiga Bean Goose | *A. fabalis* |
| Tundra Bean Goose | *A. serrirostris* |
| Bar-headed Goose | *A. indicus* |
| Emperor Goose | *A. Canagicus* |
| Ross’ Goose | *A. rossii* |
| Blue Snow Goose | *A. caerulescens* |
| White Snow Goose | *A. caerulescens* |
|  |  |
| GENUS BRANTA |  |
| Dark-bellied Brent Goose | *B. bernicla bernicla* |
| Pale-bellied Brent Goose | *B. b. hrota* |
| Black Brent Goose | *B. b. nigricans* |
| Canada Goose | *B. canadensis* |
| Cackling Goose | *B. hutchinsii* |
| Barnacle Goose | *B. leucopsis* |
| Red-breasted Goose | *B. ruficollis* |
| Hawaii Goose | *B. sandvicensis* |

Data from (Ottenburghs et al. 2016; Ottenburghs et al. 2017). ENA accession number PRJEB20373.

#### Table S5 Genes with stopcodons in some species.

| Ensembl ID | Gene ID | Position of stopcodon | Species with stopcodon |
| --- | --- | --- | --- |
| ENSAPLG00000002702 | MAP3K7 | 6 | *Anser sp., Branta sp.* |
| ENSAPLG00000002771 | TRIM27L isoform1^a^ | 56 | *Anser sp.* |
| ENSAPLG00000002771 | TRIM27L isoform2^a^ | 300 | *Branta ruficollis* |
| ENSAPLG00000004376 | IRAK1BP1 | 23 | *Anser sp, Branta sp., Aythya sp., Anas platyrhynchos* |
| ENSAPLG00000004772 | PIK3R1 | 603 | *Anas crecca, Anas penelope, Anser albifrons, Anser brachyrhynchus, Anser caerulescens, Anser erythropus, Anser fabalis, Anser serrirostris, Aythya ferina, Aythya fuligula, Branta canadensis, Branta hutchinsii, Branta leucopsis, Branta sandvicensis* |
| ENSAPLG00000005139 | MYD88 | 301 | *Anser sp., Branta sp.* |
| ENSAPLG00000006203 | IRF5 | 2 | *Anas crecca, B. b. hrota* |
| ENSAPLG00000006823 | PIK3R3 | 267 | *B. b. bernicla, B. b. hrota, B. b, nigicans* |
| ENSAPLG00000009793 | CCL5 | 89 | *Anser albifrons, Anser anser, Anser brachyrhynchus, Anser fabalis, Anser serrirostris* |
| ENSAPLG00000010913 | IFIH1 | 84 | *Anser brachyrhynchus, Anser serrirostris* |
|  |  | 291 | *Anser sp., Aythya sp., Branta sp., Anas americana, Anas penelope* |
| ENSAPLG00000011237 | IFNGR2 | 17 | *Anser sp., Branta sp.* |
| ENSAPLG00000011322 | IFNAR1 | 6 | *Anser sp., Branta sp.* |
| ENSAPLG00000011923 | AvBD11 | 105 | *Aythya sp., Anas americana, Anas crecca, Anas penelope* |
| ENSAPLG00000012022 | TYK2 | 407 | *Branta sp.* |
| ENSAPLG00000012255 | IRAK4 | 356 | *Anser sp., Aythya sp., Branta sp.* |
| ENSAPLG00000012288 | AvBD16=AvBD3a | 4 | *Branta canadensis, Branta hutchinsii, Branta leucopsis, Branta ruficollis, Branta sandvicensis* |
| ENSAPLG00000012380 | AZI2 | 202 | *Aythya ferina* |
|  |  | 204 | *Branta ruficollis* |
|  |  | 205 | *Anser indicus* |
| ENSAPLG00000013074 | MAP2K4 | 15 | *Anser sp., Branta sp.* |
| ENSAPLG00000013187 | DHX58 | 243 | *Anser sp., Branta sp.* |
| ENSAPLG00000014072 | NLRX1 | 720 | *Anas americana, Anser anser* |
| ENSAPLG00000014377 | CD14 | 422 | *Branta ruficollis* |
| ENSAPLG00000014351 | IKBKE | 260 | *Anser sp., Branta sp.* |
| ENSAPLG00000016314 | AKT1 | 16 | *Anser sp., Branta sp.* |

*Anser sp.* = All included *Anser* species (n=12). *Branta sp*. = All included *Branta* species (n=8). *Aythya sp*. = All included *Aythya* species (n=2).

^a^ TRIM27L = TRIM27-like

#### Table S6 Per gene DNA polymorphism and neutrality statistics in the wild mallards.

| Ensembl ID | GeneID | n^hap^ | n^ind^ | Sites | S | Pi | TajimaD | SigD |
| --- | --- | --- | --- | --- | --- | --- | --- | --- |
| ENSAPLG00000000983 | cathelicidinlike | 76 | 38 | 162 | 1 | 0,000162443 | -1,058325 | n.s. |
| ENSAPLG00000001279 | TLR5 | 128 | 64 | 2571 | 101 | 0,005180004 | -0,961047 | n.s. |
| ENSAPLG00000001297 | AvBD4 | 128 | 64 | 129 | 7 | 0,006209752 | -1,068636 | n.s. |
| ENSAPLG00000001534 | PML | 124 | 62 | 606 | 44 | 0,008637292 | -1,149165 | n.s. |
| ENSAPLG00000001925 | TIRAP | 126 | 63 | 693 | 50 | 0,007513319 | -1,426236 | n.s. |
| ENSAPLG00000002012 | IFNGR1 | 128 | 64 | 1242 | 37 | 0,00294394 | -1,402259 | n.s. |
| ENSAPLG00000002049 | TLR1A | 128 | 64 | 2463 | 83 | 0,004790686 | -0,729783 | n.s. |
| ENSAPLG00000002168 | MAPK13 | 128 | 64 | 1086 | 43 | 0,004857252 | -1,118994 | n.s. |
| ENSAPLG00000002299 | LITAF | 128 | 64 | 447 | 1 | 6,94E-05 | -0,909227 | n.s. |
| ENSAPLG00000002603 | MAPK14 | 128 | 64 | 966 | 20 | 0,001771348 | -1,505924 | n.s. |
| ENSAPLG00000002702 | MAP3K7 | 128 | 64 | 1779 | 21 | 0,001309915 | -1,126391 | n.s. |
| ENSAPLG00000002771 | TRIM27L isoform 1^a^ | 128 | 64 | 891 | 79 | 0,00909715 | -1,478742 | n.s. |
| ENSAPLG00000002771 | TRIM27L isoform 2^a^ | 128 | 64 | 1506 | 146 | 0,012707699 | -1,032905 | n.s. |
| ENSAPLG00000003004 | SPP1 | 128 | 64 | 807 | 48 | 0,007131101 | -1,163614 | n.s. |
| ENSAPLG00000003319 | MAVS | 98 | 49 | 1896 | 241 | 0,019382272 | -0,816489 | n.s. |
| ENSAPLG00000003430 | IFN | 128 | 64 | 540 | 6 | 0,000590323 | -1,539185 | n.s. |
| ENSAPLG00000003554 | PIK3CB | 128 | 64 | 3210 | 48 | 0,001124186 | -1,829112 | * |
| ENSAPLG00000003568 | RAC1 | 128 | 64 | 543 | 6 | 0,000674068 | -1,446805 | n.s. |
| ENSAPLG00000004139 | TLR7 | 128 | 64 | 3147 | 63 | 0,001489553 | -1,897138 | * |
| ENSAPLG00000004157 | MAPK12 | 128 | 64 | 978 | 22 | 0,002865955 | -0,879719 | n.s. |
| ENSAPLG00000004376 | IRAK1BP1 | 128 | 64 | 378 | 18 | 0,007058037 | -0,541651 | n.s. |
| ENSAPLG00000004462 | MAPK11 | 128 | 64 | 987 | 28 | 0,00166909 | -1,999561 | * |
| ENSAPLG00000004748 | JAK2 | 128 | 64 | 3423 | 16 | 0,000347673 | -1,617681 | # |
| ENSAPLG00000004772 | PIK3R1 | 128 | 64 | 2172 | 17 | 0,000610737 | -1,580558 | # |
| ENSAPLG00000004845 | LY96 | 128 | 64 | 465 | 15 | 0,003763441 | -0,984257 | n.s. |
| ENSAPLG00000004947 | TRIM25 | 124 | 62 | 1809 | 32 | 0,001749879 | -1,395793 | n.s. |
| ENSAPLG00000005139 | MYD88 b | 128 | 64 | 969 | 61 | 0,010440374 | -0,320439 | n.s. |
| ENSAPLG00000005392 | TRADD | 128 | 64 | 912 | 53 | 0,0083969 | -0,718327 | n.s. |
| ENSAPLG00000005400 | CCL19 | 54 | 27 | 285 | 9 | 0,008189767 | 0,341535 | n.s. |
| ENSAPLG00000005826 | MAP2K6 | 128 | 64 | 1005 | 39 | 0,003330542 | -1,623097 | # |
| ENSAPLG00000005856 | MAPK10 | 128 | 64 | 1173 | 21 | 0,002710574 | -0,505451 | n.s. |
| ENSAPLG00000005913 | CD86 | 128 | 64 | 834 | 25 | 0,002108648 | -1,843022 | * |
| ENSAPLG00000005942 | IL18 | 128 | 64 | 612 | 4 | 0,00015198 | -1,662898 | # |
| ENSAPLG00000006016 | RSAD2 | 128 | 64 | 912 | 22 | 0,00236323 | -1,33473 | n.s. |
| ENSAPLG00000006116 | MAPK1 | 128 | 64 | 966 | 3 | 4,85E-05 | -1,573186 | # |
| ENSAPLG00000006147 | IL12A | 128 | 64 | 501 | 36 | 0,008302657 | -1,128856 | n.s. |
| ENSAPLG00000006203 | IRF5 | 72 | 36 | 1119 | 69 | 0,009527076 | -0,879118 | n.s. |
| ENSAPLG00000006379 | IKBKB | 128 | 64 | 2070 | 6 | 6,01E-05 | -1,919054 | * |
| ENSAPLG00000006669 | PIK3CG | 128 | 64 | 3321 | 95 | 0,003186631 | -1,269704 | n.s. |
| ENSAPLG00000006823 | PIK3R3 | 104 | 52 | 2169 | 97 | 0,004171156 | -1,698581 | # |
| ENSAPLG00000007148 | MAP2K3 | 128 | 64 | 897 | 57 | 0,00763097 | -1,16092 | n.s. |
| ENSAPLG00000007417 | CD80 | 128 | 64 | 951 | 41 | 0,004077801 | -1,488461 | n.s. |
| ENSAPLG00000007876 | TANK | 128 | 64 | 1266 | 8 | 0,000135179 | -2,068118 | * |
| ENSAPLG00000008173 | TAB2 | 128 | 64 | 2037 | 29 | 0,00096269 | -1,867001 | * |
| ENSAPLG00000008183 | PIK3CD | 128 | 64 | 3144 | 96 | 0,004593645 | -0,590132 | n.s. |
| ENSAPLG00000008445 | TBK1 | 128 | 64 | 2190 | 53 | 0,003488477 | -0,724025 | n.s. |
| ENSAPLG00000008655 | LBP | 128 | 64 | 1080 | 116 | 0,019708506 | -0,095737 | n.s. |
| ENSAPLG00000008976 | TLR3 | 128 | 64 | 2697 | 68 | 0,001546812 | -2,124414 | * |
| ENSAPLG00000009092 | IL12B | 128 | 64 | 963 | 12 | 0,00069654 | -1,78988 | * |
| ENSAPLG00000009156 | TOLLIP | 128 | 64 | 825 | 10 | 0,000936531 | -1,434275 | n.s. |
| ENSAPLG00000009205 | MAP3K8 | 128 | 64 | 1416 | 37 | 0,002948238 | -1,172501 | n.s. |
| ENSAPLG00000009391 | CCL24 isoform 1 ^b^ | 128 | 64 | 267 | 4 | 0,000816984 | -1,339948 | n.s. |
| ENSAPLG00000009391 | CCL24 isoform 2 ^b^ | 128 | 64 | 273 | 5 | 0,002147869 | -0,74443 | n.s. |
| ENSAPLG00000009793 | CCL5 a | 100 | 50 | 273 | 42 | 0,028066008 | -0,373597 | n.s. |
| ENSAPLG00000009838 | CCL5 b | 128 | 64 | 273 | 5 | 0,003733758 | 0,216986 | n.s. |
| ENSAPLG00000009929 | TLR15 | 128 | 64 | 2526 | 94 | 0,004463326 | -1,164971 | n.s. |
| ENSAPLG00000010090 | NLRP12 | 128 | 64 | 2538 | 142 | 0,004959071 | -1,743727 | # |
| ENSAPLG00000010194 | MYD88 a | 126 | 63 | 408 | 8 | 0,002557112 | -0,690638 | n.s. |
| ENSAPLG00000010913 | IFIH1 | 128 | 64 | 3039 | 35 | 0,000667295 | -2,062504 | * |
| ENSAPLG00000011028 | NFKBIA | 128 | 64 | 726 | 25 | 0,002309976 | -1,939084 | * |
| ENSAPLG00000011045 | MAP2K2 | 128 | 64 | 921 | 32 | 0,002067357 | -2,053845 | * |
| ENSAPLG00000011237 | IFNGR2 | 128 | 64 | 999 | 61 | 0,006938533 | -1,203895 | n.s. |
| ENSAPLG00000011322 | IFNAR1 | 128 | 64 | 1671 | 41 | 0,001252403 | -2,205813 | ** |
| ENSAPLG00000011364 | IFNAR2 | 128 | 64 | 1524 | 64 | 0,009814345 | 0,666456 | n.s. |
| ENSAPLG00000011397 | TLR2 | 128 | 64 | 2373 | 87 | 0,003920818 | -1,365575 | n.s. |
| ENSAPLG00000011399 | TLR2a | 128 | 64 | 2352 | 92 | 0,004397958 | -1,271594 | n.s. |
| ENSAPLG00000011477 | AKT3 | 128 | 64 | 1290 | 15 | 0,00118177 | -1,202985 | n.s. |
| ENSAPLG00000011503 | CHUK | 128 | 64 | 2100 | 43 | 0,001582169 | -1,780022 | * |
| ENSAPLG00000011572 | AvBD8 | 128 | 64 | 90 | 9 | 0,009355862 | -1,18629 | n.s. |
| ENSAPLG00000011675 | PIK3R2 | 84 | 42 | 2187 | 102 | 0,006016744 | -1,291599 | n.s. |
| ENSAPLG00000011754 | IL6 | 128 | 64 | 465 | 14 | 0,001330063 | -2,013257 | * |
| ENSAPLG00000011919 | AvBD13 | 128 | 64 | 180 | 14 | 0,004106518 | -1,964654 | * |
| ENSAPLG00000011922 | AvBD12 | 128 | 64 | 198 | 21 | 0,018929452 | -0,089721 | n.s. |
| ENSAPLG00000011923 | AvBD11 | 128 | 64 | 315 | 36 | 0,016787745 | -0,802973 | n.s. |
| ENSAPLG00000011932 | AvBD10 | 128 | 64 | 204 | 15 | 0,017480725 | 0,567362 | n.s. |
| ENSAPLG00000011933 | TRAF3 | 128 | 64 | 1704 | 23 | 0,001298184 | -1,506437 | n.s. |
| ENSAPLG00000012022 | TYK2 | 120 | 60 | 2904 | 157 | 0,005570243 | -1,493232 | n.s. |
| ENSAPLG00000012047 | JAK1 | 128 | 64 | 2886 | 110 | 0,004970285 | -1,0056 | n.s. |
| ENSAPLG00000012056 | AvBD9 | 128 | 64 | 192 | 9 | 0,012650457 | 1,118248 | n.s. |
| ENSAPLG00000012239 | AvBD7 | 128 | 64 | 198 | 10 | 0,00623546 | -0,986368 | n.s. |
| ENSAPLG00000012251 | AvBD2 | 128 | 64 | 177 | 8 | 0,008699091 | 0,103413 | n.s. |
| ENSAPLG00000012255 | IRAK4 | 110 | 55 | 1413 | 70 | 0,006235064 | -1,306998 | n.s. |
| ENSAPLG00000012287 | AvBD1 | 128 | 64 | 198 | 6 | 0,001893939 | -1,429243 | n.s. |
| ENSAPLG00000012288 | AvBD16_or_AvBD3a | 128 | 64 | 204 | 22 | 0,006411509 | -2,074931 | * |
| ENSAPLG00000012380 | AZI2 | 128 | 64 | 1173 | 51 | 0,003475509 | -1,757091 | # |
| ENSAPLG00000012383 | CCL4 b | 128 | 64 | 282 | 1 | 0,000216396 | -0,731198 | n.s. |
| ENSAPLG00000012465 | AvBD5 | 126 | 63 | 201 | 12 | 0,005515281 | -1,28778 | n.s. |
| ENSAPLG00000012580 | AvBD14 | 128 | 64 | 186 | 8 | 0,006433357 | -0,441032 | n.s. |
| ENSAPLG00000012625 | TLR4 | 128 | 64 | 2538 | 87 | 0,00283622 | -1,762142 | # |
| ENSAPLG00000012692 | IL8-like b | 128 | 64 | 315 | 6 | 0,000444085 | -1,888982 | * |
| ENSAPLG00000012697 | IL8-like a | 128 | 64 | 312 | 9 | 0,000793395 | -2,049642 | * |
| ENSAPLG00000012768 | CTSK | 128 | 64 | 1008 | 29 | 0,001469909 | -2,166789 | ** |
| ENSAPLG00000012773 | CCL4 a | 128 | 64 | 279 | 22 | 0,012658133 | -0,478503 | n.s. |
| ENSAPLG00000012876 | IFNG | 128 | 64 | 495 | 10 | 0,002148453 | -1,199262 | n.s. |
| ENSAPLG00000012924 | PIK3CA isoform 1 | 128 | 64 | 2034 | 30 | 0,00099145 | -1,880445 | * |
| ENSAPLG00000012924 | PIK3CA isoform 2 | 128 | 64 | 3204 | 45 | 0,000975267 | -1,915857 | * |
| ENSAPLG00000013074 | MAP2K4 | 128 | 64 | 1203 | 8 | 0,000492228 | -1,400105 | n.s. |
| ENSAPLG00000013226 | STAT1 b | 128 | 64 | 1743 | 56 | 0,003923385 | -1,091297 | n.s. |
| ENSAPLG00000013262 | STAT1 a | 128 | 64 | 540 | 35 | 0,009597368 | -0,591363 | n.s. |
| ENSAPLG00000013460 | MAPK9 | 128 | 64 | 1287 | 11 | 0,000557896 | -1,629028 | # |
| ENSAPLG00000013488 | TRAF6 | 128 | 64 | 1656 | 35 | 0,002363824 | -1,182469 | n.s. |
| ENSAPLG00000013556 | CASP1 | 128 | 64 | 1149 | 75 | 0,01168039 | -0,211594 | n.s. |
| ENSAPLG00000013558 | CD40 | 116 | 58 | 544 | 24 | 0,003737378 | -1,59949 | # |
| ENSAPLG00000013878 | NFKB1 | 128 | 64 | 2961 | 87 | 0,003497402 | -1,180131 | n.s. |
| ENSAPLG00000014351 | IKBKE | 128 | 64 | 2166 | 176 | 0,009697052 | -1,200805 | n.s. |
| ENSAPLG00000014359 | TICAM1 | 128 | 64 | 2184 | 179 | 0,01276587 | -0,552843 | n.s. |
| ENSAPLG00000014377 | CD14 isoform 2 | 128 | 64 | 792 | 76 | 0,010256042 | -1,383934 | n.s. |
| ENSAPLG00000014377 | CD14 isoform 1 | 126 | 63 | 1281 | 143 | 0,013341065 | -1,224196 | n.s. |
| ENSAPLG00000014394 | TNFRSF6B | 116 | 58 | 843 | 38 | 0,005313002 | -1,191528 | n.s. |
| ENSAPLG00000014631 | TMEM173 | 118 | 59 | 1131 | 100 | 0,011792051 | -0,976941 | n.s. |
| ENSAPLG00000014872 | CASP8 | 128 | 64 | 1443 | 22 | 0,001261606 | -1,569928 | # |
| ENSAPLG00000014996 | IFITM3 | 120 | 60 | 270 | 9 | 0,00291109 | -1,292865 | n.s. |
| ENSAPLG00000015091 | DDX58 | 128 | 64 | 2562 | 19 | 0,000600991 | -1,562704 | # |
| ENSAPLG00000015131 | TAB1 | 128 | 64 | 1689 | 70 | 0,003228612 | -1,904466 | * |
| ENSAPLG00000015244 | CASP10 | 128 | 64 | 1557 | 35 | 0,003918439 | -0,163245 | n.s. |
| ENSAPLG00000015420 | RIPK1 | 128 | 64 | 2016 | 98 | 0,006778328 | -0,902149 | n.s. |
| ENSAPLG00000015480 | MAP2K1 | 128 | 64 | 1107 | 21 | 0,002024074 | -1,351313 | n.s. |
| ENSAPLG00000015976 | Mx | 128 | 64 | 2166 | 55 | 0,00261814 | -1,374134 | n.s. |
| ENSAPLG00000016065 | PIK3R5 | 128 | 64 | 2625 | 171 | 0,006606065 | -1,532537 | n.s. |
| ENSAPLG00000016230 | TRAF2 | 128 | 64 | 1602 | 24 | 0,001747631 | -1,058115 | n.s. |
| ENSAPLG00000016235 | AKT2 | 112 | 56 | 951 | 33 | 0,004934553 | -1,015385 | n.s. |
| ENSAPLG00000016314 | AKT1 | 128 | 64 | 1446 | 24 | 0,000879088 | -2,054043 | * |
| ENSAPLG00000016448 | MAPK8 | 128 | 64 | 1290 | 6 | 0,00016614 | -1,743434 | # |

n^hap^ = number reconstructed haplotypes

n^ind^ = number of individuals

Sites = number of nucleotide sites in the protein coding gene

S = number of segregating sites

Pi = Nucleotide diversity

Tajima’s D = Tajima’s D neutrality value

SigD = Significance value for the Tajima’s D (n.s. = not significant, * P < 0.05; ** P < 0.01; *** P < 0.001)

^a^TRIM27L = TRIM27-like

^b^ CCL24 = CCL24orCCL4

#### Table S7 Per gene DNA polymorphism and neutrality statistics in the farm mallards.

| Ensembl ID | GeneID | n^hap^ | n^ind^ | Sites | S | Pi | TajimaD | SigD |
| --- | --- | --- | --- | --- | --- | --- | --- | --- |
| ENSAPLG00000000983 | cathelicidinlike | 24 | 12 | 162 | 0 | 0 | n.a. | n.a. |
| ENSAPLG00000001279 | TLR5 | 32 | 16 | 2571 | 58 | 0,003187695 | -1,600134 | # |
| ENSAPLG00000001297 | AvBD4 | 32 | 16 | 129 | 1 | 0,001812953 | -1,063155 | n.s. |
| ENSAPLG00000001534 | PML | 30 | 15 | 606 | 15 | 0,006536171 | 0,155341 | n.s. |
| ENSAPLG00000001925 | TIRAP | 30 | 15 | 693 | 16 | 0,006342572 | 0,299938 | n.s. |
| ENSAPLG00000002012 | IFNGR1 | 32 | 16 | 1242 | 15 | 0,00302257 | 0,026246 | n.s. |
| ENSAPLG00000002049 | TLR1A | 32 | 16 | 2463 | 50 | 0,004380149 | -0,483309 | n.s. |
| ENSAPLG00000002168 | MAPK13 | 32 | 16 | 1086 | 19 | 0,004221618 | -0,096621 | n.s. |
| ENSAPLG00000002299 | LITAF | 32 | 16 | 447 | 0 | 0 | n.a. | n.a. |
| ENSAPLG00000002603 | MAPK14 | 32 | 16 | 966 | 7 | 0,001548621 | -0,405464 | n.s. |
| ENSAPLG00000002702 | MAP3K7 | 32 | 16 | 1779 | 13 | 0,00142795 | -0,694192 | n.s. |
| ENSAPLG00000002771 | TRIM27L isoform 1^a^ | 32 | 16 | 891 | 41 | 0,008098458 | -1,125704 | n.s. |
| ENSAPLG00000002771 | TRIM27L isoform 2^a^ | 32 | 16 | 1506 | 82 | 0,012103468 | -0,394073 | n.s. |
| ENSAPLG00000003004 | SPP1 | 32 | 16 | 807 | 15 | 0,005084043 | 0,109616 | n.s. |
| ENSAPLG00000003319 | MAVS | 26 | 13 | 1896 | 126 | 0,018958917 | 0,178758 | n.s. |
| ENSAPLG00000003430 | IFN | 32 | 16 | 540 | 5 | 0,001392622 | -1,058232 | n.s. |
| ENSAPLG00000003554 | PIK3CB | 32 | 16 | 3210 | 14 | 0,000851673 | -0,703419 | n.s. |
| ENSAPLG00000003568 | RAC1 | 32 | 16 | 543 | 3 | 0,001125022 | -0,415887 | n.s. |
| ENSAPLG00000004139 | TLR7 | 32 | 16 | 3147 | 18 | 0,000811064 | -1,570922 | n.s. |
| ENSAPLG00000004157 | MAPK12 | 32 | 16 | 978 | 16 | 0,003356092 | -0,582906 | n.s. |
| ENSAPLG00000004376 | IRAK1BP1 | 32 | 16 | 378 | 12 | 0,007568484 | -0,128361 | n.s. |
| ENSAPLG00000004462 | MAPK11 | 32 | 16 | 987 | 12 | 0,00207741 | -1,003943 | n.s. |
| ENSAPLG00000004748 | JAK2 | 32 | 16 | 3423 | 5 | 0,000125456 | -1,755568 | # |
| ENSAPLG00000004772 | PIK3R1 | 32 | 16 | 2172 | 9 | 0,000585058 | -1,322149 | n.s. |
| ENSAPLG00000004845 | LY96 | 32 | 16 | 465 | 10 | 0,004361776 | -0,572029 | n.s. |
| ENSAPLG00000004947 | TRIM25 | 30 | 15 | 1809 | 13 | 0,001719052 | -0,174514 | n.s. |
| ENSAPLG00000005139 | MYD88 b | 32 | 16 | 969 | 34 | 0,008304283 | -0,176037 | n.s. |
| ENSAPLG00000005392 | TRADD | 32 | 16 | 912 | 25 | 0,008057884 | 0,646717 | n.s. |
| ENSAPLG00000005400 | CCL19 | 28 | 14 | 285 | 6 | 0,005222671 | -0,23821 | n.s. |
| ENSAPLG00000005826 | MAP2K6 | 32 | 16 | 1005 | 12 | 0,002852672 | -0,121826 | n.s. |
| ENSAPLG00000005856 | MAPK10 | 32 | 16 | 1173 | 12 | 0,003238182 | 0,884439 | n.s. |
| ENSAPLG00000005913 | CD86 | 32 | 16 | 834 | 9 | 0,001798561 | -1,007121 | n.s. |
| ENSAPLG00000005942 | IL18 | 32 | 16 | 612 | 2 | 0,000388731 | -1,046839 | n.s. |
| ENSAPLG00000006016 | RSAD2 | 32 | 16 | 912 | 8 | 0,001421459 | -1,039824 | n.s. |
| ENSAPLG00000006116 | MAPK1 | 32 | 16 | 966 | 0 | 0 | n.a. | n.a. |
| ENSAPLG00000006147 | IL12A | 32 | 16 | 501 | 15 | 0,007790323 | 0,152228 | n.s. |
| ENSAPLG00000006203 | IRF5 | 16 | 8 | 1119 | 28 | 0,008028001 | 0,266134 | n.s. |
| ENSAPLG00000006379 | IKBKB | 32 | 16 | 2070 | 4 | 0,000149018 | -1,740175 | # |
| ENSAPLG00000006669 | PIK3CG | 32 | 16 | 3321 | 35 | 0,002215253 | -0,554436 | n.s. |
| ENSAPLG00000006823 | PIK3R3 | 26 | 13 | 2169 | 46 | 0,003701103 | -1,271804 | n.s. |
| ENSAPLG00000007148 | MAP2K3 | 32 | 16 | 897 | 22 | 0,007010375 | 0,352841 | n.s. |
| ENSAPLG00000007417 | CD80 | 32 | 16 | 951 | 15 | 0,003573371 | -0,491024 | n.s. |
| ENSAPLG00000007876 | TANK | 32 | 16 | 1266 | 0 | 0 | n.a. | n.a. |
| ENSAPLG00000008173 | TAB2 | 32 | 16 | 2037 | 11 | 0,000744295 | -1,412262 | n.s. |
| ENSAPLG00000008183 | PIK3CD | 32 | 16 | 3144 | 51 | 0,004676727 | 0,594606 | n.s. |
| ENSAPLG00000008445 | TBK1 | 32 | 16 | 2190 | 29 | 0,003498306 | 0,227754 | n.s. |
| ENSAPLG00000008655 | LBP | 32 | 16 | 1080 | 67 | 0,019334304 | 0,952833 | n.s. |
| ENSAPLG00000008976 | TLR3 | 32 | 16 | 2697 | 15 | 0,001263351 | -0,283309 | n.s. |
| ENSAPLG00000009092 | IL12B | 32 | 16 | 963 | 4 | 0,000893964 | -0,336331 | n.s. |
| ENSAPLG00000009156 | TOLLIP | 32 | 16 | 825 | 2 | 0,000750244 | 0,495003 | n.s. |
| ENSAPLG00000009205 | MAP3K8 | 32 | 16 | 1416 | 13 | 0,002007586 | -0,388913 | n.s. |
| ENSAPLG00000009391 | CCL24 isoform 1 ^b^ | 32 | 16 | 267 | 0 | 0 | n.a. | n.a. |
| ENSAPLG00000009391 | CCL24 isoform 2 ^b^ | 32 | 16 | 273 | 2 | 0,000672043 | -1,267102 | n.s. |
| ENSAPLG00000009793 | CCL5 a | 28 | 14 | 273 | 30 | 0,029652887 | 0,059431 | n.s. |
| ENSAPLG00000009838 | CCL5 b | 32 | 16 | 273 | 2 | 0,003655619 | 2,028686 | * |
| ENSAPLG00000009929 | TLR15 | 32 | 16 | 2526 | 46 | 0,003947654 | -0,46629 | n.s. |
| ENSAPLG00000010090 | NLRP12 | 32 | 16 | 2538 | 49 | 0,003131434 | -1,277633 | n.s. |
| ENSAPLG00000010194 | MYD88 a | 32 | 16 | 408 | 6 | 0,003399747 | -0,193738 | n.s. |
| ENSAPLG00000010913 | IFIH1 | 32 | 16 | 3039 | 11 | 0,000618049 | -0,992875 | n.s. |
| ENSAPLG00000011028 | NFKBIA | 32 | 16 | 726 | 9 | 0,001943926 | -1,128711 | n.s. |
| ENSAPLG00000011045 | MAP2K2 | 32 | 16 | 921 | 15 | 0,002567773 | -1,213773 | n.s. |
| ENSAPLG00000011237 | IFNGR2 | 32 | 16 | 999 | 29 | 0,006686122 | -0,258023 | n.s. |
| ENSAPLG00000011322 | IFNAR1 | 32 | 16 | 1671 | 14 | 0,001630036 | -0,71297 | n.s. |
| ENSAPLG00000011364 | IFNAR2 | 32 | 16 | 1524 | 40 | 0,008076422 | 0,658452 | n.s. |
| ENSAPLG00000011397 | TLR2 | 32 | 16 | 2373 | 35 | 0,003203188 | -0,455555 | n.s. |
| ENSAPLG00000011399 | TLR2a | 32 | 16 | 2352 | 39 | 0,004556006 | 0,387522 | n.s. |
| ENSAPLG00000011477 | AKT3 | 32 | 16 | 1290 | 4 | 0,00041104 | -1,176588 | n.s. |
| ENSAPLG00000011503 | CHUK | 32 | 16 | 2100 | 19 | 0,001203143 | -1,591399 | # |
| ENSAPLG00000011572 | AvBD8 | 32 | 16 | 90 | 2 | 0,003853047 | -0,606313 | n.s. |
| ENSAPLG00000011675 | PIK3R2 | 20 | 10 | 2187 | 40 | 0,004664737 | -0,533657 | n.s. |
| ENSAPLG00000011754 | IL6 | 30 | 15 | 465 | 2 | 0,000286738 | -1,507378 | n.s. |
| ENSAPLG00000011919 | AvBD13 | 32 | 16 | 180 | 3 | 0,002441756 | -0,947604 | n.s. |
| ENSAPLG00000011922 | AvBD12 | 32 | 16 | 198 | 13 | 0,011271994 | -1,005576 | n.s. |
| ENSAPLG00000011923 | AvBD11 | 32 | 16 | 315 | 15 | 0,011968766 | 0,040644 | n.s. |
| ENSAPLG00000011932 | AvBD10 | 32 | 16 | 204 | 13 | 0,013707701 | -0,435723 | n.s. |
| ENSAPLG00000011933 | TRAF3 | 32 | 16 | 1704 | 7 | 0,000795093 | -0,641734 | n.s. |
| ENSAPLG00000012022 | TYK2 | 24 | 12 | 2904 | 60 | 0,005407315 | -0,091283 | n.s. |
| ENSAPLG00000012047 | JAK1 | 32 | 16 | 2886 | 57 | 0,00476438 | -0,105802 | n.s. |
| ENSAPLG00000012056 | AvBD9 | 32 | 16 | 192 | 4 | 0,005607359 | 0,211886 | n.s. |
| ENSAPLG00000012239 | AvBD7 | 32 | 16 | 198 | 4 | 0,003614777 | -1,136682 | n.s. |
| ENSAPLG00000012251 | AvBD2 | 32 | 16 | 177 | 4 | 0,007586113 | 0,888191 | n.s. |
| ENSAPLG00000012255 | IRAK4 | 28 | 14 | 1413 | 32 | 0,006345645 | 0,116677 | n.s. |
| ENSAPLG00000012287 | AvBD1 | 32 | 16 | 198 | 1 | 0,002352151 | 1,336786 | n.s. |
| ENSAPLG00000012288 | AvBD16_or_AvBD3a | 32 | 16 | 204 | 2 | 0,004724067 | 0,678826 | n.s. |
| ENSAPLG00000012380 | AZI2 | 32 | 16 | 1173 | 18 | 0,002791299 | -0,909718 | n.s. |
| ENSAPLG00000012383 | CCL4 b | 32 | 16 | 282 | 0 | 0 | n.a. | n.a. |
| ENSAPLG00000012465 | AvBD5 | 32 | 16 | 201 | 6 | 0,003300032 | -1,558396 | n.s. |
| ENSAPLG00000012580 | AvBD14 | 32 | 16 | 186 | 5 | 0,004292404 | -0,95799 | n.s. |
| ENSAPLG00000012625 | TLR4 | 32 | 16 | 2538 | 35 | 0,002713592 | -0,749684 | n.s. |
| ENSAPLG00000012692 | IL8-like b | 32 | 16 | 315 | 1 | 0,00140809 | 1,200428 | n.s. |
| ENSAPLG00000012697 | IL8-like a | 32 | 16 | 312 | 0 | 0 | n.a. | n.a. |
| ENSAPLG00000012768 | CTSK | 32 | 16 | 1008 | 7 | 0,001388089 | -0,567478 | n.s. |
| ENSAPLG00000012773 | CCL4 a | 32 | 16 | 279 | 10 | 0,011865534 | 1,040526 | n.s. |
| ENSAPLG00000012876 | IFNG | 32 | 16 | 495 | 8 | 0,002203486 | -1,349685 | n.s. |
| ENSAPLG00000012924 | PIK3CA isoform 1 | 32 | 16 | 2034 | 11 | 0,000814778 | -1,248256 | n.s. |
| ENSAPLG00000012924 | PIK3CA isoform 2 | 32 | 16 | 3204 | 18 | 0,000780904 | -1,497376 | n.s. |
| ENSAPLG00000013074 | MAP2K4 | 32 | 16 | 1203 | 6 | 0,000933486 | -0,691705 | n.s. |
| ENSAPLG00000013226 | STAT1 b | 32 | 16 | 1743 | 26 | 0,003480512 | -0,212997 | n.s. |
| ENSAPLG00000013262 | STAT1 a | 32 | 16 | 540 | 22 | 0,007011649 | -1,066762 | n.s. |
| ENSAPLG00000013460 | MAPK9 | 32 | 16 | 1287 | 4 | 0,000831829 | 0,196515 | n.s. |
| ENSAPLG00000013488 | TRAF6 | 32 | 16 | 1656 | 15 | 0,002850095 | 0,88832 | n.s. |
| ENSAPLG00000013556 | CASP1 | 32 | 16 | 1149 | 44 | 0,010505278 | 0,383899 | n.s. |
| ENSAPLG00000013558 | CD40 | 24 | 12 | 544 | 9 | 0,003936221 | -0,365546 | n.s. |
| ENSAPLG00000013878 | NFKB1 | 32 | 16 | 2961 | 35 | 0,002916272 | -0,023161 | n.s. |
| ENSAPLG00000014351 | IKBKE | 32 | 16 | 2166 | 80 | 0,008316765 | -0,350089 | n.s. |
| ENSAPLG00000014359 | TICAM1 | 32 | 16 | 2184 | 99 | 0,012350637 | 0,285805 | n.s. |
| ENSAPLG00000014377 | CD14 isoform 2 | 32 | 16 | 792 | 38 | 0,010457397 | -0,44395 | n.s. |
| ENSAPLG00000014377 | CD14 isoform 1 | 32 | 16 | 1281 | 70 | 0,011959847 | -0,44358 | n.s. |
| ENSAPLG00000014394 | TNFRSF6B | 30 | 15 | 843 | 20 | 0,005336715 | -0,52981 | n.s. |
| ENSAPLG00000014631 | TMEM173 | 24 | 12 | 1131 | 49 | 0,011660836 | -0,058243 | n.s. |
| ENSAPLG00000014872 | CASP8 | 32 | 16 | 1443 | 12 | 0,001145687 | -1,433021 | n.s. |
| ENSAPLG00000014996 | IFITM3 | 30 | 15 | 270 | 4 | 0,002085994 | -1,135347 | n.s. |
| ENSAPLG00000015091 | DDX58 | 32 | 16 | 2562 | 5 | 0,000336022 | -0,822881 | n.s. |
| ENSAPLG00000015131 | TAB1 | 32 | 16 | 1689 | 28 | 0,003244428 | -0,851529 | n.s. |
| ENSAPLG00000015244 | CASP10 | 32 | 16 | 1557 | 18 | 0,003133611 | 0,311627 | n.s. |
| ENSAPLG00000015420 | RIPK1 | 32 | 16 | 2016 | 57 | 0,007084453 | 0,033729 | n.s. |
| ENSAPLG00000015480 | MAP2K1 | 32 | 16 | 1107 | 7 | 0,00178483 | 0,397851 | n.s. |
| ENSAPLG00000015976 | Mx | 32 | 16 | 2166 | 16 | 0,002359597 | 0,960381 | n.s. |
| ENSAPLG00000016065 | PIK3R5 | 32 | 16 | 2625 | 77 | 0,006778034 | -0,392044 | n.s. |
| ENSAPLG00000016230 | TRAF2 | 32 | 16 | 1602 | 14 | 0,00225147 | 0,123671 | n.s. |
| ENSAPLG00000016235 | AKT2 | 18 | 9 | 951 | 12 | 0,0039386 | 0,146731 | n.s. |
| ENSAPLG00000016314 | AKT1 | 32 | 16 | 1446 | 8 | 0,001199081 | -0,380609 | n.s. |
| ENSAPLG00000016448 | MAPK8 | 32 | 16 | 1290 | 0 | 0 | n.a. | n.a. |

n^hap^ = number reconstructed haplotypes

n^ind^ = number of individuals

Sites = number of nucleotide sites in the protein coding gene

S = number of segregating sites

Pi = Nucleotide diversity

Tajima’s D = Tajima’s D neutrality value

SigD = Significance value for the Tajima’s D (n.s. = not significant, * P < 0.05; ** P < 0.01; *** P < 0.001)

^a^ TRIM27L= TRIM27-like

#### Table S8 Per gene DNA polymorphism and neutrality statistics in the Pekin ducks.

| Ensembl ID | GeneID | n^hap^ | n^ind^ | Sites | S | Pi | TajimaD | SigD |
| --- | --- | --- | --- | --- | --- | --- | --- | --- |
| ENSAPLG00000000983 | cathelicidinlike | 28 | 14 | 162 | 0 | 0 | n.a. | n.a. |
| ENSAPLG00000001279 | TLR5 | 32 | 16 | 2571 | 1 | 0,000181146 | 1,336786 | n.s. |
| ENSAPLG00000001297 | AvBD4 | 32 | 16 | 129 | 1 | 0,00386034 | 1,535124 | n.s. |
| ENSAPLG00000001534 | PML | 30 | 15 | 606 | 5 | 0,000652479 | -1,873465 | * |
| ENSAPLG00000001925 | TIRAP | 30 | 15 | 693 | 12 | 0,005370619 | 0,746316 | n.s. |
| ENSAPLG00000002012 | IFNGR1 | 32 | 16 | 1242 | 8 | 0,002693042 | 2,046636 | * |
| ENSAPLG00000002049 | TLR1A | 32 | 16 | 2463 | 31 | 0,003130198 | 0,005633 | n.s. |
| ENSAPLG00000002168 | MAPK13 | 32 | 16 | 1086 | 10 | 0,001824912 | -0,630344 | n.s. |
| ENSAPLG00000002299 | LITAF | 32 | 16 | 447 | 0 | 0 | n.a. | n.a. |
| ENSAPLG00000002603 | MAPK14 | 32 | 16 | 966 | 3 | 0,000899536 | 0,384818 | n.s. |
| ENSAPLG00000002702 | MAP3K7 | 32 | 16 | 1779 | 7 | 0,001430216 | 1,349679 | n.s. |
| ENSAPLG00000002771 | TRIM27L isoform 1^a^ | 32 | 16 | 891 | 18 | 0,006358387 | 0,910025 | n.s. |
| ENSAPLG00000002771 | TRIM27L isoform 2^a^ | 32 | 16 | 1506 | 54 | 0,009029741 | 0,052481 | n.s. |
| ENSAPLG00000003004 | SPP1 | 32 | 16 | 807 | 9 | 0,002713155 | -0,369192 | n.s. |
| ENSAPLG00000003319 | MAVS | 24 | 12 | 1896 | 84 | 0,013324601 | 0,476978 | n.s. |
| ENSAPLG00000003430 | IFN | 32 | 16 | 540 | 2 | 0,001642772 | 1,580002 | n.s. |
| ENSAPLG00000003554 | PIK3CB | 32 | 16 | 3210 | 6 | 0,000518164 | 0,327036 | n.s. |
| ENSAPLG00000003568 | RAC1 | 32 | 16 | 543 | 1 | 0,000115101 | -1,142442 | n.s. |
| ENSAPLG00000004139 | TLR7 | 32 | 16 | 3147 | 4 | 0,000508677 | 1,544001 | n.s. |
| ENSAPLG00000004157 | MAPK12 | 32 | 16 | 978 | 4 | 0,001348209 | 0,826708 | n.s. |
| ENSAPLG00000004376 | IRAK1BP1 | 32 | 16 | 378 | 8 | 0,007120456 | 1,062371 | n.s. |
| ENSAPLG00000004462 | MAPK11 | 32 | 16 | 987 | 5 | 0,000725153 | -1,136682 | n.s. |
| ENSAPLG00000004748 | JAK2 | 32 | 16 | 3423 | 0 | 0 | n.a. | n.a. |
| ENSAPLG00000004772 | PIK3R1 | 32 | 16 | 2172 | 1 | 0,00010401 | -0,138355 | n.s. |
| ENSAPLG00000004845 | LY96 | 32 | 16 | 465 | 0 | 0 | n.a. | n.a. |
| ENSAPLG00000004947 | TRIM25 | 30 | 15 | 1809 | 8 | 0,000753992 | -0,987063 | n.s. |
| ENSAPLG00000005139 | MYD88 b | 30 | 15 | 969 | 27 | 0,00685376 | -0,098717 | n.s. |
| ENSAPLG00000005392 | TRADD | 32 | 16 | 912 | 15 | 0,007204566 | 2,54048 | * |
| ENSAPLG00000005400 | CCL19 | 20 | 10 | 285 | 1 | 0,000369004 | -1,164391 | n.s. |
| ENSAPLG00000005826 | MAP2K6 | 32 | 16 | 1005 | 7 | 0,002156556 | 0,718502 | n.s. |
| ENSAPLG00000005856 | MAPK10 | 32 | 16 | 1173 | 8 | 0,001998941 | 0,53986 | n.s. |
| ENSAPLG00000005913 | CD86 | 32 | 16 | 834 | 3 | 0,001655933 | 1,973715 | # |
| ENSAPLG00000005942 | IL18 | 32 | 16 | 612 | 0 | 0 | n.a. | n.a. |
| ENSAPLG00000006016 | RSAD2 | 32 | 16 | 912 | 6 | 0,001262291 | -0,638487 | n.s. |
| ENSAPLG00000006116 | MAPK1 | 32 | 16 | 966 | 0 | 0 | n.a. | n.a. |
| ENSAPLG00000006147 | IL12A | 32 | 16 | 501 | 8 | 0,005858871 | 1,420838 | n.s. |
| ENSAPLG00000006203 | IRF5 | 18 | 9 | 1119 | 33 | 0,008019532 | -0,261195 | n.s. |
| ENSAPLG00000006379 | IKBKB | 32 | 16 | 2070 | 0 | 0 | n.a. | n.a. |
| ENSAPLG00000006669 | PIK3CG | 32 | 16 | 3321 | 19 | 0,002360346 | 2,264016 | * |
| ENSAPLG00000006823 | PIK3R3 | 18 | 9 | 2169 | 4 | 0,00071115 | 0,978868 | n.s. |
| ENSAPLG00000007148 | MAP2K3 | 32 | 16 | 897 | 17 | 0,00750036 | 2,006103 | # |
| ENSAPLG00000007417 | CD80 | 32 | 16 | 951 | 10 | 0,002848924 | 0,277351 | n.s. |
| ENSAPLG00000007876 | TANK | 32 | 16 | 1266 | 0 | 0 | n.a. | n.a. |
| ENSAPLG00000008173 | TAB2 | 32 | 16 | 2037 | 5 | 0,000859107 | 1,099152 | n.s. |
| ENSAPLG00000008183 | PIK3CD | 32 | 16 | 3144 | 41 | 0,003964284 | 0,818187 | n.s. |
| ENSAPLG00000008445 | TBK1 | 32 | 16 | 2190 | 15 | 0,00186515 | 0,321403 | n.s. |
| ENSAPLG00000008655 | LBP | 30 | 15 | 1080 | 51 | 0,018937846 | 2,196736 | * |
| ENSAPLG00000008976 | TLR3 | 32 | 16 | 2697 | 7 | 0,001166918 | 2,358886 | * |
| ENSAPLG00000009092 | IL12B | 32 | 16 | 963 | 1 | 0,00040197 | 0,853336 | n.s. |
| ENSAPLG00000009156 | TOLLIP | 32 | 16 | 825 | 1 | 0,000505865 | 1,039278 | n.s. |
| ENSAPLG00000009205 | MAP3K8 | 32 | 16 | 1416 | 7 | 0,001554811 | 0,775882 | n.s. |
| ENSAPLG00000009391 | CCL24 isoform 1 ^b^ | 32 | 16 | 267 | 0 | 0 | n.a. | n.a. |
| ENSAPLG00000009391 | CCL24 isoform 2 ^b^ | 32 | 16 | 273 | 1 | 0,000228938 | -1,142442 | n.s. |
| ENSAPLG00000009793 | CCL5 a | 32 | 16 | 273 | 17 | 0,010568061 | -1,069383 | n.s. |
| ENSAPLG00000009838 | CCL5 b | 32 | 16 | 273 | 2 | 0,000672043 | -1,267102 | n.s. |
| ENSAPLG00000009929 | TLR15 | 32 | 16 | 2526 | 24 | 0,002729676 | 0,550391 | n.s. |
| ENSAPLG00000010090 | NLRP12 | 32 | 16 | 2538 | 11 | 0,000638679 | -1,290429 | n.s. |
| ENSAPLG00000010194 | MYD88 a | 28 | 14 | 408 | 4 | 0,003734827 | 1,262311 | n.s. |
| ENSAPLG00000010913 | IFIH1 | 32 | 16 | 3039 | 4 | 0,000248282 | -0,607877 | n.s. |
| ENSAPLG00000011028 | NFKBIA | 32 | 16 | 726 | 2 | 0,001083045 | 1,172108 | n.s. |
| ENSAPLG00000011045 | MAP2K2 | 32 | 16 | 921 | 6 | 0,001744685 | 0,220601 | n.s. |
| ENSAPLG00000011237 | IFNGR2 | 32 | 16 | 999 | 14 | 0,005333963 | 1,75493 | # |
| ENSAPLG00000011322 | IFNAR1 | 32 | 16 | 1671 | 9 | 0,001246356 | -0,208499 | n.s. |
| ENSAPLG00000011364 | IFNAR2 | 32 | 16 | 1524 | 36 | 0,008666444 | 1,586439 | n.s. |
| ENSAPLG00000011397 | TLR2 | 32 | 16 | 2373 | 4 | 0,000834319 | 2,507223 | * |
| ENSAPLG00000011399 | TLR2a | 32 | 16 | 2352 | 12 | 0,00137666 | 0,278937 | n.s. |
| ENSAPLG00000011477 | AKT3 | 32 | 16 | 1290 | 1 | 9,38E-05 | -0,782954 | n.s. |
| ENSAPLG00000011503 | CHUK | 32 | 16 | 2100 | 7 | 0,001103201 | 0,964897 | n.s. |
| ENSAPLG00000011572 | AvBD8 | 32 | 16 | 90 | 1 | 0,005712366 | 1,634293 | n.s. |
| ENSAPLG00000011675 | PIK3R2 | 16 | 8 | 2187 | 21 | 0,003040541 | 0,125272 | n.s. |
| ENSAPLG00000011754 | IL6 | 32 | 16 | 465 | 0 | 0 | n.a. | n.a. |
| ENSAPLG00000011919 | AvBD13 | 32 | 16 | 180 | 1 | 0,000347222 | -1,142442 | n.s. |
| ENSAPLG00000011922 | AvBD12 | 32 | 16 | 198 | 4 | 0,002128136 | -1,453258 | n.s. |
| ENSAPLG00000011923 | AvBD11 | 32 | 16 | 315 | 11 | 0,003814644 | -1,777762 | # |
| ENSAPLG00000011932 | AvBD10 | 32 | 16 | 204 | 8 | 0,003557875 | -1,899536 | * |
| ENSAPLG00000011933 | TRAF3 | 32 | 16 | 1704 | 3 | 0,000397547 | -0,209455 | n.s. |
| ENSAPLG00000012022 | TYK2 | 28 | 14 | 2904 | 48 | 0,004518602 | 0,236155 | n.s. |
| ENSAPLG00000012047 | JAK1 | 32 | 16 | 2886 | 36 | 0,00487825 | 2,080736 | * |
| ENSAPLG00000012056 | AvBD9 | 32 | 16 | 192 | 2 | 0,000955561 | -1,267102 | n.s. |
| ENSAPLG00000012239 | AvBD7 | 32 | 16 | 198 | 1 | 0,00251507 | 1,535124 | n.s. |
| ENSAPLG00000012251 | AvBD2 | 32 | 16 | 177 | 4 | 0,004203116 | -0,633495 | n.s. |
| ENSAPLG00000012255 | IRAK4 | 30 | 15 | 1413 | 16 | 0,001992796 | -1,155695 | n.s. |
| ENSAPLG00000012287 | AvBD1 | 32 | 16 | 198 | 1 | 0,000315657 | -1,142442 | n.s. |
| ENSAPLG00000012288 | AvBD16_or_AvBD3a | 32 | 16 | 204 | 2 | 0,000899352 | -1,267102 | n.s. |
| ENSAPLG00000012380 | AZI2 | 32 | 16 | 1173 | 15 | 0,004223043 | 1,09709 | n.s. |
| ENSAPLG00000012383 | CCL4 b | 32 | 16 | 282 | 0 | 0 | n.a. | n.a. |
| ENSAPLG00000012465 | AvBD5 | 32 | 16 | 201 | 1 | 0,000310945 | -1,142442 | n.s. |
| ENSAPLG00000012580 | AvBD14 | 32 | 16 | 186 | 2 | 0,005278789 | 1,963423 | # |
| ENSAPLG00000012625 | TLR4 | 32 | 16 | 2538 | 25 | 0,002673079 | 0,32677 | n.s. |
| ENSAPLG00000012692 | IL8-like b | 32 | 16 | 315 | 1 | 0,000384025 | -0,782954 | n.s. |
| ENSAPLG00000012697 | IL8-like a | 32 | 16 | 312 | 0 | 0 | n.a. | n.a. |
| ENSAPLG00000012768 | CTSK | 30 | 15 | 1008 | 4 | 0,000387703 | -1,573794 | n.s. |
| ENSAPLG00000012773 | CCL4 a | 32 | 16 | 279 | 6 | 0,011063418 | 3,010735 | ** |
| ENSAPLG00000012876 | IFNG | 32 | 16 | 495 | 0 | 0 | n.a. | n.a. |
| ENSAPLG00000012924 | PIK3CA isoform 1 | 32 | 16 | 2034 | 2 | 0,000267628 | 0,193161 | n.s. |
| ENSAPLG00000012924 | PIK3CA isoform 2 | 32 | 16 | 3204 | 3 | 0,000254848 | 0,222175 | n.s. |
| ENSAPLG00000013074 | MAP2K4 | 32 | 16 | 1203 | 4 | 0,000700534 | -0,382442 | n.s. |
| ENSAPLG00000013226 | STAT1 b | 32 | 16 | 1743 | 10 | 0,000957748 | -1,023341 | n.s. |
| ENSAPLG00000013262 | STAT1 a | 32 | 16 | 540 | 5 | 0,001120072 | -1,376391 | n.s. |
| ENSAPLG00000013460 | MAPK9 | 32 | 16 | 1287 | 3 | 0,000971251 | 1,567107 | n.s. |
| ENSAPLG00000013488 | TRAF6 | 32 | 16 | 1656 | 12 | 0,001619234 | -0,322208 | n.s. |
| ENSAPLG00000013556 | CASP1 | 30 | 15 | 1149 | 11 | 0,002454908 | 0,051083 | n.s. |
| ENSAPLG00000013558 | CD40 | 20 | 10 | 544 | 6 | 0,003463622 | 0,363666 | n.s. |
| ENSAPLG00000013878 | NFKB1 | 32 | 16 | 2961 | 11 | 0,001342724 | 1,446131 | n.s. |
| ENSAPLG00000014351 | IKBKE | 32 | 16 | 2166 | 42 | 0,005684441 | 0,659864 | n.s. |
| ENSAPLG00000014359 | TICAM1 | 32 | 16 | 2184 | 46 | 0,005638515 | 0,202863 | n.s. |
| ENSAPLG00000014377 | CD14 isoform 2 | 30 | 15 | 792 | 16 | 0,007050389 | 1,299356 | n.s. |
| ENSAPLG00000014377 | CD14 isoform 1 | 30 | 15 | 1281 | 31 | 0,00835913 | 1,334543 | n.s. |
| ENSAPLG00000014394 | TNFRSF6B | 28 | 14 | 843 | 8 | 0,003558719 | 1,418186 | n.s. |
| ENSAPLG00000014631 | TMEM173 | 28 | 14 | 1131 | 31 | 0,009651056 | 1,20359 | n.s. |
| ENSAPLG00000014872 | CASP8 | 32 | 16 | 1443 | 2 | 0,000406579 | 0,364476 | n.s. |
| ENSAPLG00000014996 | IFITM3 | 30 | 15 | 270 | 0 | 0 | n.a. | n.a. |
| ENSAPLG00000015091 | DDX58 | 32 | 16 | 2562 | 0 | 0 | n.a. | n.a. |
| ENSAPLG00000015131 | TAB1 | 32 | 16 | 1689 | 10 | 0,000920329 | -1,167863 | n.s. |
| ENSAPLG00000015244 | CASP10 | 32 | 16 | 1557 | 2 | 0,000349618 | 0,193161 | n.s. |
| ENSAPLG00000015420 | RIPK1 | 32 | 16 | 2016 | 37 | 0,005291339 | 0,583995 | n.s. |
| ENSAPLG00000015480 | MAP2K1 | 32 | 16 | 1107 | 1 | 0,000109275 | -0,782954 | n.s. |
| ENSAPLG00000015976 | Mx | 32 | 16 | 2166 | 8 | 0,001197018 | 0,913516 | n.s. |
| ENSAPLG00000016065 | PIK3R5 | 32 | 16 | 2625 | 43 | 0,004097542 | -0,056843 | n.s. |
| ENSAPLG00000016230 | TRAF2 | 32 | 16 | 1602 | 9 | 0,002043816 | 1,424671 | n.s. |
| ENSAPLG00000016235 | AKT2 | 24 | 12 | 951 | 9 | 0,002682969 | 0,082847 | n.s. |
| ENSAPLG00000016314 | AKT1 | 32 | 16 | 1446 | 5 | 0,001001093 | 0,445399 | n.s. |
| ENSAPLG00000016448 | MAPK8 | 32 | 16 | 1290 | 0 | 0 | n.a. | n.a. |

n^hap^ = number reconstructed haplotypes

n^ind^ = number of individuals

Sites = number of nucleotide sites in the protein coding gene

S = number of segregating sites

Pi = Nucleotide diversity

Tajima’s D = Tajima’s D neutrality value

SigD = Significance value for the Tajima’s D (n.s. = not significant, * P < 0.05; ** P < 0.01; *** P < 0.001)

^a^TRIM27L = TRIM27-like

^b^CCL24 = CCL24orCCL4

#### Table S9 Genetic diversity, population divergence and evidence of natural selection in waterfowl.

| Gene name | Ensembl ID | Pi | AA diversity | *F*_ST_ | *F*_ST_ non-syn | TajimaD | dNdS ratio |
| --- | --- | --- | --- | --- | --- | --- | --- |
| AKT1 | ENSAPLG00000016314 | 0,001 | 0 | 0,034 | 0 | **-2,054** | NA |
| AKT2 | ENSAPLG00000016235 | 0,005 | 0,005 | 0,072 | 0,041 | -1,015 | 0,038 |
| AKT3 | ENSAPLG00000011477 | 0,001 | 0 | 0,041 | 0,022 | -1,203 | 0,114 |
| AvBD1 | ENSAPLG00000012287 | 0,002 | 0,005 | 0,011 | 0,011 | -1,429 | **1,231** |
| AvBD10 | ENSAPLG00000011932 | **0,017** | 0,006 | 0,051 | 0,041 | 0,567 | 0,097 |
| AvBD11 | ENSAPLG00000011923 | **0,017** | 0,013 | 0,026 | 0,014 | -0,803 | NA |
| AvBD12 | ENSAPLG00000011922 | **0,019** | **0,017** | 0,034 | 0,073 | -0,09 | 0,184 |
| AvBD13 | ENSAPLG00000011919 | 0,004 | 0,005 | 0,043 | 0,084 | **-1,965** | 0,374 |
| AvBD14 | ENSAPLG00000012580 | 0,006 | 0,01 | 0,023 | 0,036 | -0,441 | 0,2 |
| AvBD16_or_AvBD3a | ENSAPLG00000012288 | 0,006 | 0,015 | 0,063 | 0,076 | **-2,075** | NA |
| AvBD2 | ENSAPLG00000012251 | 0,009 | 0,007 | 0,046 | 0,054 | 0,103 | 0,386 |
| AvBD4 | ENSAPLG00000001297 | 0,006 | 0,013 | **0,125** | **0,125** | -1,069 | **6,1** |
| AvBD5 | ENSAPLG00000012465 | 0,006 | 0,004 | 0,017 | 0,066 | -1,288 | 0,122 |
| AvBD7 | ENSAPLG00000012239 | 0,006 | 0,004 | 0,019 | 0,054 | -0,986 | 0,546 |
| AvBD8 | ENSAPLG00000011572 | 0,009 | 0,015 | 0,057 | 0,063 | -1,186 | 0,337 |
| AvBD9 | ENSAPLG00000012056 | 0,013 | **0,017** | 0,001 | -0,021 | 1,118 | **1,47** |
| AZI2 | ENSAPLG00000012380 | 0,003 | 0,003 | 0,09 | 0,063 | -1,757 | NA |
| CASP1 | ENSAPLG00000013556 | 0,012 | **0,019** | 0,081 | 0,093 | -0,212 | 0,285 |
| CASP10 | ENSAPLG00000015244 | 0,004 | 0,007 | 0,017 | 0,018 | -0,163 | 0,399 |
| CASP8 | ENSAPLG00000014872 | 0,001 | 0,002 | 0,036 | 0,063 | -1,57 | 0,215 |
| cathelicidinlike | ENSAPLG00000000983 | 0 | 0 | 0 | n.a. | -1,058 | **1,163** |
| CCL19 | ENSAPLG00000005400 | 0,008 | 0,001 | 0,088 | n.a. | 0,342 | 0,086 |
| CCL24 isoform 1 ^b^ | ENSAPLG00000009391 | 0,001 | 0 | 0,083 | 0 | -1,34 | 0,61 |
| CCL24 isoform 2 ^b^ | ENSAPLG00000009391 | 0,002 | 0,004 | 0,051 | 0,029 | -0,744 | 0,324 |
| CCL4 a | ENSAPLG00000012773 | **0,013** | **0,032** | 0,018 | 0,017 | -0,479 | 0,156 |
| CCL4 b | ENSAPLG00000012383 | 0 | 0 | **0,097** | # | -0,731 | NA |
| CCL5 a | ENSAPLG00000009793 | **0,028** | **0,056** | 0,044 | 0,035 | -0,374 | NA |
| CCL5 b | ENSAPLG00000009838 | 0,004 | 0,005 | **0,112** | **0,152** | 0,217 | 0,563 |
| CD14 isoform 1 | ENSAPLG00000014377 | 0,01 | **0,016** | 0,045 | 0,036 | -1,384 | NA |
| CD14 isoform 2 | ENSAPLG00000014377 | **0,013** | 0,014 | 0,027 | 0,021 | -1,224 | NA |
| CD40 | ENSAPLG00000013558 | 0,004 | 0,003 | 0,059 | 0,034 | -1,599 | 0,316 |
| CD80 | ENSAPLG00000007417 | 0,004 | 0,004 | 0,041 | 0,037 | -1,488 | 0,309 |
| CD86 | ENSAPLG00000005913 | 0,002 | 0,002 | 0,034 | 0,038 | **-1,843** | 0,46 |
| CHUK | ENSAPLG00000011503 | 0,002 | 0 | 0,071 | **0,155** | **-1,78** | 0,056 |
| CTSK | ENSAPLG00000012768 | 0,001 | 0,001 | 0,051 | 0,005 | **-2,167** | 0,051 |
| DDX58 (RIG-I) | ENSAPLG00000015091 | 0,001 | 0 | 0,082 | 0,11 | -1,563 | 0,332 |
| FOS | ENSAPLG00000011284 | NA | NA | NA | NA | NA | 0,05 |
| IFIH1 (MDA5) | ENSAPLG00000010913 | 0,001 | 0,001 | 0,079 | **0,134** | **-2,063** | NA |
| IFITM3 | ENSAPLG00000014996 | 0,003 | 0,006 | **0,098** | **0,121** | -1,293 | 0,553 |
| IFN | ENSAPLG00000003430 | 0,001 | 0,001 | **0,097** | **0,149** | -1,539 | 0,639 |
| IFNAR1 | ENSAPLG00000011322 | 0,001 | 0,002 | 0,07 | 0,086 | **-2,206** | NA |
| IFNAR2 | ENSAPLG00000011364 | 0,01 | **0,024** | 0,038 | 0,038 | 0,666 | **1,463** |
| IFNG | ENSAPLG00000012876 | 0,002 | 0 | 0,03 | -0,008 | -1,199 | 0,32 |
| IFNGR1 | ENSAPLG00000002012 | 0,003 | 0,004 | 0,051 | 0,039 | -1,402 | 0,809 |
| IFNGR2 | ENSAPLG00000011237 | 0,007 | 0,008 | 0,081 | 0,043 | -1,204 | NA |
| IKBKB | ENSAPLG00000006379 | 0 | 0 | 0 | 0 | **-1,919** | 0,049 |
| IKBKE | ENSAPLG00000014351 | 0,01 | 0,006 | **0,091** | 0,088 | -1,201 | NA |
| IL12A | ENSAPLG00000006147 | 0,008 | 0,01 | 0,026 | 0,011 | -1,129 | 0,178 |
| IL12B | ENSAPLG00000009092 | 0,001 | 0,001 | 0,036 | 0,051 | **-1,79** | 0,342 |
| IL18 | ENSAPLG00000005942 | 0 | 0 | 0,032 | 0,048 | -1,663 | 0,223 |
| IL6 | ENSAPLG00000011754 | 0,001 | 0 | 0,013 | 0 | **-2,013** | 0,062 |
| IL8-like a | ENSAPLG00000012697 | 0,001 | 0,001 | 0,003 | 0,006 | **-2,05** | 0,173 |
| IL8-like b | ENSAPLG00000012692 | 0 | 0 | 0 | n.a. | **-1,889** | 0,687 |
| IRAK1BP1 | ENSAPLG00000004376 | 0,007 | 0,007 | 0 | -0,009 | -0,542 | NA |
| IRAK4 | ENSAPLG00000012255 | 0,006 | 0,007 | 0,059 | 0,021 | -1,307 | NA |
| IRF5 | ENSAPLG00000006203 | 0,01 | 0,014 | 0,066 | 0,067 | -0,879 | NA |
| IRF7 | ENSAPLG00000012752 | NA | NA | NA | NA | NA | 0,136 |
| JAK1 | ENSAPLG00000012047 | 0,005 | 0 | 0,038 | 0,046 | -1,006 | 0,039 |
| JAK2 | ENSAPLG00000004748 | 0 | 0 | **0,185** | 0,013 | -1,618 | 0,089 |
| JUN | ENSAPLG00000005928 | NA | NA | NA | NA | NA | 0,014 |
| LBP | ENSAPLG00000008655 | **0,02** | **0,036** | 0,006 | -0,003 | -0,096 | 0,544 |
| LITAF | ENSAPLG00000002299 | 0 | 0 | 0,032 | # | -0,909 | 0,175 |
| LY96 | ENSAPLG00000004845 | 0,004 | 0,003 | 0,022 | 0,03 | -0,984 | 0,735 |
| MAP2K1 | ENSAPLG00000015480 | 0,002 | 0 | 0,036 | 0 | -1,351 | 0 |
| MAP2K2 | ENSAPLG00000011045 | 0,002 | 0,001 | 0,038 | 0,058 | **-2,054** | 0,025 |
| MAP2K3 | ENSAPLG00000007148 | 0,008 | 0 | 0,037 | 0 | -1,161 | 0,004 |
| MAP2K4 | ENSAPLG00000013074 | 0 | 0 | 0,079 | **0,197** | -1,4 | NA |
| MAP2K6 | ENSAPLG00000005826 | 0,003 | 0,001 | 0,039 | 0,004 | -1,623 | 0,009 |
| MAP3K7 | ENSAPLG00000002702 | 0,001 | 0 | 0,06 | 0 | -1,126 | NA |
| MAP3K8 | ENSAPLG00000009205 | 0,003 | 0 | 0,022 | 0,039 | -1,173 | 0,074 |
| MAPK1 | ENSAPLG00000006116 | 0 | 0 | 0 | # | -1,573 | 0 |
| MAPK10 | ENSAPLG00000005856 | 0,003 | 0 | 0,071 | n.a. | -0,505 | 0 |
| MAPK11 | ENSAPLG00000004462 | 0,002 | 0 | 0,069 | 0,027 | **-2** | 0 |
| MAPK12 | ENSAPLG00000004157 | 0,003 | 0,001 | 0,049 | 0,087 | -0,88 | 0,036 |
| MAPK13 | ENSAPLG00000002168 | 0,005 | 0,002 | 0,036 | 0,046 | -1,119 | 0,067 |
| MAPK14 | ENSAPLG00000002603 | 0,002 | 0 | 0 | 0 | -1,506 | 0 |
| MAPK8 | ENSAPLG00000016448 | 0 | 0 | 0,004 | n.a. | -1,743 | 0 |
| MAPK9 | ENSAPLG00000013460 | 0,001 | 0,001 | 0,015 | 0,02 | -1,629 | 0,012 |
| MAVS | ENSAPLG00000003319 | **0,019** | **0,03** | 0,074 | 0,067 | -0,816 | 0,331 |
| Mx | ENSAPLG00000015976 | 0,003 | 0,004 | **0,111** | 0,087 | -1,374 | 0,655 |
| MYD88 a | ENSAPLG00000010194 | 0,003 | 0 | 0,083 | # | -0,691 | 0 |
| MYD88 b | ENSAPLG00000005139 | 0,01 | 0,013 | 0,053 | 0,056 | -0,32 | NA |
| NFKB1 | ENSAPLG00000013878 | 0,004 | 0,001 | 0,062 | 0,06 | -1,18 | 0,112 |
| NFKBIA | ENSAPLG00000011028 | 0,002 | 0,001 | 0,04 | 0,086 | **-1,939** | 0 |
| NLRP12 | ENSAPLG00000010090 | 0,005 | 0,007 | 0,026 | 0,027 | -1,744 | 0,376 |
| PIK3CA isoform 1 | ENSAPLG00000012924 | 0,001 | 0 | 0,05 | 0 | **-1,88** | 0,09 |
| PIK3CA isoform 2 | ENSAPLG00000012924 | 0,001 | 0 | 0,047 | # | **-1,916** | NA |
| PIK3CB | ENSAPLG00000003554 | 0,001 | 0 | 0,049 | 0,027 | **-1,829** | 0,061 |
| PIK3CD | ENSAPLG00000008183 | 0,005 | 0,001 | 0,033 | 0,015 | -0,59 | 0,037 |
| PIK3CG | ENSAPLG00000006669 | 0,003 | 0 | 0,053 | 0,036 | -1,27 | 0,006 |
| PIK3R1 | ENSAPLG00000004772 | 0,001 | 0,001 | 0,07 | 0,092 | -1,581 | NA |
| PIK3R2 | ENSAPLG00000011675 | 0,006 | 0,002 | 0,062 | 0,049 | -1,292 | 0,039 |
| PIK3R3 | ENSAPLG00000006823 | 0,004 | 0,005 | 0,025 | 0,021 | -1,699 | NA |
| PIK3R5 | ENSAPLG00000016065 | 0,007 | 0,004 | 0,054 | 0,026 | -1,533 | 0,108 |
| PML | ENSAPLG00000001534 | 0,009 | 0,007 | 0,044 | 0,005 | -1,149 | 0,179 |
| RAC1 | ENSAPLG00000003568 | 0,001 | 0 | 0,06 | # | -1,447 | 0 |
| RIPK1 | ENSAPLG00000015420 | 0,007 | 0,006 | 0,089 | **0,129** | -0,902 | 0,247 |
| RSAD2 | ENSAPLG00000006016 | 0,002 | 0 | 0,029 | 0 | -1,335 | 0,12 |
| SOCS3 | ENSAPLG00000012088 | NA | NA | NA | NA | NA | 0,011 |
| SPP1 | ENSAPLG00000003004 | 0,007 | 0,008 | 0,061 | 0,028 | -1,164 | 0,257 |
| STAT1 a | ENSAPLG00000013262 | 0,01 | 0 | 0,062 | 0 | -0,591 | 0,016 |
| STAT1 b | ENSAPLG00000013226 | 0,004 | 0 | 0,088 | -0,007 | -1,091 | 0,061 |
| TAB1 | ENSAPLG00000015131 | 0,003 | 0 | 0,046 | **0,111** | **-1,904** | 0,014 |
| TAB2 | ENSAPLG00000008173 | 0,001 | 0,001 | 0,001 | -0,009 | **-1,867** | 0,192 |
| TANK | ENSAPLG00000007876 | 0 | 0 | 0,002 | -0,005 | **-2,068** | 0,22 |
| TBK1 | ENSAPLG00000008445 | 0,003 | 0 | 0,062 | 0,017 | -0,724 | 0,051 |
| TICAM1 | ENSAPLG00000014359 | **0,013** | **0,022** | 0,025 | 0,028 | -0,553 | 0,614 |
| TIRAP | ENSAPLG00000001925 | 0,008 | 0,014 | 0,061 | 0,05 | -1,426 | 0,244 |
| TLR15 | ENSAPLG00000009929 | 0,004 | 0,004 | 0,087 | 0,084 | -1,165 | 0,413 |
| TLR1A | ENSAPLG00000002049 | 0,005 | 0,009 | 0,01 | 0,014 | -0,73 | 0,392 |
| TLR2 | ENSAPLG00000011397 | 0,004 | 0,004 | 0,063 | 0,084 | -1,366 | 0,543 |
| TLR21 | ENSAPLG00000014726 | NA | NA | NA | NA | NA | 0,142 |
| TLR2a | ENSAPLG00000011399 | 0,004 | 0,004 | 0,087 | 0,075 | -1,272 | 0,34 |
| TLR3 | ENSAPLG00000008976 | 0,002 | 0,002 | 0,033 | 0,012 | **-2,124** | 0,334 |
| TLR4 | ENSAPLG00000012625 | 0,003 | 0,003 | 0,061 | 0,069 | -1,762 | 0,397 |
| TLR5 | ENSAPLG00000001279 | 0,005 | 0,011 | **0,104** | 0,097 | -0,961 | 0,953 |
| TLR7 | ENSAPLG00000004139 | 0,001 | 0,001 | 0,042 | 0,045 | **-1,897** | 0,235 |
| TMEM173 | ENSAPLG00000014631 | 0,012 | 0,011 | 0,051 | 0,043 | -0,977 | 0,145 |
| TNFRSF6B | ENSAPLG00000014394 | 0,005 | 0,003 | 0,065 | 0,03 | -1,192 | 0,072 |
| TOLLIP | ENSAPLG00000009156 | 0,001 | 0,002 | 0,037 | 0,021 | -1,434 | 0,033 |
| TRADD | ENSAPLG00000005392 | 0,008 | 0,004 | 0,02 | 0,024 | -0,718 | 0,116 |
| TRAF2 | ENSAPLG00000016230 | 0,002 | 0 | 0,063 | 0 | -1,058 | 0,137 |
| TRAF3 | ENSAPLG00000011933 | 0,001 | 0 | **0,153** | **0,264** | -1,506 | 0,017 |
| TRAF6 | ENSAPLG00000013488 | 0,002 | 0,001 | 0,065 | -0,017 | -1,182 | 0,067 |
| TRIM25 | ENSAPLG00000004947 | 0,002 | 0,003 | 0,054 | 0,072 | -1,396 | 0,362 |
| TRIM27L isoform 1^a^ | ENSAPLG00000002771 | 0,009 | 0,014 | 0,084 | 0,056 | -1,479 | NA |
| TRIM2L isoform 2^a^ | ENSAPLG00000002771 | **0,013** | 0,007 | 0,053 | 0,055 | -1,033 | NA |
| TYK2 | ENSAPLG00000012022 | 0,006 | 0,002 | 0,07 | 0,108 | -1,493 | NA |

Pi = Nucleotide diversity π, calculated for the protein coding region per gene in the wild mallards (n=64), for more details see Table S6.

AA div = Amino acid diversity, calculated as the number of amino acid differences per site from averaging over all sequence pairs in the wild mallards (n=64).

Fst = Average genetic differentiation among all wild mallard populations (Canadian, Greenlandic, Spanish, and Swedish mallards) for each gene.

Fst non-syn = Average genetic differentiation among all wild mallard populations (Canadian, Greenlandic, Spanish, and Swedish mallards) for each gene when including non-synonymous nucleotides only. n.a., not available. # = not included in analysis as these genes did not have any non-synonymous changes in the wild mallards.

Tajima's D for all immune genes tested in our study in wild mallards (n=64). Genes of which the Tajima's D-value was statistically significant are shown in bold (p<0.05, for more detail see Table S6).

Average dN/dS ratio for each immune gene as calculated in PAML, based on 26 species of ducks and geese (for more details see Table S15).

Significant genes or the top ten genes are shown in bold.

^a^ TRIM27L = TRIM27-like

^b^ CCL24 = CCL24orCCL4

#### Table S10 Amino acid diversity per gene and mallard population

| Ensembl ID | Gene | Canada | Greenland | Spain | Sweden | Farm | Pekin |
| --- | --- | --- | --- | --- | --- | --- | --- |
| ENSAPLG00000000983 | cathelicidinlike | 0,00000 | 0,00000 | 0,00000 | 0,00000 | 0,00000 | 0,00000 |
| ENSAPLG00000001279 | TLR5 | 0,01419 | 0,01415 | 0,00811 | 0,00487 | 0,00626 | 0,00000 |
| ENSAPLG00000001297 | AvBB4 | 0,00924 | 0,01838 | 0,00563 | 0,01285 | 0,00281 | 0,01158 |
| ENSAPLG00000001534 | PML | 0,00907 | 0,00661 | 0,00461 | 0,00566 | 0,00397 | 0,00097 |
| ENSAPLG00000001925 | TIRAP | 0,01319 | 0,01302 | 0,01270 | 0,01241 | 0,01053 | 0,00815 |
| ENSAPLG00000002012 | IFNGR1 | 0,00430 | 0,00426 | 0,00412 | 0,00398 | 0,00412 | 0,00368 |
| ENSAPLG00000002049 | TLR1A | 0,00996 | 0,00937 | 0,00751 | 0,00941 | 0,00800 | 0,00507 |
| ENSAPLG00000002168 | MAPK13 | 0,00159 | 0,00097 | 0,00177 | 0,00159 | 0,00142 | 0,00048 |
| ENSAPLG00000002299 | LITAF | 0,00000 | 0,00000 | 0,00000 | 0,00000 | 0,00000 | 0,00000 |
| ENSAPLG00000002603 | MAPK14 | 0,00000 | 0,00000 | 0,00019 | 0,00019 | 0,00000 | 0,00000 |
| ENSAPLG00000002702 | MAP3K7 | 0,00011 | 0,00000 | 0,00000 | 0,00000 | 0,00021 | 0,00000 |
| ENSAPLG00000002771 | TRIM27L isoform 1^a^ | 0,01381 | 0,01121 | 0,01451 | 0,01288 | 0,01257 | 0,01022 |
| ENSAPLG00000002771 | TRIM27L isoform 1^a^ | 0,00689 | 0,00398 | 0,00699 | 0,00874 | 0,00551 | 0,00435 |
| ENSAPLG00000003004 | SPP1 | 0,00711 | 0,00633 | 0,00911 | 0,00884 | 0,00579 | 0,00246 |
| ENSAPLG00000003319 | MAVS | 0,02835 | 0,02138 | 0,03202 | 0,03167 | 0,02813 | 0,02005 |
| ENSAPLG00000003430 | IFN | 0,00035 | 0,00231 | 0,00000 | 0,00000 | 0,00160 | 0,00246 |
| ENSAPLG00000003554 | PIK3CB | 0,00057 | 0,00049 | 0,00012 | 0,00022 | 0,00038 | 0,00000 |
| ENSAPLG00000003568 | RAC1 | 0,00000 | 0,00000 | 0,00000 | 0,00000 | 0,00000 | 0,00000 |
| ENSAPLG00000004139 | TLR7 | 0,00196 | 0,00058 | 0,00050 | 0,00047 | 0,00018 | 0,00000 |
| ENSAPLG00000004157 | MAPK12 | 0,00089 | 0,00000 | 0,00054 | 0,00128 | 0,00148 | 0,00084 |
| ENSAPLG00000004376 | IRAK1BP1 | 0,00602 | 0,00721 | 0,00729 | 0,00677 | 0,00581 | 0,00252 |
| ENSAPLG00000004462 | MAPK11 | 0,00053 | 0,00000 | 0,00019 | 0,00019 | 0,00083 | 0,00038 |
| ENSAPLG00000004748 | JAK2 | 0,00011 | 0,00000 | 0,00000 | 0,00016 | 0,00005 | 0,00000 |
| ENSAPLG00000004772 | PIK3R1 | 0,00066 | 0,00058 | 0,00052 | 0,00025 | 0,00042 | 0,00000 |
| ENSAPLG00000004845 | LY96 | 0,00294 | 0,00081 | 0,00549 | 0,00196 | 0,00382 | 0,00000 |
| ENSAPLG00000004947 | TRIM25 | 0,00326 | 0,00207 | 0,00284 | 0,00265 | 0,00246 | 0,00165 |
| ENSAPLG00000005139 | MYD88 b | 0,01275 | 0,00850 | 0,01323 | 0,01401 | 0,01181 | 0,00861 |
| ENSAPLG00000005392 | TRADD | 0,00412 | 0,00424 | 0,00415 | 0,00509 | 0,00220 | 0,00501 |
| ENSAPLG00000005400 | CCL19 | 0,00293 | 0,00000 | 0,00000 | 0,00185 | 0,00000 | 0,00000 |
| ENSAPLG00000005826 | MAP2K6 | 0,00091 | 0,00072 | 0,00132 | 0,00090 | 0,00019 | 0,00154 |
| ENSAPLG00000005856 | MAPK10 | 0,00016 | 0,00000 | 0,00000 | 0,00000 | 0,00000 | 0,00000 |
| ENSAPLG00000005913 | CD86 | 0,00304 | 0,00108 | 0,00172 | 0,00343 | 0,00247 | 0,00185 |
| ENSAPLG00000005942 | IL18 | 0,00086 | 0,00000 | 0,00000 | 0,00031 | 0,00086 | 0,00000 |
| ENSAPLG00000006016 | RSAD2 | 0,00021 | 0,00000 | 0,00000 | 0,00000 | 0,00075 | 0,00000 |
| ENSAPLG00000006116 | MAPK1 | 0,00000 | 0,00000 | 0,00000 | 0,00000 | 0,00000 | 0,00000 |
| ENSAPLG00000006147 | IL12A | 0,01130 | 0,00619 | 0,01096 | 0,01164 | 0,00888 | 0,00503 |
| ENSAPLG00000006203 | IRF5 | 0,00922 | 0,01526 | 0,01726 | 0,01406 | 0,01501 | 0,01379 |
| ENSAPLG00000006379 | IKBKB | 0,00009 | 0,00000 | 0,00000 | 0,00009 | 0,00000 | 0,00000 |
| ENSAPLG00000006669 | PIK3CG | 0,00023 | 0,00025 | 0,00017 | 0,00017 | 0,00045 | 0,00035 |
| ENSAPLG00000006823 | PIK3R3 | 0,00644 | 0,00439 | 0,00591 | 0,00480 | 0,00470 | 0,00000 |
| ENSAPLG00000007148 | MAP2K3 | 0,00042 | 0,00021 | 0,00000 | 0,00042 | 0,00059 | 0,00191 |
| ENSAPLG00000007417 | CD80 | 0,00406 | 0,00365 | 0,00481 | 0,00394 | 0,00260 | 0,00201 |
| ENSAPLG00000007876 | TANK | 0,00030 | 0,00000 | 0,00000 | 0,00030 | 0,00000 | 0,00000 |
| ENSAPLG00000008173 | TAB2 | 0,00138 | 0,00090 | 0,00160 | 0,00155 | 0,00151 | 0,00173 |
| ENSAPLG00000008183 | PIK3CD | 0,00069 | 0,00080 | 0,00041 | 0,00045 | 0,00045 | 0,00110 |
| ENSAPLG00000008445 | TBK1 | 0,00026 | 0,00000 | 0,00048 | 0,00034 | 0,00074 | 0,00009 |
| ENSAPLG00000008655 | LBP | 0,03588 | 0,03679 | 0,03754 | 0,03574 | 0,03631 | 0,03560 |
| ENSAPLG00000008976 | TLR3 | 0,00215 | 0,00066 | 0,00198 | 0,00174 | 0,00117 | 0,00114 |
| ENSAPLG00000009092 | IL12B | 0,00149 | 0,00000 | 0,00160 | 0,00160 | 0,00176 | 0,00000 |
| ENSAPLG00000009156 | TOLLIP | 0,00193 | 0,00082 | 0,00187 | 0,00170 | 0,00182 | 0,00152 |
| ENSAPLG00000009205 | MAP3K8 | 0,00013 | 0,00037 | 0,00013 | 0,00000 | 0,00000 | 0,00000 |
| ENSAPLG00000009391 | CCL24 isoform 1 ^b^ | 0,00000 | 0,00000 | 0,00000 | 0,00140 | 0,00000 | 0,00000 |
| ENSAPLG00000009391 | CCL24 isoform 1 ^b^ | 0,00461 | 0,00652 | 0,00419 | 0,00204 | 0,00134 | 0,00069 |
| ENSAPLG00000009793 | CCL5 a | 0,05001 | 0,05016 | 0,06166 | 0,05849 | 0,06405 | 0,02361 |
| ENSAPLG00000009838 | CCL5 b | 0,00623 | 0,00069 | 0,00759 | 0,00385 | 0,00571 | 0,00069 |
| ENSAPLG00000009929 | TLR15 | 0,00400 | 0,00404 | 0,00366 | 0,00359 | 0,00328 | 0,00270 |
| ENSAPLG00000010090 | NLRP12 | 0,00917 | 0,00469 | 0,00718 | 0,00719 | 0,00437 | 0,00083 |
| ENSAPLG00000010194 | MYD88 a | 0,00000 | 0,00000 | 0,00000 | 0,00000 | 0,00000 | 0,00000 |
| ENSAPLG00000010913 | IFIH1 | 0,00121 | 0,00006 | 0,00087 | 0,00099 | 0,00078 | 0,00037 |
| ENSAPLG00000011028 | NFKBIA | 0,00000 | 0,00131 | 0,00026 | 0,00052 | 0,00026 | 0,00000 |
| ENSAPLG00000011045 | MAP2K2 | 0,00182 | 0,00060 | 0,00057 | 0,00060 | 0,00202 | 0,00000 |
| ENSAPLG00000011237 | IFNGR2 | 0,00843 | 0,00819 | 0,00712 | 0,00803 | 0,00912 | 0,00913 |
| ENSAPLG00000011322 | IFNAR1 | 0,00448 | 0,00011 | 0,00252 | 0,00153 | 0,00413 | 0,00293 |
| ENSAPLG00000011364 | IFNAR2 | 0,02426 | 0,02342 | 0,02209 | 0,02226 | 0,01940 | 0,02090 |
| ENSAPLG00000011397 | TLR2 | 0,00360 | 0,00472 | 0,00383 | 0,00318 | 0,00319 | 0,00063 |
| ENSAPLG00000011399 | TLR2a | 0,00422 | 0,00181 | 0,00516 | 0,00417 | 0,00373 | 0,00225 |
| ENSAPLG00000011477 | AKT3 | 0,00015 | 0,00000 | 0,00028 | 0,00000 | 0,00028 | 0,00028 |
| ENSAPLG00000011503 | CHUK | 0,00045 | 0,00085 | 0,00026 | 0,00018 | 0,00035 | 0,00000 |
| ENSAPLG00000011572 | AvBD8 | 0,01909 | 0,01613 | 0,01546 | 0,00793 | 0,00403 | 0,00000 |
| ENSAPLG00000011675 | PIK3R2 | 0,00156 | 0,00091 | 0,00239 | 0,00145 | 0,00179 | 0,00199 |
| ENSAPLG00000011754 | IL6 | 0,00041 | 0,00000 | 0,00000 | 0,00041 | 0,00000 | 0,00000 |
| ENSAPLG00000011919 | AvBD13 | 0,00376 | 0,00524 | 0,00417 | 0,00524 | 0,00208 | 0,00000 |
| ENSAPLG00000011922 | AvBD12 | 0,01867 | 0,01027 | 0,02069 | 0,01414 | 0,01411 | 0,00456 |
| ENSAPLG00000011923 | AvBD11 | 0,01809 | 0,00896 | 0,01109 | 0,01365 | 0,00599 | 0,00289 |
| ENSAPLG00000011932 | AvBD10 | 0,00258 | 0,00685 | 0,00554 | 0,00652 | 0,00184 | 0,00350 |
| ENSAPLG00000011933 | TRAF3 | 0,00011 | 0,00078 | 0,00000 | 0,00000 | 0,00000 | 0,00000 |
| ENSAPLG00000012022 | TYK2 | 0,00257 | 0,00226 | 0,00229 | 0,00105 | 0,00268 | 0,00223 |
| ENSAPLG00000012047 | JAK1 | 0,00056 | 0,00018 | 0,00065 | 0,00040 | 0,00089 | 0,00107 |
| ENSAPLG00000012056 | AvBD9 | 0,01735 | 0,01843 | 0,01619 | 0,01853 | 0,00906 | 0,00099 |
| ENSAPLG00000012239 | AvBD7 | 0,00455 | 0,00660 | 0,00000 | 0,00284 | 0,00095 | 0,00000 |
| ENSAPLG00000012251 | AvBD2 | 0,00752 | 0,00844 | 0,00403 | 0,00752 | 0,00461 | 0,00106 |
| ENSAPLG00000012255 | IRAK4 | 0,00491 | 0,00700 | 0,00792 | 0,00808 | 0,00866 | 0,00353 |
| ENSAPLG00000012287 | AvBD1 | 0,00437 | 0,01008 | 0,00373 | 0,00342 | 0,00706 | 0,00095 |
| ENSAPLG00000012288 | AvBD16_or_AvBD3a | 0,01559 | 0,02242 | 0,00611 | 0,01100 | 0,00614 | 0,00178 |
| ENSAPLG00000012380 | AZI2 | 0,00290 | 0,00142 | 0,00411 | 0,00394 | 0,00165 | 0,00382 |
| ENSAPLG00000012383 | CCL4 b | 0,00000 | 0,00000 | 0,00000 | 0,00000 | 0,00000 | 0,00000 |
| ENSAPLG00000012465 | AvBD5 | 0,00397 | 0,00587 | 0,00455 | 0,00189 | 0,00360 | 0,00095 |
| ENSAPLG00000012580 | AvBD14 | 0,00889 | 0,01054 | 0,01117 | 0,00962 | 0,00760 | 0,01610 |
| ENSAPLG00000012625 | TLR4 | 0,00297 | 0,00273 | 0,00346 | 0,00312 | 0,00319 | 0,00274 |
| ENSAPLG00000012692 | IL8-like b | 0,00000 | 0,00000 | 0,00060 | 0,00060 | 0,00422 | 0,00115 |
| ENSAPLG00000012697 | IL8-like a | 0,00121 | 0,00000 | 0,00000 | 0,00117 | 0,00000 | 0,00000 |
| ENSAPLG00000012768 | CTSK | 0,00210 | 0,00100 | 0,00112 | 0,00088 | 0,00056 | 0,00000 |
| ENSAPLG00000012773 | CCL4 a | 0,02917 | 0,03357 | 0,03364 | 0,02943 | 0,03272 | 0,03355 |
| ENSAPLG00000012876 | IFNG | 0,00074 | 0,00038 | 0,00038 | 0,00038 | 0,00076 | 0,00000 |
| ENSAPLG00000012924 | PIK3CA isoform 1 | 0,00028 | 0,00000 | 0,00028 | 0,00018 | 0,00009 | 0,00000 |
| ENSAPLG00000012924 | PIK3CA isoform 1 | 0,00000 | 0,00000 | 0,00000 | 0,00000 | 0,00000 | 0,00000 |
| ENSAPLG00000013074 | MAP2K4 | 0,00016 | 0,00104 | 0,00000 | 0,00016 | 0,00030 | 0,00000 |
| ENSAPLG00000013226 | STAT1 b | 0,00000 | 0,00000 | 0,00011 | 0,00022 | 0,00000 | 0,00000 |
| ENSAPLG00000013262 | STAT1 a | 0,00000 | 0,00035 | 0,00000 | 0,00035 | 0,00000 | 0,00000 |
| ENSAPLG00000013460 | MAPK9 | 0,00104 | 0,00064 | 0,00113 | 0,00141 | 0,00132 | 0,00119 |
| ENSAPLG00000013488 | TRAF6 | 0,00098 | 0,00088 | 0,00129 | 0,00080 | 0,00076 | 0,00076 |
| ENSAPLG00000013556 | CASP1 | 0,01736 | 0,01625 | 0,02019 | 0,01867 | 0,01715 | 0,00261 |
| ENSAPLG00000013558 | CD40 | 0,00151 | 0,00345 | 0,00260 | 0,00336 | 0,00252 | 0,00241 |
| ENSAPLG00000013878 | NFKB1 | 0,00117 | 0,00055 | 0,00076 | 0,00057 | 0,00049 | 0,00000 |
| ENSAPLG00000014351 | IKBKE | 0,00616 | 0,00580 | 0,00600 | 0,00581 | 0,00491 | 0,00421 |
| ENSAPLG00000014359 | TICAM1 | 0,02267 | 0,01807 | 0,02280 | 0,02105 | 0,02158 | 0,01112 |
| ENSAPLG00000014377 | CD14 isoform 1 | 0,01503 | 0,01307 | 0,01240 | 0,01474 | 0,01281 | 0,00859 |
| ENSAPLG00000014377 | CD14 isoform 1 | 0,01503 | 0,01832 | 0,01370 | 0,01590 | 0,01358 | 0,00927 |
| ENSAPLG00000014394 | TNFRSF6B | 0,00401 | 0,00357 | 0,00065 | 0,00210 | 0,00283 | 0,00138 |
| ENSAPLG00000014631 | TMEM173 | 0,00901 | 0,00975 | 0,01297 | 0,00968 | 0,00932 | 0,01069 |
| ENSAPLG00000014872 | CASP8 | 0,00217 | 0,00106 | 0,00259 | 0,00280 | 0,00195 | 0,00066 |
| ENSAPLG00000014996 | IFITM3 | 0,01455 | 0,00000 | 0,00000 | 0,00741 | 0,00409 | 0,00000 |
| ENSAPLG00000015091 | DDX58 | 0,00041 | 0,00052 | 0,00015 | 0,00022 | 0,00015 | 0,00000 |
| ENSAPLG00000015131 | TAB1 | 0,00022 | 0,00074 | 0,00000 | 0,00011 | 0,00033 | 0,00000 |
| ENSAPLG00000015244 | CASP10 | 0,00630 | 0,00754 | 0,00741 | 0,00646 | 0,00462 | 0,00052 |
| ENSAPLG00000015420 | RIPK1 | 0,00602 | 0,00653 | 0,00577 | 0,00547 | 0,00586 | 0,00454 |
| ENSAPLG00000015480 | MAP2K1 | 0,00000 | 0,00000 | 0,00017 | 0,00017 | 0,00017 | 0,00000 |
| ENSAPLG00000015976 | Mx | 0,00415 | 0,00218 | 0,00374 | 0,00465 | 0,00493 | 0,00322 |
| ENSAPLG00000016065 | PIK3R5 | 0,00442 | 0,00203 | 0,00296 | 0,00440 | 0,00422 | 0,00126 |
| ENSAPLG00000016230 | TRAF2 | 0,00000 | 0,00000 | 0,00023 | 0,00000 | 0,00000 | 0,00000 |
| ENSAPLG00000016235 | AKT2 | 0,00555 | 0,00420 | 0,00528 | 0,00606 | 0,00546 | 0,00194 |
| ENSAPLG00000016314 | AKT1 | 0,00013 | 0,00013 | 0,00013 | 0,00013 | 0,00000 | 0,00000 |
| ENSAPLG00000016448 | MAPK8 | 0,00015 | 0,00000 | 0,00000 | 0,00000 | 0,00000 | 0,00000 |

^a^ TRIM27L = TRIM27-like

^b^ CCL24 = CCL24orCCL4

#### Table S11 Nuceotide diversity per gene and mallard population

| Ensembl ID | Gene ID | Canada | Greenland | Spain | Sweden | Farm | Pekin |
| --- | --- | --- | --- | --- | --- | --- | --- |
| ENSAPLG00000000983 | cathelicidinlike | 0,000 | 0,000 | 0,001 | 0,000 | 0,000 | 0,000 |
| ENSAPLG00000001279 | TLR5 | 0,007 | 0,007 | 0,004 | 0,002 | 0,003 | 0,000 |
| ENSAPLG00000001297 | AvBD4 | 0,004 | 0,007 | 0,006 | 0,006 | 0,002 | 0,004 |
| ENSAPLG00000001534 | PML | 0,009 | 0,008 | 0,007 | 0,009 | 0,007 | 0,001 |
| ENSAPLG00000001925 | TIRAP | 0,007 | 0,007 | 0,007 | 0,007 | 0,006 | 0,005 |
| ENSAPLG00000002012 | IFNGR1 | 0,003 | 0,003 | 0,003 | 0,003 | 0,003 | 0,003 |
| ENSAPLG00000002049 | TLR1A | 0,005 | 0,005 | 0,004 | 0,005 | 0,004 | 0,003 |
| ENSAPLG00000002168 | MAPK13 | 0,005 | 0,004 | 0,005 | 0,005 | 0,004 | 0,002 |
| ENSAPLG00000002299 | LITAF | 0,000 | 0,000 | 0,000 | 0,000 | 0,000 | 0,000 |
| ENSAPLG00000002603 | MAPK14 | 0,002 | 0,001 | 0,002 | 0,002 | 0,002 | 0,001 |
| ENSAPLG00000002702 | MAP3K7 | 0,001 | 0,001 | 0,001 | 0,001 | 0,001 | 0,001 |
| ENSAPLG00000002771 | TRIM27L isoform 1^a^ | 0,009 | 0,009 | 0,009 | 0,008 | 0,008 | 0,006 |
| ENSAPLG00000002771 | TRIM27L isoform 2^a^ | 0,013 | 0,010 | 0,012 | 0,013 | 0,012 | 0,009 |
| ENSAPLG00000003004 | SPP1 | 0,007 | 0,006 | 0,007 | 0,007 | 0,005 | 0,003 |
| ENSAPLG00000003319 | MAVS | 0,018 | 0,014 | 0,020 | 0,022 | 0,019 | 0,013 |
| ENSAPLG00000003430 | IFN | 0,001 | 0,001 | 0,000 | 0,001 | 0,001 | 0,002 |
| ENSAPLG00000003554 | PIK3CB | 0,001 | 0,001 | 0,001 | 0,001 | 0,001 | 0,001 |
| ENSAPLG00000003568 | RAC1 | 0,001 | 0,001 | 0,001 | 0,001 | 0,001 | 0,000 |
| ENSAPLG00000004139 | TLR7 | 0,002 | 0,001 | 0,001 | 0,001 | 0,001 | 0,001 |
| ENSAPLG00000004157 | MAPK12 | 0,003 | 0,002 | 0,003 | 0,003 | 0,003 | 0,001 |
| ENSAPLG00000004376 | IRAK1BP1 | 0,007 | 0,007 | 0,007 | 0,007 | 0,008 | 0,007 |
| ENSAPLG00000004462 | MAPK11 | 0,002 | 0,001 | 0,001 | 0,002 | 0,002 | 0,001 |
| ENSAPLG00000004748 | JAK2 | 0,000 | 0,000 | 0,000 | 0,000 | 0,000 | 0,000 |
| ENSAPLG00000004772 | PIK3R1 | 0,001 | 0,000 | 0,001 | 0,001 | 0,001 | 0,000 |
| ENSAPLG00000004845 | LY96 | 0,005 | 0,001 | 0,004 | 0,004 | 0,004 | 0,000 |
| ENSAPLG00000004947 | TRIM25 | 0,002 | 0,002 | 0,002 | 0,001 | 0,002 | 0,001 |
| ENSAPLG00000005139 | MYD88 b | 0,010 | 0,008 | 0,011 | 0,011 | 0,008 | 0,007 |
| ENSAPLG00000005392 | TRADD | 0,009 | 0,007 | 0,009 | 0,009 | 0,008 | 0,007 |
| ENSAPLG00000005400 | CCL19 | 0,008 | 0,006 | 0,008 | 0,009 | 0,005 | 0,000 |
| ENSAPLG00000005826 | MAP2K6 | 0,004 | 0,002 | 0,004 | 0,003 | 0,003 | 0,002 |
| ENSAPLG00000005856 | MAPK10 | 0,003 | 0,003 | 0,003 | 0,002 | 0,003 | 0,002 |
| ENSAPLG00000005913 | CD86 | 0,002 | 0,001 | 0,002 | 0,003 | 0,002 | 0,002 |
| ENSAPLG00000005942 | IL18 | 0,000 | 0,000 | 0,000 | 0,000 | 0,000 | 0,000 |
| ENSAPLG00000006016 | RSAD2 | 0,003 | 0,002 | 0,003 | 0,002 | 0,001 | 0,001 |
| ENSAPLG00000006116 | MAPK1 | 0,000 | 0,000 | 0,000 | 0,000 | 0,000 | 0,000 |
| ENSAPLG00000006147 | IL12A | 0,009 | 0,007 | 0,009 | 0,008 | 0,008 | 0,006 |
| ENSAPLG00000006203 | IRF5 | 0,010 | 0,008 | 0,010 | 0,009 | 0,008 | 0,008 |
| ENSAPLG00000006379 | IKBKB | 0,000 | 0,000 | 0,000 | 0,000 | 0,000 | 0,000 |
| ENSAPLG00000006669 | PIK3CG | 0,004 | 0,003 | 0,003 | 0,002 | 0,002 | 0,002 |
| ENSAPLG00000006823 | PIK3R3 | 0,005 | 0,004 | 0,004 | 0,004 | 0,004 | 0,001 |
| ENSAPLG00000007148 | MAP2K3 | 0,007 | 0,006 | 0,008 | 0,008 | 0,007 | 0,008 |
| ENSAPLG00000007417 | CD80 | 0,004 | 0,003 | 0,004 | 0,004 | 0,004 | 0,003 |
| ENSAPLG00000007876 | TANK | 0,000 | 0,000 | 0,000 | 0,000 | 0,000 | 0,000 |
| ENSAPLG00000008173 | TAB2 | 0,001 | 0,001 | 0,001 | 0,001 | 0,001 | 0,001 |
| ENSAPLG00000008183 | PIK3CD | 0,005 | 0,004 | 0,005 | 0,004 | 0,005 | 0,004 |
| ENSAPLG00000008445 | TBK1 | 0,004 | 0,002 | 0,004 | 0,004 | 0,003 | 0,002 |
| ENSAPLG00000008655 | LBP | 0,020 | 0,019 | 0,020 | 0,019 | 0,019 | 0,019 |
| ENSAPLG00000008976 | TLR3 | 0,002 | 0,001 | 0,002 | 0,001 | 0,001 | 0,001 |
| ENSAPLG00000009092 | IL12B | 0,001 | 0,000 | 0,001 | 0,001 | 0,001 | 0,000 |
| ENSAPLG00000009156 | TOLLIP | 0,001 | 0,000 | 0,001 | 0,001 | 0,001 | 0,001 |
| ENSAPLG00000009205 | MAP3K8 | 0,003 | 0,003 | 0,003 | 0,004 | 0,002 | 0,002 |
| ENSAPLG00000009391 | CCL24 isoform 1 ^b^ | 0,000 | 0,001 | 0,000 | 0,001 | 0,000 | 0,000 |
| ENSAPLG00000009391 | CCL24 isoform 2 ^b^ | 0,002 | 0,004 | 0,001 | 0,001 | 0,001 | 0,000 |
| ENSAPLG00000009793 | CCL5 a | 0,024 | 0,029 | 0,027 | 0,030 | 0,030 | 0,011 |
| ENSAPLG00000009838 | CCL5 b | 0,004 | 0,002 | 0,005 | 0,004 | 0,004 | 0,001 |
| ENSAPLG00000009929 | TLR15 | 0,004 | 0,005 | 0,004 | 0,004 | 0,004 | 0,003 |
| ENSAPLG00000010090 | NLRP12 | 0,006 | 0,003 | 0,005 | 0,005 | 0,003 | 0,001 |
| ENSAPLG00000010194 | MYD88 a | 0,002 | 0,002 | 0,003 | 0,003 | 0,003 | 0,004 |
| ENSAPLG00000010913 | IFIH1 | 0,001 | 0,000 | 0,001 | 0,001 | 0,001 | 0,000 |
| ENSAPLG00000011028 | NFKBIA | 0,002 | 0,002 | 0,002 | 0,002 | 0,002 | 0,001 |
| ENSAPLG00000011045 | MAP2K2 | 0,003 | 0,001 | 0,002 | 0,002 | 0,003 | 0,002 |
| ENSAPLG00000011237 | IFNGR2 | 0,007 | 0,006 | 0,007 | 0,006 | 0,007 | 0,005 |
| ENSAPLG00000011322 | IFNAR1 | 0,002 | 0,000 | 0,001 | 0,001 | 0,002 | 0,001 |
| ENSAPLG00000011364 | IFNAR2 | 0,010 | 0,010 | 0,009 | 0,009 | 0,008 | 0,009 |
| ENSAPLG00000011397 | TLR2 | 0,004 | 0,004 | 0,004 | 0,003 | 0,003 | 0,001 |
| ENSAPLG00000011399 | TLR2a | 0,005 | 0,002 | 0,005 | 0,004 | 0,005 | 0,001 |
| ENSAPLG00000011477 | AKT3 | 0,001 | 0,001 | 0,001 | 0,001 | 0,000 | 0,000 |
| ENSAPLG00000011503 | CHUK | 0,001 | 0,002 | 0,002 | 0,001 | 0,001 | 0,001 |
| ENSAPLG00000011572 | AvBD8 | 0,012 | 0,005 | 0,011 | 0,007 | 0,004 | 0,006 |
| ENSAPLG00000011675 | PIK3R2 | 0,006 | 0,004 | 0,006 | 0,006 | 0,005 | 0,003 |
| ENSAPLG00000011754 | IL6 | 0,002 | 0,000 | 0,002 | 0,002 | 0,000 | 0,000 |
| ENSAPLG00000011919 | AvBD13 | 0,004 | 0,005 | 0,003 | 0,004 | 0,002 | 0,000 |
| ENSAPLG00000011922 | AvBD12 | 0,021 | 0,015 | 0,020 | 0,018 | 0,011 | 0,002 |
| ENSAPLG00000011923 | AvBD11 | 0,018 | 0,014 | 0,016 | 0,018 | 0,012 | 0,004 |
| ENSAPLG00000011932 | AvBD10 | 0,020 | 0,012 | 0,018 | 0,018 | 0,014 | 0,004 |
| ENSAPLG00000011933 | TRAF3 | 0,000 | 0,002 | 0,001 | 0,001 | 0,001 | 0,000 |
| ENSAPLG00000012022 | TYK2 | 0,005 | 0,005 | 0,006 | 0,005 | 0,005 | 0,005 |
| ENSAPLG00000012047 | JAK1 | 0,005 | 0,004 | 0,005 | 0,005 | 0,005 | 0,005 |
| ENSAPLG00000012056 | AvBD9 | 0,012 | 0,014 | 0,012 | 0,013 | 0,006 | 0,001 |
| ENSAPLG00000012239 | AvBD7 | 0,007 | 0,007 | 0,005 | 0,006 | 0,004 | 0,003 |
| ENSAPLG00000012251 | AvBD2 | 0,010 | 0,006 | 0,007 | 0,010 | 0,008 | 0,004 |
| ENSAPLG00000012255 | IRAK4 | 0,005 | 0,006 | 0,006 | 0,007 | 0,006 | 0,002 |
| ENSAPLG00000012287 | AvBD1 | 0,001 | 0,003 | 0,002 | 0,001 | 0,002 | 0,000 |
| ENSAPLG00000012288 | AvBD16_or_AvBD3a | 0,007 | 0,010 | 0,002 | 0,005 | 0,005 | 0,001 |
| ENSAPLG00000012380 | AZI2 | 0,003 | 0,002 | 0,004 | 0,004 | 0,003 | 0,004 |
| ENSAPLG00000012383 | CCL4 b | 0,000 | 0,001 | 0,000 | 0,000 | 0,000 | 0,000 |
| ENSAPLG00000012465 | AvBD5 | 0,006 | 0,007 | 0,006 | 0,003 | 0,003 | 0,000 |
| ENSAPLG00000012580 | AvBD14 | 0,006 | 0,007 | 0,007 | 0,006 | 0,004 | 0,005 |
| ENSAPLG00000012625 | TLR4 | 0,003 | 0,002 | 0,003 | 0,003 | 0,003 | 0,003 |
| ENSAPLG00000012692 | IL8-like b | 0,001 | 0,000 | 0,001 | 0,000 | 0,001 | 0,000 |
| ENSAPLG00000012697 | IL8-like a | 0,001 | 0,000 | 0,001 | 0,002 | 0,000 | 0,000 |
| ENSAPLG00000012768 | CTSK | 0,002 | 0,001 | 0,001 | 0,001 | 0,001 | 0,000 |
| ENSAPLG00000012773 | CCL4 a | 0,012 | 0,012 | 0,014 | 0,012 | 0,012 | 0,011 |
| ENSAPLG00000012876 | IFNG | 0,002 | 0,001 | 0,003 | 0,002 | 0,002 | 0,000 |
| ENSAPLG00000012924 | PIK3CA isoform 1 | 0,001 | 0,001 | 0,001 | 0,001 | 0,001 | 0,000 |
| ENSAPLG00000012924 | PIK3CA isoform 2 | 0,001 | 0,001 | 0,001 | 0,001 | 0,001 | 0,000 |
| ENSAPLG00000013074 | MAP2K4 | 0,000 | 0,000 | 0,000 | 0,001 | 0,001 | 0,001 |
| ENSAPLG00000013226 | STAT1 b | 0,003 | 0,004 | 0,003 | 0,004 | 0,003 | 0,001 |
| ENSAPLG00000013262 | STAT1 a | 0,010 | 0,006 | 0,011 | 0,010 | 0,007 | 0,001 |
| ENSAPLG00000013460 | MAPK9 | 0,001 | 0,000 | 0,001 | 0,001 | 0,001 | 0,001 |
| ENSAPLG00000013488 | TRAF6 | 0,003 | 0,001 | 0,002 | 0,002 | 0,003 | 0,002 |
| ENSAPLG00000013556 | CASP1 | 0,010 | 0,010 | 0,012 | 0,012 | 0,011 | 0,002 |
| ENSAPLG00000013558 | CD40 | 0,004 | 0,004 | 0,003 | 0,004 | 0,004 | 0,003 |
| ENSAPLG00000013878 | NFKB1 | 0,004 | 0,003 | 0,004 | 0,003 | 0,003 | 0,001 |
| ENSAPLG00000014351 | IKBKE | 0,010 | 0,007 | 0,009 | 0,010 | 0,008 | 0,006 |
| ENSAPLG00000014359 | TICAM1 | 0,013 | 0,011 | 0,013 | 0,012 | 0,012 | 0,006 |
| ENSAPLG00000014377 | CD14 isoform 1 | 0,013 | 0,014 | 0,012 | 0,013 | 0,012 | 0,008 |
| ENSAPLG00000014377 | CD14 isoform 2 | 0,011 | 0,010 | 0,008 | 0,010 | 0,010 | 0,007 |
| ENSAPLG00000014394 | TNFRSF6B | 0,005 | 0,005 | 0,004 | 0,006 | 0,005 | 0,004 |
| ENSAPLG00000014631 | TMEM173 | 0,012 | 0,009 | 0,012 | 0,013 | 0,012 | 0,010 |
| ENSAPLG00000014872 | CASP8 | 0,001 | 0,001 | 0,001 | 0,001 | 0,001 | 0,000 |
| ENSAPLG00000014996 | IFITM3 | 0,008 | 0,000 | 0,000 | 0,003 | 0,002 | 0,000 |
| ENSAPLG00000015091 | DDX58 | 0,001 | 0,001 | 0,000 | 0,001 | 0,000 | 0,000 |
| ENSAPLG00000015131 | TAB1 | 0,003 | 0,003 | 0,003 | 0,003 | 0,003 | 0,001 |
| ENSAPLG00000015244 | CASP10 | 0,003 | 0,004 | 0,004 | 0,004 | 0,003 | 0,000 |
| ENSAPLG00000015420 | RIPK1 | 0,006 | 0,005 | 0,007 | 0,007 | 0,007 | 0,005 |
| ENSAPLG00000015480 | MAP2K1 | 0,002 | 0,002 | 0,002 | 0,002 | 0,002 | 0,000 |
| ENSAPLG00000015976 | Mx | 0,003 | 0,002 | 0,002 | 0,003 | 0,002 | 0,001 |
| ENSAPLG00000016065 | PIK3R5 | 0,007 | 0,005 | 0,007 | 0,007 | 0,007 | 0,004 |
| ENSAPLG00000016230 | TRAF2 | 0,001 | 0,001 | 0,002 | 0,002 | 0,002 | 0,002 |
| ENSAPLG00000016235 | AKT2 | 0,006 | 0,005 | 0,004 | 0,004 | 0,004 | 0,003 |
| ENSAPLG00000016314 | AKT1 | 0,001 | 0,001 | 0,001 | 0,001 | 0,001 | 0,001 |
| ENSAPLG00000016448 | MAPK8 | 0,000 | 0,000 | 0,000 | 0,000 | 0,000 | 0,000 |

^a^ TRIM27L = TRIM27-like

^b^ CCL24 = CCL24orCCL4

#### Table S12 Pairwise genetic distances (F_ST_) between populations

| *F*_ST_ | Spain | Greenland | Farm | Pekin | Swedish |
| --- | --- | --- | --- | --- | --- |
| Canada | 0.013 | 0.087 | 0.060 | 0.187 | 0.013 |
| Spain |  | 0.090 | 0.058 | 0.189 | 0.007 |
| Greenland |  |  | 0.141 | 0.265 | 0.085 |
| Farm |  |  |  | 0.179 | 0.053 |
| Pekin |  |  |  |  | 0.179 |

Canada n=16, Greenland n=16, Spain n=16, Sweden n=16, Farm n=16, Pekin ducks n=16.

#### Table S13 Pairwise genetic distances (F_ST_) between populations when including non-synonymous nucleotides only

| *F*_ST_ | Spain | Greenland | Farm | Pekin | Swedish |
| --- | --- | --- | --- | --- | --- |
| Canada | 0,014 | 0,074 | 0,050 | 0,143 | 0,012 |
| Spain |  | 0,079 | 0,049 | 0,148 | 0,009 |
| Greenland |  |  | 0,132 | 0,212 | 0,072 |
| Farm |  |  |  | 0,155 | 0,046 |
| Pekin |  |  |  |  | 0,129 |

#### Table S14. Results from site models comparisons (M1a/M2a and M7/M8) in the codeml program in PAML.

| Ensembl ID | Gene ID | LRT 1vs2 | LRT 7vs8 | FDR 1vs2 | FDR 7vs8 |
| --- | --- | --- | --- | --- | --- |
| ENSAPLG00000000983 | cathelicidinlike | 32,463 | 34,311 | 0,000 | 0,000 |
| ENSAPLG00000001279 | TLR5 | 80,493 | 81,791 | 0,000 | 0,000 |
| ENSAPLG00000001297 | AvBD4 | 22,778 | 25,281 | 0,000 | 0,000 |
| ENSAPLG00000001534 | PML | 1,574 | 2,195 | 0,885 | 0,603 |
| ENSAPLG00000001925 | TIRAP | 8,605 | 9,683 | 0,043 | 0,022 |
| ENSAPLG00000002012 | IFNGR1 | 30,463 | 31,377 | 0,000 | 0,000 |
| ENSAPLG00000002049 | TLR1A | 0,014 | 16,709 | 1,000 | 0,001 |
| ENSAPLG00000002168 | MAPK13 | 1,836 | 1,864 | 0,821 | 0,691 |
| ENSAPLG00000002299 | LITAF | 0,000 | 1,716 | 1,000 | 0,732 |
| ENSAPLG00000002603 | MAPK14 | 1,652 | 1,680 | 0,868 | 0,733 |
| ENSAPLG00000003004 | SPP1 | 2,552 | 2,683 | 0,610 | 0,500 |
| ENSAPLG00000003319 | MAVS | 59,538 | 59,453 | 0,000 | 0,000 |
| ENSAPLG00000003430 | IFN | 0,002 | 3,737 | 1,000 | 0,324 |
| ENSAPLG00000003554 | PIK3CB | 4,457 | 5,397 | 0,274 | 0,150 |
| ENSAPLG00000003568 | RAC1 | 1,421 | 1,591 | 0,939 | 0,755 |
| ENSAPLG00000004139 | TLR7 | 23,075 | 15,740 | 0,000 | 0,002 |
| ENSAPLG00000004157 | MAPK12 | 0,669 | 2,606 | 1,000 | 0,510 |
| ENSAPLG00000004462 | MAPK11 | 0,000 | 1,544 | 1,000 | 0,761 |
| ENSAPLG00000004748 | JAK2 | 4,058 | 12,775 | 0,323 | 0,005 |
| ENSAPLG00000004845 | LY96 | 2,145 | 2,386 | 0,718 | 0,559 |
| ENSAPLG00000004947 | TRIM25 | 0,802 | 5,460 | 1,000 | 0,148 |
| ENSAPLG00000005392 | TRADD | 3,702 | 4,940 | 0,373 | 0,185 |
| ENSAPLG00000005400 | CCL19 | 0,682 | 1,435 | 1,000 | 0,791 |
| ENSAPLG00000005826 | MAP2K6 | 4,605 | -0,175 | 0,261 | 1,000 |
| ENSAPLG00000005856 | MAPK10 | 1,384 | -1,130 | 0,940 | 1,000 |
| ENSAPLG00000005913 | CD86 | 2,856 | 3,020 | 0,558 | 0,446 |
| ENSAPLG00000005928 | JUN | 5,598 | 5,939 | 0,163 | 0,122 |
| ENSAPLG00000005942 | IL18 | 1,320 | -2,354 | 0,950 | 1,000 |
| ENSAPLG00000006016 | RSAD2 | 20,714 | 21,510 | 0,000 | 0,000 |
| ENSAPLG00000006116 | MAPK1 | 0,000 | -0,043 | 1,000 | 1,000 |
| ENSAPLG00000006147 | IL12A | 6,607 | 6,606 | 0,103 | 0,094 |
| ENSAPLG00000006203 | IRF5 | 52,247 | 52,912 | 0,000 | 0,000 |
| ENSAPLG00000006379 | IKBKB | -0,003 | 0,003 | 1,000 | 1,000 |
| ENSAPLG00000006669 | PIK3CG | 0,001 | -0,004 | 1,000 | 1,000 |
| ENSAPLG00000007148 | MAP2K3 | 6,309 | 6,723 | 0,117 | 0,091 |
| ENSAPLG00000007417 | CD80 | 1,293 | 6,050 | 0,950 | 0,118 |
| ENSAPLG00000007876 | TANK | -1,293 | -0,002 | 1,000 | 1,000 |
| ENSAPLG00000008173 | TAB2 | 17,902 | 18,688 | 0,001 | 0,000 |
| ENSAPLG00000008183 | PIK3CD | 1,695 | 2,169 | 0,865 | 0,603 |
| ENSAPLG00000008445 | TBK1 | 0,098 | 0,730 | 1,000 | 1,000 |
| ENSAPLG00000008655 | LBP | 35,975 | 37,421 | 0,000 | 0,000 |
| ENSAPLG00000008976 | TLR3 | 0,138 | 0,342 | 1,000 | 1,000 |
| ENSAPLG00000009092 | IL12B | 0,001 | -0,001 | 1,000 | 1,000 |
| ENSAPLG00000009156 | TOLLIP | 0,001 | -0,006 | 1,000 | 1,000 |
| ENSAPLG00000009205 | MAP3K8 | 13,477 | 13,671 | 0,004 | 0,004 |
| ENSAPLG00000009391 | CCL24 isoform 2 ^b^ | 13,736 | 14,152 | 0,004 | 0,003 |
| ENSAPLG00000009391 | CCL24 isoform 1 ^b^ | 11,926 | 13,975 | 0,009 | 0,003 |
| ENSAPLG00000009838 | CCL5 b | 2,253 | 9,893 | 0,694 | 0,021 |
| ENSAPLG00000009929 | TLR15 | 0,000 | 0,014 | 1,000 | 1,000 |
| ENSAPLG00000010090 | NLRP12 | 18,231 | 18,529 | 0,001 | 0,000 |
| ENSAPLG00000010194 | MYD88 a | 0,001 | 0,051 | 1,000 | 1,000 |
| ENSAPLG00000011028 | NFKBIA | 0,823 | 0,823 | 1,000 | 1,000 |
| ENSAPLG00000011045 | MAP2K2 | 0,001 | 0,004 | 1,000 | 1,000 |
| ENSAPLG00000011284 | FOS | 0,592 | 0,715 | 1,000 | 1,000 |
| ENSAPLG00000011364 | IFNAR2 | 203,864 | 141,703 | 0,000 | 0,000 |
| ENSAPLG00000011397 | TLR2 | 107,898 | 108,350 | 0,000 | 0,000 |
| ENSAPLG00000011399 | TLR2a | 47,131 | 48,225 | 0,000 | 0,000 |
| ENSAPLG00000011477 | AKT3 | 24,421 | 24,783 | 0,000 | 0,000 |
| ENSAPLG00000011503 | CHUK | 0,000 | 0,000 | 1,000 | 1,000 |
| ENSAPLG00000011572 | AvBD8 | 0,000 | 0,002 | 1,000 | 1,000 |
| ENSAPLG00000011675 | PIK3R2 | 0,697 | 0,710 | 1,000 | 1,000 |
| ENSAPLG00000011754 | IL6 | -1,007 | 0,149 | 1,000 | 1,000 |
| ENSAPLG00000011919 | AvBD13 | 2,777 | 2,982 | 0,568 | 0,446 |
| ENSAPLG00000011922 | AvBD12 | 17,084 | 17,487 | 0,001 | 0,001 |
| ENSAPLG00000011932 | AvBD10 | 0,016 | -0,002 | 1,000 | 1,000 |
| ENSAPLG00000011933 | TRAF3 | 0,000 | 0,011 | 1,000 | 1,000 |
| ENSAPLG00000012047 | JAK1 | 0,000 | 6,053 | 1,000 | 0,118 |
| ENSAPLG00000012056 | AvBD9 | 61,029 | 40,103 | 0,000 | 0,000 |
| ENSAPLG00000012088 | SOCS3 | 0,000 | 0,233 | 1,000 | 1,000 |
| ENSAPLG00000012239 | AvBD7 | 39,315 | 31,388 | 0,000 | 0,000 |
| ENSAPLG00000012251 | AvBD2 | 2,636 | 3,565 | 0,597 | 0,346 |
| ENSAPLG00000012287 | AvBD1 | 18,210 | 18,499 | 0,001 | 0,000 |
| ENSAPLG00000012383 | CCL4 b | 1,108 | 2,681 | 1,000 | 0,500 |
| ENSAPLG00000012465 | AvBD5 | 0,104 | 0,280 | 1,000 | 1,000 |
| ENSAPLG00000012580 | AvBD14 | 0,000 | 0,276 | 1,000 | 1,000 |
| ENSAPLG00000012625 | TLR4 | 7,206 | 7,265 | 0,079 | 0,071 |
| ENSAPLG00000012692 | IL8-like b | 12,908 | 13,091 | 0,006 | 0,005 |
| ENSAPLG00000012697 | IL8-like a | 0,000 | 0,018 | 1,000 | 1,000 |
| ENSAPLG00000012752 | IRF7 | 0,000 | -0,007 | 1,000 | 1,000 |
| ENSAPLG00000012768 | CTSK | 0,001 | -0,001 | 1,000 | 1,000 |
| ENSAPLG00000012773 | CCL4 a | 0,000 | -0,007 | 1,000 | 1,000 |
| ENSAPLG00000012876 | IFNG | 0,734 | 0,793 | 1,000 | 1,000 |
| ENSAPLG00000012924 | PIK3CA isoform 1 | 12,211 | 58,602 | 0,008 | 0,000 |
| ENSAPLG00000013226 | STAT1 b | 0,744 | -0,885 | 1,000 | 1,000 |
| ENSAPLG00000013262 | STAT1 a | 0,140 | 0,117 | 1,000 | 1,000 |
| ENSAPLG00000013460 | MAPK9 | 0,000 | 0,000 | 1,000 | 1,000 |
| ENSAPLG00000013488 | TRAF6 | 7,581 | 12,089 | 0,068 | 0,007 |
| ENSAPLG00000013556 | CASP1 | 7,565 | 7,745 | 0,068 | 0,057 |
| ENSAPLG00000013558 | CD40 | 9,921 | 10,117 | 0,023 | 0,019 |
| ENSAPLG00000013878 | NFKB1 | 15,503 | 13,744 | 0,002 | 0,004 |
| ENSAPLG00000014359 | TICAM1 | 50,020 | 63,880 | 0,000 | 0,000 |
| ENSAPLG00000014394 | TNFRSF6B | -0,003 | 0,000 | 1,000 | 1,000 |
| ENSAPLG00000014631 | TMEM173 | 0,000 | 0,006 | 1,000 | 1,000 |
| ENSAPLG00000014726 | TLR21 | 26,008 | 26,219 | 0,000 | 0,000 |
| ENSAPLG00000014872 | CASP8 | 0,000 | -0,005 | 1,000 | 1,000 |
| ENSAPLG00000014996 | IFITM3 | 28,929 | 29,571 | 0,000 | 0,000 |
| ENSAPLG00000015091 | DDX58 | -4,986 | 5,696 | 1,000 | 0,135 |
| ENSAPLG00000015131 | TAB1 | 0,001 | -0,001 | 1,000 | 1,000 |
| ENSAPLG00000015244 | CASP10 | 0,000 | 0,358 | 1,000 | 1,000 |
| ENSAPLG00000015420 | RIPK1 | 14,943 | 13,968 | 0,002 | 0,003 |
| ENSAPLG00000015480 | MAP2K1 | -3,632 | 0,230 | 1,000 | 1,000 |
| ENSAPLG00000015976 | Mx | 27,668 | 27,884 | 0,000 | 0,000 |
| ENSAPLG00000016065 | PIK3R5 | 30,120 | 30,228 | 0,000 | 0,000 |
| ENSAPLG00000016230 | TRAF2 | 0,000 | -0,001 | 1,000 | 1,000 |
| ENSAPLG00000016235 | AKT2 | 4,040 | 4,220 | 0,323 | 0,259 |
| ENSAPLG00000016448 | MAPK8 | 0,000 | -0,001 | 1,000 | 1,000 |

Likelihood ratio tests (LRT) were performed by calculating double the difference in log likelihood values between the alternative model (M2a or M8) and the null model (M1 or M7). FDR-adjusted *P* values were obtained from the chi-squared distribution with 2 degrees of freedom (calculated as the difference between the numbers of parameters included in each model). Comparisons with FDR-adjusted *p* values < 0.05 were considered to be statistically significant.

^b^ CCL24 = CCL24orCCL4

#### Table S15 LRT of positive selection (random sites model in PAML) for the innate immune coding sequences

| Ensembl ID | Gene ID | tree_length | omega | LRT_0vs3 | LRT_1vs2 | w0_M2 | p0_M2 | w2_M2 | p2_M2 | Positively_selected_sites_(M2,_BEB)^a^ |
| --- | --- | --- | --- | --- | --- | --- | --- | --- | --- | --- |
| ENSAPLG00000000983 | cathelicidinlike | 0.703 | 1.163 | 59.091*** | 32.463*** | 0.302 | 0.890 | 446.067 | 0.110 | **4**,**6**,7 |
| ENSAPLG00000001279 | TLR5 | 0.370 | 0.953 | 135.321*** | 80.497*** | 0.000 | 0.487 | 8.302 | 0.074 | *33*,*72*,**73**,**104**,*106*,**123**,*128*,*205*,*235*,*279*,*339*,*392*,**506**,*532*,*677* |
| ENSAPLG00000001297 | AvBD4 | 0.534 | 6.100 | 17.925** | 28.441*** | 1.000 | 0.801 | 30.811 | 0.198 | 1,*4*,7,*29*,**39** |
| ENSAPLG00000001534 | PML | 0.559 | 0.179 | 19.625** | 0.552^NS^ | 0.077 | 0.871 | 1.000 | 0.074 |  |
| ENSAPLG00000001925 | TIRAP | 0.272 | 0.244 | 37.120*** | 6.607^NS^ | 0.000 | 0.797 | 35.571 | 0.005 |  |
| ENSAPLG00000002012 | IFNGR1 | 0.357 | 0.809 | 48.913*** | 30.121*** | 0.027 | 0.404 | 11.541 | 0.027 | *28*,140,**158** |
| ENSAPLG00000002049 | TLR1A | 0.285 | 0.392 | 55.218*** | 16.836*** | 0.080 | 0.742 | 7.919 | 0.014 | **516**,**522**,**526**,751 |
| ENSAPLG00000002168 | MAPK13 | 0.128 | 0.067 | 3.960^NS^ | 0.014^NS^ | 0.000 | 0.939 | 1.127 | 0.061 |  |
| ENSAPLG00000002299 | LITAF | 0.276 | 0.175 | 10.165^NS^ | 0.000^NS^ | 0.000 | 0.849 | 15.971 | 0.000 |  |
| ENSAPLG00000002603 | MAPK14 | 0.077 | 0.000 | 0.013^NS^ | 0.005^NS^ | 0.000 | 1.000 | 1.000 | 0.000 |  |
| ENSAPLG00000003004 | SPP1 | 0.358 | 0.257 | 20.666*** | 1.574^NS^ | 0.038 | 0.858 | 1.844 | 0.142 |  |
| ENSAPLG00000003319 | MAVS | 1.158 | 0.331 | 354.784*** | 59.538*** | 0.039 | 0.736 | 6.607 | 0.029 | 208,*210*,222,**239**,**375**,527,529 |
| ENSAPLG00000003430 | IFN | 0.212 | 0.639 | 5.047^NS^ | 2.145^NS^ | 0.000 | 0.729 | 2.608 | 0.271 |  |
| ENSAPLG00000003554 | PIK3CB | 0.103 | 0.061 | 11.197* | 2.636^NS^ | 0.047 | 0.998 | 8.672 | 0.002 |  |
| ENSAPLG00000003568 | RAC1 | 0.100 | 0.000 | 0.008^NS^ | 0.001^NS^ | 0.000 | 1.000 | 44.751 | 0.000 |  |
| ENSAPLG00000004139 | TLR7 | 0.169 | 0.235 | 50.284*** | 14.943** | 0.063 | 0.870 | 13.358 | 0.007 | **279**,*415*,**483** |
| ENSAPLG00000004157 | MAPK12 | 0.201 | 0.036 | 2.706^NS^ | 0.000^NS^ | 0.010 | 0.972 | 1.000 | 0.025 |  |
| ENSAPLG00000004462 | MAPK11 | 0.157 | 0.000 | 0.025^NS^ | 0.002^NS^ | 0.000 | 1.000 | 22.873 | 0.000 |  |
| ENSAPLG00000004748 | JAK2 | 0.123 | 0.089 | 40.732*** | 8.605* | 0.000 | 0.931 | 18.998 | 0.002 |  |
| ENSAPLG00000004845 | LY96 | 0.195 | 0.735 | 2.684^NS^ | 9.169* | 0.467 | 0.937 | 4.669 | 0.064 |  |
| ENSAPLG00000004947 | TRIM25 | 0.329 | 0.362 | 19.734** | 2.777^NS^ | 0.221 | 0.940 | 3.033 | 0.060 |  |
| ENSAPLG00000005392 | TRADD | 0.171 | 0.116 | 8.532^NS^ | 2.552^NS^ | 0.056 | 0.943 | 18.410 | 0.003 |  |
| ENSAPLG00000005400 | CCL19 | 0.793 | 0.086 | 16.219** | 0.107^NS^ | 0.025 | 0.920 | 2.417 | 0.007 |  |
| ENSAPLG00000005826 | MAP2K6 | 0.121 | 0.009 | 1.293^NS^ | 3.508^NS^ | 0.000 | 0.996 | 4.409 | 0.004 |  |
| ENSAPLG00000005856 | MAPK10 | 0.078 | 0.000 | 0.014^NS^ | 0.001^NS^ | 0.000 | 1.000 | 1.000 | 0.000 |  |
| ENSAPLG00000005913 | CD86 | 0.232 | 0.460 | 4.832^NS^ | 0.403^NS^ | 0.000 | 0.654 | 1.366 | 0.346 |  |
| ENSAPLG00000005928 | JUN | 0.250 | 0.014 | 13.873* | 0.328^NS^ | 0.005 | 0.995 | 1.744 | 0.005 |  |
| ENSAPLG00000005942 | IL18 | 0.190 | 0.223 | 10.136^NS^ | 1.919^NS^ | 0.000 | 0.896 | 2.526 | 0.104 |  |
| ENSAPLG00000006016 | RSAD2 | 0.313 | 0.120 | 95.230*** | 20.714*** | 0.000 | 0.940 | 8.259 | 0.013 | *2*,15 |
| ENSAPLG00000006116 | MAPK1 | 0.072 | 0.000 | -0.190^NS^ | -0.010^NS^ | 0.000 | 1.000 | 1.000 | 0.000 |  |
| ENSAPLG00000006147 | IL12A | 0.435 | 0.178 | 30.729*** | 4.470^NS^ | 0.064 | 0.953 | 3.253 | 0.047 |  |
| ENSAPLG00000006203 | IRF5 | 1.255 | 0.207 | 553.054*** | 50.020*** | 0.013 | 0.835 | 2.732 | 0.104 | **1**,*155*,**159**,*173*,*181*,188,**198**,*206* |
| ENSAPLG00000006379 | IKBKB | 0.118 | 0.049 | -0.008^NS^ | 1.189^NS^ | 0.049 | 1.000 | 1.000 | 0.000 |  |
| ENSAPLG00000006669 | PIK3CG | 0.191 | 0.006 | 0.000^NS^ | 0.001^NS^ | 0.006 | 1.000 | 1.000 | 0.000 |  |
| ENSAPLG00000007148 | MAP2K3 | 0.226 | 0.004 | 4.176^NS^ | -0.212^NS^ | 0.004 | 1.000 | 9.494 | 0.000 |  |
| ENSAPLG00000007417 | CD80 | 0.301 | 0.309 | 15.073** | 4.040^NS^ | 0.215 | 0.977 | 4.961 | 0.023 |  |
| ENSAPLG00000007876 | TANK | 0.061 | 0.220 | 3.485^NS^ | 0.822^NS^ | 0.000 | 0.917 | 2.936 | 0.077 |  |
| ENSAPLG00000008173 | TAB2 | 0.075 | 0.192 | 35.227*** | 17.876*** | 0.106 | 0.996 | 32.473 | 0.004 | 600,*605* |
| ENSAPLG00000008183 | PIK3CD | 0.149 | 0.037 | 19.380** | 1.294^NS^ | 0.000 | 0.980 | 2.021 | 0.020 |  |
| ENSAPLG00000008445 | TBK1 | 0.173 | 0.051 | 7.423^NS^ | 0.000^NS^ | 0.005 | 0.950 | 1.000 | 0.016 |  |
| ENSAPLG00000008655 | LBP | 0.494 | 0.544 | 97.277*** | 48.277*** | 0.000 | 0.599 | 7.885 | 0.038 | **72**,*77*,*356* |
| ENSAPLG00000008976 | TLR3 | 0.169 | 0.334 | 12.316* | 0.593^NS^ | 0.023 | 0.742 | 2.090 | 0.058 |  |
| ENSAPLG00000009092 | IL12B | 0.113 | 0.342 | 0.000^NS^ | 0.000^NS^ | 0.342 | 1.000 | 11.381 | 0.000 |  |
| ENSAPLG00000009156 | TOLLIP | 0.096 | 0.033 | 0.000^NS^ | 0.000^NS^ | 0.033 | 1.000 | 1.000 | 0.000 |  |
| ENSAPLG00000009205 | MAP3K8 | 0.231 | 0.074 | 26.351*** | 11.926** | 0.055 | 0.998 | 16.729 | 0.002 | 59 |
| ENSAPLG00000009391 | CCL24 isoform 1 ^c^ | 0.069 | 0.610 | 17.763** | 12.908** | 0.000 | 0.976 | 70.358 | 0.024 | *47* |
| ENSAPLG00000009391 | CCL24 isoform 2 ^c^ | 0.183 | 0.324 | 26.306*** | 13.736** | 0.060 | 0.970 | 18.655 | 0.030 | **13**,*48* |
| ENSAPLG00000009838 | CCL5 b | 0.217 | 0.563 | 10.621^NS^ | 7.206^NS^ | 0.383 | 0.987 | 18.854 | 0.013 |  |
| ENSAPLG00000009929 | TLR15 | 0.224 | 0.413 | 5.880^NS^ | 0.697^NS^ | 0.312 | 0.958 | 3.408 | 0.039 |  |
| ENSAPLG00000010090 | NLRP12 | 0.339 | 0.376 | 94.578*** | 18.590*** | 0.108 | 0.900 | 3.124 | 0.100 | 385 |
| ENSAPLG00000010194 | MYD88 a | 0.271 | 0.000 | -0.134^NS^ | -0.076^NS^ | 0.000 | 1.000 | 8.624 | 0.000 |  |
| ENSAPLG00000011028 | NFKBIA | 0.165 | 0.000 | 0.019^NS^ | 0.002^NS^ | 0.000 | 1.000 | 6.896 | 0.000 |  |
| ENSAPLG00000011045 | MAP2K2 | 0.114 | 0.025 | 0.000^NS^ | 0.005^NS^ | 0.025 | 1.000 | 1.000 | 0.000 |  |
| ENSAPLG00000011284 | FOS | 0.376 | 0.050 | 27.881*** | 0.000^NS^ | 0.005 | 0.936 | 1.000 | 0.037 |  |
| ENSAPLG00000011364 | IFNAR2 | 0.575 | 1.463 | 197.817*** | 200.705*** | 0.000 | 0.313 | 13.213 | 0.087 | *29*,**33**,**40**,*46*,*51*,**71**,**73**,**76**,**99**,**101**,**102**,**104**,**106**,**108**,**120**,**122**,**126**,*128*,*130*,*149*,*187*,**189**,**225**,*278* |
| ENSAPLG00000011397 | TLR2 | 0.375 | 0.543 | 232.517*** | 107.898*** | 0.000 | 0.683 | 12.106 | 0.026 | **300**,**306**,**307**,**310**,*452*,*454*,*568*,**585**,**594**,**605**,**633** |
| ENSAPLG00000011399 | TLR2a | 0.305 | 0.340 | 133.878*** | 47.131*** | 0.000 | 0.742 | 15.353 | 0.010 | 189,*219*,*363*,**597**,**603** |
| ENSAPLG00000011477 | AKT3 | 0.093 | 0.114 | 48.641*** | 22.848*** | 0.026 | 0.995 | 37.094 | 0.005 | **14**,425 |
| ENSAPLG00000011503 | CHUK | 0.132 | 0.056 | 1.007^NS^ | 0.104^NS^ | 0.042 | 0.997 | 5.584 | 0.003 |  |
| ENSAPLG00000011572 | AvBD8 | 0.661 | 0.337 | 6.490^NS^ | 0.669^NS^ | 0.089 | 0.853 | 2.252 | 0.147 |  |
| ENSAPLG00000011675 | PIK3R2 | 0.192 | 0.039 | 31.351*** | 0.793^NS^ | 0.013 | 0.983 | 1.873 | 0.017 |  |
| ENSAPLG00000011754 | IL6 | 0.093 | 0.062 | -0.001^NS^ | 0.001^NS^ | 0.062 | 1.000 | 1.000 | 0.000 |  |
| ENSAPLG00000011919 | AvBD13 | 0.104 | 0.374 | 4.014^NS^ | 1.652^NS^ | 0.000 | 0.915 | 5.579 | 0.085 |  |
| ENSAPLG00000011922 | AvBD12 | 0.566 | 0.184 | 41.568*** | 17.084*** | 0.065 | 0.949 | 11.452 | 0.051 | 60 |
| ENSAPLG00000011932 | AvBD10 | 0.405 | 0.097 | 2.214^NS^ | 0.000^NS^ | 0.013 | 0.901 | 1.000 | 0.027 |  |
| ENSAPLG00000011933 | TRAF3 | 0.079 | 0.017 | 0.000^NS^ | 0.007^NS^ | 0.017 | 1.000 | 1.000 | 0.000 |  |
| ENSAPLG00000012047 | JAK1 | 0.177 | 0.039 | 31.134*** | 5.705^NS^ | 0.000 | 0.984 | 3.113 | 0.016 |  |
| ENSAPLG00000012056 | AvBD9 | 0.440 | 1.470 | 51.082*** | 32.317*** | 0.000 | 0.849 | 16.348 | 0.139 | **29**,**35** |
| ENSAPLG00000012088 | SOCS3 | 0.096 | 0.011 | -0.001^NS^ | 0.001^NS^ | 0.011 | 1.000 | 146.476 | 0.000 |  |
| ENSAPLG00000012239 | AvBD7 | 0.783 | 0.546 | 64.530*** | 30.463*** | 0.000 | 0.666 | 32.001 | 0.021 | 65 |
| ENSAPLG00000012251 | AvBD2 | 0.515 | 0.386 | 10.021^NS^ | 1.836^NS^ | 0.102 | 0.871 | 3.033 | 0.129 |  |
| ENSAPLG00000012287 | AvBD1 | 0.472 | 1.231 | 30.351*** | 30.572*** | 0.000 | 0.783 | 7.663 | 0.217 | **26**,*42*,48 |
| ENSAPLG00000012383 | CCL4 b | 0.022 | 999.000^b^ | 0.000^NS^ | 1.421^NS^ | 0.000 | 0.000 | 999.000 | 1.000 |  |
| ENSAPLG00000012465 | AvBD5 | 0.341 | 0.122 | 0.000^NS^ | 0.000^NS^ | 0.122 | 1.000 | 1.000 | 0.000 |  |
| ENSAPLG00000012580 | AvBD14 | 0.332 | 0.200 | 0.501^NS^ | -1.118^NS^ | 0.000 | 0.755 | 1.000 | 0.103 |  |
| ENSAPLG00000012625 | TLR4 | 0.176 | 0.397 | 25.216*** | 5.593^NS^ | 0.067 | 0.856 | 2.661 | 0.124 |  |
| ENSAPLG00000012692 | IL8-like b | 0.219 | 0.687 | 16.240** | 9.921* | 0.289 | 0.965 | 19.314 | 0.035 | *2*,**21** |
| ENSAPLG00000012697 | IL8-like a | 0.268 | 0.173 | 2.481^NS^ | 0.734^NS^ | 0.130 | 0.989 | 8.841 | 0.011 |  |
| ENSAPLG00000012752 | IRF7 | 0.518 | 0.136 | 86.528*** | 0.000^NS^ | 0.022 | 0.869 | 1.000 | 0.072 |  |
| ENSAPLG00000012768 | CTSK | 0.267 | 0.051 | 13.678* | 0.689^NS^ | 0.027 | 0.986 | 2.465 | 0.014 |  |
| ENSAPLG00000012773 | CCL4 a | 0.259 | 0.156 | 2.054^NS^ | 0.177^NS^ | 0.098 | 0.973 | 3.344 | 0.027 |  |
| ENSAPLG00000012876 | IFNG | 0.202 | 0.320 | 2.901^NS^ | 0.000^NS^ | 0.000 | 0.691 | 12.838 | 0.000 |  |
| ENSAPLG00000012924 | PIK3CA isoform 1 | 0.107 | 0.090 | 67.064*** | 52.249*** | 0.000 | 0.917 | 93.823 | 0.003 | 77,*104*,107 |
| ENSAPLG00000013226 | STAT1 b | 0.130 | 0.061 | 11.308* | 1.108^NS^ | 0.000 | 0.972 | 2.280 | 0.028 |  |
| ENSAPLG00000013262 | STAT1 a | 0.171 | 0.016 | 0.000^NS^ | 0.000^NS^ | 0.016 | 1.000 | 1.000 | 0.000 |  |
| ENSAPLG00000013460 | MAPK9 | 0.078 | 0.012 | 0.004^NS^ | 0.000^NS^ | 0.000 | 0.987 | 1.000 | 0.010 |  |
| ENSAPLG00000013488 | TRAF6 | 0.176 | 0.067 | 34.668*** | 7.571^NS^ | 0.023 | 0.993 | 10.257 | 0.007 |  |
| ENSAPLG00000013556 | CASP1 | 0.410 | 0.285 | 43.743*** | 6.306^NS^ | 0.054 | 0.769 | 8.411 | 0.009 |  |
| ENSAPLG00000013558 | CD40 | 0.299 | 0.316 | 25.665*** | 7.565^NS^ | 0.097 | 0.828 | 9.642 | 0.021 |  |
| ENSAPLG00000013878 | NFKB1 | 0.213 | 0.112 | 55.738*** | 12.212** | 0.020 | 0.925 | 11.716 | 0.004 | **829** |
| ENSAPLG00000014359 | TICAM1 | 0.472 | 0.614 | 127.471*** | 61.029*** | 0.000 | 0.535 | 11.197 | 0.024 | 252,**277**,**289**,**292**,**367**,**466**,***576***,**649** |
| ENSAPLG00000014394 | TNFRSF6B | 0.254 | 0.072 | 10.162^NS^ | -0.003^NS^ | 0.033 | 0.948 | 1.000 | 0.015 |  |
| ENSAPLG00000014631 | TMEM173 | 0.363 | 0.145 | 27.242*** | 0.000^NS^ | 0.017 | 0.846 | 1.000 | 0.054 |  |
| ENSAPLG00000014726 | TLR21 | 0.273 | 0.142 | 165.638*** | 24.421*** | 0.000 | 0.885 | 8.224 | 0.010 | **7**,*19*,*62*,**68** |
| ENSAPLG00000014872 | CASP8 | 0.182 | 0.215 | 0.396^NS^ | 0.000^NS^ | 0.158 | 0.927 | 1.000 | 0.072 |  |
| ENSAPLG00000014996 | IFITM3 | 0.695 | 0.553 | 56.140*** | 27.668*** | 0.000 | 0.560 | 12.436 | 0.044 | *84*,**85** |
| ENSAPLG00000015091 | DDX58 | 0.285 | 0.332 | 25.811*** | 2.856^NS^ | 0.070 | 0.742 | 6.022 | 0.008 |  |
| ENSAPLG00000015131 | TAB1 | 0.246 | 0.014 | 0.000^NS^ | 0.001^NS^ | 0.014 | 1.000 | 1.000 | 0.000 |  |
| ENSAPLG00000015244 | CASP10 | 0.162 | 0.399 | 5.355^NS^ | 0.303^NS^ | 0.000 | 0.692 | 1.306 | 0.308 |  |
| ENSAPLG00000015420 | RIPK1 | 0.308 | 0.247 | 62.494*** | 14.409** | 0.124 | 0.965 | 4.912 | 0.034 | **405**,**446** |
| ENSAPLG00000015480 | MAP2K1 | 0.103 | 0.000 | 2.621^NS^ | 3.872^NS^ | 0.000 | 1.000 | 3.806 | 0.000 |  |
| ENSAPLG00000015976 | Mx | 0.316 | 0.655 | 68.652*** | 26.008*** | 0.068 | 0.795 | 3.148 | 0.205 | *11*,44,**63**,*691*,*717* |
| ENSAPLG00000016065 | PIK3R5 | 0.319 | 0.108 | 172.589*** | 28.929*** | 0.027 | 0.964 | 4.239 | 0.023 | 244,**250**,*569*,**843** |
| ENSAPLG00000016230 | TRAF2 | 0.120 | 0.137 | 0.000^NS^ | 0.000^NS^ | 0.137 | 1.000 | 1.000 | 0.000 |  |
| ENSAPLG00000016235 | AKT2 | 0.372 | 0.038 | 58.020*** | 0.354^NS^ | 0.006 | 0.970 | 1.750 | 0.019 |  |
| ENSAPLG00000016448 | MAPK8 | 0.045 | 0.000 | 0.009^NS^ | 0.001^NS^ | 0.000 | 1.000 | 22.039 | 0.000 |  |

Likelihood ratio tests (LRT) were performed by calculating double the difference in log likelihood values between the alternative model (M2a or M8) and the null model (M1 or M7). FDR-adjusted *P* values were obtained from the chi-squared distribution with 2 degrees of freedom (calculated as the difference between the numbers of parameters included in each model). Comparisons with FDR-adjusted *p* values < 0.05 were considered to be statistically significant.

*FDR p < 0.05; **FDR p < 0.01; ***FDR p < 0.001; ^NS^FDR p > 0.05.

^a^ Only sites with a posterior probability higher than 95% are reported. Sites in bold were also detected as positively selected in at least two tests in HyPhy, sites in italic was also detected as positively selected in one test in HyPhy. Numbering of amino acid sites is based on *Anas platyrhynchos*.

^b^ ENSAPLG00000012383, CCL4 b had no synonymous differences which means that the w ratio will be estimated to be infinity. This gene was there excluded from Figure 2F.

^c^ CCL24= CCL24orCCL4

#### Table S16 Site specific selection as calculated using HyPhy

| Ensembl ID | Gene ID | Selection pattern | FUBAR | FEL | SLAC | MEME | Duplicated codons | No. duplicated codons | Proportions of codons under selection |
| --- | --- | --- | --- | --- | --- | --- | --- | --- | --- |
| ENSAPLG00000000983 | cathelicidinlike | Neg |  |  |  |  |  | 0 | 0 |
| ENSAPLG00000000983 | cathelicidinlike | Pos | 4 6 | 4 6 | 6 | 4 6 | **4 6** | 2 | 0.037 |
| ENSAPLG00000001279 | TLR5 | Neg |  | 46 95 99 143 157 166 308 186 225 232 276 277 354 360 385 394 403 425 431 462 472 492 583 595 600 620 693 736 752 765 774 801 | 354 583 693 736 752 765 801 |  | 354 583 693 736 752 765 801 | 7 | 0.008 |
| ENSAPLG00000001279 | TLR5 | Pos | 21 **33** 63 **72** 73 104 **106** 123 **128** 178 204 **205** **235** 244 **279** 307 **339** 340 **392** 506 508 **532** 559 591 **677** 816 | 73 104 123 506 |  | 73 104 340 459 530 | **73 104 123 506** 340 | 5 | 0.006 |
| ENSAPLG00000001297 | AvBD4 | Neg |  | 2 | 2 |  | 2 | 1 | 0.023 |
| ENSAPLG00000001297 | AvBD4 | Pos | **4** **29** 39 | 39 | 39 | 39 | **39** | 1 | 0.023 |
| ENSAPLG00000001534 | PML | Neg |  | 2 11 25 36 38 43 58 60 62 73 83 107 115 120 126 161 177 178 179 182 183 190 196 | 2 11 25 38 62 73 190 |  | 2 11 25 38 62 73 190 | 7 | 0.035 |
| ENSAPLG00000001534 | PML | Pos | 91 |  | 91 |  | 91 | 1 | 0.005 |
| ENSAPLG00000001925 | TIRAP | Neg |  | 56 60 65 88 95 102 107 120 136 144 155 163 181 191 208 229 | 102 136 229 |  | 102 136 229 | 3 | 0.013 |
| ENSAPLG00000001925 | TIRAP | Pos | 70 72 80 | 80 |  | 6 80 | 80 | 1 | 0.004 |
| ENSAPLG00000002012 | IFNGR1 | Neg |  | 31 35 103 112 136 168 186 199 221 244 248 298 355 365 | 199 212 355 |  | 199 355 | 2 | 0.005 |
| ENSAPLG00000002012 | IFNGR1 | Pos | **28** 42 154 158 | 158 |  | 42 158 191 226 318 | **158** 42 | 2 | 0.005 |
| ENSAPLG00000002049 | TLR1-A | Neg |  | 28 41 90 159 220 222 254 258 307 349 356 374 445 452 467 478 501 518 520 524 527 570 584 586 608 734 735 748 749 774 775 792 | 307 390 434 452 467 478 489 518 520 524 543 570 651 728 737 748 749 774 775 |  | 307 452 467 478 518 520 524 570 748 749 774 775 | 12 | 0.015 |
| ENSAPLG00000002049 | TLR1-A | Pos | 334 470 516 522 526 | 516 |  | 516 522 526 | **516 522 526** | 3 | 0.004 |
| ENSAPLG00000002168 | MAPK13 | Neg |  | 28 46 47 59 65 69 98 167 169 225 266 319 347 | 28 46 55 65 167 169 266 305 |  | 28 46 65 167 169 266 | 6 | 0.017 |
| ENSAPLG00000002168 | MAPK13 | Pos |  |  |  |  |  | 0 | 0 |
| ENSAPLG00000002299 | LITAF | Neg |  | 9 10 22 23 58 87 92 107 117 | 87 92 |  | 87 92 | 2 | 0.013 |
| ENSAPLG00000002299 | LITAF | Pos | 140 |  |  |  |  | 0 | 0 |
| ENSAPLG00000002603 | MAPK14 | Neg |  | 7 34 111 220 261 266 270 287 288 | 34 87 261 |  | 34 261 | 2 | 0.006 |
| ENSAPLG00000002603 | MAPK14 | Pos |  |  |  |  |  | 0 | 0 |
| ENSAPLG00000003004 | SPP1 | Neg |  | 46 77 89 106 110 123 124 127 156 160 195 204 215 217 233 254 264 | 46 77 89 123 124 195 215 233 264 |  | 46 77 89 123 124 195 215 233 264 | 9 | 0.033 |
| ENSAPLG00000003004 | SPP1 | Pos | 167 |  |  |  |  | 0 | 0 |
| ENSAPLG00000003319 | MAVS | Neg |  | 37 40 57 136 60 70 80 116 118 127 132 140 144 154 172 175 182 189 192 206 208 209 217 243 254 264 268 281 285 292 293 298 299 300 304 311 329 339 342 351 355 356 357 360 367 368 384 394 395 401 411 415 419 424 436 437 447 452 453 454 468 475 479 482 488 491 502 505 506 513 516 524 533 535 542 546 549 550 570 576 582 606 618 622 | 16 37 40 70 92 118 122 127 153 154 162 168 175 189 192 206 217 228 254 268 285 292 298 299 300 318 355 360 384 394 401 437 452 453 479 482 496 505 513 516 524 533 535 537 545 549 550 618 |  | 37 40 70 118 127 154 175 189 192 206 217 254 268 285 292 298 299 300 355 360 384 394 401 437 452 453 479 482 505 513 516 524 533 535 549 550 618 | 37 | 0.059 |
| ENSAPLG00000003319 | MAVS | Pos | 46 101 121 **210** 211 223 239 324 375 386 418 444 450 480 489 492 495 514 520 525 563 | 101 223 239 375 386 444 495 520 | 1 163 202 255 375 386 405 450 525 | 46 204 211 239 324 375 386 418 444 450 495 520 561 | 101 223 **239 375** 386 444 495 520 450 525 46 211 324 418 | 14 | 0.022 |
| ENSAPLG00000003430 | IFN | Neg |  | 97 |  |  |  | 0 | 0 |
| ENSAPLG00000003430 | IFN | Pos | 23 50 |  |  |  |  | 0 | 0 |
| ENSAPLG00000003554 | PIK3CB | Neg |  | 9 32 81 97 107 144 154 169 190 211 218 256 280 283 316 327 328 392 809 400 447 556 621 634 657 775 934 781 876 900 903 945 962 997 1062 | 154 211 256 316 447 621 903 |  | 154 211 256 316 447 621 903 | 7 | 0.007 |
| ENSAPLG00000003554 | PIK3CB | Pos | 382 |  |  |  |  | 0 | 0 |
| ENSAPLG00000003568 | RAC1 | Neg |  | 24 62 106 153 | 24 153 |  | 24 153 | 2 | 0.011 |
| ENSAPLG00000003568 | RAC1 | Pos |  |  |  |  |  | 0 | 0 |
| ENSAPLG00000004139 | TLR7 | Neg |  | 17 35 49 59 61 81 86 93 97 101 125 128 189 236 273 340 354 385 401 637 478 905 486 503 772 504 560 614 644 650 651 698 734 780 812 843 880 881 892 901 904 910 946 992 1000 1003 1024 | 77 86 128 561 573 644 651 901 992 1017 |  | 86 128 644 651 901 992 | 6 | 0.006 |
| ENSAPLG00000004139 | TLR7 | Pos | 1 279 367 387 **415** 483 | 279 483 |  | 1 279 367 483 | **279 483** 1 367 | 4 | 0.004 |
| ENSAPLG00000004157 | MAPK12 | Neg |  | 13 15 37 76 22 23 25 291 31 56 61 104 152 162 166 185 208 241 276 286 287 290 297 306 315 319 | 22 56 104 143 185 276 287 297 |  | 22 56 104 185 276 287 297 | 7 | 0.021 |
| ENSAPLG00000004157 | MAPK12 | Pos | 3 |  |  |  |  | 0 | 0 |
| ENSAPLG00000004462 | MAPK11 | Neg |  | 27 39 57 72 142 147 148 149 187 195 211 223 224 225 244 263 286 296 315 | 135 142 148 187 195 223 224 225 244 263 290 |  | 142 148 187 195 223 224 225 244 263 | 9 | 0.027 |
| ENSAPLG00000004462 | MAPK11 | Pos |  |  |  |  |  | 0 | 0 |
| ENSAPLG00000004748 | JAK2 | Neg |  | 45 53 771 116 182 190 191 230 260 297 377 443 448 455 490 491 519 534 621 639 646 674 696 713 739 751 754 785 803 822 831 861 896 937 940 987 999 1001 1044 | 260 674 721 751 754 896 936 937 940 |  | 260 674 751 754 896 937 940 | 7 | 0.006 |
| ENSAPLG00000004748 | JAK2 | Pos | 980 1070 |  |  | 312 1070 | 1070 | 1 | 0.001 |
| ENSAPLG00000004845 | LY96 | Neg |  | 23 81 126 |  |  |  | 0 | 0 |
| ENSAPLG00000004845 | LY96 | Pos | 87 |  |  | 87 | 87 | 1 | 0.006 |
| ENSAPLG00000004947 | TRIM25 | Neg |  | 33 55 57 81 92 104 105 112 118 124 125 127 141 148 153 202 222 266 303 383 395 418 438 463 464 524 525 542 556 557 560 574 582 | 81 105 112 125 127 202 266 303 383 390 395 463 524 525 |  | 81 105 112 125 127 202 266 303 383 395 463 524 525 | 13 | 0.022 |
| ENSAPLG00000004947 | TRIM25 | Pos | 350 363 | 363 | 49 | 256 350 363 393 | 363 350 | 2 | 0.003 |
| ENSAPLG00000005392 | TRADD | Neg |  | 18 26 29 34 72 138 153 178 186 190 245 279 282 293 294 295 | 18 138 178 245 258 279 |  | 18 138 178 245 279 | 5 | 0.016 |
| ENSAPLG00000005392 | TRADD | Pos | 188 196 |  |  |  |  | 0 | 0 |
| ENSAPLG00000005400 | CCL19 | Neg |  | 17 24 27 34 35 36 39 41 44 48 55 57 62 73 75 76 79 81 86 | 17 27 36 44 57 73 79 81 |  | 17 27 36 44 57 73 79 81 | 8 | 0.084 |
| ENSAPLG00000005400 | CCL19 | Pos |  |  |  | 11 90 |  | 0 | 0 |
| ENSAPLG00000005826 | MAP2K6 | Neg |  | 34 42 50 80 87 88 94 116 137 189 248 274 279 284 323 | 80 112 116 189 274 |  | 80 116 189 274 | 4 | 0.012 |
| ENSAPLG00000005826 | MAP2K6 | Pos |  |  |  |  |  | 0 | 0 |
| ENSAPLG00000005856 | MAPK10 | Neg |  | 103 104 122 150 157 195 222 273 315 322 344 349 360 376 | 104 222 273 |  | 104 222 273 | 3 | 0.008 |
| ENSAPLG00000005856 | MAPK10 | Pos |  |  |  |  |  | 0 | 0 |
| ENSAPLG00000005913 | CD86 | Neg |  | 11 36 71 135 158 165 183 233 271 | 11 |  | 11 | 1 | 0.004 |
| ENSAPLG00000005913 | CD86 | Pos | 57 113 136 |  |  | 113 | 113 | 1 | 0.004 |
| ENSAPLG00000005928 | JUN | Neg |  | 36 47 52 58 101 108 112 113 117 118 128 129 130 131 142 144 155 158 162 179 180 196 199 204 212 264 | 36 108 112 118 120 129 130 131 196 |  | 36 108 112 118 129 130 131 196 | 8 | 0.027 |
| ENSAPLG00000005928 | JUN | Pos |  |  |  |  |  | 0 | 0 |
| ENSAPLG00000005942 | IL18 | Neg |  | 53 101 119 | 53 101 119 |  | 53 101 119 | 3 | 0.015 |
| ENSAPLG00000005942 | IL18 | Pos | 8 77 |  |  |  |  | 0 | 0 |
| ENSAPLG00000006016 | RSAD2 | Neg |  | 15 18 46 47 62 69 75 80 83 86 92 99 119 130 133 142 154 181 183 214 218 221 247 255 257 283 291 297 | 62 130 133 142 183 214 218 221 257 297 |  | 62 130 133 142 183 214 218 221 257 297 | 10 | 0.033 |
| ENSAPLG00000006016 | RSAD2 | Pos | **2** |  |  |  |  | 0 | 0 |
| ENSAPLG00000006116 | MAPK1 | Neg |  | 23 95 113 128 272 |  |  |  | 0 | 0 |
| ENSAPLG00000006116 | MAPK1 | Pos |  |  |  |  |  | 0 | 0 |
| ENSAPLG00000006147 | IL12A | Neg |  | 1 12 21 48 58 59 61 64 99 100 102 105 121 129 141 | 1 12 21 48 58 59 61 105 127 164 |  | 1 12 21 48 58 59 61 105 | 8 | 0.048 |
| ENSAPLG00000006147 | IL12A | Pos | 2 14 167 | 167 | 167 | 167 | 167 | 1 | 0.006 |
| ENSAPLG00000006203 | IRF5 | Neg | 2 5 97 103 104 117 126 134 138 170 175 180 187 222 238 249 250 252 279 284 285 291 292 295 300 314 316 317 319 320 | 5 10 14 22 51 53 65 87 97 103 104 106 111 112 116 117 119 126 134 138 142 169 170 172 175 180 187 190 222 236 238 249 250 252 258 279 284 285 291 292 295 300 308 314 316 317 319 320 335 336 368 | 103 104 126 134 175 181 222 232 249 250 252 279 284 285 295 314 316 317 320 |  | 5 97 103 104 117 126 134 138 170 175 180 187 222 238 249 250 252 279 284 285 291 292 295 300 314 316 317 319 320 | 29 | 0.078 |
| ENSAPLG00000006203 | IRF5 | Pos | 1 128 135 140 151 **155** 159 **173** 179 198 200 202 **206** 331 350 | 1 159 | 1 159 | 1 37 159 **181** 193 198 | **1** **159** **198** | 3 | 0.008 |
| ENSAPLG00000006379 | IKBKB | Neg |  | 31 53 374 38 55 64 91 102 155 194 197 629 239 258 274 433 286 289 337 340 437 457 483 508 515 539 560 600 665 670 | 38 91 515 |  | 38 91 515 | 3 | 0.004 |
| ENSAPLG00000006379 | IKBKB | Pos |  |  |  |  |  | 0 | 0 |
| ENSAPLG00000006669 | PIK3CG | Neg |  | 46 48 79 100 110 136 159 164 172 182 190 209 225 733 837 229 240 253 263 281 286 294 324 330 336 353 356 395 399 404 428 438 445 450 459 491 501 511 512 520 566 572 576 581 636 639 647 657 713 716 759 767 795 824 827 838 862 869 882 904 906 916 918 950 960 966 975 992 1025 1044 1048 1051 | 79 100 136 164 172 209 286 336 356 395 428 445 450 501 511 512 521 656 713 716 759 824 827 882 906 918 950 956 960 966 992 1051 |  | 79 100 136 164 172 209 286 336 356 395 428 445 450 501 511 512 713 716 759 824 827 882 906 918 950 960 966 992 1051 | 29 | 0.026 |
| ENSAPLG00000006669 | PIK3CG | Pos |  |  |  |  |  | 0 | 0 |
| ENSAPLG00000007148 | MAP2K3 | Neg |  | 31 36 47 52 70 78 81 86 99 115 120 133 165 180 187 190 200 202 205 207 212 213 219 220 224 234 244 252 260 286 297 298 | 70 180 187 200 202 207 212 213 219 220 286 |  | 70 180 187 200 202 207 212 213 219 220 286 | 11 | 0.037 |
| ENSAPLG00000007148 | MAP2K3 | Pos |  |  |  |  |  | 0 | 0 |
| ENSAPLG00000007417 | CD80 | Neg |  | 40 46 41 54 72 108 128 133 139 191 205 211 291 300 | 113 128 133 291 300 |  | 128 133 291 300 | 4 | 0.013 |
| ENSAPLG00000007417 | CD80 | Pos | 24 92 292 |  | 19 |  |  | 0 | 0 |
| ENSAPLG00000007876 | TANK | Neg |  | 182 333 365 380 |  |  |  | 0 | 0 |
| ENSAPLG00000007876 | TANK | Pos | 126 |  |  |  |  | 0 | 0 |
| ENSAPLG00000008173 | TAB2 | Neg |  | 53 92 98 104 146 211 374 406 505 534 | 92 98 |  | 92 98 | 2 | 0.003 |
| ENSAPLG00000008173 | TAB2 | Pos | **605** |  |  |  |  | 0 | 0 |
| ENSAPLG00000008183 | PIK3CD | Neg |  | 17 364 51 57 58 71 88 95 101 126 147 184 195 234 277 304 305 349 380 398 401 426 434 450 461 465 477 486 495 595 649 778 807 837 852 869 875 884 886 895 936 971 999 1007 1021 | 58 71 88 95 126 304 305 426 434 450 465 486 518 589 595 807 837 856 875 |  | 58 71 88 95 126 304 305 426 434 450 465 486 595 807 837 875 | 16 | 0.015 |
| ENSAPLG00000008183 | PIK3CD | Pos | 214 |  |  |  |  | 0 | 0 |
| ENSAPLG00000008445 | TBK1 | Neg |  | 5 64 90 138 145 153 158 179 187 192 214 252 266 281 299 305 315 346 347 351 353 365 380 381 386 389 390 419 424 443 469 506 517 560 571 576 577 639 644 648 650 652 659 703 710 719 | 64 145 153 158 214 281 315 353 380 389 424 506 517 648 652 699 719 |  | 64 145 153 158 214 281 315 353 380 389 424 506 517 648 652 719 | 16 | 0.022 |
| ENSAPLG00000008445 | TBK1 | Pos | 610 |  |  |  |  | 0 | 0 |
| ENSAPLG00000008655 | LBP | Neg |  | 9 12 13 30 61 75 88 92 121 133 178 219 222 231 244 249 258 268 272 293 296 330 339 | 5 12 13 26 29 30 55 66 88 182 219 222 224 249 293 296 308 330 |  | 12 13 30 88 219 222 249 293 296 330 | 10 | 0.028 |
| ENSAPLG00000008655 | LBP | Pos | 11 60 69 72 **77** 281 299 **356** |  | 10 72 76 220 297 | 11 69 72 | **72** 11 69 | 3 | 0.008 |
| ENSAPLG00000008976 | TLR3 | Neg |  | 29 282 75 86 121 144 246 248 267 295 397 430 435 439 468 472 653 658 660 693 697 712 723 752 759 760 769 770 784 809 882 | 121 246 295 430 657 658 693 745 759 809 |  | 121 246 295 430 658 693 759 809 | 8 | 0.009 |
| ENSAPLG00000008976 | TLR3 | Pos | 243 313 423 885 |  |  | 170 515 |  | 0 | 0 |
| ENSAPLG00000009092 | IL12B | Neg |  | 23 77 27 79 98 134 168 | 98 168 |  | 98 168 | 2 | 0.006 |
| ENSAPLG00000009092 | IL12B | Pos |  |  |  |  |  | 0 | 0 |
| ENSAPLG00000009156 | TOLLIP | Neg |  | 108 145 155 230 231 261 | 230 231 |  | 230 231 | 2 | 0.007 |
| ENSAPLG00000009156 | TOLLIP | Pos |  |  |  |  |  | 0 | 0 |
| ENSAPLG00000009205 | MAP3K8 | Neg |  | 78 95 96 120 134 151 160 168 182 190 211 237 243 250 255 279 281 313 322 338 339 345 375 378 388 399 433 465 467 470 | 78 96 120 160 182 203 250 255 313 322 338 339 345 375 378 388 433 470 |  | 78 96 120 160 182 250 255 313 322 338 339 345 375 378 388 433 470 | 17 | 0.036 |
| ENSAPLG00000009205 | MAP3K8 | Pos |  |  |  |  |  | 0 | 0 |
| ENSAPLG00000009391 | CCL24 isoform 1 ^a^ | Neg |  | 2 53 |  |  |  | 0 | 0 |
| ENSAPLG00000009391 | CCL24 isoform 2 ^a^ | Neg |  | 8 | 8 |  | 8 | 1 | 0.011 |
| ENSAPLG00000009391 | CCL24 isoform 1 ^a^ | Pos | **47** |  |  |  |  | 0 | 0 |
| ENSAPLG00000009391 | CCL24 isoform2 ^a^ | Pos | 13 **48** | 13 |  |  | **13** | 1 | 0.011 |
| ENSAPLG00000009838 | CCL5 | Neg |  | 36 39 |  |  |  | 0 | 0 |
| ENSAPLG00000009838 | CCL5 | Pos | 89 |  |  |  |  | 0 | 0 |
| ENSAPLG00000009929 | TLR15 | Neg |  | 32 39 65 68 84 101 132 162 178 179 182 196 205 261 309 316 336 350 373 379 413 436 511 524 558 585 607 733 781 810 832 | 32 196 205 379 436 558 596 810 |  | 32 196 205 379 436 558 810 | 7 | 0.008 |
| ENSAPLG00000009929 | TLR15 | Pos | 21 170 256 554 |  |  | 21 22 419 736 | 21 | 1 | 0.001 |
| ENSAPLG00000010090 | NLRP12 | Neg |  | 1 9 54 71 91 111 131 132 145 192 204 217 223 225 232 243 254 255 291 309 322 333 369 384 394 400 419 434 455 465 469 477 504 510 515 554 557 571 578 586 594 595 602 603 604 619 620 631 642 647 648 652 667 668 687 714 735 752 754 819 821 823 827 | 1 71 74 111 223 322 465 469 477 504 594 604 619 631 647 667 821 827 |  | 1 71 111 223 322 465 469 477 504 594 604 619 631 647 667 821 827 | 17 | 0.02 |
| ENSAPLG00000010090 | NLRP12 | Pos | 37 43 142 196 299 437 475 541 635 644 728 | 37 437 635 |  | 437 475 478 635 | 37 437 635 475 | 4 | 0.005 |
| ENSAPLG00000010194 | MYD88 | Neg |  | 92 122 123 132 | 122 123 132 |  | 122 123 132 | 3 | 0.022 |
| ENSAPLG00000010194 | MYD88 | Pos |  |  |  |  |  | 0 | 0 |
| ENSAPLG00000011028 | NFKBIA | Neg |  | 11 53 62 83 86 109 112 126 140 145 155 166 169 184 224 230 | 109 145 |  | 109 145 | 2 | 0.008 |
| ENSAPLG00000011028 | NFKBIA | Pos |  |  |  |  |  | 0 | 0 |
| ENSAPLG00000011045 | MAP2K2 | Neg |  | 21 32 37 39 58 238 244 259 266 278 | 21 238 244 259 |  | 21 238 244 259 | 4 | 0.013 |
| ENSAPLG00000011045 | MAP2K2 | Pos |  |  |  |  |  | 0 | 0 |
| ENSAPLG00000011284 | FOS | Neg |  | 17 19 27 57 74 76 83 91 92 94 96 102 103 106 111 118 123 124 130 133 136 138 139 140 143 150 156 158 159 173 180 184 196 207 220 233 253 | 17 42 76 92 102 111 118 136 143 159 173 184 255 |  | 17 76 92 102 111 118 136 143 159 173 184 | 11 | 0.042 |
| ENSAPLG00000011284 | FOS | Pos | 256 |  |  | 6 |  | 0 | 0 |
| ENSAPLG00000011364 | IFNAR2 | Neg |  | 26 28 64 113 146 157 160 180 182 188 197 211 221 407 244 305 311 319 324 325 327 349 416 448 451 | 180 244 |  | 180 244 | 2 | 0.004 |
| ENSAPLG00000011364 | IFNAR2 | Pos | **29** 33 40 **46 51** 71 73 76 96 99 101 102 104 106 108 120 122 126 **128 130** **149** 175 **187** 189 223 225 **278** 464 | 71 73 76 99 101 102 108 120 126 225 |  | 13 18 33 37 40 71 73 76 99 101 102 104 106 120 122 126 189 364 389 464 | **71 73 76 99 101 102 108** **120 126** **225** **33 40** **104 106** **122** **189** 464 | 17 | 0.033 |
| ENSAPLG00000011397 | TLR2 | Neg |  | 57 74 79 141 146 167 227 232 240 284 298 315 404 424 459 468 478 501 502 544 559 562 566 571 615 630 640 642 643 653 655 664 665 678 697 700 702 708 718 730 735 736 739 | 203 258 459 468 478 502 562 566 615 640 642 643 655 |  | 459 468 478 502 562 566 615 640 642 643 655 | 11 | 0.014 |
| ENSAPLG00000011397 | TLR2 | Pos | 44 300 306 307 310 333 **452** 456 504 561 **568** 585 594 605 633 | 300 306 307 310 594 605 633 | 306 307 633 | 87 306 307 310 **454** 585 594 633 | **300 306 307 310** **594** **605** **633** **585** | 8 | 0.01 |
| ENSAPLG00000011399 | TLR2a | Neg |  | 12 32 35 50 62 65 72 378 96 130 139 155 161 192 196 220 225 313 344 345 348 443 451 476 493 494 536 551 554 576 607 620 622 647 656 675 692 700 710 714 722 723 727 728 776 | 50 130 139 192 196 451 460 494 554 607 622 647 656 727 |  | 50 130 139 192 196 451 494 554 607 622 647 656 727 | 13 | 0.017 |
| ENSAPLG00000011399 | TLR2a | Pos | 37 **219** 277 **363** 389 597 603 775 | 597 603 | 603 | 597 603 775 | **597 603** 775 | 3 | 0.004 |
| ENSAPLG00000011477 | AKT3 | Neg |  | 71 81 105 169 174 193 325 345 418 | 325 |  | 325 | 1 | 0.002 |
| ENSAPLG00000011477 | AKT3 | Pos | 14 |  |  | 14 | **14** | 1 | 0.002 |
| ENSAPLG00000011503 | CHUK | Neg |  | 25 80 93 123 127 199 211 216 235 250 312 358 473 481 568 592 632 635 641 643 644 648 692 | 123 250 481 632 635 648 |  | 123 250 481 632 635 648 | 6 | 0.009 |
| ENSAPLG00000011503 | CHUK | Pos |  |  | 1 |  |  | 0 | 0 |
| ENSAPLG00000011572 | AvBD8 | Neg |  | 2 6 22 | 2 6 7 |  | 2 6 | 2 | 0.067 |
| ENSAPLG00000011572 | AvBD8 | Pos | 13 |  |  |  |  | 0 | 0 |
| ENSAPLG00000011675 | PIK3R2 | Neg |  | 8 9 21 26 38 42 44 57 61 66 72 83 85 96 97 100 126 131 135 136 164 174 179 189 190 194 196 210 235 269 314 315 326 327 339 342 347 349 355 356 357 376 378 382 384 389 393 396 399 427 468 476 478 486 498 505 514 518 535 540 543 550 591 616 617 623 627 636 638 654 669 670 706 728 | 9 26 89 180 314 339 347 355 384 468 476 478 486 492 530 531 591 675 |  | 9 26 314 339 347 355 384 468 476 478 486 591 | 12 | 0.016 |
| ENSAPLG00000011675 | PIK3R2 | Pos | 24 108 109 211 |  | 106 | 24 | 24 | 1 | 0.001 |
| ENSAPLG00000011754 | IL6 | Neg |  | 34 51 65 86 |  |  |  | 0 | 0 |
| ENSAPLG00000011754 | IL6 | Pos |  |  |  |  |  | 0 | 0 |
| ENSAPLG00000011919 | AvBD13 | Neg |  |  |  |  |  | 0 | 0 |
| ENSAPLG00000011919 | AvBD13 | Pos |  |  | 32 33 34 |  |  | 0 | 0 |
| ENSAPLG00000011922 | AvBD12 | Neg |  | 5 9 14 18 20 22 33 40 50 | 14 20 33 |  | 14 20 33 | 3 | 0.045 |
| ENSAPLG00000011922 | AvBD12 | Pos |  |  |  |  |  | 0 | 0 |
| ENSAPLG00000011932 | AvBD10 | Neg |  | 29 31 48 49 51 53 63 65 | 31 49 |  | 31 49 | 2 | 0.029 |
| ENSAPLG00000011932 | AvBD10 | Pos |  |  |  |  |  | 0 | 0 |
| ENSAPLG00000011933 | TRAF3 | Neg |  | 7 22 26 36 42 47 83 88 141 142 164 205 208 239 324 407 460 530 | 83 141 530 |  | 83 141 530 | 3 | 0.005 |
| ENSAPLG00000011933 | TRAF3 | Pos |  |  |  |  |  | 0 | 0 |
| ENSAPLG00000012047 | JAK1 | Neg |  | 6 15 71 92 112 161 166 232 241 246 271 286 386 401 416 473 489 493 499 577 584 621 627 668 669 679 694 700 704 709 716 751 753 802 822 839 859 875 909 923 925 945 946 952 | 15 71 112 166 241 246 271 416 489 493 499 577 584 627 668 669 694 709 802 822 834 839 859 873 902 923 925 943 945 946 952 |  | 15 71 112 166 241 246 271 416 489 493 499 577 584 627 668 669 694 709 802 822 839 859 923 925 945 946 952 | 27 | 0.028 |
| ENSAPLG00000012047 | JAK1 | Pos | 815 |  |  | 1 |  | 0 | 0 |
| ENSAPLG00000012056 | AvBD9 | Neg |  | 3 28 54 |  |  |  | 0 | 0 |
| ENSAPLG00000012056 | AvBD9 | Pos | 29 35 | 29 35 | 35 | 29 35 | **29 35** | 2 | 0.031 |
| ENSAPLG00000012088 | SOCS3 | Neg |  | 22 35 64 71 99 147 180 187 188 | 7 22 29 |  | 22 | 1 | 0.005 |
| ENSAPLG00000012088 | SOCS3 | Pos |  |  |  |  |  | 0 | 0 |
| ENSAPLG00000012239 | AvBD7 | Neg |  | 6 15 39 53 63 65 | 39 |  | 39 | 1 | 0.015 |
| ENSAPLG00000012239 | AvBD7 | Pos |  |  |  | 54 |  | 0 | 0 |
| ENSAPLG00000012251 | AvBD2 | Neg |  | 8 38 50 | 8 38 50 |  | 8 38 50 | 3 | 0.051 |
| ENSAPLG00000012251 | AvBD2 | Pos |  |  |  |  |  | 0 | 0 |
| ENSAPLG00000012287 | AvBD1 | Neg |  | 9 16 25 | 8 |  |  | 0 | 0 |
| ENSAPLG00000012287 | AvBD1 | Pos | 14 26 **42** | 26 |  |  | **26** | 1 | 0.015 |
| ENSAPLG00000012383 | CCL4 | Neg |  |  |  |  |  | 0 | 0 |
| ENSAPLG00000012383 | CCL4 | Pos |  |  |  |  |  | 0 | 0 |
| ENSAPLG00000012465 | AvBD5 | Neg |  | 17 27 32 34 56 60 | 27 |  | 27 | 1 | 0.015 |
| ENSAPLG00000012465 | AvBD5 | Pos |  |  |  | 2 |  | 0 | 0 |
| ENSAPLG00000012580 | AvBD14 | Neg |  | 6 14 29 32 40 43 52 | 35 40 52 |  | 40 52 | 2 | 0.032 |
| ENSAPLG00000012580 | AvBD14 | Pos | 61 |  |  |  |  | 0 | 0 |
| ENSAPLG00000012625 | TLR4 | Neg |  | 6 7 54 187 188 196 219 247 305 317 383 457 424 439 454 472 473 505 534 539 544 588 598 602 672 683 700 717 753 772 799 800 803 | 6 7 187 219 305 439 454 472 505 544 588 598 683 698 753 799 803 |  | 6 7 187 219 305 439 454 472 505 544 588 598 683 753 799 803 | 16 | 0.019 |
| ENSAPLG00000012625 | TLR4 | Pos | 200 318 372 403 503 666 681 832 |  |  | 503 812 842 | 503 | 1 | 0.001 |
| ENSAPLG00000012692 | IL8-like | Neg |  | 47 64 103 |  |  |  | 0 | 0 |
| ENSAPLG00000012692 | IL8-like | Pos | **2** 5 21 | 21 |  | 5 | **21** 5 | 2 | 0.019 |
| ENSAPLG00000012697 | IL8-like | Neg |  | 22 37 40 52 56 76 87 102 | 52 56 |  | 52 56 | 2 | 0.019 |
| ENSAPLG00000012697 | IL8-like | Pos |  |  |  |  |  | 0 | 0 |
| ENSAPLG00000012752 | IRF7 | Neg |  | 4 17 24 35 44 45 48 52 64 67 91 97 99 108 116 117 123 143 153 162 175 195 216 257 270 276 291 335 | 4 44 45 48 67 78 99 123 162 257 270 |  | 4 44 45 48 67 99 123 162 257 270 | 10 | 0.029 |
| ENSAPLG00000012752 | IRF7 | Pos | 54 60 68 88 128 242 | 68 242 | 56 68 | 68 88 128 | 68 242 88 128 | 4 | 0.012 |
| ENSAPLG00000012768 | CTSK | Neg |  | 28 38 55 62 78 86 89 109 113 121 140 142 144 153 176 181 183 190 192 193 194 202 203 213 255 256 274 283 284 291 300 313 328 | 62 109 142 176 181 192 193 194 328 |  | 62 109 142 176 181 192 193 194 328 | 9 | 0.027 |
| ENSAPLG00000012768 | CTSK | Pos | 107 |  |  |  |  | 0 | 0 |
| ENSAPLG00000012773 | CCL4 | Neg |  | 15 47 49 76 86 88 | 47 49 76 |  | 47 49 76 | 3 | 0.032 |
| ENSAPLG00000012773 | CCL4 | Pos |  |  |  |  |  | 0 | 0 |
| ENSAPLG00000012876 | IFNG | Neg |  | 17 69 95 102 111 149 | 95 |  | 95 | 1 | 0.006 |
| ENSAPLG00000012876 | IFNG | Pos | 151 |  |  |  |  | 0 | 0 |
| ENSAPLG00000012924 | PIK3CA isoform 1 | Neg |  | 13 77 258 327 386 399 541 543 569 586 593 | 13 586 |  | 13 586 | 2 | 0.003 |
| ENSAPLG00000012924 | PIK3CA isoform 1 | Pos | **104** 114 |  |  |  |  | 0 | 0 |
| ENSAPLG00000013226 | STAT1 | Neg |  | 26 343 129 145 174 219 244 250 276 291 293 309 334 340 421 498 508 | 129 145 219 250 291 293 340 498 |  | 129 145 219 250 291 293 340 498 | 8 | 0.014 |
| ENSAPLG00000013226 | STAT1 | Pos | 151 419 |  |  |  |  | 0 | 0 |
| ENSAPLG00000013262 | STAT1 | Neg |  | 9 27 45 68 85 98 117 120 127 | 9 45 |  | 9 45 | 2 | 0.011 |
| ENSAPLG00000013262 | STAT1 | Pos |  |  |  |  |  | 0 | 0 |
| ENSAPLG00000013460 | MAPK9 | Neg |  | 96 105 164 173 193 244 262 269 271 378 | 96 173 244 269 |  | 96 173 244 269 | 4 | 0.009 |
| ENSAPLG00000013460 | MAPK9 | Pos |  |  |  |  |  | 0 | 0 |
| ENSAPLG00000013488 | TRAF6 | Neg |  | 5 7 16 17 28 37 38 39 40 60 77 79 107 115 148 156 195 378 203 238 239 250 255 309 318 322 351 358 419 436 454 470 497 499 | 17 38 39 74 77 148 309 470 |  | 17 38 39 77 148 309 470 | 7 | 0.013 |
| ENSAPLG00000013488 | TRAF6 | Pos | 103 137 |  |  | 103 137 | 103 137 | 2 | 0.004 |
| ENSAPLG00000013556 | CASP1 | Neg |  | 12 15 17 22 43 57 65 75 78 122 165 214 223 235 238 249 250 266 273 278 306 317 360 369 374 | 12 15 17 78 206 223 235 249 266 306 317 360 |  | 12 15 17 78 223 235 249 266 306 317 360 | 11 | 0.029 |
| ENSAPLG00000013556 | CASP1 | Pos | 267 269 296 300 | 267 296 |  | 267 296 | 267 296 | 2 | 0.005 |
| ENSAPLG00000013558 | CD40 | Neg |  | 13 16 25 46 58 74 83 90 135 136 164 183 202 | 46 |  | 46 | 1 | 0.006 |
| ENSAPLG00000013558 | CD40 | Pos | 100 130 132 214 218 | 214 218 |  | 214 218 | 214 218 | 2 | 0.011 |
| ENSAPLG00000013878 | NFKB1 | Neg |  | 16 38 50 75 112 125 151 191 201 232 262 266 359 383 398 407 409 422 427 444 525 543 558 581 582 592 604 645 649 669 691 692 704 714 720 721 750 766 781 786 793 799 807 809 830 843 846 854 879 916 920 922 925 929 941 962 973 | 16 38 48 75 112 151 191 383 398 444 493 525 548 558 582 645 649 674 692 714 750 766 781 843 849 916 922 929 |  | 16 38 75 112 151 191 383 398 444 525 558 582 645 649 692 714 750 766 781 843 916 922 929 | 23 | 0.023 |
| ENSAPLG00000013878 | NFKB1 | Pos | 458 782 829 | 782 |  | 829 | 782 **829** | 2 | 0.002 |
| ENSAPLG00000014359 | TICAM1 | Neg |  | 4 11 35 43 68 100 118 131 194 199 205 244 250 320 554 281 286 297 317 342 355 360 401 431 453 471 474 476 480 497 519 530 540 547 549 693 552 562 574 589 617 628 653 673 687 703 718 724 726 | 4 35 43 68 199 286 297 431 471 480 540 547 628 673 724 |  | 4 35 43 68 199 286 297 431 471 480 540 547 628 673 724 | 15 | 0.021 |
| ENSAPLG00000014359 | TICAM1 | Pos | 28 34 263 277 289 292 367 466 568 576 577 625 649 | 263 277 289 367 466 576 625 | 289 576 | 263 289 292 367 415 576 577 625 649 | 263 **277 289 367 466 576** 625 **292** 577 **649** | 10 | 0.014 |
| ENSAPLG00000014394 | TNFRSF6B | Neg |  | 7 8 24 39 63 70 90 119 122 129 141 181 195 205 210 213 232 236 241 244 263 265 270 | 7 129 195 |  | 7 129 195 | 3 | 0.011 |
| ENSAPLG00000014394 | TNFRSF6B | Pos |  |  |  |  |  | 0 | 0 |
| ENSAPLG00000014631 | TMEM173 | Neg |  | 2 8 9 11 15 17 18 20 25 27 34 38 39 45 55 59 71 80 87 114 119 123 129 134 150 158 181 196 226 264 271 279 307 310 346 351 357 365 370 373 | 25 27 80 150 196 357 373 |  | 25 27 80 150 196 357 373 | 7 | 0.019 |
| ENSAPLG00000014631 | TMEM173 | Pos | 54 |  | 44 | 136 |  | 0 | 0 |
| ENSAPLG00000014726 | TLR21 | Neg |  | 11 15 41 43 80 101 105 123 129 131 139 146 167 170 200 204 208 216 217 226 269 278 315 318 320 324 332 338 345 350 366 395 405 412 455 467 472 473 480 486 498 512 517 522 523 546 557 572 | 41 105 123 170 318 338 350 395 405 416 425 467 512 546 557 |  | 41 105 123 170 318 338 350 395 405 467 512 546 557 | 13 | 0.022 |
| ENSAPLG00000014726 | TLR21 | Pos | 7 **19** 36 39 49 **62** 68 76 96 104 | 39 68 | 7 | 39 68 | 39 **68 7** | 3 | 0.005 |
| ENSAPLG00000014872 | CASP8 | Neg |  | 5 33 35 47 56 62 64 103 113 115 122 230 262 265 266 275 346 391 393 397 413 454 461 465 470 | 413 461 |  | 413 461 | 2 | 0.004 |
| ENSAPLG00000014872 | CASP8 | Pos | 182 |  |  |  |  | 0 | 0 |
| ENSAPLG00000014996 | IFITM3 | Neg |  | 5 19 58 67 | 83 |  |  | 0 | 0 |
| ENSAPLG00000014996 | IFITM3 | Pos | **84** 85 | 85 | 85 | 85 | **85** | 1 | 0.011 |
| ENSAPLG00000015091 | DDX58 | Neg |  | 4 56 63 66 70 74 92 139 151 168 207 227 248 269 283 286 305 313 397 429 431 435 451 464 476 485 500 530 548 579 608 622 625 733 752 792 800 814 822 834 839 | 70 92 139 151 286 305 429 435 464 485 530 579 622 733 822 |  | 70 92 139 151 286 305 429 435 464 485 530 579 622 733 822 | 15 | 0.018 |
| ENSAPLG00000015091 | DDX58 | Pos | 421 694 695 | 421 |  | 422 442 | 421 | 1 | 0.001 |
| ENSAPLG00000015131 | TAB1 | Neg |  | 14 20 39 74 81 83 100 119 133 136 144 155 165 178 182 209 229 238 297 360 383 392 400 438 450 451 461 462 464 472 476 481 482 484 503 510 511 521 532 542 | 39 81 83 119 133 136 155 165 182 188 209 238 297 383 438 450 451 462 464 482 503 510 511 521 |  | 39 81 83 119 133 136 155 165 182 209 238 297 383 438 450 451 462 464 482 503 510 511 521 | 23 | 0.041 |
| ENSAPLG00000015131 | TAB1 | Pos |  |  |  |  |  | 0 | 0 |
| ENSAPLG00000015244 | CASP10 | Neg |  | 28 40 43 146 161 192 264 288 303 314 341 356 364 407 431 434 503 |  |  |  | 0 | 0 |
| ENSAPLG00000015244 | CASP10 | Pos | 220 334 413 420 |  |  |  |  | 0 | 0 |
| ENSAPLG00000015420 | RIPK1 | Neg |  | 24 61 117 146 157 185 188 207 225 229 252 308 318 331 368 371 373 385 387 425 436 536 460 490 585 617 636 648 654 | 24 41 61 117 146 191 207 229 252 318 331 373 387 401 425 475 617 648 654 |  | 24 61 117 146 207 229 252 318 331 373 387 425 617 648 654 | 15 | 0.022 |
| ENSAPLG00000015420 | RIPK1 | Pos | 82 360 405 446 573 | 82 405 446 |  | 82 405 446 | 82 **405 446** | 3 | 0.004 |
| ENSAPLG00000015480 | MAP2K1 | Neg |  | 13 92 96 97 100 107 119 200 236 242 258 259 303 312 | 13 96 97 104 107 |  | 13 96 97 107 | 4 | 0.011 |
| ENSAPLG00000015480 | MAP2K1 | Pos |  |  |  |  |  | 0 | 0 |
| ENSAPLG00000015976 | Mx | Neg |  | 36 44 62 108 135 162 217 218 230 279 313 322 343 345 354 362 403 420 441 460 509 514 521 584 591 618 666 675 700 714 | 44 139 162 178 230 614 666 700 714 |  | 44 162 230 666 700 714 | 6 | 0.008 |
| ENSAPLG00000015976 | Mx | Pos | **11** 46 52 63 320 428 462 565 626 **691** **717** | 63 |  | 63 485 604 | **63** | 1 | 0.001 |
| ENSAPLG00000016065 | PIK3R5 | Neg |  | 16 22 29 88 105 116 124 136 142 147 162 167 168 188 190 205 217 222 232 233 234 239 240 241 243 244 246 253 285 286 299 314 318 348 363 366 373 381 395 396 400 405 413 448 456 458 469 472 483 488 493 495 503 508 509 518 521 522 524 545 560 561 581 602 613 617 629 631 661 679 684 688 696 697 699 707 727 743 759 788 802 822 828 837 840 847 856 858 870 874 | 105 147 162 168 222 239 241 246 253 285 299 300 303 323 363 395 400 413 448 458 472 483 488 493 495 518 560 561 581 602 613 631 679 688 696 699 707 741 743 788 802 847 856 870 874 |  | 105 147 162 168 222 239 241 246 253 285 299 363 395 400 413 448 458 472 483 488 493 495 518 560 561 581 602 613 631 679 688 696 699 707 743 788 802 847 856 870 874 | 41 | 0.047 |
| ENSAPLG00000016065 | PIK3R5 | Pos | 229 250 252 **569** 842 843 | 250 843 |  | 229 454 843 | **250 843** 229 | 3 | 0.003 |
| ENSAPLG00000016230 | TRAF2 | Neg |  | 30 34 49 56 98 118 173 178 193 200 205 318 319 371 480 482 499 | 56 118 482 499 |  | 56 118 482 499 | 4 | 0.007 |
| ENSAPLG00000016230 | TRAF2 | Pos |  |  |  | 226 |  | 0 | 0 |
| ENSAPLG00000016235 | AKT2 | Neg |  | 21 22 27 51 72 74 99 105 106 110 113 116 118 122 139 147 157 158 165 166 167 176 180 188 192 193 208 218 231 250 251 255 268 269 275 286 287 288 293 294 300 303 | 21 27 39 51 74 75 106 113 116 122 139 218 269 288 294 301 303 |  | 21 27 51 74 106 113 116 122 139 218 269 288 294 303 | 14 | 0.044 |
| ENSAPLG00000016235 | AKT2 | Pos | 125 |  | 90 217 |  |  | 0 | 0 |
| ENSAPLG00000016448 | MAPK8 | Neg |  | 19 112 149 321 332 350 | 112 |  | 112 | 1 | 0.002 |
| ENSAPLG00000016448 | MAPK8 | Pos |  |  |  |  |  | 0 | 0 |

Numbering of amino acid sites is based on *Anas platyrhynchos*.

Sites in bold were also detected as positively selected in the PAML analysis.

^a^ CCL24 = CCL24orCCL4

#### Table S17. Sites under positive selection in PAML M1/M2 and/or at least two tests from HyPhy .

| Ensembl ID | Gene ID | Site under selection | Test | | | | |
| --- | --- | --- | --- | --- | --- | --- | --- |
|  |  |  | PAML | FUBAR | FEL | SLAC | MEME |
| ENSAPLG00000000983 | cathelicidinlike | 4 | x | x | x |  | x |
|  |  | 6 | x | x | x | x | x |
|  |  | 7 | x |  |  |  |  |
| ENSAPLG00000001279 | TLR5 | 33 | *x* | x |  |  |  |
|  |  | 72 | x | x |  |  |  |
|  |  | 73 | x | x | x |  | x |
|  |  | 104 | x | x | x |  | x |
|  |  | 106 | x | x |  |  |  |
|  |  | 123 | x | x | x |  |  |
|  |  | 128 | x | x |  |  |  |
|  |  | 205 | x | x |  |  |  |
|  |  | 235 | x | x |  |  |  |
|  |  | 279 | x | x |  |  |  |
|  |  | 339 | x | x |  |  |  |
|  |  | 392 | x | x |  |  |  |
|  |  | 506 | x | x | x |  |  |
|  |  | 532 | x | x |  |  |  |
|  |  | 677 | x | x |  |  |  |
| ENSAPLG00000001297 | AvBD4 | 1 | x |  |  |  |  |
|  |  | 4 | x | x |  |  |  |
|  |  | 7 | x |  |  |  |  |
|  |  | 29 | x | x |  |  |  |
|  |  | 39 | x | x | x | x | x |
| ENSAPLG00000001534 | PML | 91 |  | x |  | x |  |
| ENSAPLG00000001925 | TIRAP | 80 |  | x | x |  | x |
| ENSAPLG00000002012 | IFNGR1 | 28 | *x* | x |  |  |  |
|  |  | 140 | x |  |  |  |  |
|  |  | 158 | x | x | x |  | x |
| ENSAPLG00000002049 | TLR1A | 516 | x | x | x |  | x |
|  |  | 522 | x | x |  |  | x |
|  |  | 526 | x | x |  |  | x |
|  |  | 751 | x |  |  |  |  |
| ENSAPLG00000003319 | MAVS | 208 | x |  |  |  |  |
|  |  | 210 | x | x |  |  |  |
|  |  | 222 | x |  |  |  |  |
|  |  | 239 | x | x | x |  | x |
|  |  | 375 | x | x | x | x | x |
|  |  | 527 | x |  |  |  |  |
|  |  | 529 | x |  |  |  |  |
| ENSAPLG00000004139 | TLR7 | 279 | x | x | x |  | x |
| ENSAPLG00000004748 | JAK2 | 415 | x | x |  |  |  |
| ENSAPLG00000004845 | LY96 | 483 | x | x | x |  | x |
| ENSAPLG00000004947 | TRIM25 | 350 |  | x |  |  | x |
|  |  | 363 |  | x | x |  | x |
| ENSAPLG00000005913 | CD86 | 113 |  | x |  |  | x |
| ENSAPLG00000006016 | RSAD2 | 2 | *x* | x |  |  |  |
|  |  | 15 | x |  |  |  |  |
| ENSAPLG00000006147 | IL12A | 167 |  | x | x | x | x |
| ENSAPLG00000006203 | IRF5 | 1 | x | x | x | x | x |
|  |  | 155 | x | x |  |  |  |
|  |  | 159 | x | x | x | x | x |
|  |  | 173 | x | x |  |  |  |
|  |  | 181 | x |  |  |  | x |
|  |  | 188 | x |  |  |  |  |
|  |  | 198 | x | x |  |  | x |
|  |  | 206 | x | x |  |  |  |
| ENSAPLG00000008173 | TAB2 | 600 | x |  |  |  |  |
|  |  | 605 | x | x |  |  |  |
| ENSAPLG00000008655 | LBP | 72 | x | x |  | x | x |
|  |  | 77 | x | x |  |  |  |
|  |  | 356 | x | x |  |  |  |
| ENSAPLG00000009205 | MAP3K8 | 59 | x |  |  |  |  |
| ENSAPLG00000009391 | CCL24 isoform 2 ^a^ | 13 | x | x | x |  |  |
|  |  | 48 | x | x |  |  |  |
| ENSAPLG00000009391 | CCL24 isoform 1 ^a^ | 47 | *x* | x |  |  |  |
| ENSAPLG00000009929 | TLR15 | 21 |  | x |  |  | x |
| ENSAPLG00000010090 | NLRP12 | 385 | x |  |  |  |  |
| ENSAPLG00000011364 | IFNAR2 | 29 | *x* | x |  |  |  |
|  |  | 33 | x | x |  |  | x |
|  |  | 40 | x | x |  |  | x |
|  |  | 46 | x | x |  |  |  |
|  |  | 51 | x | x |  |  |  |
|  |  | 71 | x | x | x |  | x |
|  |  | 73 | x | x | x |  | x |
|  |  | 76 | x | x | x |  | x |
|  |  | 99 | x | x | x |  | x |
|  |  | 101 | x | x | x |  | x |
|  |  | 102 | x | x | x |  | x |
|  |  | 104 | x | x |  |  | x |
|  |  | 106 | x | x |  |  | x |
|  |  | 108 | x | x | x |  |  |
|  |  | 120 | x | x | x |  | x |
|  |  | 122 | x | x |  |  | x |
|  |  | 126 | x | x | x |  | x |
|  |  | 128 | x | x |  |  |  |
|  |  | 130 | x | x |  |  |  |
|  |  | 149 | x | x |  |  |  |
|  |  | 187 | x | x |  |  |  |
|  |  | 189 | x | x |  |  | x |
|  |  | 225 | x | x | x |  |  |
|  |  | 278 | x | x |  |  |  |
| ENSAPLG00000011397 | TLR2 | 300 | x | x | x |  |  |
|  |  | 306 | x | x | x | x | x |
|  |  | 307 | x | x | x | x | x |
|  |  | 310 | x | x | x |  | x |
|  |  | 452 | x | x |  |  |  |
|  |  | 454 | x |  |  |  | x |
|  |  | 568 | x | x |  |  |  |
|  |  | 585 | x | x |  |  | x |
|  |  | 594 | x | x | x |  | x |
|  |  | 605 | x | x | x |  |  |
|  |  | 633 | x | x | x | x | x |
| ENSAPLG00000011399 | TLR2a | 189 | x |  |  |  |  |
|  |  | 219 | x | x |  |  |  |
|  |  | 363 | x | x |  |  |  |
|  |  | 597 | x | x | x |  | x |
|  |  | 603 | x | x | x | x | x |
| ENSAPLG00000011477 | AKT3 | 14 | x | x |  |  | x |
|  |  | 425 | x |  |  |  |  |
| ENSAPLG00000011675 | PIK3R2 | 24 |  | x |  |  | x |
| ENSAPLG00000011922 | AvBD12 | 60 | x |  |  |  |  |
| ENSAPLG00000012056 | AvBD9 | 29 | x | x | x |  | x |
|  |  | 35 | x | x | x | x | x |
| ENSAPLG00000012239 | AvBD7 | 65 | x |  |  |  |  |
| ENSAPLG00000012287 | AvBD1 | 26 | x | x | x |  |  |
|  |  | 42 | x | x |  |  |  |
|  |  | 48 | x |  |  |  |  |
| ENSAPLG00000012625 | TLR4 | 503 |  | x |  |  | x |
| ENSAPLG00000012692 | IL8-like b | 2 | x | x |  |  |  |
|  |  | 21 | x | x | x |  |  |
| ENSAPLG00000012752 | IRF7 | 68 |  | x | x | x | x |
|  |  | 88 |  | x |  |  | x |
|  |  | 128 |  | x |  |  | x |
|  |  | 242 |  | x | x |  |  |
| ENSAPLG00000012924 | PIK3CA isoform 1 | 77 | x |  |  |  |  |
|  |  | 104 | x | x |  |  |  |
|  |  | 107 | x |  |  |  |  |
| ENSAPLG00000013488 | TRAF6 | 103 |  | x |  |  | x |
|  |  | 137 |  | x |  |  | x |
| ENSAPLG00000013556 | CASP1 | 267 |  | x | x |  | x |
|  |  | 296 |  | x | x |  | x |
| ENSAPLG00000013558 | CD40 | 214 |  | x | x |  | x |
|  |  | 218 |  | x | x |  | x |
| ENSAPLG00000013878 | NFKB1 | 829 | x | x |  |  | x |
| ENSAPLG00000014359 | TICAM1 | 252 | x |  |  |  |  |
|  |  | 277 | x | x | x |  |  |
|  |  | 289 | x | x | x | x | x |
|  |  | 292 | x | x |  |  | x |
|  |  | 367 | x | x | x |  | x |
|  |  | 466 | x | x | x |  |  |
|  |  | 576 | x | x | x | x | x |
|  |  | 649 | x | x |  |  | x |
| ENSAPLG00000014726 | TLR21 | 7 | x | x |  | x |  |
|  |  | 19 | x | x |  |  |  |
|  |  | 62 | x | x |  |  |  |
|  |  | 68 | x | x | x |  | x |
| ENSAPLG00000014996 | IFITM3 | 84 | x | x |  |  |  |
|  |  | 85 | x | x | x | x | x |
| ENSAPLG00000015091 | DDX58 | 421 |  | x | x |  |  |
| ENSAPLG00000015420 | RIPK1 | 405 | x | x | x |  | x |
|  |  | 446 | x | x | x |  | x |
| ENSAPLG00000015976 | Mx | 11 | x | x |  |  |  |
|  |  | 44 | x |  |  |  |  |
|  |  | 63 | x | x | x |  | x |
|  |  | 691 | x | x |  |  |  |
|  |  | 717 | x | x |  |  |  |
| ENSAPLG00000016065 | PIK3R5 | 244 | x |  |  |  |  |
|  |  | 250 | x | x | x |  | x |
|  |  | 569 | x | x |  |  |  |
|  |  | 843 | x | x | x |  | x |

Numbering of nucleotide sites is based on *Anas platyrhynchos*.

^a^ CCL24 = CCL24orCCL4

#### Table S18 Adaptive evolution in the mallards as measured using the MacDonald Kreitman test using Aythya fuligula

|  |  |  |  | Synonymous Substitutions | | Nonsynonymous Substitutions | |  |  |  |  |
| --- | --- | --- | --- | --- | --- | --- | --- | --- | --- | --- | --- |
| Ensembl ID | Gene ID | Mallard (n phased alleles) | Tufted Duck (n phased alleles) | Fixed differences between species | Polymorphic sites | Fixed differences between species | Polymorphic sites | NI | Alfa | *p*-value | adj. *p*-value |
| ENSAPLG00000001279 | TLR5 | 128 | 6 | 12 | 30 | 32 | 77 | 0,963 | 0,038 | 1,000 | 1,000 |
| ENSAPLG00000001297 | AvBD4 | 128 | 6 | 0 | 1 | 5 | 8 | 0,000 | 1,000 | 1,000 | 1,000 |
| ENSAPLG00000001925 | TIRAP | 126 | 6 | 2 | 30 | 3 | 30 | 0,667 | 0,333 | 1,000 | 1,000 |
| ENSAPLG00000002012 | IFNGR1 | 128 | 6 | 8 | 18 | 13 | 23 | 0,786 | 0,214 | 0,788 | 1,000 |
| ENSAPLG00000002049 | TLR1A | 128 | 6 | 15 | 35 | 36 | 59 | 0,702 | 0,298 | 0,367 | 1,000 |
| ENSAPLG00000002168 | MAPK13 | 128 | 6 | 0 | 45 | 1 | 13 | 0,000 | 1,000 | 0,237 | 1,000 |
| ENSAPLG00000002299 | LITAF | 128 | 6 | 5 | 1 | 8 | 0 | 0,000 | 1,000 | 0,429 | 1,000 |
| ENSAPLG00000002603 | MAPK14 | 128 | 6 | 0 | 21 | 0 | 2 | NA | NA | NA | NA |
| ENSAPLG00000002702 | MAP3K7 | 128 | 6 | 1 | 20 | 0 | 1 | NA | NA | 1,000 | 1,000 |
| ENSAPLG00000002771 | TRIM27L isoform 2 ^a^ | 128 | 6 | 0 | 97 | 2 | 53 | 0,000 | 1,000 | 0,129 | 0,809 |
| ENSAPLG00000002771 | TRIM27L isoform 1 ^a^ | 128 | 6 | 0 | 37 | 1 | 41 | 0,000 | 1,000 | 1,000 | 1,000 |
| ENSAPLG00000003004 | SPP1 | 128 | 6 | 4 | 24 | 5 | 20 | 0,667 | 0,333 | 0,719 | 1,000 |
| ENSAPLG00000003430 | IFN | 128 | 6 | 2 | 4 | 6 | 2 | 0,167 | 0,833 | 0,277 | 1,000 |
| ENSAPLG00000003554 | PIK3CB | 128 | 6 | 8 | 42 | 2 | 11 | 1,048 | -0,048 | 1,000 | 1,000 |
| ENSAPLG00000003568 | RAC1 | 128 | 6 | 2 | 6 | 0 | 0 | NA | NA | NA | NA |
| ENSAPLG00000004139 | TLR7 | 128 | 6 | 21 | 50 | 6 | 16 | 1,120 | -0,120 | 1,000 | 1,000 |
| ENSAPLG00000004157 | MAPK12 | 128 | 6 | 6 | 24 | 1 | 3 | 0,750 | 0,250 | 1,000 | 1,000 |
| ENSAPLG00000004376 | IRAK1BP1 | 128 | 6 | 0 | 7 | 2 | 11 | 0,000 | 1,000 | 0,521 | 1,000 |
| ENSAPLG00000004462 | MAPK11 | 128 | 6 | 5 | 26 | 0 | 2 | NA | NA | 1,000 | 1,000 |
| ENSAPLG00000004748 | JAK2 | 128 | 6 | 10 | 12 | 3 | 5 | 1,389 | -0,389 | 1,000 | 1,000 |
| ENSAPLG00000004772 | PIK3R1 | 128 | 6 | 9 | 10 | 4 | 6 | 1,350 | -0,350 | 1,000 | 1,000 |
| ENSAPLG00000004845 | LY96 | 128 | 6 | 2 | 10 | 3 | 5 | 0,333 | 0,667 | 0,347 | 1,000 |
| ENSAPLG00000004947 | TRIM25 | 124 | 6 | 15 | 30 | 15 | 69 | 2,300 | -1,300 | 0,079 | 0,646 |
| ENSAPLG00000005139 | MYD88 b | 128 | 6 | 2 | 31 | 1 | 29 | 1,871 | -0,871 | 1,000 | 1,000 |
| ENSAPLG00000005392 | TRADD | 128 | 6 | 1 | 46 | 0 | 9 | NA | NA | 1,000 | 1,000 |
| ENSAPLG00000005826 | MAP2K6 | 128 | 6 | 2 | 37 | 0 | 7 | NA | NA | 1,000 | 1,000 |
| ENSAPLG00000005856 | MAPK10 | 128 | 6 | 7 | 22 | 0 | 1 | NA | NA | 1,000 | 1,000 |
| ENSAPLG00000005913 | CD86 | 128 | 6 | 4 | 17 | 6 | 9 | 0,353 | 0,647 | 0,260 | 1,000 |
| ENSAPLG00000005942 | IL18 | 128 | 6 | 5 | 2 | 2 | 2 | 2,500 | -1,500 | 0,576 | 1,000 |
| ENSAPLG00000006016 | RSAD2 | 128 | 6 | 5 | 21 | 1 | 2 | 0,476 | 0,524 | 1,000 | 1,000 |
| ENSAPLG00000006116 | MAPK1 | 128 | 6 | 2 | 4 | 0 | 0 | NA | NA | NA | NA |
| ENSAPLG00000006147 | IL12A | 128 | 6 | 1 | 22 | 3 | 16 | 0,242 | 0,758 | 0,313 | 1,000 |
| ENSAPLG00000006379 | IKBKB | 128 | 6 | 9 | 4 | 0 | 2 | NA | NA | 0,143 | 0,840 |
| ENSAPLG00000006669 | PIK3CG | 128 | 6 | 13 | 95 | 1 | 8 | 1,095 | -0,095 | 1,000 | 1,000 |
| ENSAPLG00000006823 | PIK3R3 | 104 | 6 | 6 | 70 | 2 | 48 | 2,057 | -1,057 | 0,476 | 1,000 |
| ENSAPLG00000007148 | MAP2K3 | 128 | 6 | 0 | 53 | 0 | 4 | NA | NA | NA | NA |
| ENSAPLG00000007417 | CD80 | 128 | 6 | 5 | 28 | 9 | 13 | 0,258 | 0,742 | 0,056 | 0,646 |
| ENSAPLG00000007876 | TANK | 128 | 6 | 0 | 5 | 2 | 4 | 0,000 | 1,000 | 0,455 | 1,000 |
| ENSAPLG00000008173 | TAB2 | 128 | 6 | 3 | 20 | 2 | 9 | 0,675 | 0,325 | 1,000 | 1,000 |
| ENSAPLG00000008183 | PIK3CD | 128 | 6 | 7 | 100 | 0 | 13 | NA | NA | 0,605 | 1,000 |
| ENSAPLG00000008445 | TBK1 | 128 | 6 | 10 | 53 | 3 | 7 | 0,440 | 0,560 | 0,369 | 1,000 |
| ENSAPLG00000008655 | LBP | 128 | 6 | 0 | 67 | 3 | 73 | 0,000 | 1,000 | 0,248 | 1,000 |
| ENSAPLG00000008976 | TLR3 | 128 | 6 | 15 | 38 | 13 | 35 | 1,063 | -0,063 | 1,000 | 1,000 |
| ENSAPLG00000009092 | IL12B | 128 | 6 | 4 | 5 | 3 | 7 | 1,867 | -0,867 | 0,650 | 1,000 |
| ENSAPLG00000009156 | TOLLIP | 128 | 6 | 2 | 7 | 0 | 4 | NA | NA | 0,538 | 1,000 |
| ENSAPLG00000009205 | MAP3K8 | 128 | 6 | 6 | 36 | 3 | 3 | 0,167 | 0,833 | 0,071 | 0,646 |
| ENSAPLG00000009391 | CCL24 isoform 1 ^b^ | 128 | 6 | 0 | 2 | 1 | 3 | 0,000 | 1,000 | 1,000 | 1,000 |
| ENSAPLG00000009391 | CCL24 isoform 2 ^b^ | 128 | 6 | 0 | 2 | 2 | 4 | 0,000 | 1,000 | 1,000 | 1,000 |
| ENSAPLG00000009838 | CCL5 b | 128 | 6 | 1 | 2 | 2 | 4 | 1,000 | 0,000 | 1,000 | 1,000 |
| ENSAPLG00000009929 | TLR15 | 128 | 6 | 12 | 67 | 24 | 33 | 0,246 | 0,754 | 0,001 | 0,023 |
| ENSAPLG00000010090 | NLRP12 | 128 | 6 | 16 | 63 | 21 | 113 | 1,367 | -0,367 | 0,455 | 1,000 |
| ENSAPLG00000010194 | MYD88 a | 126 | 6 | 0 | 9 | 0 | 0 | NA | NA | NA | NA |
| ENSAPLG00000010913 | IFIH1 | 128 | 6 | 23 | 22 | 20 | 30 | 1,568 | -0,568 | 0,308 | 1,000 |
| ENSAPLG00000011028 | NFKBIA | 128 | 6 | 9 | 24 | 0 | 5 | NA | NA | 0,312 | 1,000 |
| ENSAPLG00000011045 | MAP2K2 | 128 | 6 | 2 | 28 | 0 | 8 | NA | NA | 1,000 | 1,000 |
| ENSAPLG00000011237 | IFNGR2 | 128 | 6 | 8 | 27 | 13 | 35 | 0,798 | 0,202 | 0,800 | 1,000 |
| ENSAPLG00000011322 | IFNAR1 | 128 | 6 | 10 | 16 | 46 | 28 | 0,380 | 0,620 | 0,042 | 0,597 |
| ENSAPLG00000011364 | IFNAR2 | 128 | 6 | 10 | 16 | 31 | 70 | 1,411 | -0,411 | 0,485 | 1,000 |
| ENSAPLG00000011397 | TLR2 | 128 | 6 | 7 | 155 | 17 | 286 | 0,760 | 0,240 | 0,663 | 1,000 |
| ENSAPLG00000011399 | TLR2a | 128 | 6 | 11 | 52 | 11 | 58 | 1,115 | -0,115 | 0,820 | 1,000 |
| ENSAPLG00000011477 | AKT3 | 128 | 6 | 4 | 13 | 0 | 2 | NA | NA | 1,000 | 1,000 |
| ENSAPLG00000011503 | CHUK | 128 | 6 | 17 | 33 | 5 | 11 | 1,133 | -0,133 | 1,000 | 1,000 |
| ENSAPLG00000011572 | AvBD8 | 128 | 6 | 0 | 5 | 0 | 4 | NA | NA | NA | NA |
| ENSAPLG00000011754 | IL6 | 128 | 6 | 2 | 12 | 0 | 2 | NA | NA | 1,000 | 1,000 |
| ENSAPLG00000011919 | AvBD13 | 128 | 6 | 0 | 10 | 0 | 7 | NA | NA | NA | NA |
| ENSAPLG00000011922 | AvBD12 | 128 | 6 | 1 | 16 | 1 | 5 | 0,313 | 0,688 | 0,462 | 1,000 |
| ENSAPLG00000011923 | AvBD11 | 128 | 6 | 0 | 17 | 0 | 17 | NA | NA | NA | NA |
| ENSAPLG00000011932 | AvBD10 | 128 | 6 | 0 | 15 | 1 | 2 | 0,000 | 1,000 | 0,167 | 0,926 |
| ENSAPLG00000011933 | TRAF3 | 128 | 6 | 4 | 26 | 0 | 2 | NA | NA | 1,000 | 1,000 |
| ENSAPLG00000012047 | JAK1 | 128 | 6 | 6 | 119 | 6 | 6 | 0,050 | 0,950 | 0,000 | 0,006 |
| ENSAPLG00000012056 | AvBD9 | 128 | 6 | 3 | 3 | 2 | 6 | 3,000 | -2,000 | 0,580 | 1,000 |
| ENSAPLG00000012251 | AvBD2 | 128 | 6 | 1 | 6 | 3 | 2 | 0,111 | 0,889 | 0,222 | 1,000 |
| ENSAPLG00000012255 | IRAK4 | 110 | 6 | 10 | 42 | 6 | 36 | 1,429 | -0,429 | 0,590 | 1,000 |
| ENSAPLG00000012287 | AvBD1 | 128 | 6 | 0 | 2 | 0 | 5 | NA | NA | NA | NA |
| ENSAPLG00000012288 | AvBD16_or_AvBD3a | 128 | 6 | 1 | 7 | 1 | 19 | 2,714 | -1,714 | 0,497 | 1,000 |
| ENSAPLG00000012380 | AZI2 | 128 | 6 | 2 | 32 | 2 | 27 | 0,844 | 0,156 | 1,000 | 1,000 |
| ENSAPLG00000012383 | CCL4 b | 128 | 6 | 0 | 1 | 0 | 1 | NA | NA | NA | NA |
| ENSAPLG00000012465 | AvBD5 | 126 | 6 | 1 | 9 | 0 | 5 | NA | NA | 1,000 | 1,000 |
| ENSAPLG00000012580 | AvBD14 | 128 | 6 | 0 | 7 | 0 | 4 | NA | NA | NA | NA |
| ENSAPLG00000012625 | TLR4 | 128 | 6 | 13 | 56 | 9 | 37 | 0,954 | 0,046 | 1,000 | 1,000 |
| ENSAPLG00000012692 | IL8-like b | 128 | 6 | 0 | 5 | 2 | 1 | 0,000 | 1,000 | 0,107 | 0,714 |
| ENSAPLG00000012697 | IL8-like a | 128 | 6 | 3 | 8 | 3 | 2 | 0,250 | 0,750 | 0,299 | 1,000 |
| ENSAPLG00000012768 | CTSK | 128 | 6 | 11 | 18 | 0 | 13 | NA | NA | 0,009 | 0,231 |
| ENSAPLG00000012773 | CCL4 a | 128 | 6 | 1 | 8 | 0 | 15 | NA | NA | 0,375 | 1,000 |
| ENSAPLG00000012876 | IFNG | 128 | 6 | 2 | 9 | 1 | 2 | 0,444 | 0,556 | 1,000 | 1,000 |
| ENSAPLG00000012924 | PIK3CA isoform 1 | 128 | 6 | 6 | 29 | 3 | 5 | 0,345 | 0,655 | 0,332 | 1,000 |
| ENSAPLG00000012924 | PIK3CA isoform 2 | 128 | 6 | 11 | 54 | 0 | 0 | NA | NA | NA | NA |
| ENSAPLG00000013074 | MAP2K4 | 128 | 6 | 4 | 6 | 1 | 2 | 1,333 | -0,333 | 1,000 | 1,000 |
| ENSAPLG00000013226 | STAT1 b | 128 | 6 | 4 | 59 | 2 | 2 | 0,068 | 0,932 | 0,037 | 0,597 |
| ENSAPLG00000013262 | STAT1 a | 128 | 6 | 1 | 35 | 0 | 3 | NA | NA | 1,000 | 1,000 |
| ENSAPLG00000013460 | MAPK9 | 128 | 6 | 1 | 11 | 0 | 2 | NA | NA | 1,000 | 1,000 |
| ENSAPLG00000013488 | TRAF6 | 128 | 6 | 4 | 36 | 0 | 5 | NA | NA | 1,000 | 1,000 |
| ENSAPLG00000013556 | CASP1 | 128 | 6 | 7 | 36 | 13 | 45 | 0,673 | 0,327 | 0,465 | 1,000 |
| ENSAPLG00000013558 | CD40 | 116 | 6 | 2 | 19 | 7 | 7 | 0,105 | 0,895 | 0,015 | 0,306 |
| ENSAPLG00000013878 | NFKB1 | 128 | 6 | 12 | 79 | 6 | 26 | 0,658 | 0,342 | 0,561 | 1,000 |
| ENSAPLG00000014351 | IKBKE | 128 | 6 | 2 | 131 | 4 | 58 | 0,221 | 0,779 | 0,082 | 0,646 |
| ENSAPLG00000014359 | TICAM1 | 128 | 6 | 6 | 84 | 13 | 109 | 0,599 | 0,401 | 0,344 | 1,000 |
| ENSAPLG00000014377 | CD14 isoform 1 | 126 | 6 | 1 | 62 | 3 | 63 | 0,339 | 0,661 | 0,619 | 1,000 |
| ENSAPLG00000014377 | CD14 isoform 2 | 128 | 6 | 1 | 36 | 2 | 41 | 0,569 | 0,431 | 1,000 | 1,000 |
| ENSAPLG00000014394 | TNFRSF6B | 116 | 6 | 5 | 37 | 2 | 34 | 2,297 | -1,297 | 0,442 | 1,000 |
| ENSAPLG00000014631 | TMEM173 | 118 | 6 | 1 | 78 | 1 | 72 | 0,923 | 0,077 | 1,000 | 1,000 |
| ENSAPLG00000014872 | CASP8 | 128 | 6 | 11 | 11 | 3 | 12 | 4,000 | -3,000 | 0,090 | 0,646 |
| ENSAPLG00000015091 | DDX58 | 128 | 6 | 24 | 20 | 14 | 8 | 0,686 | 0,314 | 0,600 | 1,000 |
| ENSAPLG00000015131 | TAB1 | 128 | 6 | 3 | 75 | 1 | 4 | 0,160 | 0,840 | 0,224 | 1,000 |
| ENSAPLG00000015244 | CASP10 | 128 | 6 | 5 | 16 | 9 | 20 | 0,694 | 0,306 | 0,752 | 1,000 |
| ENSAPLG00000015420 | RIPK1 | 128 | 6 | 5 | 65 | 9 | 39 | 0,333 | 0,667 | 0,081 | 0,646 |
| ENSAPLG00000015480 | MAP2K1 | 128 | 6 | 5 | 24 | 0 | 2 | NA | NA | 1,000 | 1,000 |
| ENSAPLG00000015976 | Mx | 128 | 6 | 6 | 28 | 39 | 28 | 0,154 | 0,846 | 0,000 | 0,006 |
| ENSAPLG00000016065 | PIK3R5 | 128 | 6 | 10 | 134 | 2 | 50 | 1,866 | -0,866 | 0,522 | 1,000 |
| ENSAPLG00000016230 | TRAF2 | 128 | 6 | 6 | 22 | 0 | 2 | NA | NA | 1,000 | 1,000 |
| ENSAPLG00000016314 | AKT1 | 128 | 6 | 3 | 24 | 3 | 4 | 0,167 | 0,833 | 0,086 | 0,646 |
| ENSAPLG00000016448 | MAPK8 | 128 | 6 | 6 | 5 | 0 | 1 | NA | NA | 1,000 | 1,000 |

^a^ TRIM27L = TRIM27-like

^b^ CCL24 = CCL24orCCL4

#### Table S19 Adaptive evolution in each mallard population as measured using the MacDonald Kreitman test using Aythya fuligula

|  |  |  |  |  | Synonymous Substitutions | | Nonsynonymous Substitutions | |  |  |  |  |
| --- | --- | --- | --- | --- | --- | --- | --- | --- | --- | --- | --- | --- |
| Ensembl ID | Gene ID | Population | Mallard (n phased alleles) | Tufted duck (n phased alleles) | Fixed differences between species | Polymorphic sites | Fixed differences between species | Polymorphic sites | NI | Alfa | *p*-value | adj. *p*-value |
| ENSAPLG00000012047 | JAK1 | Canada | 32 | 6 | 6 | 94 | 6 | 3 | 0,03 | 0,97 | 3,30E-05 | 0,000 |
|  |  | Spain | 32 | 6 | 7 | 82 | 7 | 3 | 0,04 | 0,96 | 2,20E-05 | 0,000 |
|  |  | Greenland | 30 | 6 | 11 | 51 | 7 | 2 | 0,06 | 0,94 | 6,21E-04 | 0,001 |
|  |  | Sweden | 32 | 6 | 9 | 74 | 7 | 1 | 0,02 | 0,98 | 1,00E-05 | 0,000 |
| ENSAPLG00000015976 | MX | Canada | 32 | 6 | 6 | 20 | 40 | 15 | 0,11 | 0,89 | 3,40E-05 | 0,000 |
|  |  | Spain | 32 | 6 | 6 | 10 | 42 | 10 | 0,14 | 0,86 | 3,29E-03 | 0,004 |
|  |  | Greenland | 30 | 6 | 6 | 9 | 42 | 8 | 0,13 | 0,87 | 1,61E-03 | 0,003 |
|  |  | Sweden | 32 | 6 | 6 | 14 | 39 | 19 | 0,21 | 0,79 | 7,64E-03 | 0,008 |
| ENSAPLG00000009929 | TLR15 | Canada | 32 | 6 | 12 | 45 | 24 | 26 | 0,29 | 0,71 | 4,14E-03 | 0,005 |
|  |  | Spain | 32 | 6 | 13 | 38 | 24 | 18 | 0,26 | 0,74 | 2,77E-03 | 0,004 |
|  |  | Greenland | 30 | 6 | 13 | 30 | 25 | 13 | 0,23 | 0,78 | 1,82E-03 | 0,003 |
|  |  | Sweden | 32 | 6 | 13 | 42 | 24 | 18 | 0,23 | 0,77 | 1,39E-03 | 0,003 |

#### Table S20 Domain prediction using Interpro

| Gene | Predicted Domain | Predicted location | | Fixed differences between the Mallard and the Tufted duck | |
| --- | --- | --- | --- | --- | --- |
|  |  | Amino acid position | Nucleotide position | Synonymous changes (nucl. position) | Nonsynonymous changes (nucl. position) |
| Mx | Dynamin, GTPase domain | 107-401 | 319-1203 | 621, 709, 1035, 1086 | 530, 553, 806, 1012, 1072, 1106 |
|  | Dynamin central domain | 322-606 | 964-1818 | 1035, 1086, 1260, 1618 | 1012, 1072, 1106, 1214, 1282, 1288, 1354, 1438, 1451, 1669, 1693, 1697, 1810, 1811, 1816 |
|  | GTPase effector domain | 627-721 | 1879-2163 |  | 1934, 1940, 2014, 2071, 2091, 2111 |
|  | *Fixed codons outside predicted domains* |  |  |  | 3, 65, 85, 89, 100, 133, 140, 155, 188, 190, 202, 230, 264, 1834, 1865 |
| TLR15 | Leucine-rich repeat domains | 25-121, 156-628 | 73-363, 466-1884 | 117, 537, 588, 774, 783, 823, 1033, 1035, 1464, 1572, 1713 | 266, 313, 316, 341, 502, 739, 767, 847, 938, 1181, 1276, 1277, 1382, 1465, 1661, 1741, 1847 |
|  | Toll/interleukin-1 receptor homology (TIR) domain | 670-824 | 2008-2472 | 2316 | 2081 |
|  | *Fixed codons outside predicted domains* |  |  |  | 31, 400, 413, 1925, 1969, 1983 |
| JAK1 | FERM domain | 1-259 | 1-777 | 27, 696 | 367, 697 |
|  | SH2 domain | 276-370 | 826-1110 |  |  |
|  | Protein kinase domain | 421-686, 714-962 | 1261-2058, 2140-2886 | 1374, 2007, 2502 | 2444, 2506, 2511 |
|  | *Fixed codons outside predicted domains* |  |  | 1227 | 1216 |

#### Table S21 SNPs under natural selection in wild mallards identified using Bayescan

| Gene | Gene name | Scaffold name | Location | Exon/Intron | Locus ID (SNP) | prob | log10(PO) | qval | alpha | *F*_ST_ | Protein alternation |
| --- | --- | --- | --- | --- | --- | --- | --- | --- | --- | --- | --- |
| ENSAPLG00000002299 | LITAF | KB744198.1 | 64858 | intron | 603 | 0.96759 | 1.4751 | 0.0048310 | 1.9077 | 0.19876 |  |
| ENSAPLG00000002771 | TRIM27L^a^ | KB742989.1 | 101314 | intron | 1145 | 0.87558 | 0.84739 | 0.024181 | 1.3304 | 0.15065 |  |
| ENSAPLG00000005856 | MAPK10 | KB742619.1 | 4006345 | intron | 1994 | 0.86717 | 0.81482 | 0.028360 | 1.3259 | 0.15042 |  |
| ENSAPLG00000004376 | IRAK1BP1 | KB742953.1 | 397168 | intron | 4582 | 0.73315 | 0.43892 | 0.095545 | 1.0785 | 0.13295 |  |
| ENSAPLG00000004772 | PIK3R1 | KB742448.1 | 1057150 | intron | 5174 | 0.99240 | 2.1158 | 0.0014356 | 1.6512 | 0.17519 |  |
| ENSAPLG00000005826 | MAP2K6 | KB743912.1 | 247390 | intron | 5855 | 0.73015 | 0.43228 | 0.099506 | 1.1604 | 0.13989 |  |
| ENSAPLG00000005942 | IL18 | KB746795.1 | 113472 | intron | 6006 | 0.96799 | 1.4806 | 0.0033796 | 1.8875 | 0.19708 |  |
| ENSAPLG00000009156 | TOLLIP | KB743110.1 | 86366 | intron | 8603 | 1.0000 | 1000.0 | 0.0000 | 2.5333 | 0.25567 |  |
| ENSAPLG00000009156 | TOLLIP | KB743110.1 | 86401 | intron | 8604 | 1.0000 | 1000.0 | 0.0000 | 2.5349 | 0.25593 |  |
| ENSAPLG00000009156 | TOLLIP | KB743110.1 | 86594 | intron | 8605 | 1.0000 | 1000.0 | 0.0000 | 2.5445 | 0.25672 |  |
| ENSAPLG00000009156 | TOLLIP | KB743110.1 | 88403 | intron | 8611 | 1.0000 | 1000.0 | 0.0000 | 2.5155 | 0.25404 |  |
| ENSAPLG00000009156 | TOLLIP | KB743110.1 | 88641 | intron | 8613 | 1.0000 | 1000.0 | 0.0000 | 2.5176 | 0.25409 |  |
| ENSAPLG00000009156 | TOLLIP | KB743110.1 | 88854 | intron | 8616 | 1.0000 | 1000.0 | 0.0000 | 2.5439 | 0.25689 |  |
| ENSAPLG00000009156 | TOLLIP | KB743110.1 | 90142 | intron | 8622 | 1.0000 | 1000.0 | 0.0000 | 2.5321 | 0.25581 |  |
| ENSAPLG00000009156 | TOLLIP | KB743110.1 | 90147 | intron | 8623 | 1.0000 | 1000.0 | 0.0000 | 2.5197 | 0.25424 |  |
| ENSAPLG00000009156 | TOLLIP | KB743110.1 | 91157 | intron | 8629 | 1.0000 | 1000.0 | 0.0000 | 2.3039 | 0.23371 |  |
| ENSAPLG00000009156 | TOLLIP | KB743110.1 | 91368 | intron | 8632 | 0.99540 | 2.3352 | 0.00080016 | 2.0477 | 0.21076 |  |
| ENSAPLG00000009156 | TOLLIP | KB743110.1 | 92313 | intron | 8639 | 0.99940 | 3.2215 | 0.00010911 | 2.1862 | 0.22314 |  |
| ENSAPLG00000009156 | TOLLIP | KB743110.1 | 92801 | intron | 8642 | 0.99940 | 3.2215 | 0.00010911 | 2.1776 | 0.22247 |  |
| ENSAPLG00000009156 | TOLLIP | KB743110.1 | 92964 | intron | 8644 | 0.99780 | 2.6565 | 0.00052868 | 2.1944 | 0.22390 |  |
| ENSAPLG00000009156 | TOLLIP | KB743110.1 | 94373 | intron | 8650 | 0.99800 | 2.6980 | 0.00040008 | 2.1709 | 0.22180 |  |
| ENSAPLG00000009156 | TOLLIP | KB743110.1 | 94437 | intron | 8652 | 0.99220 | 2.1044 | 0.0017892 | 2.0340 | 0.20958 |  |
| ENSAPLG00000009156 | TOLLIP | KB743110.1 | 94813 | intron | 8654 | 0.99800 | 2.6980 | 0.00040008 | 2.1770 | 0.22242 |  |
| ENSAPLG00000009929 | TLR-15 | KB743040.1 | 2572409 | exon | 9163 | 0.99520 | 2.3166 | 0.0010502 | 2.1461 | 0.21951 | synonymous |
| ENSAPLG00000009929 | TLR-15 | KB743040.1 | 2574030 | exon | 9182 | 0.89458 | 0.92869 | 0.015812 | 1.4923 | 0.16399 | non-synonymous, A-V |
| ENSAPLG00000009929 | TLR-15 | KB743040.1 | 2574035 | exon | 9183 | 0.88358 | 0.88020 | 0.020004 | 1.6121 | 0.17489 | synonymous |
| ENSAPLG00000009929 | TLR-15 | KB743040.1 | 2575048 | intron | 9203 | 0.82356 | 0.66911 | 0.054441 | 1.4485 | 0.16216 |  |
| ENSAPLG00000009929 | TLR-15 | KB743040.1 | 2575778 | intron | 9212 | 0.91218 | 1.0165 | 0.011739 | 1.5975 | 0.17262 |  |
| ENSAPLG00000009929 | TLR-15 | KB743040.1 | 2581687 | intron | 9333 | 0.75235 | 0.48258 | 0.087364 | 1.3731 | 0.15793 |  |
| ENSAPLG00000009929 | TLR-15 | KB743040.1 | 2583536 | intron | 9356 | 0.77956 | 0.54855 | 0.070728 | 1.3346 | 0.15353 |  |
| ENSAPLG00000009929 | TLR-15 | KB743040.1 | 2585694 | intron | 9398 | 0.74035 | 0.45504 | 0.091466 | 1.3391 | 0.15505 |  |
| ENSAPLG00000009929 | TLR-15 | KB743040.1 | 2586358 | intron | 9402 | 0.76275 | 0.50718 | 0.075110 | 1.3672 | 0.15699 |  |
| ENSAPLG00000009929 | TLR-15 | KB743040.1 | 2586635 | intron | 9410 | 0.78196 | 0.55464 | 0.066569 | 1.3856 | 0.15792 |  |
| ENSAPLG00000009929 | TLR-15 | KB743040.1 | 2586797 | intron | 9412 | 0.84117 | 0.72395 | 0.046835 | 1.5519 | 0.17078 |  |
| ENSAPLG00000009929 | TLR-15 | KB743040.1 | 2586862 | intron | 9414 | 0.75875 | 0.49764 | 0.083357 | 1.3724 | 0.15736 |  |
| ENSAPLG00000009929 | TLR-15 | KB743040.1 | 2587065 | intron | 9418 | 0.85197 | 0.76008 | 0.043102 | 1.5818 | 0.17297 |  |
| ENSAPLG00000009929 | TLR-15 | KB743040.1 | 2587107 | intron | 9419 | 0.86537 | 0.80807 | 0.032295 | 1.5947 | 0.17379 |  |
| ENSAPLG00000009929 | TLR-15 | KB743040.1 | 2587288 | intron | 9422 | 0.86497 | 0.80658 | 0.035964 | 1.5991 | 0.17414 |  |
| ENSAPLG00000009929 | TLR-15 | KB743040.1 | 2589835 | intron | 9460 | 0.81296 | 0.63814 | 0.058341 | 1.4481 | 0.16235 |  |
| ENSAPLG00000011364 | IFNAR2 | KB742682.1 | 797722 | intron | 9756 | 0.86197 | 0.79553 | 0.039484 | 1.6227 | 0.17665 |  |
| ENSAPLG00000013878 | NFKB1 | KB742932.1 | 478239 | inton | 13673 | 0.83177 | 0.69409 | 0.050629 | 1.2800 | 0.14750 |  |
| ENSAPLG00000014351 | IKBKE | KB742563.1 | 644481 | intron | 13849 | 0.80516 | 0.61621 | 0.062241 | 1.2545 | 0.14583 |  |
| ENSAPLG00000015420 | RIPK1 | KB743404.1 | 1381140 | intron | 14970 | 0.92619 | 1.0986 | 0.0081159 | 1.4655 | 0.16093 |  |
| ENSAPLG00000015976 | Mx | KB743889.1 | 35670 | intron | 15527 | 0.76115 | 0.50335 | 0.079308 | 1.1244 | 0.13601 |  |

^a^ TRIM27L = TRIM27-like

#### Table S22 SNPs under natural selection in all mallards (wild mallards, farm mallards and Pekin ducks) combined identified using Bayescan

| Gene | Gene name | Scaffold name | Location | Exon/Intron | Locus ID (SNP) | prob | log10(PO) | qval | alpha | Fst | Protein alternation |
| --- | --- | --- | --- | --- | --- | --- | --- | --- | --- | --- | --- |
| ENSAPLG00000002299 | LITAF | KB744198.1 | 64858 | intron | 603 | 1,00 | 2,35 | 0,001 | 1,93 | 0,34 |  |
| ENSAPLG00000002771 | TRIM27L^a^ | KB742989.1 | 106271 | intron | 1260 | 0,73 | 0,43 | 0,083 | -1,16 | 0,06 |  |
| ENSAPLG00000005856 | MAPK10 | KB742619.1 | 3984008 | intron | 1609 | 0,90 | 0,96 | 0,026 | 1,27 | 0,26 |  |
| ENSAPLG00000005856 | MAPK10 | KB742619.1 | 3989127 | intron | 1686 | 0,95 | 1,30 | 0,012 | 1,36 | 0,27 |  |
| ENSAPLG00000005856 | MAPK10 | KB742619.1 | 4006345 | intron | 1994 | 0,85 | 0,74 | 0,044 | 1,17 | 0,25 |  |
| ENSAPLG00000004462 | MAPK11 | KB743204.1 | 2159532 | intron | 3393 | 0,88 | 0,88 | 0,029 | 1,20 | 0,25 |  |
| ENSAPLG00000004376 | IRAK1BP1 | KB742953.1 | 387343 | intron | 4479 | 0,93 | 1,10 | 0,019 | 1,23 | 0,26 |  |
| ENSAPLG00000004772 | PIK3R1 | KB742448.1 | 1047332 | intron | 5111 | 0,88 | 0,86 | 0,030 | 1,28 | 0,26 |  |
| ENSAPLG00000004772 | PIK3R1 | KB742448.1 | 1047442 | intron | 5112 | 0,86 | 0,80 | 0,035 | 1,28 | 0,26 |  |
| ENSAPLG00000004772 | PIK3R1 | KB742448.1 | 1047573 | intron | 5114 | 0,72 | 0,42 | 0,084 | 0,94 | 0,22 |  |
| ENSAPLG00000004772 | PIK3R1 | KB742448.1 | 1048739 | intron | 5120 | 0,96 | 1,44 | 0,008 | 1,40 | 0,28 |  |
| ENSAPLG00000004772 | PIK3R1 | KB742448.1 | 1049361 | intron | 5122 | 0,97 | 1,53 | 0,007 | 1,45 | 0,28 |  |
| ENSAPLG00000004772 | PIK3R1 | KB742448.1 | 1049514 | intron | 5124 | 0,99 | 2,18 | 0,002 | 1,51 | 0,29 |  |
| ENSAPLG00000004772 | PIK3R1 | KB742448.1 | 1052036 | intron | 5133 | 0,91 | 1,02 | 0,022 | 1,44 | 0,28 |  |
| ENSAPLG00000004772 | PIK3R1 | KB742448.1 | 1053833 | intron | 5147 | 0,93 | 1,15 | 0,017 | 1,28 | 0,26 |  |
| ENSAPLG00000004772 | PIK3R1 | KB742448.1 | 1057150 | intron | 5174 | 0,99 | 2,04 | 0,002 | 1,48 | 0,29 |  |
| ENSAPLG00000004772 | PIK3R1 | KB742448.1 | 1057304 | intron | 5175 | 0,90 | 0,94 | 0,027 | 1,23 | 0,26 |  |
| ENSAPLG00000004772 | PIK3R1 | KB742448.1 | 1057316 | intron | 5176 | 0,86 | 0,78 | 0,041 | 1,15 | 0,25 |  |
| ENSAPLG00000004772 | PIK3R1 | KB742448.1 | 1057389 | intron | 5177 | 0,93 | 1,15 | 0,017 | 1,35 | 0,27 |  |
| ENSAPLG00000004772 | PIK3R1 | KB742448.1 | 1058224 | intron | 5182 | 0,84 | 0,72 | 0,047 | 1,30 | 0,27 |  |
| ENSAPLG00000004772 | PIK3R1 | KB742448.1 | 1077978 | intron | 5274 | 0,76 | 0,51 | 0,072 | 0,93 | 0,22 |  |
| ENSAPLG00000004772 | PIK3R1 | KB742448.1 | 1089692 | intron | 5326 | 0,93 | 1,16 | 0,016 | 1,55 | 0,29 |  |
| ENSAPLG00000005942 | IL18 | KB746795.1 | 113472 | intron | 6006 | 1,00 | 2,44 | 0,001 | 1,92 | 0,34 |  |
| ENSAPLG00000006147 | IL12A | KB744208.1 | 567939 | intron | 6174 | 0,95 | 1,30 | 0,013 | 1,23 | 0,25 |  |
| ENSAPLG00000006669 | PIK3CG | KB742471.1 | 569698 | intron | 6297 | 0,85 | 0,74 | 0,045 | 1,13 | 0,24 |  |
| ENSAPLG00000006669 | PIK3CG | KB742471.1 | 571455 | intron | 6313 | 0,92 | 1,04 | 0,020 | 1,23 | 0,26 |  |
| ENSAPLG00000007417 | CD80 | KB742931.1 | 1566776 | intron | 7217 | 0,77 | 0,53 | 0,064 | 0,93 | 0,22 |  |
| ENSAPLG00000010913 | IFIH1 | KB744072.1 | 1090355 | intron | 7464 | 0,78 | 0,54 | 0,063 | 1,16 | 0,25 |  |
| ENSAPLG00000008445 | TBK1 | KB744799.1 | 52648 | intron | 8142 | 0,71 | 0,40 | 0,087 | 0,87 | 0,22 |  |
| ENSAPLG00000008445 | TBK1 | KB744799.1 | 52671 | intron | 8143 | 0,72 | 0,41 | 0,086 | 0,87 | 0,22 |  |
| ENSAPLG00000009156 | TOLLIP | KB743110.1 | 86366 | intron | 8603 | 1,00 | 1000,00 | 0,000 | 2,45 | 0,41 |  |
| ENSAPLG00000009156 | TOLLIP | KB743110.1 | 86401 | intron | 8604 | 1,00 | 1000,00 | 0,000 | 2,45 | 0,41 |  |
| ENSAPLG00000009156 | TOLLIP | KB743110.1 | 86594 | intron | 8605 | 1,00 | 1000,00 | 0,000 | 2,47 | 0,41 |  |
| ENSAPLG00000009156 | TOLLIP | KB743110.1 | 88403 | intron | 8611 | 1,00 | 1000,00 | 0,000 | 2,46 | 0,41 |  |
| ENSAPLG00000009156 | TOLLIP | KB743110.1 | 88641 | intron | 8613 | 1,00 | 1000,00 | 0,000 | 2,46 | 0,41 |  |
| ENSAPLG00000009156 | TOLLIP | KB743110.1 | 88854 | intron | 8616 | 1,00 | 1000,00 | 0,000 | 2,46 | 0,41 |  |
| ENSAPLG00000009156 | TOLLIP | KB743110.1 | 90142 | intron | 8622 | 1,00 | 1000,00 | 0,000 | 2,46 | 0,41 |  |
| ENSAPLG00000009156 | TOLLIP | KB743110.1 | 90147 | intron | 8623 | 1,00 | 1000,00 | 0,000 | 2,46 | 0,41 |  |
| ENSAPLG00000009156 | TOLLIP | KB743110.1 | 91157 | intron | 8629 | 1,00 | 1000,00 | 0,000 | 2,26 | 0,38 |  |
| ENSAPLG00000009156 | TOLLIP | KB743110.1 | 91368 | intron | 8632 | 1,00 | 2,85 | 0,000 | 2,04 | 0,35 |  |
| ENSAPLG00000009156 | TOLLIP | KB743110.1 | 92313 | intron | 8639 | 1,00 | 3,70 | 0,000 | 2,17 | 0,37 |  |
| ENSAPLG00000009156 | TOLLIP | KB743110.1 | 92801 | intron | 8642 | 1,00 | 3,70 | 0,000 | 2,14 | 0,37 |  |
| ENSAPLG00000009156 | TOLLIP | KB743110.1 | 92964 | intron | 8644 | 1,00 | 3,70 | 0,000 | 2,17 | 0,37 |  |
| ENSAPLG00000009156 | TOLLIP | KB743110.1 | 94373 | intron | 8650 | 1,00 | 3,70 | 0,000 | 2,15 | 0,37 |  |
| ENSAPLG00000009156 | TOLLIP | KB743110.1 | 94437 | intron | 8652 | 1,00 | 2,85 | 0,000 | 2,04 | 0,35 |  |
| ENSAPLG00000009156 | TOLLIP | KB743110.1 | 94813 | intron | 8654 | 1,00 | 1000,00 | 0,000 | 2,15 | 0,37 |  |
| ENSAPLG00000009205 | MAP3K8 | KB742630.1 | 58826 | intron | 8700 | 0,96 | 1,44 | 0,008 | 1,34 | 0,27 |  |
| ENSAPLG00000009205 | MAP3K8 | KB742630.1 | 59049 | intron | 8706 | 0,98 | 1,73 | 0,004 | 1,40 | 0,27 |  |
| ENSAPLG00000009205 | MAP3K8 | KB742630.1 | 59114 | intron | 8709 | 0,98 | 1,69 | 0,006 | 1,40 | 0,28 |  |
| ENSAPLG00000009205 | MAP3K8 | KB742630.1 | 59833 | intron | 8721 | 0,96 | 1,43 | 0,009 | 1,34 | 0,27 |  |
| ENSAPLG00000009205 | MAP3K8 | KB742630.1 | 60591 | intron | 8735 | 1,00 | 2,66 | 0,001 | 1,57 | 0,30 |  |
| ENSAPLG00000009205 | MAP3K8 | KB742630.1 | 60681 | intron | 8736 | 0,71 | 0,38 | 0,089 | 0,86 | 0,21 |  |
| ENSAPLG00000009205 | MAP3K8 | KB742630.1 | 61834 | intron | 8754 | 0,98 | 1,75 | 0,004 | 1,41 | 0,28 |  |
| ENSAPLG00000009205 | MAP3K8 | KB742630.1 | 64103 | intron | 8793 | 0,99 | 1,86 | 0,003 | 1,43 | 0,28 |  |
| ENSAPLG00000009205 | MAP3K8 | KB742630.1 | 69094 | intron | 8866 | 0,95 | 1,24 | 0,014 | 1,22 | 0,25 |  |
| ENSAPLG00000009205 | MAP3K8 | KB742630.1 | 70684 | intron | 8877 | 0,91 | 1,02 | 0,022 | 1,16 | 0,25 |  |
| ENSAPLG00000009205 | MAP3K8 | KB742630.1 | 71390 | intron | 8883 | 0,95 | 1,29 | 0,013 | 1,29 | 0,26 |  |
| ENSAPLG00000009929 | TLR-15 | KB743040.1 | 2572409 | exon | 9163 | 0,99 | 2,28 | 0,001 | 1,87 | 0,33 | synonymous |
| ENSAPLG00000009929 | TLR-15 | KB743040.1 | 2574035 | exon | 9183 | 0,86 | 0,77 | 0,042 | 1,38 | 0,28 | synonymous |
| ENSAPLG00000009929 | TLR-15 | KB743040.1 | 2575048 | intron | 9203 | 0,82 | 0,66 | 0,052 | 1,29 | 0,26 |  |
| ENSAPLG00000009929 | TLR-15 | KB743040.1 | 2575432 | intron | 9207 | 0,68 | 0,33 | 0,097 | 1,04 | 0,24 |  |
| ENSAPLG00000009929 | TLR-15 | KB743040.1 | 2575778 | intron | 9212 | 0,93 | 1,12 | 0,018 | 1,46 | 0,28 |  |
| ENSAPLG00000009929 | TLR-15 | KB743040.1 | 2581687 | intron | 9333 | 0,77 | 0,52 | 0,069 | 1,22 | 0,26 |  |
| ENSAPLG00000009929 | TLR-15 | KB743040.1 | 2582034 | intron | 9342 | 0,69 | 0,34 | 0,092 | 1,02 | 0,23 |  |
| ENSAPLG00000009929 | TLR-15 | KB743040.1 | 2583536 | intron | 9356 | 0,83 | 0,70 | 0,049 | 1,28 | 0,26 |  |
| ENSAPLG00000009929 | TLR-15 | KB743040.1 | 2584150 | intron | 9368 | 0,68 | 0,33 | 0,099 | 0,84 | 0,21 |  |
| ENSAPLG00000009929 | TLR-15 | KB743040.1 | 2585694 | intron | 9398 | 0,76 | 0,51 | 0,070 | 1,21 | 0,26 |  |
| ENSAPLG00000009929 | TLR-15 | KB743040.1 | 2586358 | intron | 9402 | 0,77 | 0,53 | 0,066 | 1,22 | 0,26 |  |
| ENSAPLG00000009929 | TLR-15 | KB743040.1 | 2586635 | intron | 9410 | 0,83 | 0,69 | 0,051 | 1,29 | 0,27 |  |
| ENSAPLG00000009929 | TLR-15 | KB743040.1 | 2586797 | intron | 9412 | 0,86 | 0,79 | 0,040 | 1,39 | 0,28 |  |
| ENSAPLG00000009929 | TLR-15 | KB743040.1 | 2586862 | intron | 9414 | 0,78 | 0,55 | 0,059 | 1,23 | 0,26 |  |
| ENSAPLG00000009929 | TLR-15 | KB743040.1 | 2587065 | intron | 9418 | 0,86 | 0,79 | 0,039 | 1,40 | 0,28 |  |
| ENSAPLG00000009929 | TLR-15 | KB743040.1 | 2587107 | intron | 9419 | 0,87 | 0,82 | 0,033 | 1,41 | 0,28 |  |
| ENSAPLG00000009929 | TLR-15 | KB743040.1 | 2587288 | intron | 9422 | 0,86 | 0,80 | 0,038 | 1,40 | 0,28 |  |
| ENSAPLG00000009929 | TLR-15 | KB743040.1 | 2589835 | intron | 9460 | 0,81 | 0,62 | 0,056 | 1,27 | 0,26 |  |
| ENSAPLG00000011399 | TLR2a | KB743211.1 | 435033 | exon | 9969 | 0,68 | 0,33 | 0,094 | 0,81 | 0,21 | synonymous |
| ENSAPLG00000011477 | AKT3 | KB742963.1 | 440422 | intron | 10128 | 0,82 | 0,65 | 0,054 | 1,41 | 0,28 |  |
| ENSAPLG00000011477 | AKT3 | KB742963.1 | 448309 | intron | 10202 | 0,78 | 0,54 | 0,060 | 0,95 | 0,22 |  |
| ENSAPLG00000011477 | AKT3 | KB742963.1 | 451692 | intron | 10227 | 0,83 | 0,70 | 0,048 | 1,05 | 0,24 |  |
| ENSAPLG00000011932 | AvBD10 | KB744359.1 | 86136 | intron | 10815 | 0,91 | 0,98 | 0,024 | 1,24 | 0,26 |  |
| ENSAPLG00000011932 | AvBD10 | KB744359.1 | 86464 | exon | 10830 | 0,91 | 0,99 | 0,023 | 1,29 | 0,26 | synonymous |
| ENSAPLG00000012251 | AvBD2 | KB744359.1 | 111229 | intron | 10894 | 0,73 | 0,43 | 0,081 | 0,95 | 0,23 |  |
| ENSAPLG00000012251 | AvBD2 | KB744359.1 | 112426 | intron | 10919 | 0,75 | 0,47 | 0,073 | 0,96 | 0,23 |  |
| ENSAPLG00000012251 | AvBD2 | KB744359.1 | 112569 | exon | 10922 | 0,73 | 0,43 | 0,079 | 0,95 | 0,23 | synonymous |
| ENSAPLG00000012251 | AvBD2 | KB744359.1 | 113428 | intron | 10940 | 0,77 | 0,52 | 0,067 | 1,03 | 0,23 |  |
| ENSAPLG00000012287 | AvBD1 | KB744359.1 | 116085 | intron | 10949 | 0,86 | 0,80 | 0,036 | 1,20 | 0,25 |  |
| ENSAPLG00000011933 | TRAF3 | KB743197.1 | 271360 | intron | 11157 | 0,92 | 1,07 | 0,020 | 1,19 | 0,25 |  |
| ENSAPLG00000011933 | TRAF3 | KB743197.1 | 272176 | intron | 11163 | 1,00 | 3,22 | 0,000 | 1,61 | 0,30 |  |
| ENSAPLG00000011933 | TRAF3 | KB743197.1 | 272202 | intron | 11164 | 0,98 | 1,69 | 0,006 | 1,36 | 0,27 |  |
| ENSAPLG00000011933 | TRAF3 | KB743197.1 | 285264 | intron | 11266 | 0,86 | 0,80 | 0,037 | 1,14 | 0,25 |  |
| ENSAPLG00000011933 | TRAF3 | KB743197.1 | 286423 | intron | 11272 | 0,98 | 1,75 | 0,004 | 1,39 | 0,27 |  |
| ENSAPLG00000011933 | TRAF3 | KB743197.1 | 286633 | intron | 11274 | 0,69 | 0,35 | 0,091 | 0,84 | 0,21 |  |
| ENSAPLG00000011933 | TRAF3 | KB743197.1 | 287365 | intron | 11281 | 0,79 | 0,57 | 0,057 | 0,97 | 0,23 |  |
| ENSAPLG00000011933 | TRAF3 | KB743197.1 | 288013 | intron | 11286 | 0,98 | 1,79 | 0,003 | 1,48 | 0,29 |  |
| ENSAPLG00000011933 | TRAF3 | KB743197.1 | 292522 | exon | 11322 | 0,98 | 1,72 | 0,005 | 1,38 | 0,27 | synonymous |
| ENSAPLG00000011933 | TRAF3 | KB743197.1 | 292719 | intron | 11324 | 0,97 | 1,55 | 0,007 | 1,35 | 0,27 |  |
| ENSAPLG00000011933 | TRAF3 | KB743197.1 | 294681 | intron | 11337 | 0,90 | 0,96 | 0,026 | 1,15 | 0,25 |  |
| ENSAPLG00000011933 | TRAF3 | KB743197.1 | 294804 | intron | 11339 | 0,87 | 0,84 | 0,032 | 1,10 | 0,24 |  |
| ENSAPLG00000011933 | TRAF3 | KB743197.1 | 295405 | intron | 11341 | 0,98 | 1,71 | 0,005 | 1,37 | 0,27 |  |
| ENSAPLG00000013226 | STAT1 | KB743811.1 | 232316 | intron | 12674 | 0,99 | 2,05 | 0,002 | 1,57 | 0,30 |  |
| ENSAPLG00000013226 | STAT1 | KB743811.1 | 238616 | intron | 12754 | 0,68 | 0,33 | 0,096 | 0,86 | 0,22 |  |
| ENSAPLG00000013460 | MAPK9 | KB743857.1 | 228482 | intron | 12966 | 0,84 | 0,73 | 0,046 | 1,07 | 0,24 |  |
| ENSAPLG00000013460 | MAPK9 | KB743857.1 | 228530 | intron | 12968 | 0,96 | 1,41 | 0,009 | 1,33 | 0,27 |  |
| ENSAPLG00000013878 | NFKB1 | KB742932.1 | 448102 | intron | 13312 | 0,74 | 0,45 | 0,076 | 1,24 | 0,26 |  |
| ENSAPLG00000013878 | NFKB1 | KB742932.1 | 448168 | intron | 13314 | 0,73 | 0,43 | 0,078 | 1,23 | 0,26 |  |
| ENSAPLG00000013878 | NFKB1 | KB742932.1 | 462848 | intron | 13479 | 0,89 | 0,89 | 0,028 | 1,12 | 0,24 |  |
| ENSAPLG00000014351 | IKBKE | KB742563.1 | 644481 | intron | 13849 | 0,99 | 2,14 | 0,002 | 1,52 | 0,29 |  |
| ENSAPLG00000015091 | DDX58 | KB751155.1 | 115539 | intron | 14464 | 0,98 | 1,72 | 0,005 | 1,53 | 0,29 |  |
| ENSAPLG00000015091 | DDX58 | KB751155.1 | 117581 | intron | 14481 | 0,94 | 1,22 | 0,015 | 1,33 | 0,27 |  |
| ENSAPLG00000015091 | DDX58 | KB751155.1 | 119442 | intron | 14498 | 1,00 | 2,52 | 0,001 | 1,80 | 0,32 |  |
| ENSAPLG00000015091 | DDX58 | KB751155.1 | 121049 | intron | 14505 | 1,00 | 2,40 | 0,001 | 1,78 | 0,32 |  |
| ENSAPLG00000015091 | DDX58 | KB751155.1 | 121280 | intron | 14506 | 0,88 | 0,85 | 0,031 | 1,19 | 0,25 |  |
| ENSAPLG00000015091 | DDX58 | KB751155.1 | 125521 | intron | 14515 | 0,99 | 2,01 | 0,002 | 1,59 | 0,30 |  |
| ENSAPLG00000015091 | DDX58 | KB751155.1 | 126197 | exon | 14518 | 0,96 | 1,40 | 0,010 | 1,41 | 0,28 | synonymous |
| ENSAPLG00000015091 | DDX58 | KB751155.1 | 127447 | intron | 14521 | 1,00 | 2,62 | 0,001 | 1,81 | 0,33 |  |
| ENSAPLG00000015091 | DDX58 | KB751155.1 | 127967 | intron | 14524 | 1,00 | 2,55 | 0,001 | 1,79 | 0,32 |  |
| ENSAPLG00000015091 | DDX58 | KB751155.1 | 129194 | intron | 14526 | 1,00 | 2,66 | 0,001 | 1,79 | 0,32 |  |
| ENSAPLG00000015976 | Mx | KB743889.1 | 35539 | intron | 15523 | 0,99 | 1,87 | 0,003 | 1,38 | 0,27 |  |
| ENSAPLG00000015976 | Mx | KB743889.1 | 36145 | intron | 15537 | 0,95 | 1,30 | 0,013 | 1,24 | 0,26 |  |
| ENSAPLG00000015976 | Mx | KB743889.1 | 36266 | intron | 15541 | 0,95 | 1,24 | 0,014 | 1,24 | 0,26 |  |
| ENSAPLG00000015976 | Mx | KB743889.1 | 37293 | intron | 15555 | 1,00 | 3,40 | 0,000 | 1,56 | 0,29 |  |
| ENSAPLG00000015976 | Mx | KB743889.1 | 37354 | intron | 15556 | 1,00 | 3,40 | 0,000 | 1,54 | 0,29 |  |
| ENSAPLG00000015976 | Mx | KB743889.1 | 37528 | intron | 15558 | 1,00 | 2,62 | 0,001 | 1,48 | 0,28 |  |
| ENSAPLG00000015976 | Mx | KB743889.1 | 37777 | intron | 15561 | 1,00 | 2,52 | 0,001 | 1,46 | 0,28 |  |
| ENSAPLG00000015976 | Mx | KB743889.1 | 37817 | intron | 15563 | 1,00 | 2,74 | 0,000 | 1,47 | 0,28 |  |
| ENSAPLG00000015976 | Mx | KB743889.1 | 39121 | intron | 15579 | 0,82 | 0,66 | 0,053 | 1,14 | 0,25 |  |
| ENSAPLG00000015976 | Mx | KB743889.1 | 39446 | intron | 15584 | 0,96 | 1,35 | 0,011 | 1,33 | 0,27 |  |
| ENSAPLG00000015976 | Mx | KB743889.1 | 53057 | exon | 15628 | 0,98 | 1,61 | 0,007 | 1,42 | 0,28 | non-synonymous, I-V |
| ENSAPLG00000015976 | Mx | KB743889.1 | 60176 | intron | 15698 | 0,96 | 1,40 | 0,010 | 1,27 | 0,26 |  |
| ENSAPLG00000015976 | Mx | KB743889.1 | 60717 | intron | 15718 | 0,99 | 1,88 | 0,003 | 1,39 | 0,27 |  |
| ENSAPLG00000015976 | Mx | KB743889.1 | 61447 | intron | 15729 | 1,00 | 2,92 | 0,000 | 1,67 | 0,31 |  |
| ENSAPLG00000016065 | PIK3R5 | KB746093.1 | 435330 | intron | 15996 | 0,78 | 0,54 | 0,062 | 1,00 | 0,23 |  |
| ENSAPLG00000016065 | PIK3R5 | KB746093.1 | 439869 | intron | 16053 | 0,98 | 1,65 | 0,006 | 1,55 | 0,29 |  |
| ENSAPLG00000016448 | MAPK8 | KB743335.1 | 878617 | intron | 16683 | 0,74 | 0,47 | 0,075 | 0,95 | 0,23 |  |

^a^ TRIM27L = TRIM27-like

#### Table S23 Bed12 file used for extracting the protein coding region using getfasta in bedtools

| chrom | chrom  Start | chrom  End | name | score | strand | thick  Start | thick  End | itemRgb | block  Count | blockSizes | blockStarts |
| --- | --- | --- | --- | --- | --- | --- | --- | --- | --- | --- | --- |
| KB755733.1 | 15 | 177 | ENSAPLG00000000983 | 0 | + | 15 | 15 | 0 | 1 | 162 | 0 |
| KB742799.1 | 310496 | 313067 | ENSAPLG00000001279 | 0 | - | 310496 | 310496 | 0 | 1 | 2571 | 0 |
| KB742693.1 | 10679 | 10808 | ENSAPLG00000001297 | 0 | - | 10679 | 10679 | 0 | 1 | 129 | 0 |
| KB743248.1 | 82852 | 83458 | ENSAPLG00000001534 | 0 | + | 82852 | 82852 | 0 | 1 | 606 | 0 |
| KB743106.1 | 212158 | 212984 | ENSAPLG00000001925 | 0 | + | 212158 | 212158 | 0 | 2 | 673,20 | 0,806 |
| KB742937.1 | 243680 | 253980 | ENSAPLG00000002012 | 0 | - | 243680 | 243680 | 0 | 6 | 489,116,184,155,173,125 | 0,6298,7489,8412,9596,10175 |
| KB744909.1 | 5475 | 7938 | ENSAPLG00000002049 | 0 | + | 5475 | 5475 | 0 | 1 | 2463 | 0 |
| KB743510.1 | 62 | 12637 | ENSAPLG00000002168 | 0 | - | 62 | 62 | 0 | 13 | 298,177,35,83,80,72,115,48,30,109,59,130,119 | 0,694,1331,2180,2726,3349,3790,4876,5369,6573,7832,8262,12456 |
| KB744198.1 | 63011 | 65879 | ENSAPLG00000002299 | 0 | + | 63011 | 63011 | 0 | 3 | 178,160,300 | 0,2088,2568 |
| KB743510.1 | 17399 | 34232 | ENSAPLG00000002603 | 0 | - | 17399 | 17399 | 0 | 11 | 161,174,79,80,72,115,48,30,112,59,129 | 0,704,1234,4063,9746,10851,11433,11920,12543,13020,16704 |
| KB742594.1 | 899222 | 935792 | ENSAPLG00000002702 | 0 | - | 899222 | 899222 | 0 | 16 | 178,116,68,109,68,81,160,152,82,131,129,125,139,46,66,129 | 0,2642,3286,6148,12596,19927,22590,23367,24691,25187,27553,31491,32740,33732,34767,36441 |
| KB742989.1 | 95006 | 108877 | ENSAPLG00000002771 | 0 | - | 95006 | 95006 | 0 | 19 | 596,143,23,231,44,5,93,32,420,284,77,23,25,40,85,20,57,41,158 | 0,1615,1894,2154,2484,2662,3164,3225,4179,4179,5491,5928,7383,9901,10844,10954,12413,12670,13713 |
| KB743363.1 | 326 | 2528 | ENSAPLG00000002833 | 0 | - | 326 | 326 | 0 | 4 | 46,143,84,180 | 0,141,952,2022 |
| KB742619.1 | 3622445 | 3624837 | ENSAPLG00000003004 | 0 | - | 3622445 | 3622445 | 0 | 8 | 278,73,164,37,48,105,48,54 | 0,329,766,987,1289,1490,2185,2338 |
| KB742464.1 | 2071991 | 2076188 | ENSAPLG00000003319 | 0 | - | 2071991 | 2071991 | 0 | 8 | 627,60,74,409,177,257,175,117 | 0,1004,1090,1226,2137,2661,3196,4080 |
| KB742655.1 | 930473 | 931028 | ENSAPLG00000003430 | 0 | + | 930473 | 930473 | 0 | 2 | 87,453 | 0,102 |
| KB744477.1 | 683782 | 740675 | ENSAPLG00000003554 | 0 | + | 683782 | 683782 | 0 | 25 | 86,186,226,224,183,171,21,78,252,97,131,54,189,122,144,100,179,110,79,25,131,124,146,133,138 | 0,14552,19708,21527,25067,25498,25683,26548,31316,32043,32966,34928,35989,38617,40779,41830,44351,45019,45424,52605,52642,53243,54414,56106,56755 |
| KB743145.1 | 26642 | 33568 | ENSAPLG00000003568 | 0 | - | 26642 | 26642 | 0 | 5 | 243,160,63,118,74 | 0,407,1273,5333,6852 |
| KB744694.1 | 21805 | 26090 | ENSAPLG00000004139 | 0 | - | 21805 | 21805 | 0 | 2 | 3099,48 | 0,4237 |
| KB743204.1 | 2114253 | 2146062 | ENSAPLG00000004157 | 0 | - | 2114253 | 2114253 | 0 | 11 | 187,174,79,87,74,115,48,30,112,59,129 | 0,1007,2326,3175,5470,6194,11398,12796,18644,22467,31680 |
| KB742953.1 | 321037 | 421223 | ENSAPLG00000004376 | 0 | - | 321037 | 321037 | 0 | 4 | 292,131,66,84 | 0,1314,4861,100102 |
| KB743204.1 | 2155931 | 2174086 | ENSAPLG00000004462 | 0 | - | 2155931 | 2155931 | 0 | 11 | 447,174,79,80,72,115,48,30,112,59,144 | 0,4074,5653,9454,10391,10586,13080,13260,15719,17010,18011 |
| KB742640.1 | 386551 | 430950 | ENSAPLG00000004748 | 0 | - | 386551 | 386551 | 0 | 26 | 484,101,16,118,173,137,190,137,151,152,139,128,61,27,135,129,186,112,158,120,18,313,146,130,112,226 | 0,2352,2554,4295,5390,10197,11099,13093,13899,14661,16025,16996,19162,19240,20782,21415,22282,24097,25661,28543,30105,30837,32392,33040,33940,44173 |
| KB742448.1 | 1039591 | 1090140 | ENSAPLG00000004772 | 0 | + | 1039591 | 1039591 | 0 | 17 | 334,96,75,132,202,80,103,99,181,126,143,52,119,31,38,171,190 | 0,28290,29112,32164,33186,33516,43460,44273,44485,45136,46314,46724,46789,46992,47152,49738,50359 |
| KB743235.1 | 169962 | 175410 | ENSAPLG00000004845 | 0 | - | 169962 | 169962 | 0 | 5 | 533,53,129,90,94 | 0,1796,2712,3834,5354 |
| KB742588.1 | 458690 | 466549 | ENSAPLG00000004947 | 0 | - | 458690 | 458690 | 0 | 9 | 524,186,84,157,234,96,297,86,145 | 0,1190,2848,4393,5094,6321,7265,7616,7714 |
| KB744154.1 | 102076 | 110088 | ENSAPLG00000005139 | 0 | - | 102076 | 102076 | 0 | 9 | 29,180,61,110,92,181,132,94,90 | 0,319,515,578,2786,3507,4277,7824,7922 |
| KB743829.1 | 1072475 | 1080797 | ENSAPLG00000005392 | 0 | + | 1072475 | 1072475 | 0 | 4 | 151,278,187,1537 | 0,3671,5715,6785 |
| KB743432.1 | 425481 | 427431 | ENSAPLG00000005400 | 0 | - | 425481 | 425481 | 0 | 4 | 245,97,136,52 | 0,544,985,1898 |
| KB743912.1 | 233900 | 251452 | ENSAPLG00000005826 | 0 | - | 233900 | 233900 | 0 | 11 | 556,46,140,78,128,52,117,120,114,49,83 | 0,4033,6849,7848,8591,10754,11236,12097,13779,16274,17469 |
| KB742619.1 | 3962586 | 4019261 | ENSAPLG00000005856 | 0 | - | 3962586 | 3962586 | 0 | 10 | 63,125,183,72,166,139,59,130,170,66 | 0,9770,11489,23647,26875,28382,30035,32390,45956,56609 |
| KB744434.1 | 31919 | 41134 | ENSAPLG00000005913 | 0 | + | 31919 | 31919 | 0 | 4 | 46,342,312,134 | 0,1451,6324,9081 |
| KB743412.1 | 2014942 | 2015905 | ENSAPLG00000005928 | 0 | + | 2014942 | 2014942 | 0 | 2 | 90,795 | 0,168 |
| KB746795.1 | 112537 | 114574 | ENSAPLG00000005942 | 0 | - | 112537 | 112537 | 0 | 5 | 285,146,138,30,67 | 0,404,774,1433,1970 |
| KB743007.1 | 206856 | 212148 | ENSAPLG00000006016 | 0 | + | 206856 | 206856 | 0 | 7 | 48,124,162,230,150,33,165 | 0,271,587,1193,2404,4582,5127 |
| KB743228.1 | 670281 | 684011 | ENSAPLG00000006116 | 0 | + | 670281 | 670281 | 0 | 7 | 182,190,117,115,135,110,117 | 0,1821,7006,9185,10095,12066,13613 |
| KB744208.1 | 566465 | 568812 | ENSAPLG00000006147 | 0 | + | 566465 | 566465 | 0 | 7 | 16,74,108,42,96,147,18 | 0,198,1345,1558,1703,2162,2329 |
| KB743988.1 | 21710 | 25833 | ENSAPLG00000006203 | 0 | - | 21710 | 21710 | 0 | 4 | 392,351,184,192 | 0,2356,2815,3931 |
| KB744812.1 | 409766 | 420934 | ENSAPLG00000006379 | 0 | + | 409766 | 409766 | 0 | 19 | 120,70,89,90,125,108,130,195,109,124,152,62,110,50,100,148,128,91,528 | 0,914,2662,3236,3557,4009,4426,4961,5388,5862,6239,6494,7552,7922,8524,8798,9131,9961,10640 |
| KB742471.1 | 563929 | 592062 | ENSAPLG00000006669 | 0 | + | 563929 | 563929 | 0 | 10 | 2004,66,226,104,147,91,131,112,158,282 | 0,6277,6441,9909,11660,12542,16610,18622,22532,27851 |
| KB742606.1 | 295221 | 346517 | ENSAPLG00000006823 | 0 | - | 295221 | 295221 | 0 | 19 | 187,171,75,177,143,126,181,99,112,147,31,16,97,132,78,38,74,206,79 | 0,1609,2159,2579,3925,4829,5750,7542,9923,21464,23578,29733,41720,42911,48520,48847,48931,50966,51217 |
| KB742987.1 | 145083 | 157802 | ENSAPLG00000007148 | 0 | - | 145083 | 145083 | 0 | 11 | 138,78,128,52,117,120,114,49,70,33,91 | 0,1953,2635,3283,3567,4170,4610,6914,8196,8519,12628 |
| KB742931.1 | 1563028 | 1582485 | ENSAPLG00000007417 | 0 | + | 1563028 | 1563028 | 0 | 5 | 94,342,282,126,107 | 0,11281,14159,16461,19350 |
| KB744072.1 | 669953 | 692210 | ENSAPLG00000007876 | 0 | + | 669953 | 669953 | 0 | 7 | 99,109,113,77,119,581,168 | 0,3799,4725,13254,15373,17033,22089 |
| KB742386.1 | 415204 | 432269 | ENSAPLG00000008173 | 0 | + | 415204 | 415204 | 0 | 10 | 191,994,186,168,161,94,81,40,58,153 | 0,4779,5914,6124,13832,14586,15576,15773,16096,16912 |
| KB742794.1 | 82413 | 103591 | ENSAPLG00000008183 | 0 | - | 82413 | 82413 | 0 | 23 | 642,133,146,124,168,79,40,70,179,100,144,122,171,60,131,97,222,90,150,180,230,229,141 | 0,1228,1973,3816,4761,5775,6460,6504,6785,7838,8594,8898,9747,10075,11035,11673,14909,16376,16782,17657,18670,19444,21037 |
| KB744799.1 | 49078 | 71477 | ENSAPLG00000008445 | 0 | + | 49078 | 49078 | 0 | 20 | 87,141,130,182,161,111,180,197,59,92,102,79,122,77,40,102,97,107,72,303 | 0,1066,1979,2688,3986,5570,7812,9547,12240,13384,13915,15476,16445,16682,17090,19378,19580,20732,21203,22096 |
| KB745027.1 | 25370 | 31584 | ENSAPLG00000008655 | 0 | - | 25370 | 25370 | 0 | 12 | 42,77,64,49,68,168,60,177,92,64,64,155 | 0,554,1168,1568,1751,2198,2590,2832,3157,3668,5651,6059 |
| KB742391.1 | 217674 | 223928 | ENSAPLG00000008976 | 0 | - | 217674 | 217674 | 0 | 4 | 733,1826,192,453 | 0,1311,4762,5801 |
| KB742414.1 | 396396 | 403983 | ENSAPLG00000009092 | 0 | + | 396396 | 396396 | 0 | 6 | 82,282,127,200,146,126 | 0,2075,3855,5009,6032,7461 |
| KB743110.1 | 86023 | 103854 | ENSAPLG00000009156 | 0 | - | 86023 | 86023 | 0 | 6 | 3535,91,153,183,166,17 | 0,12128,13437,14929,17504,17814 |
| KB742630.1 | 58049 | 72273 | ENSAPLG00000009205 | 0 | + | 58049 | 58049 | 0 | 7 | 336,177,262,107,153,247,464 | 0,3131,4823,7778,11165,11942,13760 |
| KB742811.1 | 451576 | 475770 | ENSAPLG00000009391 | 0 | + | 451576 | 451576 | 0 | 5 | 140,73,112,85,88 | 0,23164,23707,24106,24106 |
| KB742489.1 | 1579334 | 1580073 | ENSAPLG00000009793 | 0 | + | 1579334 | 1579334 | 0 | 3 | 73,109,91 | 0,358,648 |
| KB742811.1 | 483422 | 485503 | ENSAPLG00000009838 | 0 | - | 483422 | 483422 | 0 | 3 | 671,112,207 | 0,1304,1874 |
| KB743040.1 | 2572219 | 2591289 | ENSAPLG00000009929 | 0 | - | 2572219 | 2572219 | 0 | 3 | 2073,425,28 | 0,2109,19042 |
| KB745096.1 | 3854 | 7596 | ENSAPLG00000010090 | 0 | - | 3854 | 3854 | 0 | 7 | 166,174,171,153,1733,31,110 | 0,264,640,1151,1457,3371,3632 |
| KB752419.1 | 1321 | 3057 | ENSAPLG00000010194 | 0 | - | 1321 | 1321 | 0 | 3 | 94,181,133 | 0,833,1603 |
| KB744072.1 | 1085548 | 1111688 | ENSAPLG00000010913 | 0 | - | 1085548 | 1085548 | 0 | 21 | 183,94,191,162,150,257,225,124,117,221,211,227,18,94,38,62,6,22,24,157,456 | 0,281,1889,2557,3622,5249,7150,7476,8114,9623,12385,13591,14192,17197,17831,18198,19087,19175,20629,23023,25684 |
| KB742730.1 | 790980 | 794030 | ENSAPLG00000011028 | 0 | - | 790980 | 790980 | 0 | 5 | 761,261,89,211,117 | 0,1257,1638,2240,2933 |
| KB743016.1 | 255174 | 261771 | ENSAPLG00000011045 | 0 | - | 255174 | 255174 | 0 | 10 | 146,2,62,65,214,125,52,78,147,30 | 0,810,1465,2335,3113,4789,5009,5534,6274,6567 |
| KB742682.1 | 766664 | 775159 | ENSAPLG00000011237 | 0 | - | 766664 | 766664 | 0 | 7 | 900,155,154,146,176,136,58 | 0,3166,4115,5697,7636,8257,8437 |
| KB742685.1 | 132402 | 134032 | ENSAPLG00000011284 | 0 | + | 132402 | 132402 | 0 | 5 | 180,309,255,9,27 | 0,311,635,1461,1603 |
| KB742682.1 | 794344 | 807422 | ENSAPLG00000011322 | 0 | - | 794344 | 794344 | 0 | 11 | 225,155,154,155,203,115,142,158,194,121,49 | 0,2638,4955,5894,7184,8207,9275,10334,11086,12749,13029 |
| KB742682.1 | 837693 | 848168 | ENSAPLG00000011364 | 0 | - | 837693 | 837693 | 0 | 8 | 684,119,163,173,164,130,48,43 | 0,2414,2968,4385,6424,6764,7225,10432 |
| KB743211.1 | 424316 | 426689 | ENSAPLG00000011397 | 0 | + | 424316 | 424316 | 0 | 1 | 2373 | 0 |
| KB743211.1 | 432864 | 435216 | ENSAPLG00000011399 | 0 | + | 432864 | 432864 | 0 | 1 | 2352 | 0 |
| KB742963.1 | 423820 | 483138 | ENSAPLG00000011477 | 0 | - | 423820 | 423820 | 0 | 12 | 39,88,215,129,123,69,66,132,145,112,147,25 | 0,507,3263,20457,22528,28788,30506,35065,43825,45445,58801,59293 |
| KB742907.1 | 23133 | 54201 | ENSAPLG00000011503 | 0 | + | 23133 | 23133 | 0 | 20 | 62,115,70,89,90,125,108,136,195,103,124,152,62,110,50,97,148,137,100,27 | 0,1101,3315,5784,6526,6820,7912,10026,11150,11665,12838,16149,17416,20428,22630,23426,24361,27212,28821,31041 |
| KB744359.1 | 100428 | 100518 | ENSAPLG00000011572 | 0 | - | 100428 | 100428 | 0 | 1 | 90 | 0 |
| KB744335.1 | 16860 | 28174 | ENSAPLG00000011675 | 0 | - | 16860 | 16860 | 0 | 16 | 1160,171,72,177,140,126,181,99,106,86,202,132,63,134,41,270 | 0,1473,1832,2141,2533,3123,3442,3794,3999,4417,4606,5009,6110,6670,7431,11044 |
| KB743177.1 | 1054877 | 1057794 | ENSAPLG00000011754 | 0 | + | 1054877 | 1054877 | 0 | 3 | 126,147,192 | 0,296,2725 |
| KB744359.1 | 50831 | 51892 | ENSAPLG00000011919 | 0 | - | 50831 | 50831 | 0 | 4 | 119,119,61,61 | 0,0,1000,1000 |
| KB744359.1 | 57517 | 58188 | ENSAPLG00000011922 | 0 | - | 57517 | 57517 | 0 | 2 | 149,49 | 0,622 |
| KB744359.1 | 63129 | 65532 | ENSAPLG00000011923 | 0 | - | 63129 | 63129 | 0 | 6 | 454,454,129,129,58,58 | 0,0,1211,1211,2345,2345 |
| KB744359.1 | 86043 | 86503 | ENSAPLG00000011932 | 0 | + | 86043 | 86043 | 0 | 4 | 82,82,146,146 | 0,0,314,314 |
| KB743197.1 | 270351 | 297934 | ENSAPLG00000011933 | 0 | - | 270351 | 270351 | 0 | 10 | 949,175,141,93,75,81,168,105,52,242 | 0,2170,7327,12126,14035,16819,21006,22086,24484,27341 |
| KB745726.1 | 2980 | 13212 | ENSAPLG00000012022 | 0 | - | 2980 | 2980 | 0 | 27 | 177,119,101,83,98,154,152,40,60,128,88,177,107,198,40,103,52,70,107,48,90,260,164,48,76,124,40 | 0,1025,1336,1472,1867,2247,2521,2951,3029,3532,3749,3933,4422,4609,4924,5556,5756,5835,6161,6281,6561,6704,7243,7720,7784,8936,10192 |
| KB743412.1 | 1069 | 20199 | ENSAPLG00000012047 | 0 | + | 1069 | 1069 | 0 | 20 | 176,343,174,158,124,187,107,147,88,128,136,152,151,95,193,125,173,118,111,698 | 0,711,2564,3515,4358,5201,7367,8736,9671,10307,10941,11509,12470,13831,14408,15213,16170,16806,17279,18432 |
| KB744359.1 | 94513 | 96095 | ENSAPLG00000012056 | 0 | + | 94513 | 94513 | 0 | 2 | 70,134 | 0,1448 |
| KB742551.1 | 286668 | 287303 | ENSAPLG00000012088 | 0 | + | 286668 | 286668 | 0 | 2 | 387,219 | 0,416 |
| KB744359.1 | 105818 | 106943 | ENSAPLG00000012239 | 0 | + | 105818 | 105818 | 0 | 3 | 58,127,13 | 0,428,1112 |
| KB744359.1 | 110443 | 113568 | ENSAPLG00000012251 | 0 | + | 110443 | 110443 | 0 | 8 | 57,57,79,79,121,121,228,228 | 0,0,1173,1173,2073,2073,2897,2897 |
| KB742451.1 | 1774292 | 1786587 | ENSAPLG00000012255 | 0 | - | 1774292 | 1774292 | 0 | 15 | 244,159,63,142,26,19,15,110,115,65,161,198,146,85,76 | 0,333,3460,4388,4809,5370,6524,6577,8741,9155,10432,11153,11670,11932,12219 |
| KB744359.1 | 116018 | 117844 | ENSAPLG00000012287 | 0 | - | 116018 | 116018 | 0 | 6 | 13,13,127,127,58,58 | 0,0,1392,1392,1768,1768 |
| KB744359.1 | 116387 | 123049 | ENSAPLG00000012288 | 0 | - | 116387 | 116387 | 0 | 2 | 89,115 | 0,6547 |
| KB742966.1 | 2107269 | 2121829 | ENSAPLG00000012380 | 0 | + | 2107269 | 2107269 | 0 | 8 | 225,126,100,149,26,31,124,2248 | 0,1345,1759,2986,3391,6557,8697,12312 |
| KB819788.1 | 511 | 1080 | ENSAPLG00000012383 | 0 | - | 511 | 511 | 0 | 2 | 88,194 | 0,375 |
| KB742693.1 | 6739 | 7818 | ENSAPLG00000012465 | 0 | - | 6739 | 6739 | 0 | 3 | 16,127,102 | 0,333,977 |
| KB742693.1 | 18788 | 19108 | ENSAPLG00000012580 | 0 | - | 18788 | 18788 | 0 | 2 | 128,58 | 0,262 |
| KB743815.1 | 116839 | 122803 | ENSAPLG00000012625 | 0 | - | 116839 | 116839 | 0 | 5 | 10,15,2256,167,90 | 0,164,1289,4872,5874 |
| KB742619.1 | 2217223 | 2219608 | ENSAPLG00000012692 | 0 | + | 2217223 | 2217223 | 0 | 4 | 100,136,84,31 | 0,539,1476,2354 |
| KB742619.1 | 2233586 | 2236693 | ENSAPLG00000012697 | 0 | + | 2233586 | 2233586 | 0 | 4 | 96,136,84,852 | 0,863,1596,2255 |
| KB742884.1 | 385355 | 387613 | ENSAPLG00000012752 | 0 | - | 385355 | 385355 | 0 | 6 | 129,119,375,130,122,166 | 0,224,493,1196,1455,2092 |
| KB744049.1 | 12820 | 15312 | ENSAPLG00000012768 | 0 | - | 12820 | 12820 | 0 | 7 | 447,106,166,219,156,123,138 | 0,526,747,1442,1739,2010,2354 |
| KB820844.1 | 0 | 1135 | ENSAPLG00000012773 | 0 | - | 0 | 0 | 0 | 3 | 213,115,140 | 0,541,995 |
| KB744042.1 | 347537 | 351885 | ENSAPLG00000012876 | 0 | + | 347537 | 347537 | 0 | 4 | 240,69,180,919 | 0,2175,2607,3429 |
| KB743644.1 | 1765832 | 1788018 | ENSAPLG00000012924 | 0 | - | 1765832 | 1765832 | 0 | 25 | 1156,152,118,171,79,122,107,172,104,165,82,125,49,17,135,162,153,33,106,86,246,251,210,415,352 | 0,1843,2146,2775,3581,4186,4746,5191,6174,6843,7495,7932,8317,9924,11788,12031,12031,12736,13550,16696,17680,19203,20060,21834,21834 |
| KB742686.1 | 50687 | 114279 | ENSAPLG00000013074 | 0 | + | 50687 | 50687 | 0 | 11 | 90,131,175,120,120,52,128,78,149,46,939 | 0,539,19226,29465,38432,40641,41147,55809,56837,61518,62653 |
| KB743181.1 | 355200 | 359586 | ENSAPLG00000013187 | 0 | - | 355200 | 355200 | 0 | 14 | 525,97,191,162,150,213,105,90,114,117,59,96,202,168 | 0,800,1117,1654,2058,2387,2671,2793,3095,3285,3550,3644,3912,4218 |
| KB743811.1 | 230743 | 245451 | ENSAPLG00000013226 | 0 | + | 230743 | 230743 | 0 | 18 | 128,145,99,96,79,92,152,159,93,60,30,94,42,84,99,136,56,99 | 0,753,2090,4068,4344,5399,6092,6528,7966,8558,9785,9897,10848,11385,11638,12238,14150,14609 |
| KB742542.1 | 882906 | 885554 | ENSAPLG00000013262 | 0 | - | 882906 | 882906 | 0 | 4 | 136,76,192,136 | 0,572,1213,2512 |
| KB743857.1 | 225454 | 241776 | ENSAPLG00000013460 | 0 | + | 225454 | 225454 | 0 | 12 | 178,130,59,139,166,72,183,125,64,72,12,143 | 0,4551,5386,6124,7436,8851,12932,13786,14359,15321,15951,16179 |
| KB742537.1 | 865620 | 875533 | ENSAPLG00000013488 | 0 | - | 865620 | 865620 | 0 | 7 | 882,78,72,159,160,232,73 | 0,1699,3162,3817,4604,9458,9840 |
| KB742811.1 | 820235 | 823436 | ENSAPLG00000013556 | 0 | + | 820235 | 820235 | 0 | 7 | 359,104,177,235,153,110,93 | 0,646,980,1390,1751,2641,3108 |
| KB743810.1 | 291671 | 294699 | ENSAPLG00000013558 | 0 | - | 291671 | 291671 | 0 | 7 | 161,62,100,144,126,82,30 | 0,246,1227,1547,1793,2520,2998 |
| KB742932.1 | 441354 | 484880 | ENSAPLG00000013878 | 0 | + | 441354 | 441354 | 0 | 23 | 54,85,41,99,149,164,159,105,92,139,123,96,195,142,115,202,170,103,122,70,173,175,188 | 0,1195,9472,11970,19072,20680,22096,23271,25232,25903,28464,29433,30451,32791,35853,38031,39255,40277,41241,41708,42122,42433,43338 |
| KB744100.1 | 74343 | 78435 | ENSAPLG00000014072 | 0 | - | 74343 | 74343 | 0 | 6 | 313,210,123,1409,617,103 | 0,432,1167,1438,3280,3989 |
| KB742563.1 | 635377 | 644771 | ENSAPLG00000014351 | 0 | + | 635377 | 635377 | 0 | 20 | 84,141,130,182,168,296,203,69,88,49,55,56,113,77,40,102,97,113,72,31 | 0,815,1048,1570,2027,2507,2983,3343,3553,4150,4856,4931,5074,5337,5637,6231,7591,8181,8562,9363 |
| KB744239.1 | 110072 | 112303 | ENSAPLG00000014359 | 0 | - | 110072 | 110072 | 0 | 2 | 1340,844 | 0,1387 |
| KB742452.1 | 22264 | 44368 | ENSAPLG00000014377 | 0 | + | 22264 | 22264 | 0 | 4 | 63,1032,729,249 | 0,20799,20961,21855 |
| KB744325.1 | 174534 | 179257 | ENSAPLG00000014394 | 0 | + | 174534 | 174534 | 0 | 3 | 376,198,269 | 0,2452,4454 |
| KB742452.1 | 62285 | 66328 | ENSAPLG00000014631 | 0 | - | 62285 | 62285 | 0 | 8 | 194,187,40,211,109,175,84,131 | 0,947,2084,2205,2530,3179,3817,3912 |
| KB750090.1 | 2812 | 4757 | ENSAPLG00000014726 | 0 | - | 2812 | 2812 | 0 | 5 | 8,637,402,246,456 | 0,185,826,1239,1489 |
| KB742719.1 | 55430 | 60677 | ENSAPLG00000014872 | 0 | - | 55430 | 55430 | 0 | 9 | 136,502,136,47,15,57,139,106,305 | 0,437,1778,2420,2724,2825,2983,4250,4942 |
| KB745312.1 | 69808 | 71132 | ENSAPLG00000014996 | 0 | + | 69808 | 69808 | 0 | 1 | 1324 | 0 |
| KB751155.1 | 114374 | 133540 | ENSAPLG00000015091 | 0 | + | 114374 | 114374 | 0 | 17 | 204,151,16,104,111,154,256,163,102,158,136,149,91,171,152,147,297 | 0,1181,1425,2688,3639,4840,5868,6707,7964,10169,10651,11674,13183,14042,15826,17324,18869 |
| KB744410.1 | 60904 | 80777 | ENSAPLG00000015131 | 0 | + | 60904 | 60904 | 0 | 12 | 238,146,137,154,87,139,114,112,145,223,163,248 | 0,876,12225,12532,13319,13897,14575,17129,17343,17927,18466,19625 |
| KB742719.1 | 78528 | 86721 | ENSAPLG00000015244 | 0 | - | 78528 | 78528 | 0 | 10 | 142,505,112,89,72,16,89,136,100,296 | 0,668,1904,3479,4690,5897,6354,7217,7660,7897 |
| KB743404.1 | 1374523 | 1392704 | ENSAPLG00000015420 | 0 | + | 1374523 | 1374523 | 0 | 10 | 164,154,138,229,150,77,91,573,153,751 | 0,1430,2070,4550,5925,7234,10324,12250,16925,17430 |
| KB742800.1 | 574302 | 596101 | ENSAPLG00000015480 | 0 | + | 574302 | 574302 | 0 | 10 | 213,147,78,52,125,208,65,62,46,915 | 0,2381,5600,6215,13416,16381,17237,18932,19862,20884 |
| KB743889.1 | 32452 | 62323 | ENSAPLG00000015976 | 0 | + | 32452 | 32452 | 0 | 14 | 26,319,196,138,155,139,199,79,123,142,159,77,240,531 | 0,2678,4447,6108,7644,9423,20554,22179,23234,23964,24683,25559,27087,29340 |
| KB746093.1 | 418889 | 441854 | ENSAPLG00000016065 | 0 | - | 418889 | 418889 | 0 | 21 | 142,113,83,94,77,94,126,126,154,254,404,60,84,43,114,175,70,139,69,101,103 | 0,1217,1540,3036,3656,5019,5763,6164,6450,7004,7271,7711,8489,8835,9154,9944,11641,13572,14434,15790,22862 |
| KB742736.1 | 655447 | 676020 | ENSAPLG00000016230 | 0 | + | 655447 | 655447 | 0 | 10 | 188,79,99,162,75,75,378,178,149,219 | 0,780,1444,8905,11863,13693,14499,15823,19454,20354 |
| KB743761.1 | 139 | 5098 | ENSAPLG00000016235 | 0 | - | 139 | 139 | 0 | 10 | 138,123,41,25,66,132,173,29,178,46 | 0,299,758,862,1119,1504,1726,2584,2980,4913 |
| KB743650.1 | 139359 | 175814 | ENSAPLG00000016314 | 0 | - | 139359 | 139359 | 0 | 14 | 1611,103,88,215,129,126,69,66,132,148,112,129,7,42 | 0,2301,7065,14884,19447,20826,21679,23565,23775,25616,28608,35323,36287,36413 |
| KB743335.1 | 859285 | 881206 | ENSAPLG00000016448 | 0 | - | 859285 | 859285 | 0 | 11 | 146,78,64,125,183,78,166,139,59,130,122 | 0,582,3957,4978,5626,8246,12707,17520,17782,19061,21799 |

chrom - The name of the scaffold.

chromStart - The starting position of the feature in the chromosome or scaffold.

chromEnd - The ending position of the feature in the chromosome or scaffold.

name - Defines the name of the BED line.

score - A score between 0 and 1000.

strand - Defines the strand. Either "+" or "-".

thickStart – NA, in our case starting position of the gene

thickEnd – NA, in our case starting position of the gene

itemRgb - NA, in our case set to 0

blockCount - The number of blocks (exons) in the BED line.

blockSizes - A comma-separated list of the block sizes. The number of items in this list should correspond to *blockCount*.

blockStarts - A comma-separated list of block starts. All of the *blockStart* positions should be calculated relative to *chromStart*. The number of items in this list should correspond to *blockCount*.

#### Table S24 shows individuals for which sequencing data was lacking for certain immune genes in wild mallards, farm mallards and Pekin ducks. These individuals were all excluded from the analyses for the respective gene.

| Ensembl ID | Gene ID | No. individuals missing data | Individuals missing data |
| --- | --- | --- | --- |
| ENSAPLG00000000983 | cathelicidinlike | 6 | ESDO045, ESDO111, FARM019, GLNU021, SEOB005, SEOB013 |
| ENSAPLG00000001534 | PML | 1 | Pekin010 |
| ENSAPLG00000005400 | CCL19 | 1 | Pekin010 |
| ENSAPLG00000006203 | IRF5 | 5 | ESDO111, GLNU002, GLNU008, GLIS009, SEOB017 |
| ENSAPLG00000011675 | PIK3R2 | 8 | CARM019, FARM016, FARM019, Pekin012, Pekin025, SEOB003, SEOB005, SEOB017 |
| ENSAPLG00000013558 | CD40 | 9 | FARM011, FARM017, GLIS002, Pekin010, Pekin017, Pekin024, Pekin025, SEOB006, SEOB011 |
| ENSAPLG00000013187 | DHX58 | 24 | CARM013, CARM015, CASL001, ESDO006, ESDO045, ESDO053, ESDO092, ESDO108, FARM012, FARM019, FARM029, GLIS002, GLNU002, GLNU004, GLNU007, Pekin010, Pekin022, SEOB003, SEOB005, SEOB006, SEOB009, SEOB011, SEOB012, SEOB017 |
| ENSAPLG00000016235 | AKT2 | 1 | FARM019 |
| ENSAPLG00000006823 | PIK3R3 | 22 | CARM002, CARM013, CARM015, CARM018, ESDO021, ESDO092, FARM011, FARM017, FARM019, GLIS009, GLNU004, Pekin010, Pekin012, Pekin013, Pekin015, Pekin022, Pekin025, Pekin027, SEOB003, SEOB005, SEOB007, SEOB008 |
| ENSAPLG00000010194 | MYD88 | 3 | Pekin010, Pekin022, SEOB007 |
| ENSAPLG00000012022 | TYK2 | 10 | CARM001, ESDO006, FARM001, FARM005, FARM019, FARM020, Pekin010, Pekin012, SEOB017, SEOB018 |
| ENSAPLG00000012255 | IRAK4 | 12 | CASL001, CARM015, CARM017, CARM019, ESDO092, ESDO110, FARM012, FARM017, Pekin010, SEOB010, SEOB003, SEOB007 |

CARM, CASL = wild mallard from Canada, ESDO = wild mallard from Spain, GLIS and GLNU = wild mallard from Greenland, SEOB = wild mallard from Sweden, FARM = Farm mallard, Pekin = Pekin duck

#### Table S25 shows individuals for which sequencing data was lacking for certain immune genes in Anas crecca, Anas Penelope, Anas americana, Aythya ferina, Aythya fuligula. These individuals were all excluded from the analyses for the respective gene

| Ensembl ID | Gene ID | No. individuals missing data | Individuals with more than 25% missing data |
| --- | --- | --- | --- |
| ENSAPLG00000000983 | cathelicidinlike | 3 | CSANAM004, CSANPE002, CSAYFU005 |
| ENSAPLG00000006203 | IRF5 | 2 | CSAYFU005, CSAYFE005 |
| ENSAPLG00000012022 | TYK2 | 9 | CSANAM004, CSANAM007, CSANCR001, CSANCR007, CSAYFE003, CSAYFE005, CSAYFE006, CSAYFU001, CSAYFU002 |
| ENSAPLG00000012255 | IRAK4 | 2 | CSANAM007, CSANCR002 |
| ENSAPLG00000013187 | DHX58 | 3 | CSANPE001, CSAYFE003, CSAYFE005 |
| ENSAPLG00000014726 | TLR21 | 7 | CSANCR001, CSANCR007, CSANPE001, CSANPE002, CSANPE003, CSAYFE006, CSAYFU001 |

CSANAM = *Anas americana,* CSANCR = *Anas crecca,* CSANPE = *Anas penelope,* CSAYFE = *Aythya ferina,* CSAYFU = *Aythya fuligula*

#### Table S26 shows individuals for which more than 25% of the nucleotides of certain immune genes were missing in wild mallards, farm mallards and Pekin ducks. These individuals were all excluded from the interspecies analyses for the respective gene.

| Ensembl ID | Gene ID | No. individuals missing data | Individuals with more than 25% missing data |
| --- | --- | --- | --- |
| ENSAPLG00000000983 | cathelicidinlike | 26 | CARM008, CARM009, CARM017, CARM019, CARM020, ESDO021, ESDO053, ESDO108, ESDO110, ESDO115, FARM005, FARM011, FARM029, GLIS002, GLIS009, GLNU009, GLNU002, GLNU003, GLNU015, Pekin010, Pekin012, SEOB003, SEOB008, SEOB011, SEOB016, SEOB018 |
| ENSAPLG00000001534 | PML | 3 | CARM002, FARM029, SEOB017 |
| ENSAPLG00000001925 | TIRAP | 3 | CARM019, FARM019, Pekin010 |
| ENSAPLG00000003319 | MAVS | 22 | CARM002, CARM007, CARM019, ESDO028, ESDO085, ESDO111, FARM012, FARM017, FARM019, GLNU009, GLNU003, GLNU004, GLNU008, Pekin010, Pekin012, Pekin016, Pekin024, SEOB003, SEOB005, SEOB007, SEOB012, SEOB017 |
| ENSAPLG00000004947 | TRIM25 | 4 | FARM017, Pekin010, SEOB008, SEOB017 |
| ENSAPLG00000005139 | MYD88 | 1 | Pekin010 |
| ENSAPLG00000005400 | CCL19 | 44 | CARM001, CARM002, CARM003, CARM008, CARM009, CARM011, CARM013, CARM014, CARM015, ESDO038, ESDO053, ESDO071, ESDO092, ESDO095, ESDO105, ESDO108, ESDO110, ESDO111, ESDO115, FARM017, FARM027, GLIS001, GLIS002, GLNU009, GLNU002, GLNU003, GLNU005, GLNU007, GLNU019, Pekin002, Pekin012, Pekin013, Pekin024, Pekin025, SEOB003, SEOB005, SEOB006, SEOB007, SEOB010, SEOB011, SEOB013, SEOB014, SEOB016, SEOB017 |
| ENSAPLG00000005928 | JUN | 73 | CARM001, CARM002, CARM003, CARM009, CARM012, CARM013, CARM014, CARM015, CARM018, CARM019, CARM020, CASL001, ESDO006, ESDO028, ESDO038, ESDO045, ESDO051, ESDO053, ESDO071, ESDO085, ESDO092, ESDO105, ESDO108, ESDO110, ESDO115, FARM001, FARM003, FARM004, FARM005, FARM012, FARM017, FARM019, FARM020, FARM029, GLIS001, GLIS002, GLIS009, GLNU009, GLNU002, GLNU004, GLNU005, GLNU007, GLNU008, GLNU012, GLNU015, GLNU019, GLNU020, GLNU021, Pekin004, Pekin006, Pekin009, Pekin010, Pekin013, Pekin014, Pekin015, Pekin017, Pekin022, Pekin023, Pekin024, Pekin025, SEOB003, SEOB005, SEOB006, SEOB007, SEOB008, SEOB009, SEOB010, SEOB011, SEOB012, SEOB013, SEOB014, SEOB017, SEOB018 |
| ENSAPLG00000006203 | IRF5 | 38 | CARM001, CARM002, CARM009, CARM012, CARM017, CARM019, CARM020, ESDO006, ESDO053, ESDO071, ESDO085, ESDO092, ESDO108, FARM001, FARM003, FARM005, FARM014, FARM017, FARM023, FARM027, FARM029, GLIS001, GLNU005, GLNU011, GLNU015, GLNU020, Pekin002, Pekin006, Pekin010, Pekin012, Pekin016, Pekin017, Pekin023, SEOB003, SEOB011, SEOB013, SEOB016, SEOB019 |
| ENSAPLG00000008655 | LBP | 1 | Pekin010 |
| ENSAPLG00000009793 | CCL5 | 16 | CARM013, ESDO028, ESDO053, ESDO092, ESDO105, ESDO110, FARM019, FARM020, GLNU003, GLNU004, GLNU012, GLNU021, SEOB005, SEOB011, SEOB014, SEOB016 |
| ENSAPLG00000011284 | FOS | 76 | CARM001, CARM002, CARM008, CARM009, CARM012, CARM013, CARM014, CARM015, CARM017, CARM018, CARM019, CARM020, CASL001, ESDO006, ESDO028, ESDO038, ESDO045, ESDO051, ESDO053, ESDO085, ESDO092, ESDO095, ESDO105, ESDO108, ESDO110, ESDO115, FARM003, FARM004, FARM012, FARM014, FARM016, FARM017, FARM019, FARM020, FARM021, FARM023, FARM024, FARM027, FARM029, GLIS001, GLIS002, GLIS009, GLNU009, GLNU002, GLNU003, GLNU004, GLNU005, GLNU008, GLNU012, GLNU019, GLNU020, GLNU021, Pekin002, Pekin010, Pekin012, Pekin013, Pekin015, Pekin016, Pekin017, Pekin022, Pekin023, Pekin024, Pekin025, Pekin027, SEOB003, SEOB005, SEOB006, SEOB007, SEOB008, SEOB011, SEOB013, SEOB014, SEOB016, SEOB017, SEOB018, SEOB019 |
| ENSAPLG00000011675 | PIK3R2 | 28 | CARM009, CARM012, CARM017, CARM018, ESDO038, ESDO053, ESDO092, ESDO108, ESDO110, FARM003, FARM017, FARM020, FARM023, GLIS002, GLNU008, GLNU009, GLNU004, GLNU005, GLNU007, Pekin002, Pekin004, Pekin010, Pekin022, Pekin023, Pekin024, SEOB007, SEOB011, SEOB013 |
| ENSAPLG00000011754 | IL6 | 1 | FARM019 |
| ENSAPLG00000012465 | AvBD5 | 1 | CARM019 |
| ENSAPLG00000012768 | CTSK | 1 | Pekin010 |
| ENSAPLG00000013187 | DHX58 | 54 | CARM002, CARM003, CARM007, CARM009, CARM011, CARM012, CARM014, CARM018, CARM019, CARM020, ESDO021, ESDO028, ESDO038, ESDO051, ESDO071, ESDO085, ESDO095, ESDO105, ESDO111, ESDO115, FARM001, FARM003, FARM004, FARM005, FARM011, FARM014, FARM016, FARM017, FARM020, FARM023, FARM027, GLNU009, GLNU003, GLNU011, GLNU012, GLNU020, GLNU021, Pekin002, Pekin004, Pekin006, Pekin009, Pekin012, Pekin013, Pekin015, Pekin016, Pekin017, Pekin023, Pekin025, Pekin027, SEOB010, SEOB013, SEOB014, SEOB018, SEOB020 |
| ENSAPLG00000013556 | CASP1 | 1 | Pekin010 |
| ENSAPLG00000013558 | CD40 | 7 | ESDO006, FARM019, FARM029, Pekin012, Pekin015, SEOB005, SEOB017 |
| ENSAPLG00000014377 | CD14 | 2 | Pekin010, SEOB017 |
| ENSAPLG00000014377 | CD14 | 1 | Pekin010 |
| ENSAPLG00000014394 | TNFRSF6B | 9 | CARM002, FARM019, GLNU005, GLNU008, Pekin010, Pekin025, SEOB005, SEOB012, SEOB017 |
| ENSAPLG00000014631 | TMEM173 | 11 | ESDO053, FARM003, FARM017, FARM019, FARM029, GLNU015, Pekin010, Pekin027, SEOB003, SEOB005, SEOB017 |
| ENSAPLG00000014996 | IFITM3 | 6 | CARM003, CARM017, FARM019, Pekin012, SEOB005, SEOB017 |
| ENSAPLG00000016235 | AKT2 | 18 | CARM001, ESDO085, ESDO111, FARM003, FARM005, FARM011, FARM012, FARM016, FARM029, GLNU004, GLNU012, Pekin004, Pekin010, Pekin017, Pekin027, SEOB003, SEOB017, SEOB018 |

CARM, CASL = wild mallard from Canada, ESDO = wild mallard from Spain, GLIS and GLNU = wild mallard from Greenland, SEOB = wild mallard from Sweden, FARM = Farm mallard, Pekin = Pekin duck

#### Table S27 shows individuals for which more than 25% of the nucleotides of certain immune genes were missing in Anas crecca, Anas Penelope, Anas americana, Aythya ferina, Aythya fuligula. These individuals were all excluded from the interspecies analyses for the respective gene.

| Ensembl ID | Gene ID | No. individuals 25% missing data | Individuals with more than 25% missing data |
| --- | --- | --- | --- |
| ENSAPLG00000000983 | cathelicidinlike | 6 | CSANAM007, CSANCR001, CSANCR002, CSANCR003, CSAYFE005, CSAYFU002 |
| ENSAPLG00000001534 | PML | 1 | CSAYFU005 |
| ENSAPLG00000001925 | TIRAP | 1 | CSAYFE005 |
| ENSAPLG00000003319 | MAVS | 6 | CSANAM004, CSANCR003, CSANCR007, CSAYFE006, CSAYFU002, CSAYFU005 |
| ENSAPLG00000004947 | TRIM25 | 1 | CSAYFE003 |
| ENSAPLG00000005139 | MYD88 | 1 | CSAYFE003 |
| ENSAPLG00000005400 | CCL19 | 7 | CSANAM004, CSANAM007, CSANCR001, CSANCR002, CSANCR007, CSANPE002, CSAYFU001 |
| ENSAPLG00000006203 | IRF5 | 14 | CSANAM001, CSANAM004, CSANAM007, CSANCR001, CSANCR002, CSANCR003, CSANCR007, CSANPE001, CSANPE002, CSANPE003, CSAYFE003, CSAYFE006, CSAYFU001, CSAYFU002 |
| ENSAPLG00000009793 | CCL5 | 4 | CSANCR001, CSAYFU001, CSAYFU002, CSAYFU005 |
| ENSAPLG00000011675 | PIK3R2 | 5 | CSANCR007, CSANPE001, CSANPE002, CSAYFU001, CSAYFU005 |
| ENSAPLG00000012239 | AvBD7 | 6 | CSANAM001, CSANAM004, CSANAM007, CSANPE001, CSANPE002, CSANPE003 |
| ENSAPLG00000005928 | JUN | 12 | CSANAM001, CSANAM004, CSANCR001, CSANCR003, CSANPE001, CSANPE002, CSANPE003, CSAYFE003, CSAYFE005, CSAYFE006, CSAYFU002, CSAYFU005 |
| ENSAPLG00000011284 | FOS | 13 | CSANAM004, CSANAM007, CSANCR001, CSANCR002, CSANCR003, CSANCR007, CSANPE001, CSANPE002, CSAYFE003, CSAYFE005, CSAYFE006, CSAYFU001, CSAYFU005, |
| ENSAPLG00000012088 | SOCS3 | 14 | CSANAM004, CSANAM007, CSANCR001, CSANCR002, CSANCR007, CSANPE001, CSANPE002, CSANPE003, CSAYFE003, CSAYFE005, CSAYFE006, CSAYFU001, CSAYFU002, CSAYFU005 |
| ENSAPLG00000012239 | AvBD7 | 6 | CSANAM001, CSANCR003, CSANPE003, CSAYFE005, CSAYFU002, CSAYFU005 |
| ENSAPLG00000012752 | IRF7 | 16 | CSANAM001, CSANAM004, CSANAM007, CSANCR001, CSANCR002, CSANCR003, CSANCR007, CSANPE001, CSANPE002, CSANPE003, CSAYFE003, CSAYFE005, CSAYFE006, CSAYFU001, CSAYFU002, CSAYFU005 |
| ENSAPLG00000013187 | DHX58 | 10 | CSANAM001, CSANAM004, CSANAM007, CSANCR001, CSANCR002, CSANCR007, CSANPE002, CSANPE003, CSAYFE006, CSAYFU001 |
| ENSAPLG00000014072 | NLRX1 | 8 | CSANAM001, CSANAM004, CSANAM007, CSANCR002, CSANPE003, CSAYFE003, CSAYFE005, CSAYFU001 |
| ENSAPLG00000014394 | TNFRSF6B | 2 | CSANAM007, CSAYFE005 |
| ENSAPLG00000014631 | TMEM173 | 4 | CSANAM001, CSANAM007, CSANCR002, CSAYFE005 |
| ENSAPLG00000014726 | TLR21 | 5 | CSANAM001, CSANAM004, CSAYFE003, CSAYFE005, CSAYFU002 |
| ENSAPLG00000014996 | IFITM3 | 5 | CSANAM001, CSANAM007, CSANCR002, CSAYFE003, CSAYFU001 |
| ENSAPLG00000016235 | AKT2 | 11 | CSANAM001, CSANAM007, CSANCR001, CSANCR002, CSANPE001, CSANPE002, CSANPE003, CSAYFE003, CSAYFE006, CSAYFU001, CSAYFU002 |

CSANAM = *Anas americana,* CSANCR = *Anas crecca,* CSANPE = *Anas penelope,* CSAYFE = *Aythya ferina,* CSAYFU = *Aythya fuligula*

#### Table S28 VCFtools options used for filtering the vcf file

| Used options and values | Description from the VCFtools manual, https://vcftools.github.io/man_latest.html |
| --- | --- |
| --minQ 50 | Includes only sites with Quality value above this threshold. |
| --max-missing 0.5 | Exclude sites on the basis of the proportion of missing data (defined to be between 0 and 1, where 0 allows sites that are completely missing and 1 indicates no missing data allowed). |
| --maf 0.05 | Minor Allele Frequency greater than or equal to the "--maf" value. |
| --min-alleles 2 | Include only sites with a number of alleles greater than or equal to the "--min-alleles" value. |
| --minDP 3 | Includes only genotypes greater than or equal to the "--minDP" value. |
| --min-meanDP 20 | Includes only sites with mean depth values (over all included individuals) greater than or equal to the "--min-meanDP" value. |
| --missing-indv | Generates a file reporting the missingness on a per-individual basis. The file has the suffix ".imiss". |
| --missing-site | Generates a file reporting the missingness on a per-site basis. The file has the suffix ".lmiss". |
| --exclude-positions | Include or exclude a set of sites on the basis of a list of positions in a file. |
| --remove-indels | Include or exclude sites that contain an indel. For these options "indel" means any variant that alters the length of the REF allele. |

The --missing-site option was run separately for each mallard population, to estimate missing data for all loci in each population. We then removed all loci that had missing data in any of the mallard populations using the --exclude-positions option.

#### Table S29 Classification used for comparisons between different components of the innate immune signalling pathways.

| Functional group | Gene ID |
| --- | --- |
| detection | TLR5, TLR1-A, TLR7, TLR3, TLR-15, IFIH1, TLR2a, TLR4, DDX58, TLR2, TLR21 |
| signalling | TIRAP, IFNGR1, MAPK13, LITAF, MAPK14, MAP3K7, TRIM27 isoform1, TRIM27 isoform2, PIK3CB, RAC1, MAPK12, IRAK1BP1, MAPK11, JAK2, PIK3R1, LY96, TRIM25, MYD88, TRADD, MAP2K6, MAPK10, CD86, JUN, MAPK1, IRF5, IKBKB, PIK3CG, PIK3R3, MAP2K3, CD80, TANK, TAB2, PIK3CD, TBK1, LBP, TOLLIP, MAP3K8, NLRP12, MYD88, NFKBIA, MAP2K2, IFNGR2, IFNAR1, IFNAR2, AKT3, CHUK, PIK3R2, TRAF3, TYK2, JAK1, SOCS3, IRAK4, AZI2, CTSK, PIK3CA isoform1, PIK3CA isoform2, MAP2K4, STAT1, MAPK9, TRAF6, CASP1, CD40, NFKB1, CD14 isoform2, TNFRSF6B, TMEM173, CASP8, TAB1, CASP10, RIPK1, MAP2K1, PIK3R5, TRAF2, AKT2, AKT1, MAPK8, MAVS, IKBKE, TICAM1 |
| response | cathelicidinlike, AvBD4, PML, SPP1, IFN, CCL19, IL18, RSAD2, IL12A, IL12B, CCL24or4 isoform1, CCL24or4 isoform2, CCL5, CCL5, AvBD8, IL6, AvBD13, AvBD12, AvBD11, AvBD10, AvBD9, AvBD7, AvBD2, AvBD1, AvBD16=AvBD3a, CCL4, AvBD5, AvBD14, IL8-like, IL8-like, IFNG, IFITM3, Mx |

## Supplementary notes

#### Note S1. Statistics from the population comparison of nucleotide and amino acid diversity using a Kruskal‐Wallis rank sum test and a pairwise Wilcoxon tests with Benjamini Hochberg (BH) false discovery rate (FDR) correction

#Kruskal-Wallis non-parametric ANOVA

#Kruskal-Wallis, chi-squared = 34.547, df = 5, p-value = 1.852e-06

#Pairwise comparisons using Wilcoxon rank sum test, P value adjustment method: BH

| **Pairwise Wilcoxon tests nucleotide diversity** | Canada | Greenland | Spain | Sweden | Farm |
| --- | --- | --- | --- | --- | --- |
| Greenland | 0.34165 | - | - | - | - |
| Spain | 0.75778 | 0.58304 | - | - | - |
| Sweden | 0.75778 | 0.55678 | 0.93644 | - | - |
| Farm | 0.40238 | 0.89047 | 0.59191 | 0.58304 | - |
| Pekin | 7.1e-06 | 0.00110 | 3.3e-05 | 2.3e-05 | 0.00031 |

#Kruskal-Wallis non-parametric ANOVA

#Kruskal-Wallis chi-squared = 19.923, df = 5, p-value = 0.001292

#Pairwise comparisons using Wilcoxon rank sum test, P value adjustment method: BH

| **Pairwise Wilcoxon tests amino acid diversity** | Canada | Greenland | Spain | Sweden | Farm |
| --- | --- | --- | --- | --- | --- |
| Greenland | 0.45770 | - | - | - | - |
| Spain | 0.53839 | 0.88405 | - | - | - |
| Sweden | 0.81972 | 0.62312 | 0.76883 | - | - |
| Farm | 0.53839 | 0.86999 | 0.96914 | 0.76883 | - |
| Pekin | 0.00063 | 0.05028 | 0.01232 | 0.00209 | 0.00769 |

#### Note S2. Statistics from the immune function group comparisons using a Kruskal‐Wallis rank sum test and a pairwise Wilcoxon tests with Benjamini Hochberg (BH) false discovery rate (FDR) correction.

##### Nucleotide diversity in wild mallards

#Kruskal-Wallis non-parametric ANOVA

Kruskal-Wallis chi-squared = 1.6063, df = 2, p-value = 0.4479

#Pairwise comparisons using Wilcoxon rank sum test, P value adjustment method: BH

|  | detection | signalling |
| --- | --- | --- |
| signalling | 0.5 | - |
| response | 0.5 | 0.5 |

Number of genes in each category: detection 10, signalling 79, response 34 (Table S9 and S29).

1. Amino acid diversity in wild mallards

#Kruskal-Wallis non-parametric ANOVA

Kruskal-Wallis chi-squared = 6.0709, df = 2, p-value = 0.04805

#Pairwise comparisons using Wilcoxon rank sum test, P value adjustment method: BH

|  | detection | signalling |
| --- | --- | --- |
| signalling | 0.34 | - |
| response | 0.46 | 0.06 |

##### Number of genes in each category: detection 10, signalling 79, response 34 (Table S9 and S29)Average population divergence (Fst) among the wild mallard populations

#Kruskal-Wallis non-parametric ANOVA

Kruskal-Wallis chi-squared = 3.9061, df = 2, p-value = 0.1418

#Pairwise comparisons using Wilcoxon rank sum test, P value adjustment method: BH

|  | detection | signalling |
| --- | --- | --- |
| signalling | 0.21 | - |
| response | 0.21 | 0.30 |

Number of genes in each category: detection 10, signalling 79, response 34 (Table S9 and Figure S29).

##### Average population divergence (Fst) among the wild mallard populations when including nucleotides that lead to a non-synonymous change on the protein level

#Kruskal-Wallis non-parametric ANOVA

Kruskal-Wallis chi-squared = 5.3701, df = 2, p-value = 0.06822

#Pairwise comparisons using Wilcoxon rank sum test, P value adjustment method: BH

|  | detection | signalling |
| --- | --- | --- |
| signalling | 0.07 | - |
| response | 0.1 | 0.63 |

Number of genes in each category: detection 10, response 30, signalling 72 (Table S9 and S29).

##### Tajima’s D in wild mallards

#Kruskal-Wallis non-parametric ANOVA

Kruskal-Wallis chi-squared = 4.9686, df = 2, p-value = 0.08338

#Pairwise comparisons using Wilcoxon rank sum test, P value adjustment method: BH

|  | detection | signalling |
| --- | --- | --- |
| signalling | 0.30 | - |
| response | 0.11 | 0.11 |

Number of genes in each category: detection 10, response 34, signalling 79 (Table S9 and S29).

##### dN/dS estimated from a total of 26 species of waterfowl

#Kruskal-Wallis non-parametric ANOVA

#Kruskal-Wallis chi-squared = 32.084, df = 2, p-value = 1.079e-07

#Pairwise comparisons using Wilcoxon rank sum test, P value adjustment method: BH

|  | detection | signalling |
| --- | --- | --- |
| signalling | 0.00037 | - |
| response | 0.65564 | 2e-06 |

Number of genes in each category: detection 10, signalling 64, response 30 (Table S9 and S29).

ENSAPLG00000012383 was excluded from the comparison as it had a dN/dS of 999, which is likely a sign that the sequences are too divergent.

##### proportion of positively selected sites estimated from a total of 26 species of waterfowl

#Kruskal-Wallis non-parametric ANOVA

#Kruskal-Wallis chi-squared = 8.079, df = 2, p-value = 0.01761

#Pairwise comparisons using Wilcoxon rank sum test, P value adjustment method: BH

|  | detection | signalling |
| --- | --- | --- |
| signalling | 0.0094 | - |
| response | 0.1120 | 0.5474 |

Number of genes in each category: detection 10, signalling 64, response 31 (Table S16 and S29).

##### proportion of negatively selected sites per gene for the functional groups estimated from a total of 26 species of waterfowl

#Kruskal-Wallis non-parametric ANOVA

Kruskal-Wallis chi-squared = 0.04177, df = 2, p-value = 0.9793

#Pairwise comparisons using Wilcoxon rank sum test, P value adjustment method: BH

|  | detection | signalling |
| --- | --- | --- |
| signalling | 1 | - |
| response | 1 | 1 |

Number of genes in each category: detection 10, signalling 64, response 31 (Table S16 and S29).

## References

Jetz W, Thomas G, Joy J, Hartmann K, Mooers A. 2012. The global diversity of birds in space and time. *Nature* 491(7424):444.

Ottenburghs J, Megens H-J, Kraus RHS, Madsen O, van Hooft P, van Wieren SE, Crooijmans RPMA, Ydenberg RC, Groenen MAM, Prins HHT. 2016. A tree of geese: A phylogenomic perspective on the evolutionary history of True Geese. *Mol Phylogenet Evol* 101:303-313.

Ottenburghs J, Megens H-J, Kraus RHS, van Hooft P, van Wieren SE, Crooijmans RPMA, Ydenberg RC, Groenen MAM, Prins HHT. 2017. A history of hybrids? Genomic patterns of introgression in the True Geese. *BMC Evol Biol* 17(1):201.
